# Dual-additive enabled inorganic-rich solid electrolyte interphase for high-rate lithium negative electrodes

**DOI:** 10.1038/s41467-026-76653-7

**Published:** 2026-08-14

**Authors:** Yehui Wu, Xihao Wang, Shengchuang Du, Tiansheng Bai, Huijie Tian, Qunhui Yuan, Wanbao Wu, Jiawen Huang, Guanhua Lin, Xingjun Liu, Deping Li, Lijie Ci, Jingyu Lu

**Affiliations:** 1 School of Science, Harbin Institute of Technology (Shenzhen), Shenzhen, China; 2 State Key Laboratory of Precision Welding & Joining of Materials and Structures, School of Materials Science and Engineering, Harbin Institute of Technology (Shenzhen), Shenzhen, China; 3 Shenzhen Key Laboratory of Flexible Printed Electronics Technology and School of Materials Science and Engineering, Harbin Institute of Technology (Shenzhen), Shenzhen, China; 4 Changzhou Qianmu New Energy Co. Ltd, Changzhou, China; 5 School of Environmental and Chemical Engineering, Shanghai University, Shang, China; 6 Strait Laboratory of Flexible Electronics (SLoFE), Fujian Key Laboratory of Flexible Electronics, and Strait Institute of Flexible Electronics (SIFE, Future Technologies), Fujian Normal University, Fuzhou, China

**Keywords:** Batteries, Batteries

## Abstract

Constructing a robust, highly conductive solid electrolyte interphase is the key to unlock the high-rate potential of lithium negative electrodes for broad applications. Here we propose a simple yet effective strategy, by introducing a sulfur-containing compound and LiNO_3_ as dual additives into a conventional carbonate electrolyte. As an example, 3-sulfolene is found to enhance the dissolution of LiNO_3_, and it works with NO_3_^−^ to regulate the Li^+^-solvation structure and facilitate the formation of a robust while uniform solid electrolyte interphase rich in Li_3_N/Li_2_S/Li_2_O inorganics, enabling rapid Li^+^ transfer and high interfacial stability. Consequently, Li | |LiFePO_4_ cells achieve 81.7% capacity retention after 6,000 cycles at 80 C, while Li | |LiNi_0.8_Co_0.1_Mn_0.1_O_2_ cells maintain 81.3% capacity after 500 cycles at 10 C. Moreover, two pouch cells with specific energy exceeding 500 Wh kg^−1^ (calculated based on total mass of the pouch cells) retain 92.1% and 91.8% of initial capacities after 143 and 149 cycles (charged at 0.1 C and discharged at 0.3 C), respectively. This work provides a general dual-additive strategy to promote the formation of an inorganic-rich solid electrolyte interphase for high-rate lithium negative electrodes.

## Abstract (web summary)

Lithium metal anodes face challenges in carbonate electrolytes at high rates. Here, authors propose a dual-additive strategy using a sulfur-containing compound and LiNO3 to promote the formation of an inorganic-rich SEI, enabling stable high-rate cycling and pouch cells exceeding 500 Wh kg–1.

## Introduction

Lithium negative electrode (LNE), with the low standard redox potential ( − 3.04 V versus standard hydrogen electrode) and high theoretical specific capacity (3860 mAh g^−1^), has long been regarded as the ultimate alternative to intercalation-limited graphite negative electrodes, allowing lithium metal batteries (LMBs) to achieve a specific energy exceeding 500 Wh kg^−1^^1,2^. However, achieving long-lasting high-rate LNEs remains a huge challenge for practical applications^3^. When a LNE is operated at elevated current densities, the sluggish lithium-ion transport kinetics at the interphases exacerbates the uncontrolled dendritic Li growth, the accumulation of isolated dead Li (*i.e*., Li metal regions which are electronically disconnected from the current collector), and the irreversible electrolyte depletion, leading to an early failure of the battery^4,5^.

It has been well-established that, the quality of the solid electrolyte interphase (SEI), which serves as both the ionic conductor and mechanical barrier, is critical to the performance of a LNE^6,7^. However, in commercial carbonate electrolytes, carbonate molecules primarily occupy the first Li^+^-solvation sheath and undergo preferential reduction on LNEs, leading to an organic-dominated SEI with the low Li^+^ conductivity^8^. The resultant inhomogeneous Li^+^ flux across the SEI tends to trigger the nucleation and growth of Li dendrites from surface protrusions^9^. During the cycle process, such SEI is prone to experience continuous cracking and reformation, leading to the electrolyte consumption and reduction in cycle life of the LMB^10^. These limitations aggravate severely at high rates, blocking LNEs from broad practical applications.

Recent investigations have demonstrated great potential to alleviate these challenges by increasing the inorganic content in the SEI^11–13^. A case in point is the SEI enriched with LiF^14,15^, as LiF has excellent chemical and electrochemical stability, along with high mechanical strength and high interfacial energy^16^; however, the intrinsically low ionic conductivity of LiF (10^−14^–10^−13^ S cm^−1^ at room temperature) increases the cycle overpotentials and kinetically limits the uniform Li^+^ transport across the SEI at high rates, leading to a rapid capacity fading^17,18^. In this case, some other inorganic compounds, such as Li_3_N^19^, Li_2_S^20^, and Li_2_O^21^, with the Li^+^ conductivity on the scale of 10^−3^, 10^−5^ and 10^−9^ S cm^−1^ at room temperature, respectively, have been incorporated into the SEI to enhance interfacial kinetics for high-rate LNEs, yet these components have been rarely explored as the dominant SEI constituent^22,23^.

Rational electrolyte design via the additive incorporation has emerged as a promising strategy to modulate SEI properties, where the control over the electrolyte solvation structures and reduction pathways could allow precise tailoring of the SEI composition, ionic conductivity, and mechanical resilience^24^. LiNO_3_, a low-cost additive, has demonstrated exceptional efficacy in constructing a SEI enriched with Li_3_N/Li_2_O^11,25^; however, its application in conventional carbonate electrolytes has been severely constrained by its inherent low solubility ( < 0.01 mg mL^−1^ at room temperature)^12,26^. To overcome this limitation, extensive efforts have been devoted to developing LiNO_3_ solubilizers^27^; sulfur-containing compounds (SCCs), particularly those featured with the S = O double bond (*e.g*., sulfonates, sulfates, sulfites and sulfones), have emerged as very promising candidates due to their strong interactions with Li^+^^11,28^, and their inherent potential to engineer the S-incorporated interfacial architecture. Among them, sulfones can be used to construct sulfur-containing inorganic-rich interphase (Li_2_S, Li_2_SO_3_ and Li_2_SO_4_, etc.), enabling improved interfacial stability, charge-transfer kinetics, and mechanical robustness, which are essential for stable LMB operations at high rates^29,30^. Nevertheless, the broader influence of sulfur-containing LiNO_3_-solubilizers on electrolyte solvation structures and interfacial chemistry remains largely underexplored, and the potential of the resultant LMBs requires demonstrations for practical applications.

**Fig. 1:**
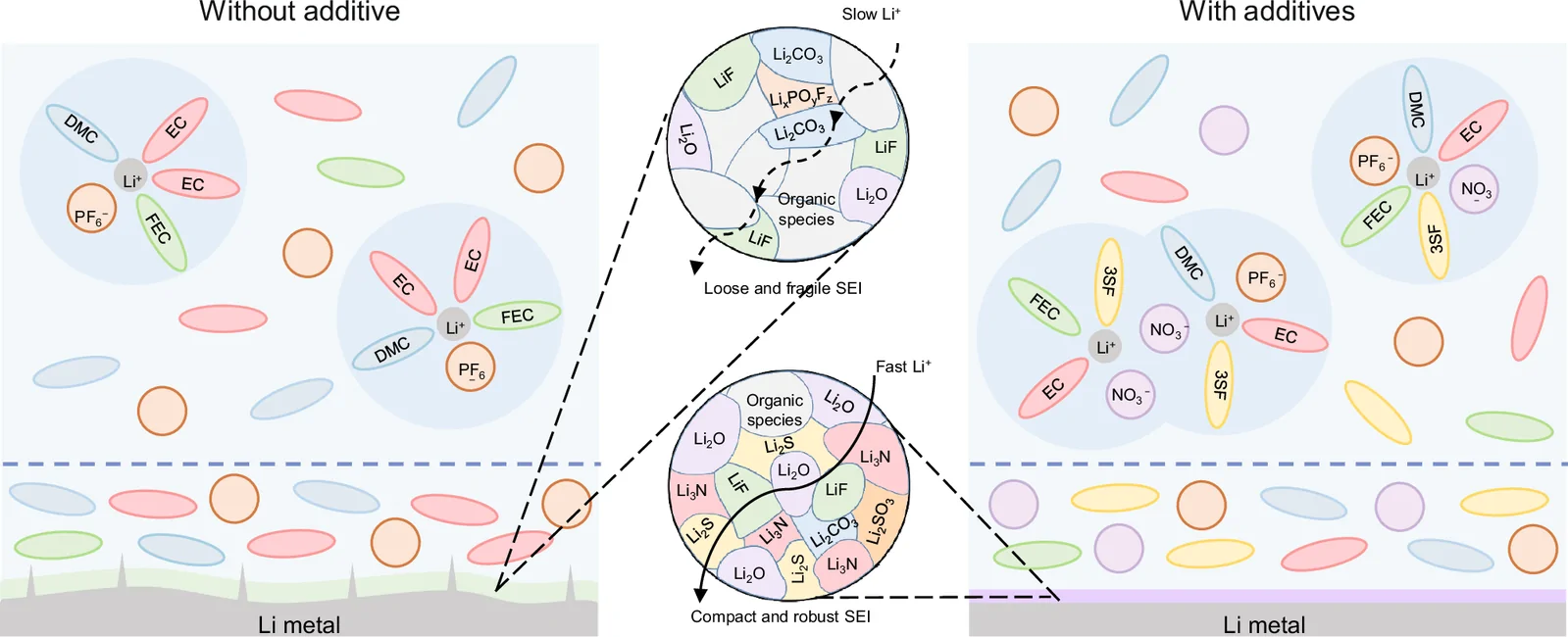
Schematic illustration of SEIs formed in different electrolytes. **a** Illustration of the SEI formed in a conventional carbonate-based electrolyte without the proposed additives. **b** Illustration of the SEI formed in the proposed electrolyte (with additives), which is more favorable for enhancing the Li^+^ transfer kinetics and suppressing the lithium dendrite growth during the cycling process.

Herein, we propose a rapid screening criterion centered on a strong binding energy of SCC-Li^+^-NO_3_^−^ complexes to select SCCs with strong LiNO_3_‑solubilizing capability, and the advantage of sulfone is confirmed through systematic investigations. Among all SCCs tested, 3-sulfolene (3SF) exhibits great potential when it is introduced with LiNO_3_ as dual additives into a commercial carbonate electrolyte simultaneously to enable long-lasting high-rate LNEs. We found that, 3SF works not only as a LiNO_3_-solubilizer, it also enters into the Li^+^-solvation sheath together with NO_3_^−^, to facilitate rapid Li^+^ desolvation kinetics with weakened Li^+^-carbonate interactions, leading to the formation of a SEI layer rich in Li_3_N/Li_2_S/Li_2_O inorganics, which have significantly improved the Li^+^ transfer kinetics and enabled the dendrite-free Li deposition even at high current densities (Fig. 1). With the formulated carbonate electrolyte, Li | |Li, Li | |Cu, Li | |LiFePO_4_ (LFP) and Li | |LiNi_0.8_Co_0.1_Mn_0.1_O_2_ (NCM811) cells have all exhibited stable electrochemical performance. Specifically, Li | |Li symmetric cells show stable Li plating/stripping cycling at 3.0 mA cm^−2^/3.0 mAh cm^−2^ and 5.0 mA cm^−2^/1.0 mAh cm^−2^ for 400 h and 270 h, respectively; a Li | |Cu half cell delivers a high Coulombic efficiency (CE) of 98.6% at 2.0 mA cm^−2^ and 2.0 mAh cm^−2^. Meanwhile, a Li | |LFP full cell delivers a capacity retention of 81.7% after 6000 cycles at 80 C; a Li | |NCM811 full cell displays cycle stability with a capacity retention of 81.3% after 500 cycles at 10 C. Based on the proposed electrolyte, two high-specific-energy ( > 500 Wh kg^−1^) pouch cells retained 92.1% and 91.8% of their capacities after 143 and 149 cycles, respectively. Our work demonstrates the great potential of the the facile dual-additive (SCC + LiNO_3_) strategy, and it highlights the previously underappreciated dual role of SCCs to enhance the solubility of LiNO_3_, while regulating the electrolyte chemistry together with LiNO_3_. The synergistic effect of the dual additives leads to the formation of an inorganic-rich and highly conductive SEI to enhance the high-rate capability of LNEs towards practical applications.

## Results

### Electrolyte design with dual additives

The baseline electrolyte in this work consists of 1.0 M LiPF_6_ in a solvent mixture of ethylene carbonate (EC) and dimethyl carbonate (DMC) with a volume ratio of 1:1, supplemented with 5 wt.% fluoroethylene carbonate (FEC). It is designated as EDF electrolyte, which is a commercial carbonate electrolyte widely used for investigations of LMBs^31,32^. It should be noted that, in this study, only EC and DMC are considered as solvent components; FEC is a basic additive in the baseline and modified electrolytes. The molecular structures of the candidate SCCs are presented in Supplementary Fig. 1. To verify whether these SCCs could work as LiNO_3_-solubilizers in carbonate electrolytes, 0.1 mmol of LiNO_3_ was first added into 1.0 mL of EDF electrolyte. The solution became cloudy with visible undissolved white particles even after stirring for 12 h (Supplementary Fig. 2a), indicating that this concentration has exceeded the solubility limit of LiNO_3_ in carbonate electrolytes, which is consistent with prior studies^33,34^. 0.5 mmol of each SCC was then introduced into the pre-mixed solution (EDF + LiNO_3_). After 12 h of stirring, the majority of resultant solutions became clear (Supplementary Fig. 2b–q). To identify whether the residual turbidity originates from the limited intrinsic solubility of the SCCs themselves or from their insufficient LiNO_3_-solubilizing capability, the same amount of each undissolved SCC was introduced into 1 mL EDF electrolyte and stirred. The results show that, except for methylene methanedisulfonate (MMDS) which still leads to cloudy solution (Supplementary Fig. 3), all other SCCs tested dissolve readily. MMDS was then excluded from subsequent investigations, as it fails to solubilize LiNO_3_ due to its intrinsic solubility limitation. In this case, the turbidity in some dual-additive systems (as compared in Supplementary Fig. 2) indicates that the corresponding SCCs could not effectively promote LiNO_3_ dissolution in EDF electrolyte.

To further elucidate the origin of these differences, the binding energies of complexes formed between Li^+^, NO_3_^−^ and each SCC were calculated. As revealed in Supplementary Table 1 and Supplementary Data 1, systems exhibiting poor LiNO_3_-solubilizing performance generally display less negative binding energies of the SCC-Li^+^-NO_3_^−^ complexes. In contrast, systems that yield clear solutions are associated with more negative (stronger) binding energies. In this case, the binding energy of the SCC-Li^+^-NO_3_^−^ complexes can serve as a descriptor for predicting the overall LiNO_3_-solubilization capability. In addition, electrostatic potential (ESP) maps of SCC molecules (Supplementary Fig. 4) and the SCC-Li^+^-NO_3_^−^ complexes (Supplementary Fig. 5) further reveal that, negative ESP regions associated with S = O groups preferentially coordinate with Li^+^, whereas positive ESP regions around C–H bonds tend to interact with NO_3_^−^. The modified electrolytes incorporating only SCCs are hereafter referred to as EDF‑SCC, whereas those incorporating both a SCC and LiNO_3_ are denoted as EDF‑SCC/LNO.

**Fig. 2:**
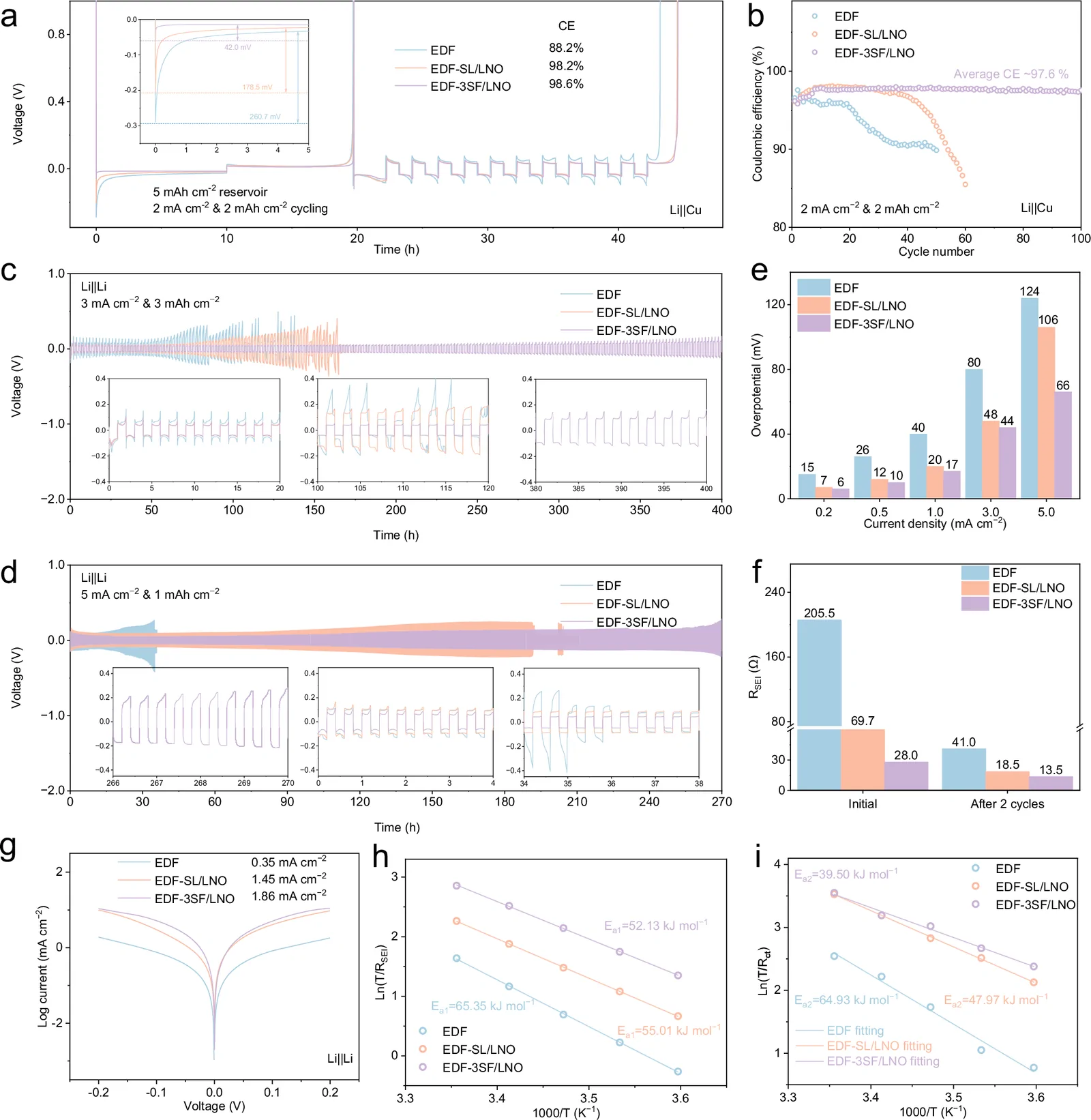
Basic electrochemical performance of Li | |Cu half cells and Li | |Li symmetric cells. CEs measured by the **a** Aurbach method and **b** traditional method in Li | |Cu half cells using different electrolytes, and the inset in **a** compares the respective initial Li nucleation potentials. Voltage-time profiles of Li | |Li symmetric cells at **c** 3.0 mA cm^−2^/3.0 mAh cm^−2^ and **d** 5.0 mA cm^−2^/1.0 mAh cm^−2^, and insets are typical enlarged views of the voltage profile during the Li plating/stripping process. **e** Overpotentials of Li | |Li symmetric cells using different electrolytes at current densities (0.2, 0.5, 1.0, 3.0 and 5.0 mA cm^−2^) for 1.0 mAh cm^−2^. **f** *R*_SEI_ values of Li | |Li symmetric cells with different electrolytes before and after two formation Li plating/stripping steps at 0.2 mA cm^−2^ and 1.0 mAh cm^−2^. **g** Tafel plots of Li | |Li symmetric cells with different electrolytes. Measurement of the activation energies **h** *E*_a1_ and **i** *E*_a2_ in different electrolytes.

Among the systems that solubilize LiNO_3_, the reversibility of Li plating/stripping in EDF and the modified electrolytes was evaluated in Li | |Cu half cells based on the Aurbach method^35,36^. As shown in Fig. 2a and Supplementary Fig. 6, EDF electrolyte displays a Coulombic efficiency (CE) of only 88.2% and a Li nucleation overpotential of 260.7 mV, indicating a poor plating/stripping reversibility and a high interfacial impedance. In contrast, the modified electrolytes demonstrate markedly enhanced performance.

Li | |Cu half cells employing EDF-3SF/LNO or EDF-SL/LNO electrolytes, in which the SCC (3SF, or SL) contains exclusively S = O double bonds between S and O atoms, achieved CEs of 98.2% and 98.6%, respectively, which are higher than those based on additives bearing S–O single bonds (*e.g*., sulfonates, sulfates, and sulfites). Moreover, the nucleation overpotential for EDF‑3SF/LNO is as low as 42.0 mV, and 178.5 mV for EDF‑SL/LNO, both are significantly lower than that in EDF baseline electrolyte. Under LiNO_3_‑free conditions, electrolytes added with sulfones also lead to higher CEs among the evaluated SCCs (Supplementary Fig. 7). These results can be rationalized by previous studies demonstrating that sulfones preferentially undergo electrochemical reduction to form sulfur-containing inorganic species^29,30^, rather than the organic sulfur derivatives typically generated from additives bearing S–O single bonds^37,38^. In general, these inorganics exhibit higher Li^+^ ionic conductivity and mechanical robustness, which are beneficial for suppressing electrolyte depletion and inhibiting dendrite growth.

The interfacial chemistry depends largely on the solvation structure of the electrolyte^39,40^. To further elucidate the effect of the additives on the solvation structure, molecular dynamics (MD) simulations were conducted. Equilibrated snapshots and radial distribution functions (RDFs) (Supplementary Figs. 8 − 10 and Supplementary Data 2) show that additive introduction induces a pronounced reconstruction of the local solvation environment, and NO_3_^−^ can bridge two Li^+^ ions to form aggregate clusters (AGGs). Raman spectrum further corroborates that the NO_3_^−^ coordination leads to a CIP/AGG‑dominated solvation structure in the EDF‑3SF/LNO electrolyte (Supplementary Fig. 11). Coordination numbers obtained via integrating RDFs (Supplementary Figs. 12 and 13) show that, all dual-additive systems, particularly those contains 3SF or SL, weaken the Li^+^-carbonate coordination while increasing the NO_3_^−^ and SCC coordination, which in turn suppresses the formation of organic species in the interphase, enhances Li^+^ transport across the SEI, and promotes Li^+^ desolvation at the electrode interface^41^.

Supplementary Fig. 14 shows the Raman spectra acquired from EDF and EDF-3SF/LNO electrolytes. The intensity of the characteristic peaks corresponding to the coordination of carbonate molecules with Li^+^ significantly decreases upon the addition of the dual additives, which is consistent with the above MD simulations. Nuclear magnetic resonance (NMR) analysis in Supplementary Fig. 15 further indicates that, in EDF‑3SF/LNO electrolyte, the ^19^F NMR spectra reveal a downfield shift of the characteristic peak for PF_6_^−^ upon the addition of LiNO_3_ and 3SF, indicating a weakened interaction between PF_6_^−^ and Li^+^. This phenomenon can be attributed to the partial displacement of PF_6_^−^ by 3SF and NO_3_^−^ in the Li^+^-solvation sheath^42,43^. Additionally, as illustrated in Supplementary Fig. 16, the Li characteristic peak in the ^7^Li NMR spectra undergoes a downfield shift after the introduction of the dual additives, suggesting a decrease in the electron density around Li^+^. These analyses provide strong evidence that LiNO_3_ and 3SF additives play a significant role to tune the Li^+^-solvation sheath in the electrolyte, and they are preferentially reduced on LNEs to form the SEI enriched with inorganic species (Li_3_N, Li_2_S and Li_2_O, etc.), which are far more conductive to Li^+^ and more mechanically robust than that in conventional carbonate electrolytes, endowing the LNE with significantly enhanced high-rate stability over long durations^11–13^.

Density functional theory (DFT) calculations reveal that the presence of the C = C double bond lowers the lowest unoccupied molecular orbital (LUMO) energy of 3SF by 1.05 eV compared to SL (Supplementary Fig. 17 and Supplementary Data 3), suggesting that 3SF is thermodynamically more likely to be reduced than SL; in the lithium-bound state, Li^+^−3SF also exhibits a lower LUMO energy than that of Li^+^-SL (Supplementary Fig. 18), indicating faster reduction kinetics that enable the preferential reduction of 3SF molecules, which was further echoed by a series of experiments. Compared with those tested with EDF-SL electrolyte, EDF-3SF electrolyte leads to a smaller reduction current in the cyclic voltammetry (CV) of Li | |Cu cells (Supplementary Fig. 19), a more rapid current decay and lower leakage current during the constant voltage holding test at 0 V (Supplementary Fig. 20), and the enhanced interfacial stability with the lithium negative electrode during the galvanostatic cycling between 0-1 V (Supplementary Fig. 21). The more effective passivation of a LNE with 3SF is further supported by the scanning electron microscope (SEM) observations of morphologies of the deposited lithium. As compared in Supplementary Fig. 22, EDF-3SF electrolyte yields a denser and more uniform lithium deposit than that formed in EDF-SL electrolyte. These results demonstrate that, the 3SF additive promotes the formation of a more compact and stable SEI, in contrast to the SEI formed with the SL additive. While physically, the three electrolytes exhibit negligible differences in density, viscosity, ionic conductivity, and separator wettability (Supplementary Fig. 23), implying that the introduction of the dual additives SL/LiNO_3_ or 3SF/LiNO_3_ has not changed the basic physical properties of the electrolyte. To explore the mechanism behind the improved interfacial kinetics, we measured the Li^+^ transference number (*t*_+_). As shown in Supplementary Fig. 24, EDF-3SF/LNO electrolyte achieves a significantly higher *t*_+_ value (0.63) than that of EDF electrolyte (0.25), confirming more efficient and selective Li^+^ transport across the interphase, suggesting the accelerated Li^+^ transport and improved stability with the additives.

### Electrochemical compatibility with lithium negative electrodes

Excellent electrochemical compatibility with the LNE is critical for advanced LMB electrolytes. To elucidate the interfacial kinetic modulation mechanisms, systematic investigations employing dual additives in carbonate electrolytes were conducted.

Figure 2b compares the CE of EDF, EDF-SL/LNO and EDF-3SF/LNO electrolytes based on the long-term galvanostatic cycling method. Under the demanding conditions of 2.0 mA cm^−2^ and 2.0 mAh cm^−2^, the Li | |Cu half cell with EDF-3SF/LNO electrolyte exhibits cycle stability, with an average CE of 97.6% over the first 100 cycles, higher than those of EDF (below 90% after 50 cycles) and EDF-SL/LNO (95.7% after 60 cycles) electrolytes. As compared in Supplementary Fig. 25, EDF-SL/LNO (38.9 mV, 37.1 mV, 40.4 mV and 50.9 mV) and EDF-3SF/LNO (41.4 mV, 39.7 mV, 39.9 mV and 41.6 mV) electrolytes deliver significantly lower polarization than EDF electrolyte (44.5 mV, 54.9 mV, 74.7 mV and 91.9 mV) at the 5^th^, 20^th^, 35^th^ and 50^th^ cycles, respectively. Even after 100 cycles, the Li | |Cu half cell using EDF-3SF/LNO electrolyte maintains an overpotential of 57.7 mV. These results demonstrate that, the incorporation of the dual additives effectively reduces the energy barrier for Li nucleation and markedly improves the interfacial compatibility of the carbonate electrolyte with the LNE. Supplementary Fig. 26 presents the CV curve of Li | |Cu half cells using different electrolytes scanned between −0.4 V and 0.8 V at 5 mV s^−1^. The addition of 3SF and LiNO_3_ additives reduces the plating overpotential and significantly increases the capacity (as suggested by the far larger peak area), which indicates enhanced reaction kinetics for the Li plating/stripping process.

Furthermore, the influence of additives on the electrochemical performance of the electrolyte was systematically evaluated using Li | |Li symmetric cells. As depicted in Fig. 2c, when subjected to Li plating/stripping cycling at 3.0 mA cm^−2^ and 3.0 mAh cm^−2^, the Li | |Li symmetric cell employing EDF electrolyte experiences a short circuit after approximately 100 h. This can be attributed to the low ionic conductivity of the SEI formed in conventional carbonate electrolytes, which impedes Li^+^ transport across the interphase, leading to the rapid Li dendrite growth and gradual accumulation of “dead Li”, ultimately causing the cell failure^12,44^. In comparison, the cell with EDF-SL/LNO electrolyte shows improved stability, undergoing short circuit after about 150 h, and the cell using EDF-3SF/LNO electrolyte demonstrates stable Li plating/stripping for over 400 h with a much lower overpotential. Even when at a high current density of 5.0 mA cm^−2^, the cells with EDF-3SF/LNO and EDF-SL/LNO electrolyte maintain stable Li plating/stripping for 270 h (Fig. 2d) and about 200 h, respectively, while the cell with EDF electrolyte experiences a short circuit in about 35 h.

When tested with a fixed areal capacity of 1.0 mAh cm^−2^ at current densities of 0.2, 0.5, 1.0, 3.0 and 5.0 mA cm^−2^ (Fig. 2e and Supplementary Fig. 27), the Li | |Li symmetric cell using EDF-3SF/LNO electrolyte exhibits overpotentials of 6 mV, 10 mV, 17 mV, 44 mV and 66 mV, respectively, which are consistently lower than those using EDF-SL/LNO electrolyte (7 mV, 12 mV, 20 mV, 48 mV, and 106 mV, respectively) and EDF electrolyte (15 mV, 26 mV, 40 mV, 80 mV, and 124 mV, respectively). It is evident that the addition of 3SF and LiNO_3_ additives significantly improves interfacial compatibility between the carbonate electrolyte and the LNE along with a reduced interfacial impedance, promoting uniform Li deposition and extending the cycling lifespan of the LNEs, even under high-rate conditions.

### Interfacial impedance and Li deposition behavior

The interfacial impedance in different electrolytes was investigated using electrochemical impedance spectroscopy (EIS). In the EIS plot, the SEI resistance (*R*_SEI_), corresponding to the width of the high-frequency semicircle, directly reflects the difficulty of Li^+^ transport through the SEI^45,46^. Figure 2f illustrates the changes in *R*_SEI_ measured before and after two formation cycles in different electrolytes, with corresponding EIS results shown in Supplementary Fig. 28 and Supplementary Tables 2–4. The *R*_SEI_ values of EDF-3SF/LNO electrolyte (from 28.0 Ω to 13.5 Ω) remain lower than that of EDF-SL/LNO electrolyte (from 69.7 Ω to 18.5 Ω), and both are much lower than that of EDF electrolyte (from 205.5 Ω to 41.0 Ω). Extended screening of other SCCs with LiNO_3_ further indicated that, although these additive systems showed a clear reduction in *R*_SEI_ compared to EDF, their performance remained inferior to that of the 3SF‑based electrolyte (Supplementary Figs. 29 and 30, and Supplementary Tables 5–10). The same trend is observed in cells after 40 Li plating/stripping cycles at 3.0 mA cm^−2^ and 3.0 mAh cm^−2^ (Supplementary Fig. 31), indicating that the introduction of the 3SF/LiNO_3_ additives have reduced interfacial impedance and enhanced the stability of the SEI. The electrochemical reaction kinetics of different electrolytes were compared by assembling Li | |Li symmetric cells and measuring the Tafel plots. As illustrated in Fig. 2g, the exchange current density (*i*_0_) derived from the fitted Tafel plot in EDF-3SF/LNO electrolyte (1.86 mA cm^−2^) is higher than that in EDF-SL/LNO electrolyte (1.45 mA cm^−2^), and both are higher than that in EDF electrolyte (0.35 mA cm^−2^). Extended screening of other SCCs with LiNO_3_ further demonstrated that, while these additives exhibited faster kinetics relative to EDF, their performance remained inferior to the 3SF-based electrolyte (Supplementary Fig. 32). The enhanced kinetics of the 3SF system promote more uniform Li^+^ flux^47^, which is consistent with the above tests.

The kinetic behavior of Li^+^ transport at the interphase was analyzed by acquiring the temperature-dependent EIS data with Li | |Li symmetric cells. Specifically, EIS data were measured within a temperature range of 273 K to 293 K (Supplementary Fig. 33), and the activation energies for Li^+^ transport through the SEI (*E*_a1_) and Li^+^ desolvation (*E*_a2_) were calculated using the Arrhenius equation^48^. As revealed in Fig. 2h, the addition of LiNO_3_ and SL additives to EDF electrolyte reduces *E*_a1_ from 65.35 kJ mol^−1^ to 55.01 kJ mol^−1^, and the *E*_a2_ value is decreased from 64.93 kJ mol^−1^ to 47.97 kJ mol^−1^ (Fig. 2i). In comparison, the addition of LiNO_3_ and 3SF additives further reduced the *E*_a1_ value to 52.13 kJ mol^−1^ (Fig. 2h) and the *E*_a2_ value to 39.50 kJ mol^−1^ (Fig. 2i). These findings demonstrate that, the introduction of the additives significantly enhances the interfacial kinetics, with the 3SF/LiNO_3_ system providing the most improvement, which is crucial for achieving stable operation of high-rate LNEs. Based on the above analysis, EDF‑3SF/LNO electrolyte demonstrates the best overall performance among the SCCs tested and it was therefore selected for further investigations.

**Fig. 3:**
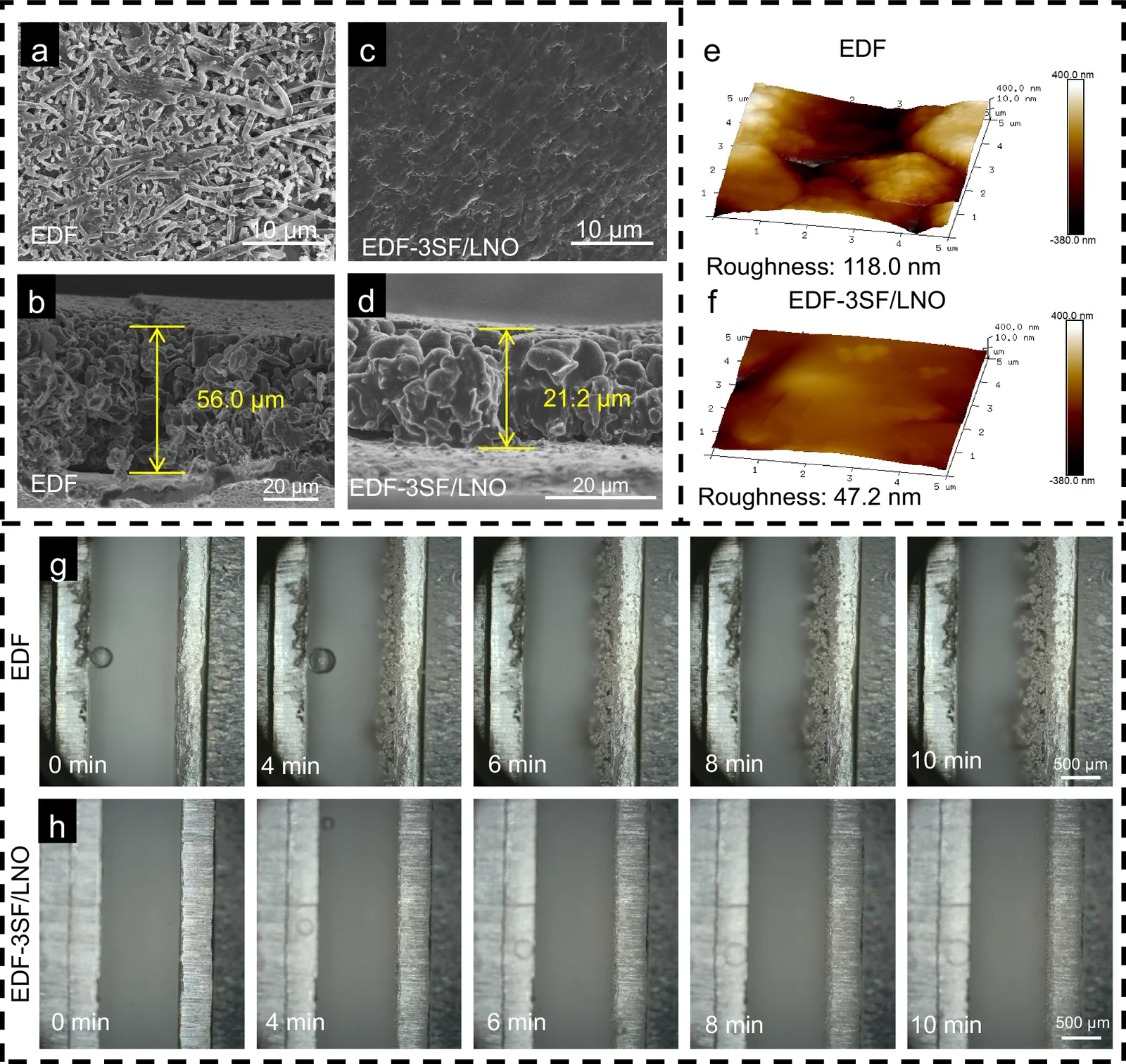
Morphologies of the Li deposited on Cu substrate in the baseline and modified electrolytes. SEM images: **a** top view and **b** cross-sectional view of the Li deposited in EDF baseline electrolyte, **c** top view and **d** cross-sectional view of the Li deposited in EDF-3SF/LNO modified electrolyte. 3D AFM images of Li deposited on Cu substrates in **e** EDF and **f** EDF-3SF/LNO electrolytes (deposition condition: 0.5 mA cm^−2^ and 3.0 mAh cm^−2^ at 25 °C). Time sequence images acquired during in situ optical microscopy observation of the Li deposition process in **g** EDF and **h** EDF-3SF/LNO electrolytes at 3.0 mA cm^−2^ and 25 ± 2 °C.

The microstructure of the electrodeposited Li plays a key role to the cycle stability of LNEs^49^. Optical microscopy observations were performed to elucidate the overall morphology of the Li deposited on the Cu substrate in the two electrolytes. As shown in Supplementary Fig. 34, after the Li deposition at 0.5 mA cm^−2^ for 3.0 mAh cm^−2^, the Cu substrate in EDF electrolyte is very bumpy with partial Li exfoliation at the surface, while the Li deposited in EDF-3SF/LNO electrolyte maintains a silvery appearance with a homogeneous and compact structure on the Cu substrate. SEM was employed to characterize the Li microstructures deposited in different electrolytes. For the Li deposited in EDF electrolyte, the top view shown in Fig. 3a reveals needle-like dendritic structures with a high tortuosity, while the cross-sectional view in Fig. 3b shows that these structures are loosely packed with a total thickness of 56.0 µm. In sharp contrast, the Li deposited in EDF-3SF/LNO electrolyte are dendrite-free and highly compact (Fig. 3c), with a total thickness of only 21.2 µm in the cross-sectional view (Fig. 3d). The more uniform Li deposition in EDF-3SF/LNO electrolyte than in EDF electrolyte is further confirmed by the atomic force microscope (AFM) measurements, as demonstrated by a large reduction in average surface roughness (from 118.0 nm in Figs. 3e 47.2 nm in Fig. 3f).

To compare the effect of 3SF/LiNO_3_ additives on the Li deposition behavior, in situ optical microscopy observation was employed to monitor the Li deposition process on LNEs in EDF and EDF-3SF/LNO electrolytes at a high current density of 3.0 mA cm^−2^. As shown in Fig. 3g (and Supplementary Movie 1), in EDF electrolyte, the Li dendrites grow steadily with the plating duration (deposition capacity). On the contrary, the Li deposition in EDF-3SF/LNO electrolyte exhibits a uniform and compact morphology, with no evident dendrite formation or growth observed (Fig. 3h and Supplementary Movie 2), which is consistent with the lower overpotential after introducing the additives (Supplementary Fig. 35). These observations further confirm that, the SEI formed in the modified electrolyte is uniform and compact, together with the reduced interfacial impedance analyzed above, they promote the uniform and rapid Li deposition in carbonate electrolytes for enhanced cycle stability even under high rates.

### Compositions of the solid electrolyte interphase

**Fig. 4:**
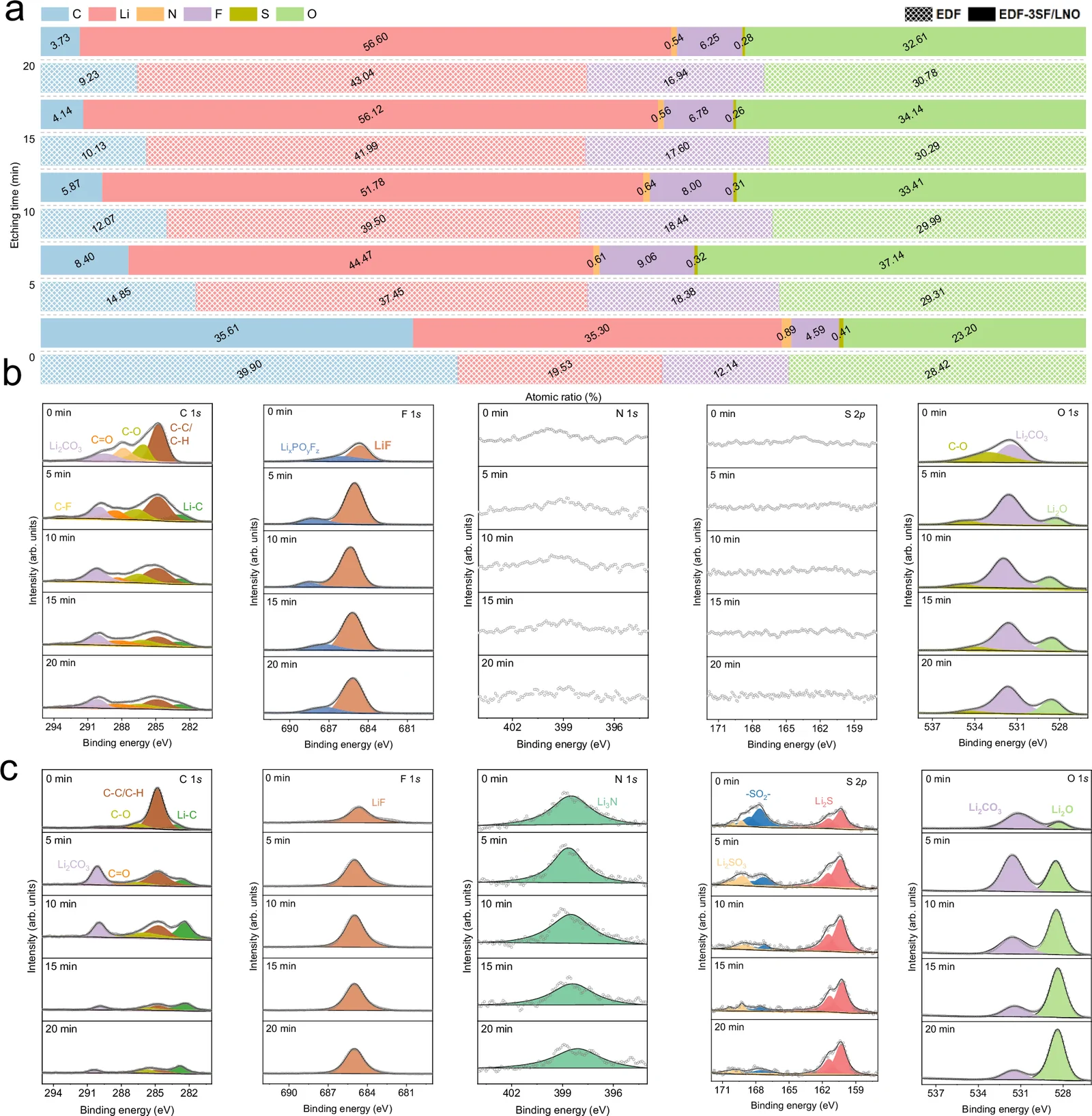
XPS characterization of the SEI compositions. **a** Atomic ratios of different elements in the SEI of LNEs after Li plating/stripping in different electrolytes. C 1 *s*, F 1 *s*, N 1 s, S 2*p* and O 1 *s* XPS depth profiling spectra acquired from SEIs on LNEs after Li plating/stripping in **b** EDF and **c** EDF-3SF/LNO electrolytes (cycle condition: 3.0 mA cm^−2^ and 3.0 mAh cm^−2^ for 10 Li plating/stripping cycles at 25 °C).

The differences in the interfacial kinetics and Li^+^ deposition behavior are highly dependent on the SEI compositions^50,51^. In this case, the X-ray photoelectron spectroscopy (XPS) depth profiling, by controlling the Ar^+^ sputtering duration before the data acquisition, was performed on the SEI formed in the baseline and modified electrolytes. Typically, the ratio of C and Li elements in the SEI can reflect the overall contents of its organic and inorganic components, respectively^52^. As shown in Fig. 4a, the SEI formed in EDF electrolyte exhibits a significantly higher portion of C along with a much lower Li content throughout the entire sputtering process, compared to those formed in EDF-3SF/LNO electrolyte, suggesting that the SEI formed in EDF-3SF/LNO electrolyte carries a much higher ratio of inorganic components.

High-resolution XPS C 1 *s* spectra in Fig. 4b reveals the detection of C–C/C–H (284.8 eV), C–O (286.5 eV), C = O (288.5 eV), and Li_2_CO_3_ (289.8 eV), which is consistent with those of the SEI formed in conventional carbonate electrolytes^11^. In the F 1 *s* spectra, a peak at 685.6 eV is attributed to LiF, generated by the decomposition of fluorides (LiPF_6_ and FEC); another peak at 686.8 eV is attributed to Li_x_PO_y_F_z_. Generally, Li_x_PO_y_F_z_ is a byproduct from the LiPF_6_ hydrolysis, and its accumulation at the interface significantly increases the interfacial impedance, reducing the high-rate performance and long-term cycle stability of LNEs^42,43^.

In comparison, the SEI formed in EDF-3SF/LNO electrolyte exhibits very different characteristics, highlighting the key role of the dual additives to tune the interfacial chemistry (Fig. 4c). Specifically, additional peaks from Li_3_N and Li_2_S are detected in the N 1 *s* and S 2*p* spectra, respectively, and a stronger Li_2_O peak is observed in the O 1 *s* spectra. The intensity of these inorganic peaks remain relatively stable even after increasing the Ar^+^ sputtering time, indicating that they are distributed throughout the SEI. The formation of these inorganic species, including Li_3_N, Li_2_S, and Li_2_O, within the SEI is primarily attributed to the electrochemical decomposition of the LiNO_3_ and 3SF additives, as they are absent or only scarcely detected in the SEI formed in EDF electrolyte alone. XPS analysis of the SEI formed in EDF‑SL/LNO (Supplementary Fig. 36) likewise detects Li_3_N, Li_2_S, and Li_2_O, similar to EDF-3SF/LNO electrolyte, corroborating the feasibility of our dual‑additive strategy for constructing inorganic‑rich SEIs.

As discussed above, Li_3_N, Li_2_S and Li_2_O possess far higher ionic conductivities than LiF, which is beneficial for rapid Li^+^ transfer and suppression of Li dendrite growth, thereby enhancing the long-term cycle stability of high-rate LNEs^17–21^. Additionally, the incorporation of S-/N-containing species into the SEI could significantly accelerate the Li^+^ desolvation kinetics by leveraging their electron-rich characteristics^20,53,54^. In the F 1 *s* spectra, the SEI formed in EDF-3SF/LNO electrolyte shows almost no detectable Li_x_PO_y_F_z_ peak, suggesting that the introduction of additives suppresses the side reactions from the LiPF_6_ hydrolysis. These results indicate that the simultaneous introduction of LiNO_3_ and 3SF additives could effectively improve the interfacial kinetics and thermodynamic stability of LNEs in carbonate electrolytes, which is critical to enhance the high-rate performance of LMBs.

### Full cell performance

**Fig. 5:**
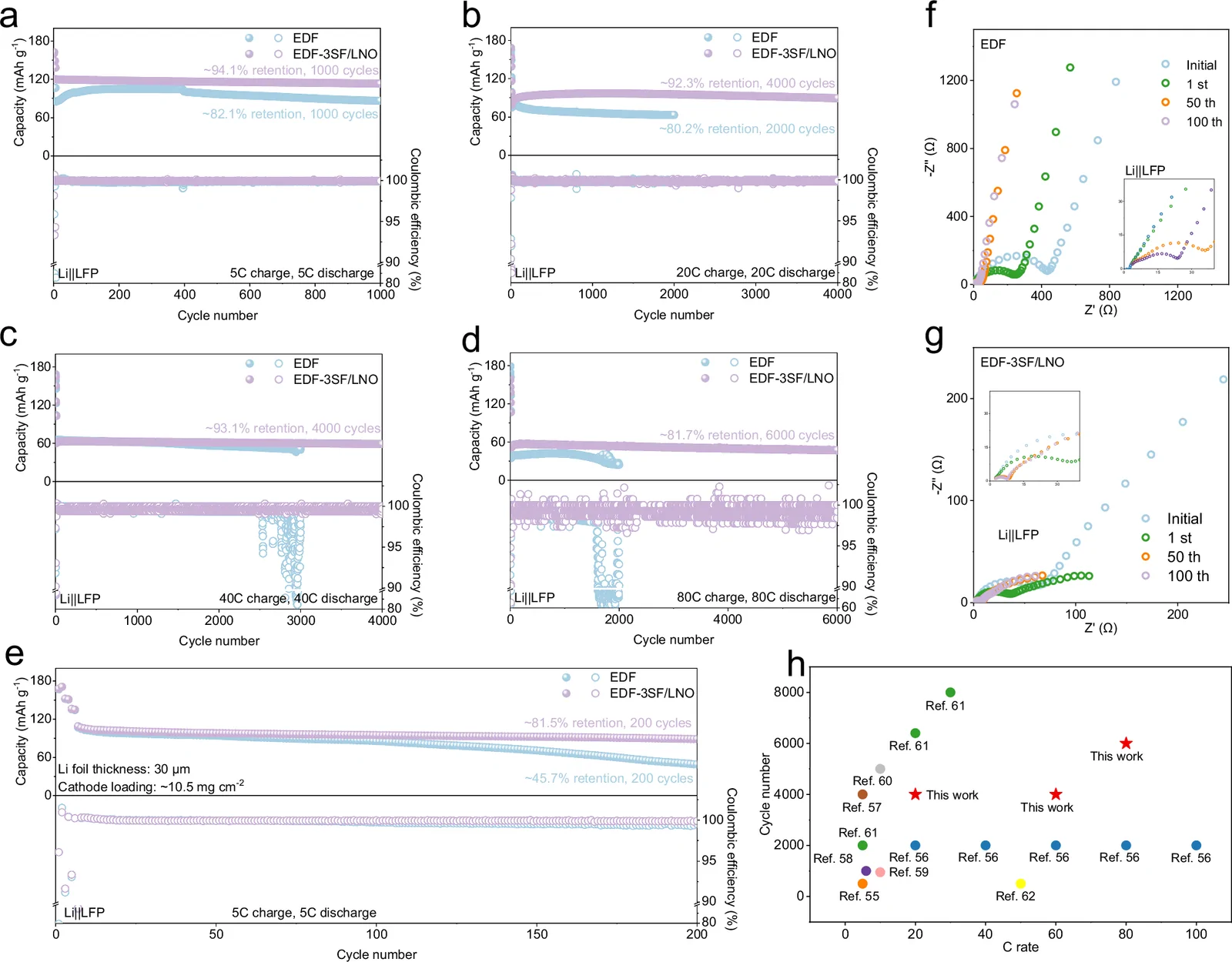
Electrochemical performance of Li | |LFP full cells using the baseline and modified electrolytes. Cycle performance tested at: **a** 5 C, **b** 20 C, **c** 40 C, **d** 80 C, **e** 5 C using a thin (30 µm) Li foil paired with a high-loading (10.5 mg cm^−2^) LFP positive electrode. EIS profiles plots of Li | |LFP full cells using **f** EDF and **g** EDF-3SF/LNO electrolytes after different cycles at 10 C, and the insets are the corresponding enlarged views at the high-frequency region. **h** Overall rate performance and cycle life of Li | |LFP full cells in the literature and this work. For Li | |LFP cells, 1 C = 170 mA g^−1^.

To evaluate the practical applicability of the proposed electrolytes, Li | |LFP and Li | |NCM811 full cells were assembled and tested. The rate performance of Li | |LFP full cells with different electrolytes was compared at rates ranging from 0.5 C to 100 C (1 C = 170 mA g^−1^). As shown in Supplementary Fig. 37, EDF-3SF/LNO electrolyte enables higher capacity and improved stability across all tested rates, compared to EDF electrolyte. For the long-term cycle stability, as depicted in Fig. 5a–d, the cells using EDF-3SF/LNO electrolyte cycled at high rates of 5 C, 20 C, 40 C, and 80 C, exhibit capacity retention rates of 94.1%, 92.3%, 93.1% and 81.7%, after 1000, 4000, 4000 and 6000 cycles, respectively, which are higher than those using EDF electrolyte.

To further validate the advantages of the designed electrolyte, the electrochemical performance was examined under conditions close to practical applications, using a thin Li foil (30 µm) negative electrode paired with a high-loading (10.5 mg cm^−2^) LFP positive electrode at a high rate of 5 C. As shown in Fig. 5e, the cell with EDF-3SF/LNO electrolyte shows a high discharge capacity of 88.75 mAh g^−1^ with a capacity retention of 81.5% after 200 cycles, while that with EDF electrolyte shows a rapid capacity decay and retains only 45.7% of its initial capacity over the same cycling period. Meanwhile, the addition of LiNO_3_ and 3SF additives allows reduction in the cycle overpotential while delivering an increased specific capacity at all rates tested (Supplementary Figs. 38–42). This aligns with the EIS results, which show that the cell employing EDF-3SF/LNO electrolyte exhibits lower interfacial impedance and smaller impedance fluctuations compared to those with EDF electrolyte (Fig. 5f, g). Furthermore, Fig. 5h and Supplementary Table 11 compares the electrochemical performance of high-rate Li | |LFP full cells in this work with that of reported works^55–62^, highlighting the rate capability and cycle stability achieved in this work.

**Fig. 6:**
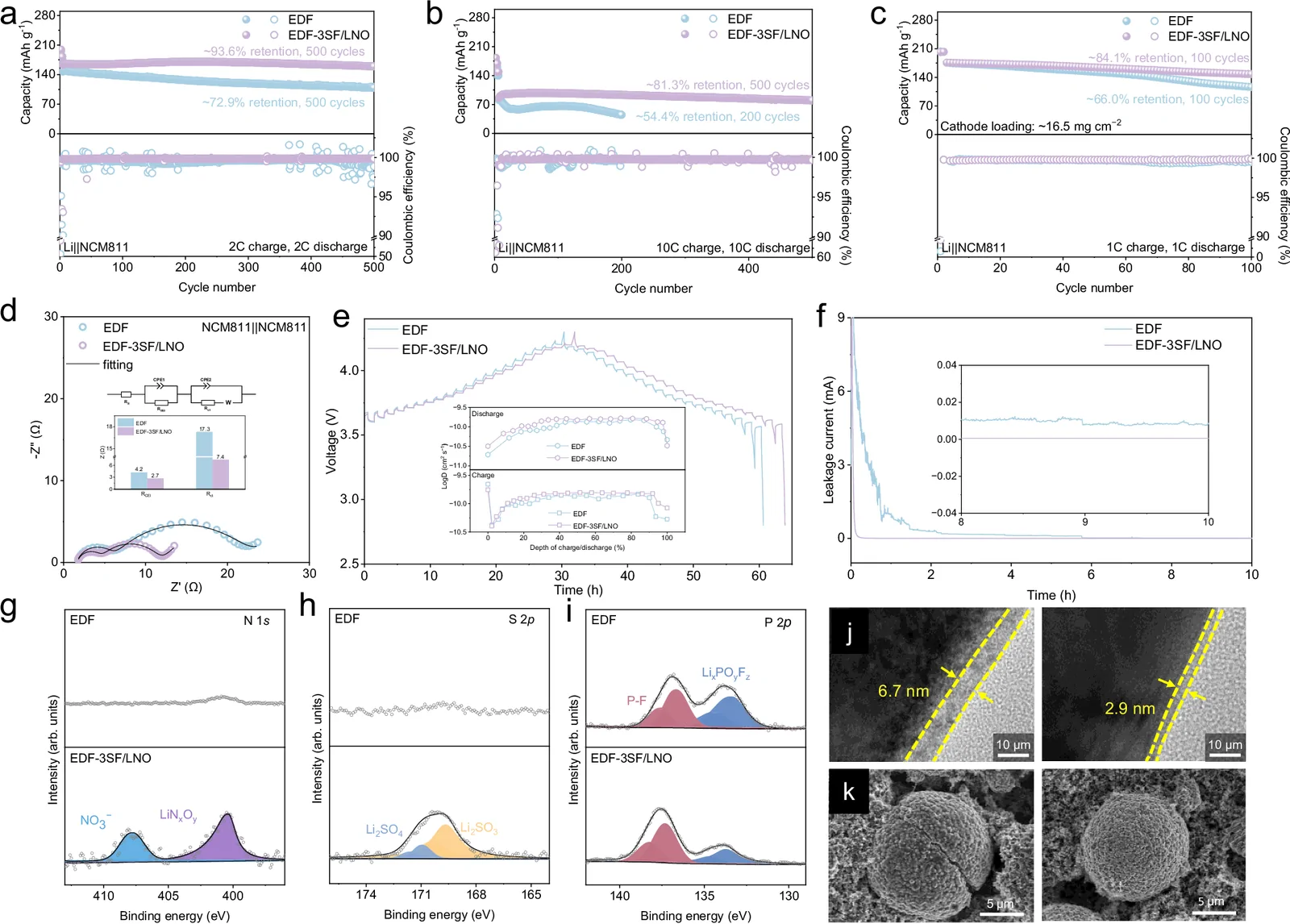
Electrochemical performance of Li | |NCM811 full cells using the baseline and modified electrolytes. Cycle performance tested at: **a** 2 C, **b** 10 C, and **c** 1 C with a high-loading (16.5 mg cm^−2^) positive electrode. **d** EIS plots of NCM811 | | NCM811 symmetric cells with different electrolytes, and the inset shows the fitting result. **e** Charge/discharge voltage profiles of GITT measurements after 5 cycles, and the inset is the corresponding log*D* of NCM811 positive electrodes in different electrolytes; *D* is the diffusion coefficient of Li^+^. **f** Leakage currents of Li | |NCM811 full cells using different electrolytes at a constant applied voltage of 4.3 V, and the inset is a local enlarged view. **g** N 1 *s*, **h** S 2*p* and **i** P 2*p* XPS spectra acquired from the CEIs of NCM811 positive electrodes after 50 cycles at 2 C with different electrolytes. **j** HRTEM and **k** SEM images of the NCM811 positive electrode particles collected from Li | |NCM811 full cells after 300 cycles at 2 C with different electrolytes. For Li | |NCM811 cells, 1 C = 200 mA g^−1^.

To assess the compatibility of the designed electrolyte with high-voltage positive electrodes, full cells were assembled and tested using NCM811 as the positive electrode. The electrochemical oxidation stability of the electrolytes was examined using the linear sweep voltammetry (LSV) with stainless-steel electrodes. As shown in Supplementary Fig. 43, the modified electrolyte demonstrates an oxidation stability potential above 4.6 V, higher than about 4.3 V of the EDF baseline electrolyte. Besides, the CV curve of the Li | |NCM811 cell employing the modified electrolyte exhibits three well-defined redox couples (Supplementary Fig. 44), corresponding to the characteristic phase transitions of the layered NCM811 positive electrode during the charge/discharge process^63,64^. Therefore, the modified electrolyte demonstrates high compatibility with the high-voltage nickel-rich positive electrode. EDF-3SF/LNO electrolyte allows an improved rate capability, especially at high rates (Supplementary Fig. 45). During long-term cycling, the Li | |NCM811 full cells with EDF-3SF/LNO electrolyte demonstrate high cycle stability, with capacity retention ratios of 93.6% and 81.3% after 500 cycles at 2 C (Fig. 6a and Supplementary Fig. 46, 1 C = 200 mA g^−1^) and 10 C (Fig. 6b and Supplementary Fig. 47), respectively, which are higher than those obtained with EDF electrolyte (72.9% and 54.4%, respectively). Furthermore, under even more stringent conditions with an upper cut-off voltage of 4.6 V and at a 5 C rate, the cell retains 81.7% of its initial capacity after 300 cycles (Supplementary Fig. 48). To confirm the reproducibility of these high-performance full cells using the modified electrolyte, for all the full cell data shown in Figs. 5 and 6, each cell has been repeated twice under the same condition, and the results are encouraging and highly reproducible, as shown in Supplementary Fig. 49. Moreover, the effectiveness of the dual-additive strategy is further validated in an ether-based electrolyte (Supplementary Fig. 50), demonstrating its broad applicability across different electrolyte systems.

The surface morphologies of lithium negative electrodes after 200 cycles under various conditions are compared in Supplementary Fig. 51, including including those tested in Li | |LFP cells at 5 C, 20 C, 40 C, and 80 C, as well as in Li | |NCM811 cells at 2 C and 10 C, using both baseline and modified electrolytes. At all rates tested, the Li cycled in EDF electrolyte becomes pulverized into porous architectures with large cracks, indicating the uneven Li plating and stripping during the high-rate cycling process. In contrast, the surface of the Li cycled in EDF-3SF/LNO electrolyte exhibits a much more uniform and compact morphology, characterized by large granular structures, which can be attributed to the formation of a compact and robust inorganics-rich SEI, leading to the significantly enhanced interfacial stability and uniform Li^+^ flux across the interphase.

To test the potential of the electrolyte for practical applications, a high-loading (16.5 mg cm^−2^) NCM811 positive electrode was incorporated into Li-metal full cells. The cell using EDF-3SF/LNO electrolyte operates steadily and demonstrates cycle stability, retaining 97.9% of its initial capacity after 200 cycles at a charging rate of 0.2 C and a discharging rate of 0.5 C (Supplementary Fig. 52); and it has a capacity retention of 84.1% after 100 cycles at 1 C, far higher than 66.0% of the cell using EDF electrolyte tested under the same condition (Fig. 6c and Supplementary Fig. 53). Clearly, significant improvement in interfacial stability and reaction kinetics on the LMBs has been enabled by the additives. Besides, we tested high-loading ( ~ 16.5 mg cm^−2^) NCM811 full‑cells with the electrolyte incorporating LiNO_3_ together with some other SCCs (Supplementary Fig. 54), they have all exhibited significantly improved cycle stability over those based on the baseline electrolyte, suggesting that the dual-additive (SCC-LiNO_3_) strategy could be an universal and effective electrolyte design strategy.

We further conducted a series of testings with pouch cells, using a Li metal negative electrode (50 µm thick) paired with a NCM811 positive electrode. We first cycled three 0.25-Ah pouch cells, they have all demonstrated cycle stability with capacity retention rates exceeding 86.0% after at least 150 cycles (Supplementary Figs. 55 − 57), confirming the process compatibility and reproducibility of this electrolyte. We then prepared two 7.8-Ah pouch cells, both of which have delivered specific energy exceeding 500 Wh kg^−1^, with capacity retentions of 92.1% after 143 cycles and 91.8% after 149 cycles, respectively (Supplementary Figs. 58 and 59). These results are competitive with some recent reports on electrolyte engineering evaluated in pouch‑cell configurations (Supplementary Fig. 60 and Supplementary Table 12). The corresponding key parameters of these two pouch cells with specific energy exceeding 500 Wh kg^−1^ are listed in Supplementary Tables 13 and 14, respectively.

Besides the SEI on the negative electrode with the modified electrolyte discussed above, the formation of a high-quality cathode-electrolyte interphase (CEI) layer on the positive electrode could also contribute to the enhanced rate performance of full cells. To investigate the interfacial charge transfer kinetics across the CEI, two NCM811 positive electrodes, collected from Li | |NCM811 full cells after 50 cycles at 2 C, were reassembled into a NCM811 | | NCM811 symmetric cell. The impedance of positive electrodes retrieved from EDF electrolyte is higher than that from EDF-3SF/LNO electrolyte, indicating the sluggish interfacial lithium-ion transfer on the positive electrode (Fig. 6d and Supplementary Table 15). As depicted in Fig. 6e and Supplementary Fig. 61, galvanostatic intermittent titration (GITT) and its derived Li^+^ diffusion coefficient (*D*) results reveal a lower charge-discharge overpotential and markedly higher *D* for the NCM811 positive electrode cycled in the modified electrolyte, indicating the superior Li^+^ diffusion kinetics and enhanced structural stability^31^. The 4.3 V constant-voltage floating tests reveal a reduced leakage current in a Li | |NCM811 full cell employing EDF-3SF/LNO electrolyte compared to that with EDF electrolyte (Fig. 6f), suggesting a reduced active Li capacity loss and suppressed side reactions in the delithiated NCM811 positive electrode by the addition of dual additives^32^. With higher highest occupied molecular orbital (HOMO) energy levels over the solvents (Supplementary Fig. 17), the two additives are more susceptible to electron loss and oxidation on the positive electrode, thus modulating the CEI chemistry. As evidenced by the N 1 *s* (Fig. 6g) and S 2*p* (Fig. 6h) spectra, the incorporation of additives gives rise to characteristic peaks corresponding to LiN_x_O_y_, Li_2_SO_3_ and Li_2_SO_4_, respectively, which exhibit high ionic diffusion coefficients and thus enable rapid Li^+^ transport within the CEI^12,65,66^. Besides, the weaker Li_x_PO_y_F_z_ signal observed in EDF-3SF/LNO electrolyte compared to that in EDF electrolyte indicates that the side reactions associated with LiPF_6_ hydrolysis have been effectively suppressed by the introduction of the additives (Fig. 6i)^42,43^. High-resolution transmission electron microscopy (HRTEM) images in Fig. 6j show that the NCM811 particles in EDF electrolyte are covered by a thick CEI of ~6.7 nm, whereas the CEI formed in EDF-3SF/LNO electrolyte is only ~2.9 nm thick, implying that the decomposition of additives leads to a thin and robust CEI that protects the electrolyte from further oxidation. SEM images in Fig. 6k further support these findings, revealing pronounced fracturing of NCM811 particles in EDF electrolyte, in contrast to the preserved morphology observed in EDF-3SF/LNO electrolyte. The inductively coupled plasma mass spectrometry (ICP-MS) data in Supplementary Fig. 62 further reveals that, the introduced additives effectively mitigate the transition metal dissolution, thereby enhancing the structural integrity and overall electrochemical stability of the system. What’s more, Supplementary Fig. 63 shows that, the CEI derived from EDF-3SF/LNO electrolyte exhibits a significantly higher Derjaguin-Muller-Toporov (DMT) modulus (3.70 GPa) compared to that from EDF electrolyte (1.05 GPa). These results reveal that the CEI formed in EDF-3SF/LNO electrolyte is thin, uniform, robust, and it is rich in ionically-highly conductive inorganic components, which are critical to enhance the rate performance of LMBs.

Moreover, by leveraging the enhanced interfacial charge-transfer kinetics and thermodynamic resilience brought by the rationally formulated carbonate electrolyte, Li | |NCM811 full cells exhibit operational stability across temperature conditions. At an elevated temperature of 60 °C, the cell utilizing EDF-3SF/LNO electrolyte demonstrates cycle stability, retaining a capacity of 190.9 mAh g^−1^ (94.9%) after 200 cycles at 2 C (Supplementary Fig. 64); while the Li | |NCM811 full cell can be cycled for 300 cycles at −15 °C (Supplementary Fig. 65).

## Discussion

In summary, bassed on the binding energies of SCC-Li^+^-NO_3_^−^ complexes and systematic investigations, we identified 3-sulfolene (3SF) as a very promising LiNO_3_ solubilizer, and a formulated carbonate electrolyte containing 3SF/LiNO_3_ dual additives is designed for long-lasting high-rate LNEs. The strong affinity between 3SF and Li^+^ enables 3SF molecules to promote the dissociation of LiNO_3_ in carbonate electrolytes. Expermental results reveal that, the incorporation of LiNO_3_ and 3SF as synergistic additives significantly alters the original solvation structure of the electrolyte. Specifically, NO_3_^−^ and 3SF displace carbonate molecules in the first Li^+^-solvation sheath, facilitating the in situ formation of a Li_3_N/Li_2_S/Li_2_O-rich SEI along with a significantly reduced interfacial impedance. Therefore, rapid while uniform Li^+^ transport across the interphase is promoted to effectively suppress the Li dendrite formation and growth. As a result, Li | |Li symmetric cells exhibit the stable Li plating/stripping of 400 h at 3.0 mA cm^−2^/3.0 mAh cm^−2^ and 270 h at 5.0 mA cm^−2^/1.0 mAh cm^−2^, and a Li | |Cu half cell delivers a high CE of 98.6% at 2 mA cm^−2^ and 2 mAh cm^−2^. Furthermore, a Li | |LFP full cell shows a capacity retention of 81.7% after 6000 cycles at a high rate of 80 C; a Li | |NCM811 full cell retains 81.3% of its initial capacity after 500 cycles at 10 C. Moreover, two pouch full cells with specific energy exceeding 500 Wh kg^−1^ have maintained 92.1% and 91.8% of their initial capacity after 143 and 149 cycles, respectively. This work opens up new possibilities for the stabilization of high-rate LNEs for broad applications.

## Methods

### Materials Preparation

The baseline electrolyte consists of 1.0 M LiPF_6_ in a 1:1 (v/v) mixture of ethylene carbonate (EC), and dimethyl carbonate (DMC) with 5 wt % fluoroethylene carbonate (FEC) (denoted as EDF electrolyte) was purchased from DoDoChem. LiNO_3_ (99.9%), was ordered from DoDoChem. Various sulfur-containing compounds (respective purities: 1,3-propanediolcyclic sulfate (PCS) 98.0%; 1,3-propane sultone (PS) 99.0%; prop-1-ene-1,3-sultone (PES) 99.93%; 1,4-butane sultone (BS) 97.0%; diisopropyl sulfate (DiS) 97.0%; methylene methanedisulfonate (MMDS) 99.0%; 1,3,2-dioxathiolane-2,2-dioxide (DTD) 99.5%; ethylene sulfite (ES) 99.0%; methyl ethanesulfonate (ME) 97.0%; propylene sulfite (PSi) > 98%; sulfolane (SL) 99.5%; 3-sulfolene (3SF) > 98.0%; difurfurylsulfide (DFS) > 98.0%; diethyl sulfite (DES) 99.0%; 1,3-dithiane (DTH) 98.0%; dimethyl sulfide (DMS) > 99.0%) and *N*-methyl-2-pyrrolidone (NMP, 99.5%) were supplied by Shanghai Titan Technology Co., Ltd. Li foil (99.9%, 450 µm or 30 µm thickness) was purchased from Guangdong Canrd New Energy Technology Co., Ltd; Li foils were used as received without cleaning or polishing. LiFePO_4_ (LFP), LiNi_0.8_Mn_0.1_Co_0.1_O_2_ (NCM811), conductive carbon (Super P), polyvinylidene fluoride (PVDF, mW~1.0 ×10^6^), Cu foil (99.8%, 9 µm thick), and Al foil (99.6%, 16 µm thick) were obtained from Shenzhen Kejing Star Technology Co., Ltd. The modified electrolyte was prepared by adding ~6.9 mg of LiNO_3_ and 59.1 mg of 3SF to 1.0 mL of the baseline electrolyte and stirring overnight at 25 ± 2 °C, denoted as EDF-3SF/LNO electrolyte (containing ~0.1 M LiNO_3_). All electrolytes were prepared in an argon-filled glovebox (MBraun UNILab Pro SP, H_2_O < 0.1 ppm, O_2_ < 0.1 ppm). The LFP and NCM811 (3–4 mg cm^−2^) positive electrodes were fabricated by mixing the active material, Super P carbon, and PVDF powders with a weight ratio of 8:1:1 in NMP. The slurry was then coated onto Al foil using a doctor blade and dried for 1 h at 60 °C. Afterwards, the coated-Al foil was cut into discs (MSK-T10, Shenzhen Kejing Star Technology Co., Ltd.; 10 mm in diameter) and dried overnight at 90 °C under vacuum. The commercial high-loading NCM811 positive electrodes ( ~ 16.5 mg cm^−2^) were purchased from Changzhou Qianmu New Energy Co., Ltd, and they were used as received. According to the supplier, the electrode composition is 90 wt% active material, 5 wt% conductive carbon powder, and 5 wt% PVDF powder. The mass loading of conventional LFP positive electrode pieces was 1–2 mg cm^−2^, while that of the high mass loading pieces reached ~10.5 mg cm^−2^. For NCM811, the conventional mass loading was 3–4 mg cm^−2^, and the high loading ones reached ~16.5 mg cm^−2^. All the positive electrodes employed in coin cells are single-side coated with relevant active materials.

### Electrochemical measurements

All electrochemical evaluations (except pouch cells) were conducted using CR2032 coin cells (the spacer, spring, and case are all made of stainless steel) assembled under rigorously controlled conditions. A polypropylene microporous separator (Celgard 3501, thickness 25μm, diameter 16 mm) was placed between the electrodes, and 50 µL of electrolyte was added to each cell using a micropipette to ensure uniform distribution. The cell assembly was carried out in a high-purity argon-filled glovebox, followed by a 6-hour aging at 25 °C to allow complete electrolyte saturation before electrochemical measurements. Unless otherwise specified, all electrochemical measurements were carried out at 25 °C, and all galvanostatic charge/discharge tests were performed using a Neware battery cycler (CT-4008) in a climate chamber at 25°C. Prior to the long-term Li plating/stripping evaluation, the Li | |Li symmetric cells were subjected to two formation Li plating/stripping steps at 0.2 mA cm^−2^ and 1.0 mAh cm^−2^, aiming to establish a stable solid electrolyte interphase (SEI) layer. For the Li | |Cu half cells, the initial activation was performed by cycling at 0.05 mA cm^−2^ for five cycles from 0 V to 1.0 V, so as to stabilize the SEI and remove impurities from the electrode. The Li | |LFP (1 C = 170 mA g^−1^) and Li | |NCM811 (1 C = 200 mA g^−1^) full cells were tested within voltage windows of 2.5–4.0 V and 2.8–4.3 V, respectively, unless otherwise specified. For the Li | |LFP cells, the rate capability test at 5 C was started after pre-cycling at 0.2 C, 1 C, and 2 C for two cycles each. For the 20 C, 40 C, and 80 C rate tests, the cells were pre-cycled at 0.2 C, 1 C, 2 C, 5 C, and 10 C for two cycles each, followed by long-term cycling at the target rate (20 C, 40 C, or 80 C, respectively). For the Li | |NCM811 cells, the 1 C test was performed after pre-cycling at 0.1 C for two cycles; the 2 C test was conducted after pre-cycling at 0.1 C and 0.5 C for two cycles each; and the 10 C rate performance was tested after pre-cycling at 0.1 C, 0.5 C, and 2 C for two cycles each. For the Li | |NCM811 pouch cells, assembly was carried out in a dry room (dew point <−60 °C, temperature 10 °C). The electrolyte was injected using a pipette, and the pouch cells were then vacuum-sealed at 170 °C under −90 kPa to remove residual gas. An external pressure of 0.35 MPa was applied during the cycle process. All pouch cells have been pre-cycled at 0.1 C for two cycles before the start of 0.2 C charge / 0.5 C discharge cycling. The specific energy was calculated based on the total mass of the pouch cell, including the packaging (see Supplementary Tables 13 and 14 for detailed parameters). The cycle performance of the cells was tested using Neware Battery Measurement System (MIHW-200-160CH-B, Shenzhen, China). The Aurbach method^35,36^ was used to test the Coulombic efficiency (CE) of the batteries. Specifically, 5.0 mAh cm^−2^ of Li was initially deposited onto a Cu foil at a current density of 0.5 mA cm^−2^, followed by charging to 1.0 V to clean the Cu surface. Next, a Li reservoir (*Q*_T_) was deposited onto the Cu foil and then stripped to 1.0 V. Galvanostatic cycles were then conducted with a fixed areal capacity (*Q*_C_) at a desired current density for a set number of cycles. Finally, the remaining Li was stripped to 1.0 V. The CE was calculated using the following formula:

1 $$CE=\frac{n{Q}_{{{{\rm{c}}}}}+{Q}_{{{{\rm{R}}}}}}{n{Q}_{{{{\rm{c}}}}}+{Q}_{{{{\rm{T}}}}}}$$

where *Q*_R_ indicates the remaining capacity in the final stripping step.

A BioLogic VSP 300 electrochemical workstation was employed for linear sweep voltammetry (LSV), cyclic voltammetry (CV), electrochemical impedance spectroscopy (EIS), Tafel plots and Galvanostatic intermittent titration technique (GITT). GITT measurements were performed with a data acquisition interval of 30 seconds. EIS measurements were performed from 100 kHz to 100 mHz at an amplitude of 10 mV (6 points per decade). Before each measurement, the cells were rested for 6 h EIS data collected at various temperatures were then fitted, and the activation energy (*E*_a_) was then calculated using the Arrhenius equation^48^:

2 $$\frac{T}{{R}_{(SEI/CT)}}=A\,\exp (-\frac{{E}_{(a1/a2)}}{RT})$$

where *T* is the absolute temperature, *R*_(SEI/CT)_ is the resistance, *A* is the pre-exponential factor, *R* is the gas constant. *E*_a_ can be extracted from the data fitting of ln(*T*/*R*_(SEI/CT)_) vs 1/*T*.

### Material characterizations

The viscosity of the electrolyte was measured using a rotational viscometer (NDJ-8S, Shanghai Precision & Scientific Instrument Co., Ltd.) under thermostatically controlled conditions at room temperature. The wettability was quantitatively evaluated by measuring the static contact angle between the electrolyte and the Celgard 3501 separator using a tensiometer (OSA 100, LAUDA Scientific GmbH). Raman spectroscopy (Renishaw inVia) was employed to analyze the electrolyte’s solvation structure using a 532 nm laser over a spectral range of 100–4000 cm^−1^ with 3 accumulations. The ^7^Li and ^19^F NMR spectra were recorded on a 500 MHz Bruker Nuclear magnetic resonance (NMR) spectrometer at 11.7 T and 20°C. A 1 M LiCl solution served as the internal standard for ^7^Li NMR, with the Li chemical shift referenced to 0 ppm. The surface morphology and microstructure were examined using a field emission scanning electron microscope (SEM, HITACHI SU8010) and atomic force microscope (AFM, Bruker Dimension Icon). AFM measurements were performed in Peakforce QNM mode using a silicon antimony‑coated cantilever (RTESPA‑525, Bruker) with a tip radius of ~8 nm. The dendrite growth process during cycling was monitored in real time through in situ optical microscopy (YUESCOPE YD650). To investigate the cathode electrolyte interphase (CEI) formed after cycling, NCM811 powders were carefully scraped from the Al foil, ultrasonically dispersed in an alcohol solution, and subsequently deposited onto a Cu grid for transmission electron microscopy (TEM, FEI Tecnai G2 F30) analysis. The chemical composition of SEI and CEI layers were investigated through X-ray photoelectron spectroscopy (XPS, Kratos AXIS Supra + ) using a monochromatic Al Kα source operated at 14.5 kV and 15 mA, and the C1 *s* peak was calibrated to 284.8 eV. Ni, Co and Mn deposited on Li disks were dissolved in a 2 wt% HCl solution and measured using an inductively coupled plasma mass spectrometry (ICP-MS, Agilent 7850). All cells were systematically disassembled within an argon-filled glovebox at 25 ± 5 °C, and the electrodes were rinsed with DMC to remove the residual electrolyte and impurities.

### Theoretical calculations

Molecular dynamics (MD) simulations were performed using GROMACS 2024 with the OPLS-AA force field to investigate the electrolyte solvation structures. The EDF electrolyte model was composed of 80 LiPF_6_, 588 EC, 449 DMC, and 52 FEC molecules, while the EDF-3SF/LNO electrolyte model contained 80 LiPF_6_, 588 EC, 449 DMC, 52 FEC, 8 LiNO_3_, and 40 3SF molecules. Solvent molecules were characterized by the 1.2CM5 charge obtained from Multiwfn software^67^, while the charges of PF_6_^−^ and NO_3_^−^ were referenced from the literature^68,69^. The charges of cations and anions were scaled by 0.9 to mimic the polarization effects in the solvent^68^. The initial configuration of all simulation systems was obtained by uniformly mixing the components using the Packmol software package. The energy minimization was performed using the steepest descent method for 3000 steps to remove any unreasonable atomic overlaps. Subsequently, at 298.15 K, the system was relaxed for 100 ps under NVT and NPT ensembles, respectively, with an integration time step of 1.0 fs. A production simulation of 20 ns was then performed under the NPT ensemble. Periodic boundary conditions were applied in all three directions in the MD simulations, with a time step of 2.0 fs. The temperature was maintained at 298.15 K using the V-rescale thermostat with a coupling time of 0.5 ps, while the pressure was controlled at 1 bar using the Parrinello-Rahman barostat with a coupling time of 2.0 ps. The LINCS algorithm was used to constrain all bonds involving hydrogen atoms. Long-range electrostatic interactions were treated using the Particle-Mesh Ewald method, with short-range electrostatic and van der Waals interactions calculated using a cutoff of 1.2 nm. The post-processing of simulation trajectories was performed using GROMACS, while visualization and analysis were carried out using VMD 1.9.3 software.

DFT calculations were performed to further investigate the binding energies and energy level profiles of electrolyte components. The B3LYP hybrid functional, implemented in the Gaussian 16 software package, was employed for the calculations. Geometric optimization and frequency analysis of the molecules were carried out at the B3LYP/6-311 + + G (d, p) level to obtain the optimized structures and confirm that they correspond to energy minima. The binding energies of SCC-Li^+^-NO_3_^−^ were calculated based on this computational level:

3 $${E}_{{bind}}={E}_{{complex}}-{E}_{({molecular}\; {fragment})}-{E}_{({ion}\; {fragment})}$$

where *E*_complex_ is the total energy of the complex formed by Li^+^, NO_3_^−^, and the SCC, *E*_(molecular fragment)_ is the energy of the isolated SCC molecule, and *E*_(ion fragment)_ is the combined energy of the isolated Li^+^ and NO_3_^−^ ions. The energy level information was computed at the B3LYP-D3(BJ)/def2TZVPP level, while the electrostatic potential (ESP) was calculated using the embedded module in Gaussian. To model solvent effects, the Solvation Model based on Density (SMD) was applied.

## Supplementary information

Supplementary Information

Description of Additional Supplementary Files

Supplementary Data 1-3

Supplementary Movie 1

Supplementary Movie 2

Transparent Peer Review file

## Source data

Source Data

## Data Availability

All relevant data are included in this article and its Supplementary Information files. Source data are provided with this paper. Further information is available from the corresponding author upon request. Source data are provided with this paper.
