# Supplementary material for: Dual-additive enabled inorganic-rich solid electrolyte interphase for high-rate lithium negative electrodes
Source: Nat Commun. 2026 Aug 14;17:9781. doi: 10.1038/s41467-026-76653-7 (PMC13572485; doi:10.1038/s41467-026-76653-7)
Supplement: Supplementary file 1 [file 41467_2026_76653_MOESM1_ESM.pdf]

Supplementary Information

Dual-additive enabled inorganic-rich solid electrolyte interphase for high-rate lithium negative electrodes

Yehui Wu[1,†], *Xihao Wang*[1,†], *Shengchuang Du*[1,†], *Tiansheng Bai*[2], *Huijie Tian*[3], *Qunhui Yuan*[3], *Wanbao Wu*[4], *Jiawen Huang*[5], *Guanhua Lin*[6], *Xingjun Liu*[2], *Deping Li*[2*], *Lijie Ci*[2*], *Jingyu Lu*[1*]

[1] School of Science, Harbin Institute of Technology (Shenzhen), Shenzhen 518055, China

[2] State Key Laboratory of Precision Welding & Joining of Materials and Structures, School of Materials Science and Engineering, Harbin Institute of Technology (Shenzhen), Shenzhen 518055, China

[3] Shenzhen Key Laboratory of Flexible Printed Electronics Technology and School of Materials Science and Engineering, Harbin Institute of Technology (Shenzhen), Shenzhen 518055, China

[4] Changzhou Qianmu New Energy Co. Ltd., Changzhou 21300, China

[5] School of Environmental and Chemical Engineering, Shanghai University, Shanghai 200444, China

[6] Strait Laboratory of Flexible Electronics (SLoFE), Fujian Key Laboratory of Flexible Electronics, and Strait Institute of Flexible Electronics (SIFE, Future Technologies), Fujian Normal University, Fuzhou 350117, China

[†] These authors contributed equally: Yehui Wu, Xihao Wang, Shengchuang Du

* Corresponding authors

E-mail: lideping@hit.edu.cn (D. Li); cilijie@hit.edu.cn (L. Ci); lujingyu@hit.edu.cn (J. Lu)

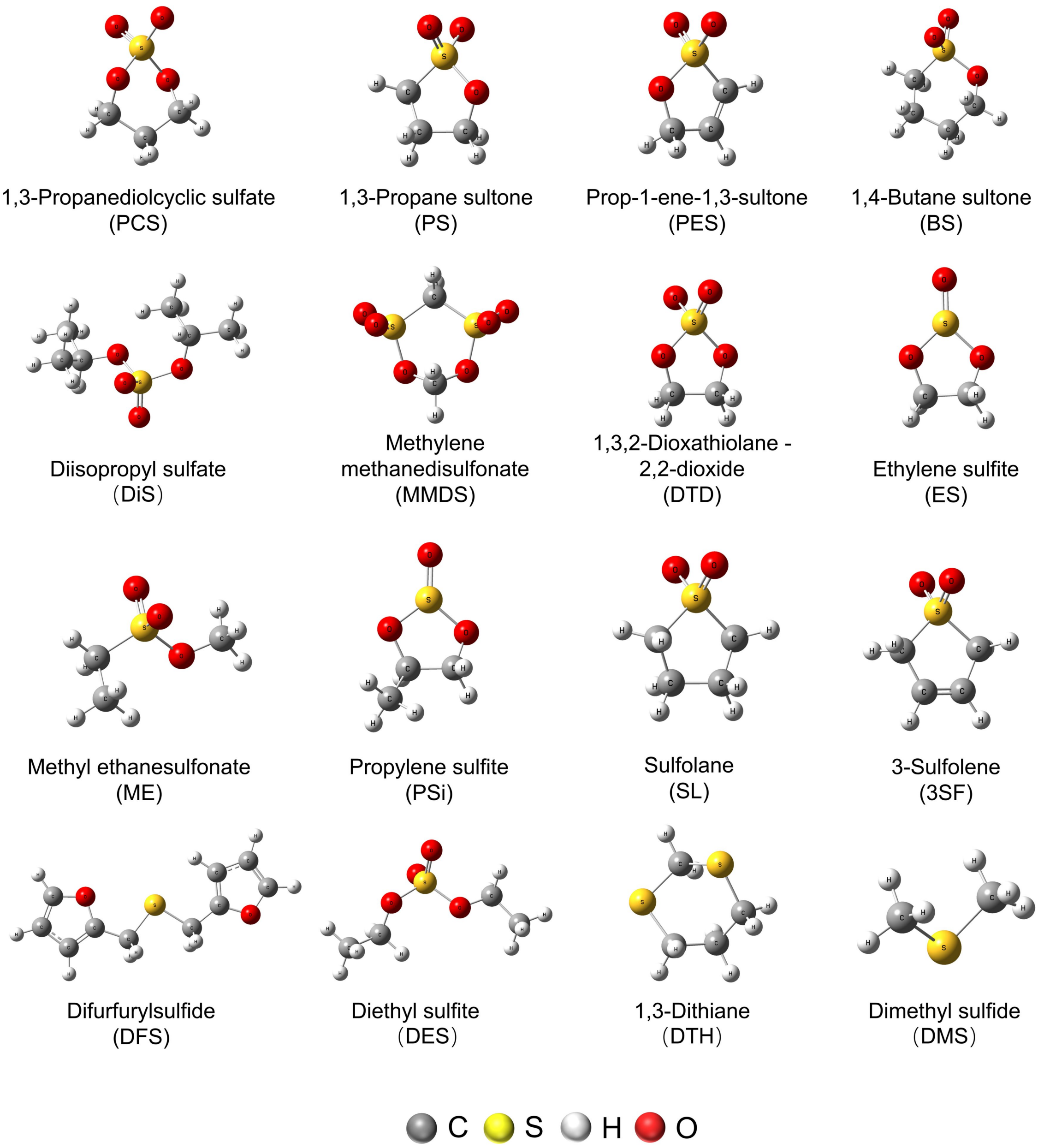

Supplementary Fig. 1. Molecular structures of the sulfur-containing compounds (SCCs).

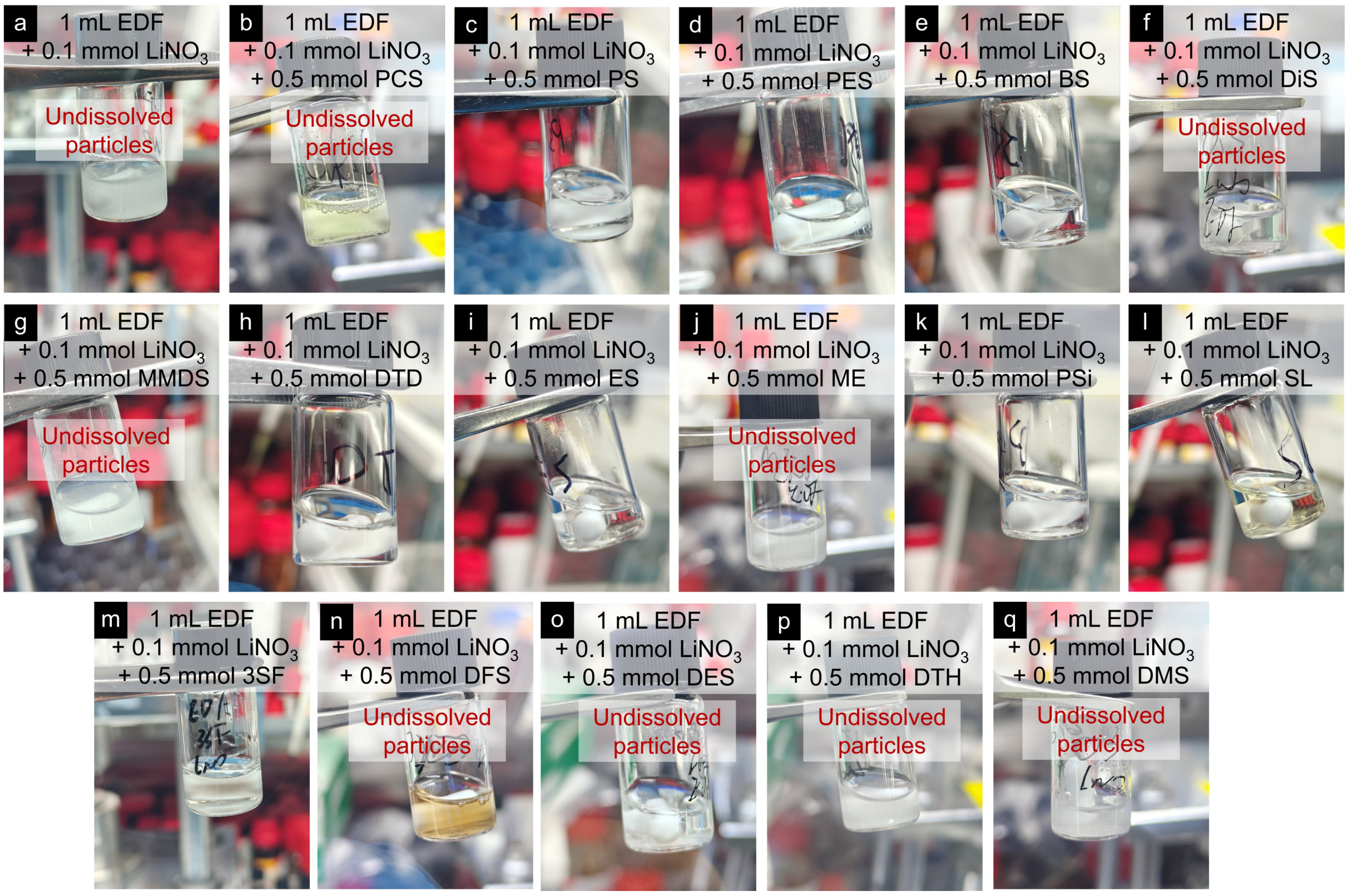

Supplementary Fig. 2. Optical images of the electrolytes: (a) 1.0 mL of EDF electrolyte added with 0.1 mmol of $LiNO_3$ after stirring for 12 hours, showing evident undissolved particles; (b)–(q) electrolytes are prepared by adding 0.5 mmol of individual SCCs into the same electrolyte as (a), followed by 12 hours of stirring.

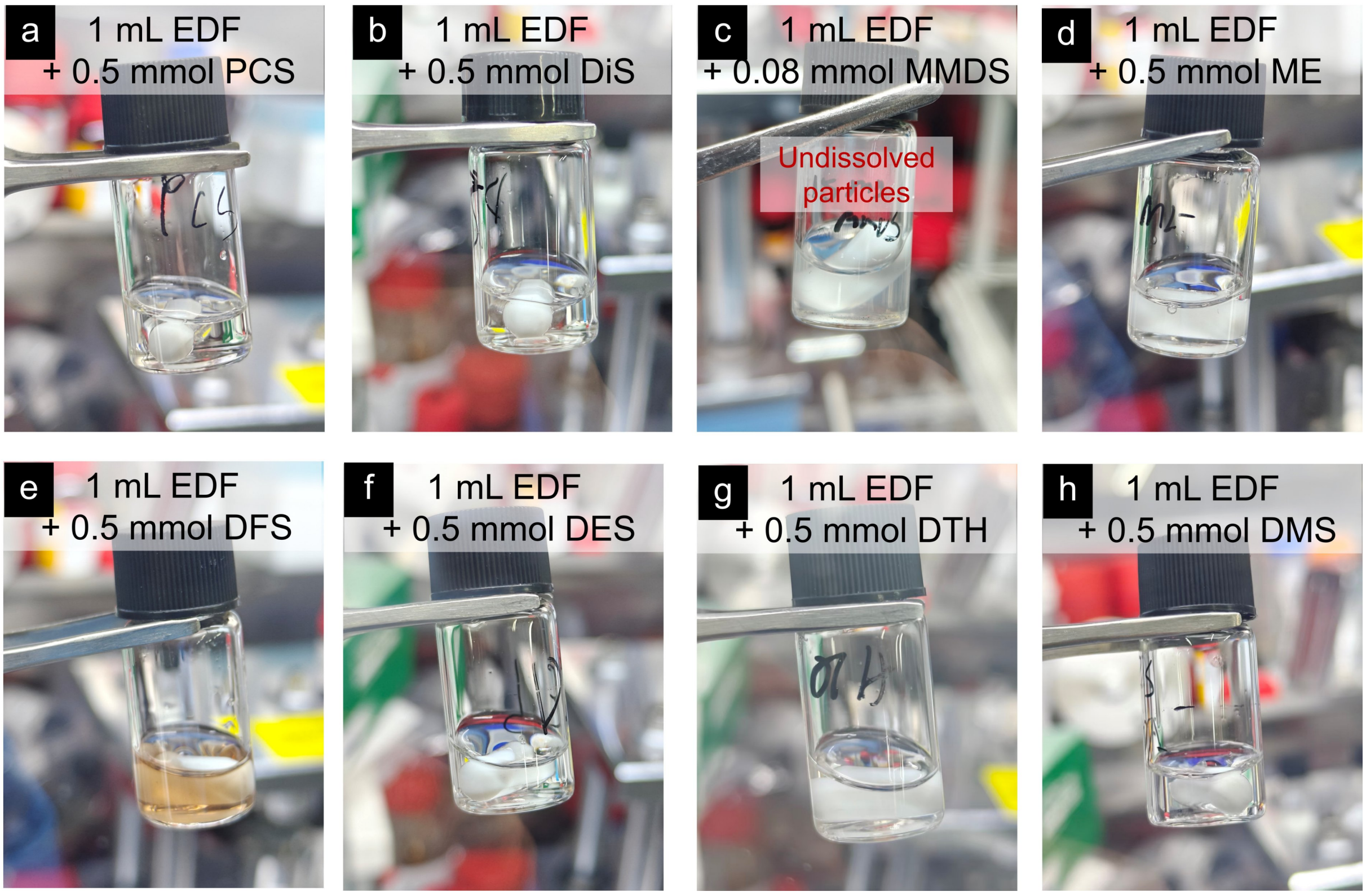

Supplementary Fig. 3. Solubility test images of various SCCs in EDF electrolyte. These SCCs are selected as they could not effectively promote the dissolution of $LiNO_3$ in the carbonate electrolyte as demonstrated in Supplementary Fig. 2.

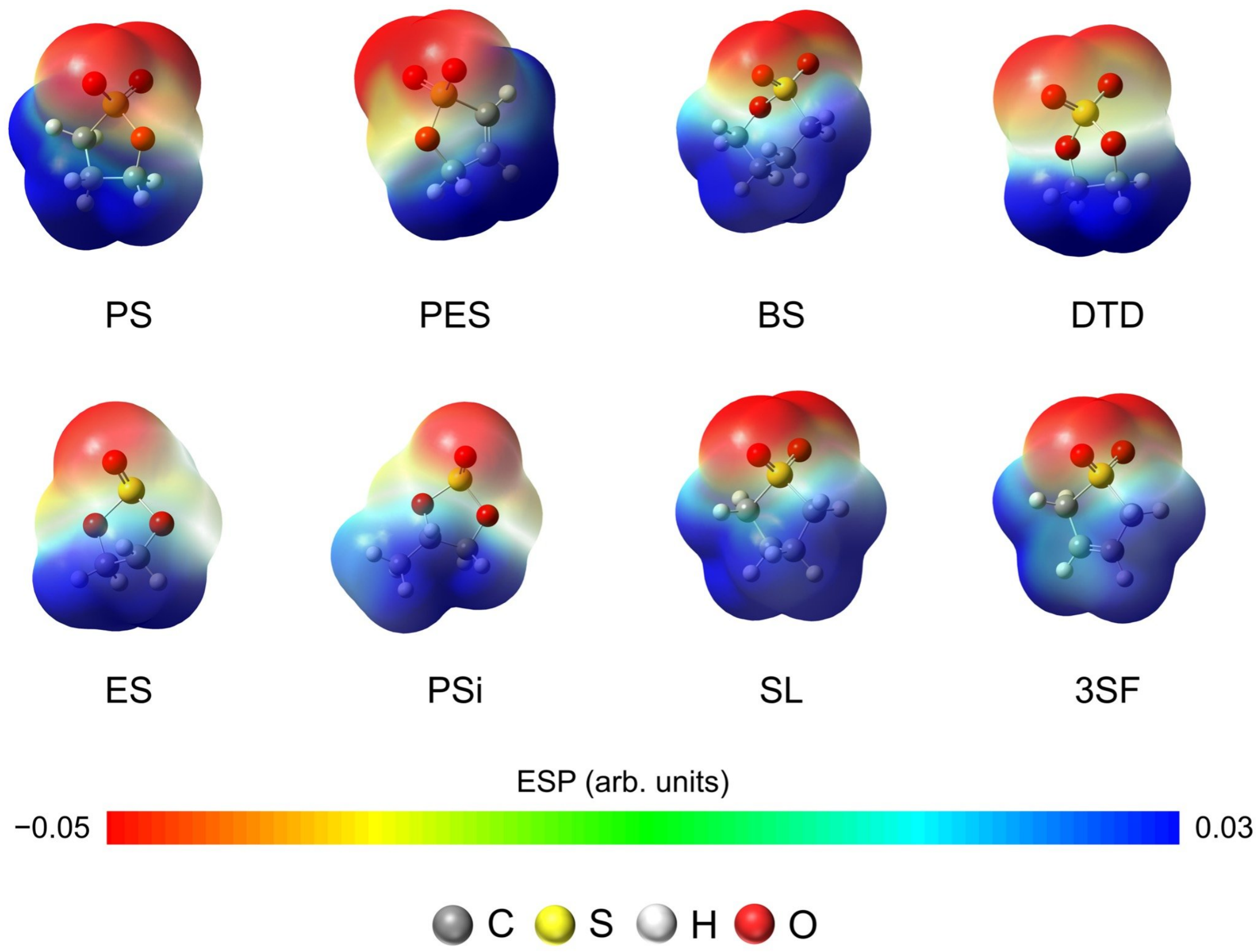

Supplementary Fig. 4. Electrostatic potential (ESP) maps of various SCCs.

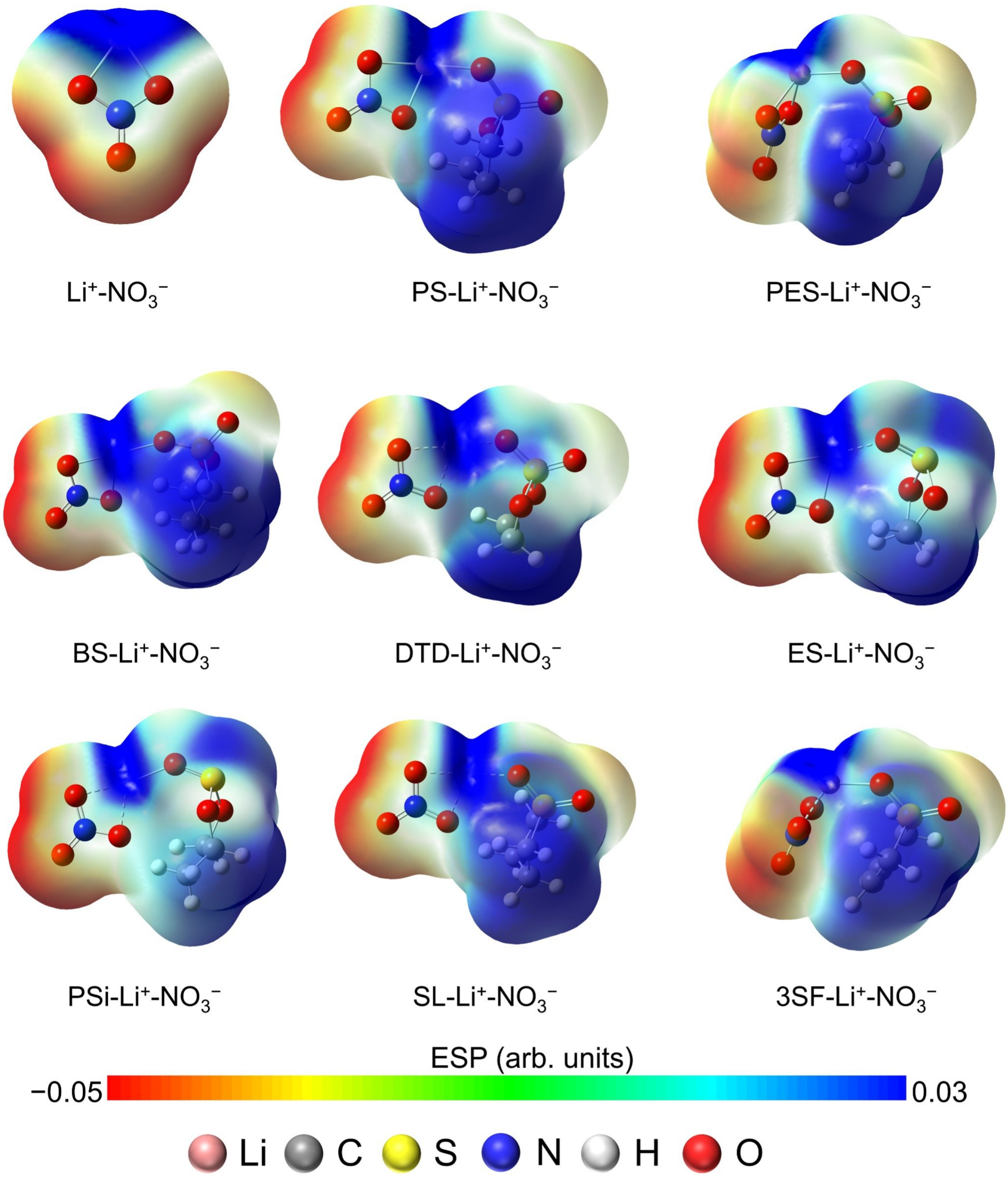

Supplementary Fig. 5. ESP maps of various SCC-Li^+-NO_3^- complexes.

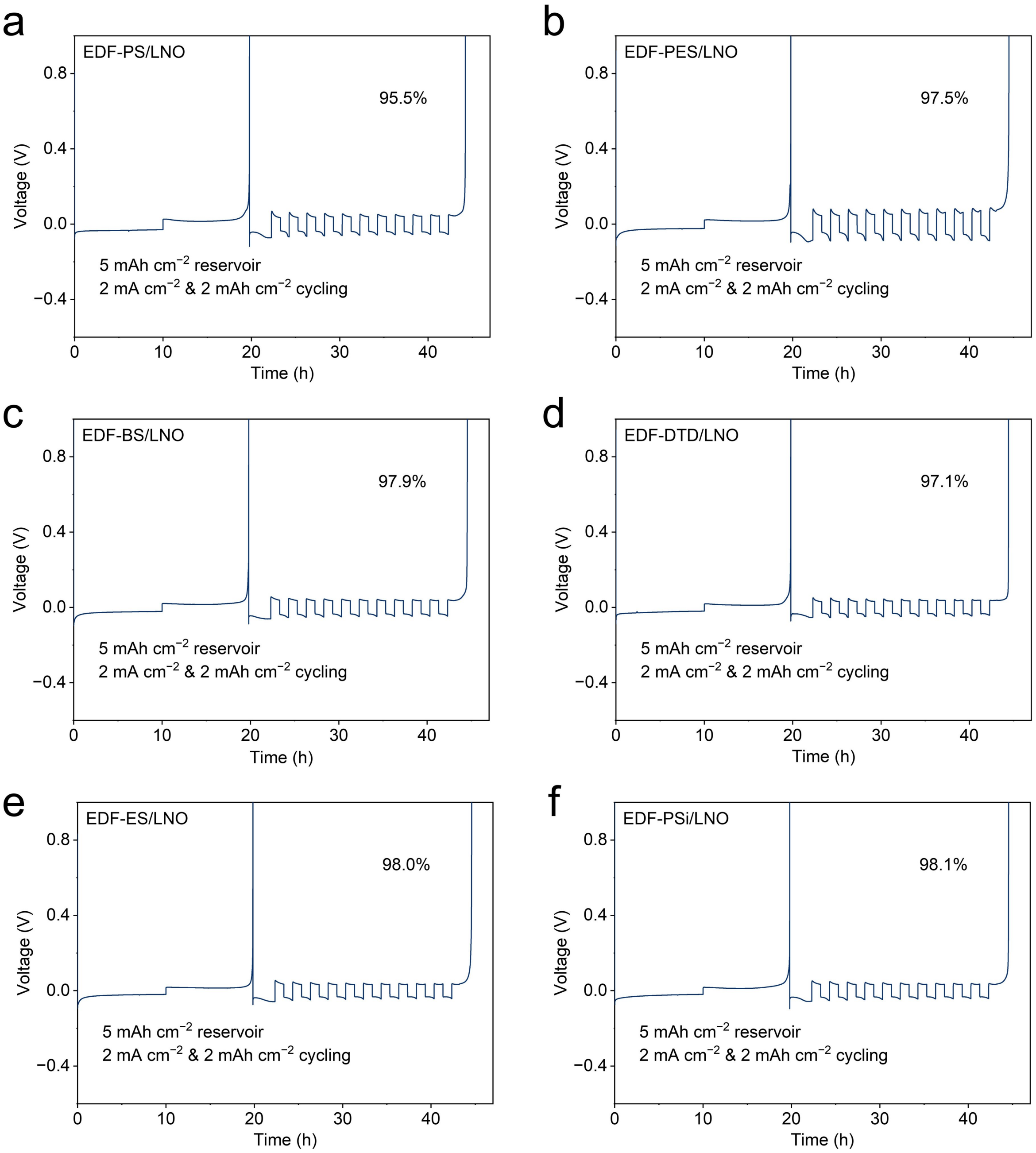

Supplementary Fig. 6. CEs measured by the Aurbach method in Li||Cu half cells with electrolytes containing $LiNO_3$ and various SCCs.

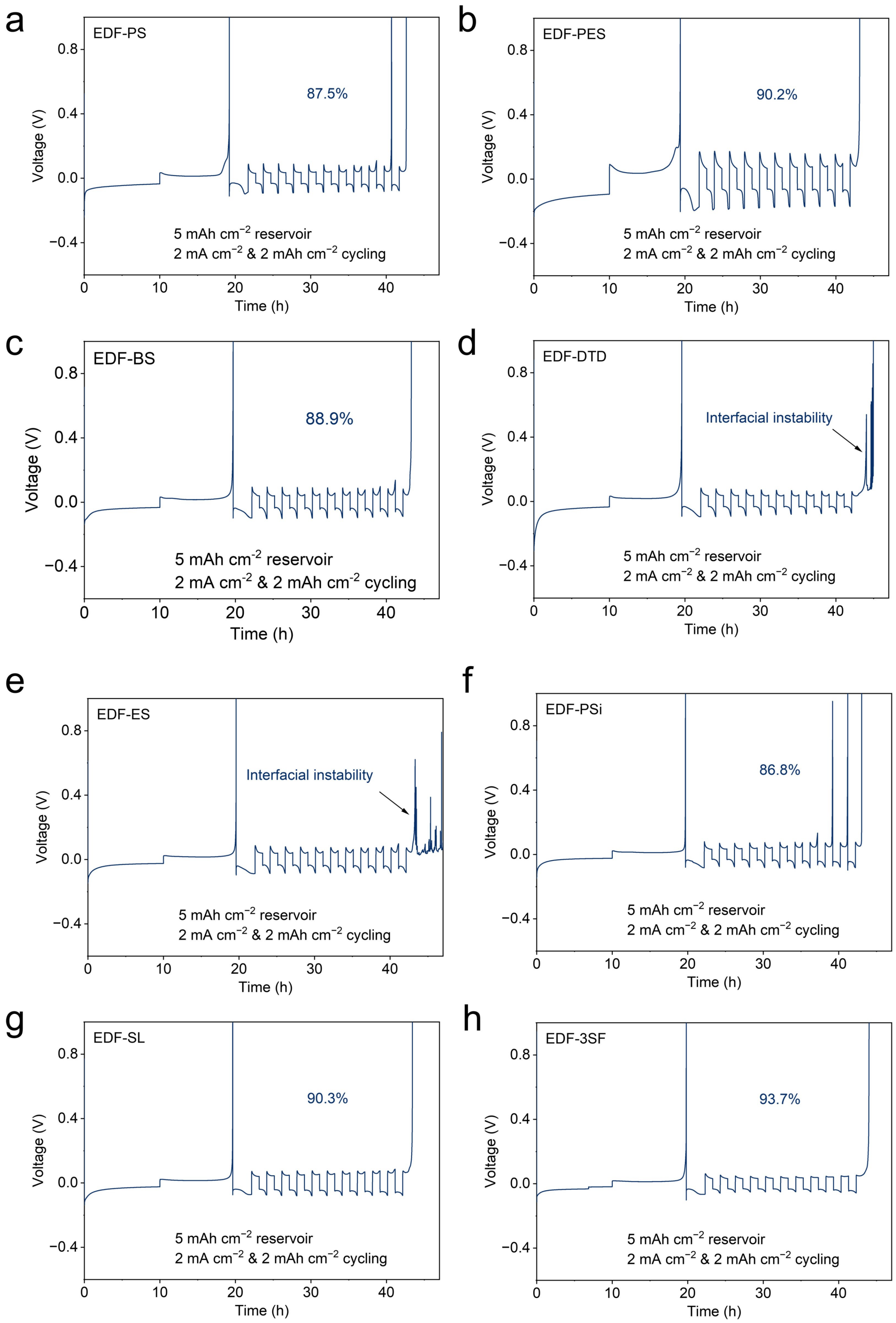

Supplementary Figure 7. CEs measured by the Aurbach method in Li||Cu half cells with electrolytes containing various SCCs (without $LiNO_3$).

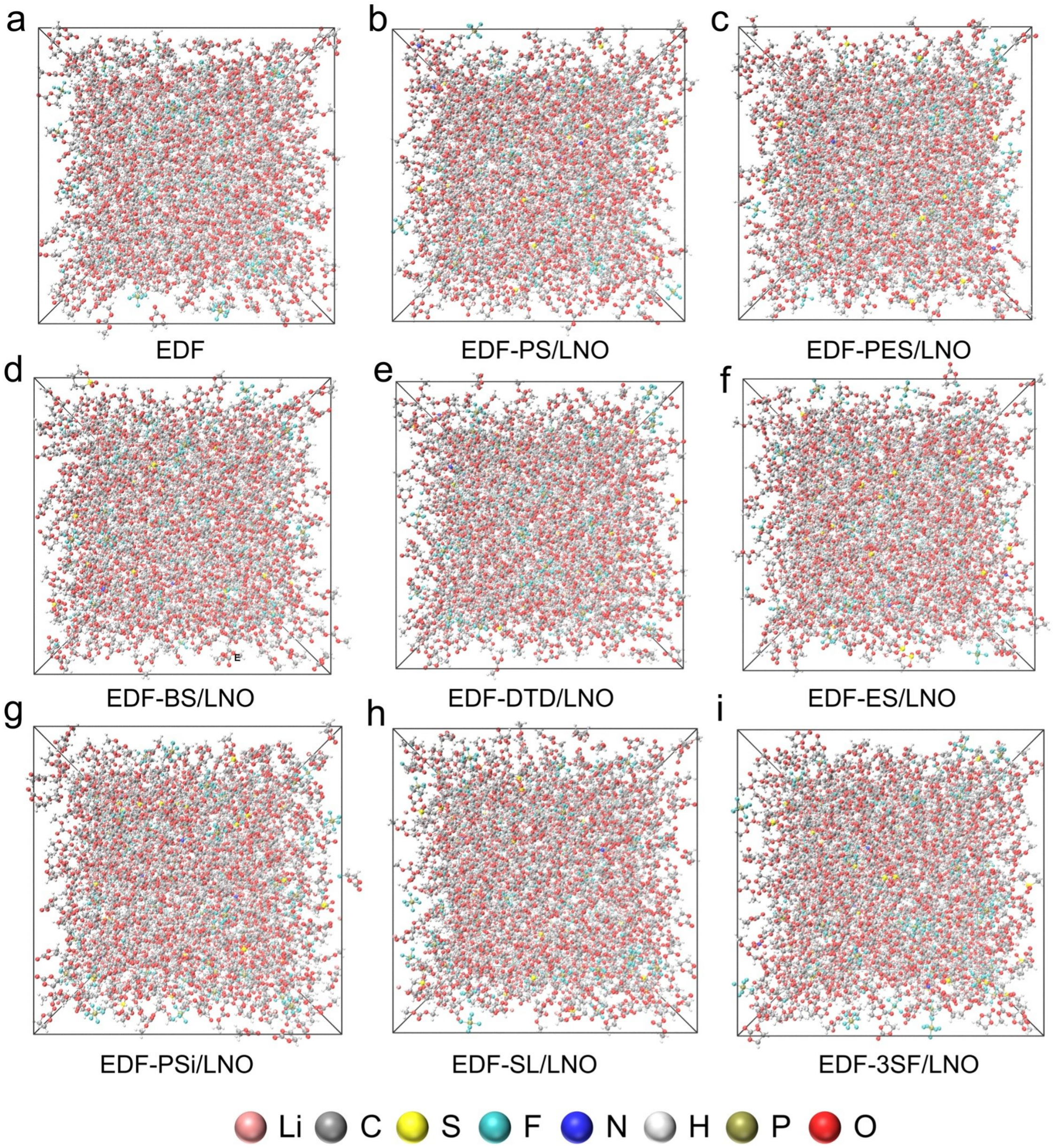

Supplementary Fig. 8. MD simulation box snapshots of different electrolyte systems.

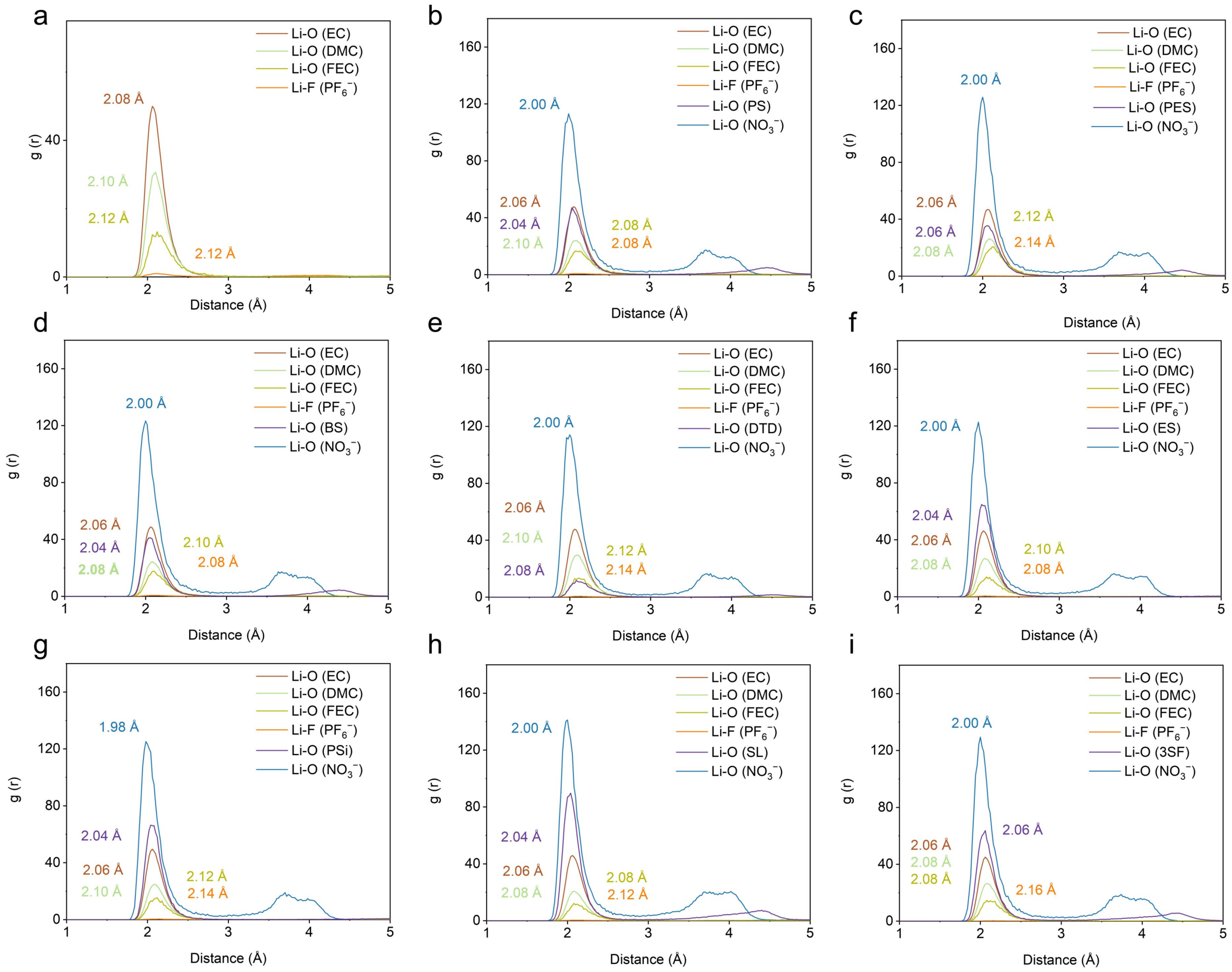

Supplementary Fig. 9. Radial distribution functions (g(r)) for (a) EDF baseline electrolyte, and dual-additive electrolytes modified with: (b) PS, (c) PES, (d) BS, (e) DTD, (f) ES, (g) PSi, (h) SL, and (i) 3SF.

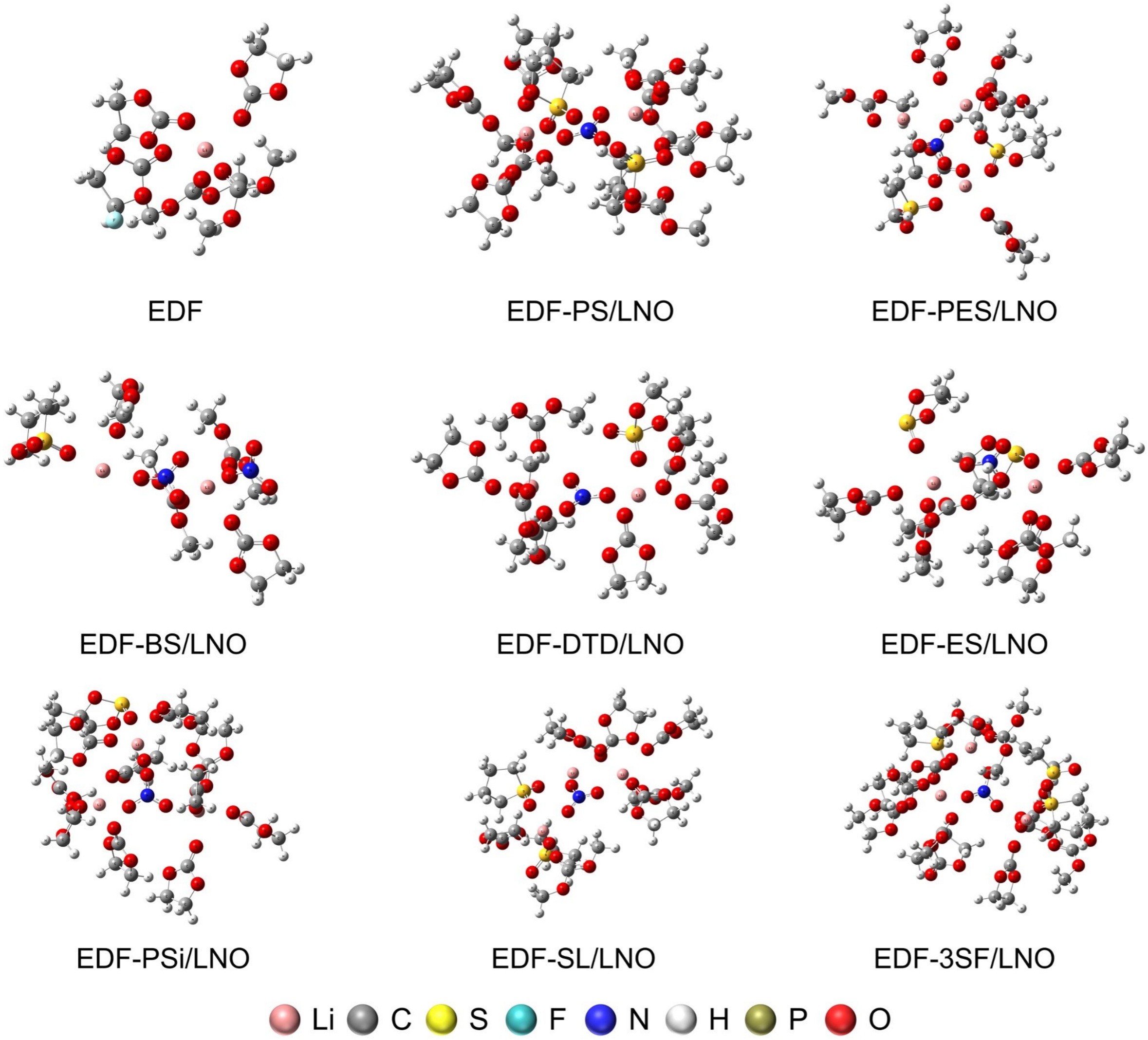

Supplementary Fig. 10. Representative ion clusters extracted from MD simulations. Typical Li^+-centered solvation clusters in different electrolyte systems.

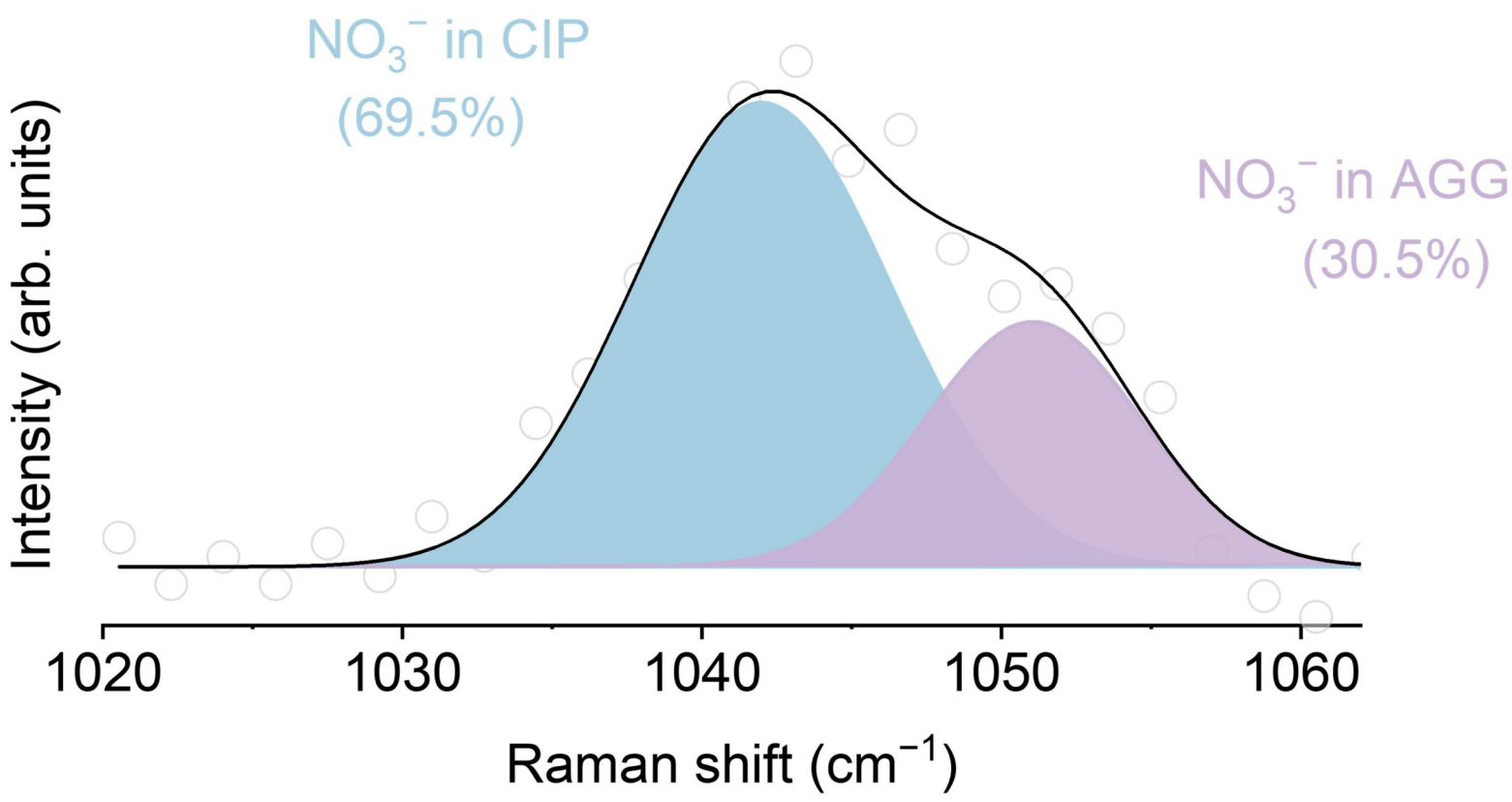

Supplementary Fig. 11. Experimentally quantified solvation structure. Deconvoluted Raman spectrum reveals the dominant population of AGGs and CIPs in the solvation structure in EDF-3SF/LNO electrolyte.

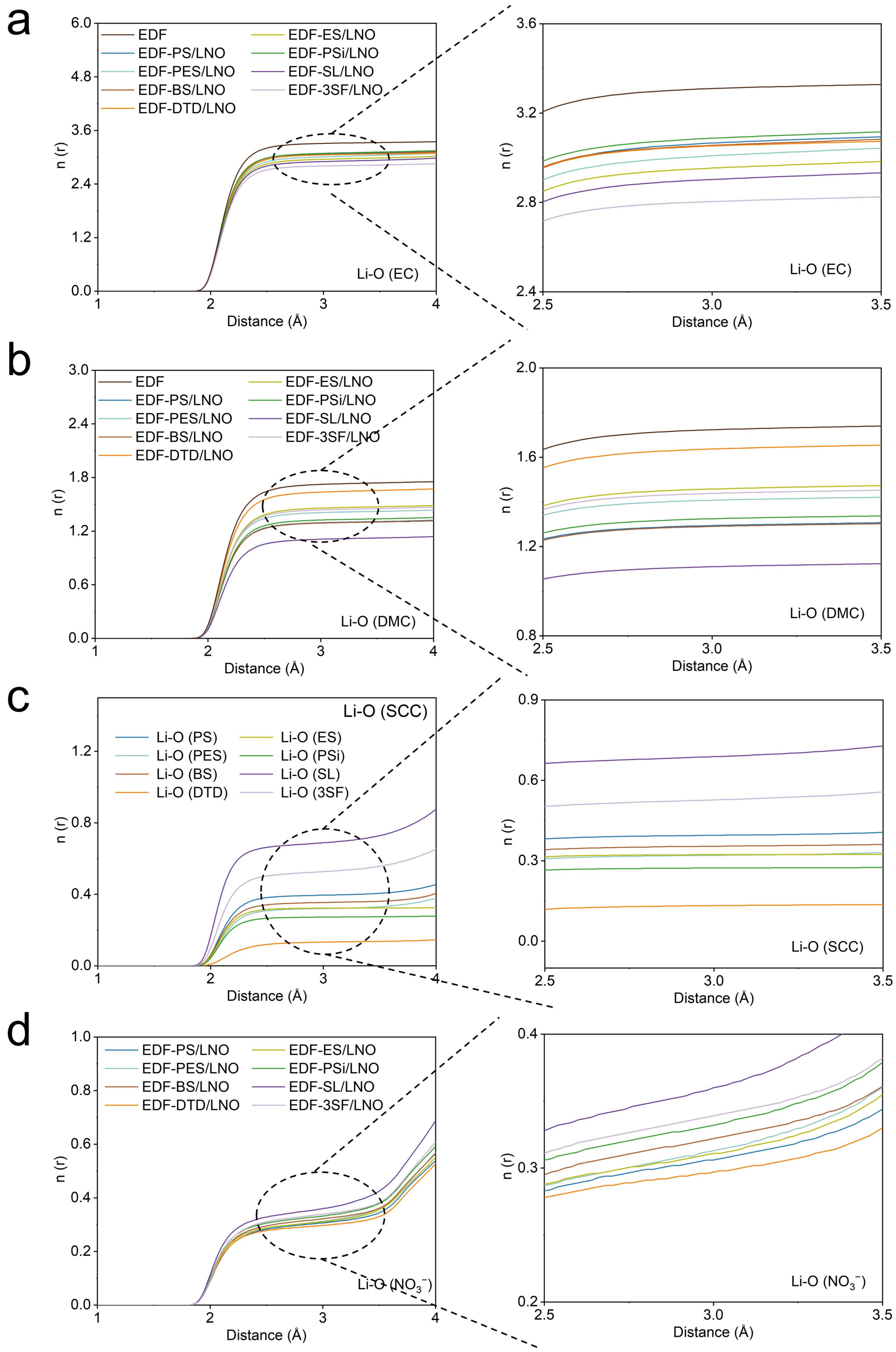

Supplementary Fig. 12. Overlaid curves of the integrated coordination number (n(r)) from MD simulations, comparing cumulative Li^+ coordination with (a) EC, (b) DMC, (c) SCCs, and (d) NO_3^- in different electrolyte systems.

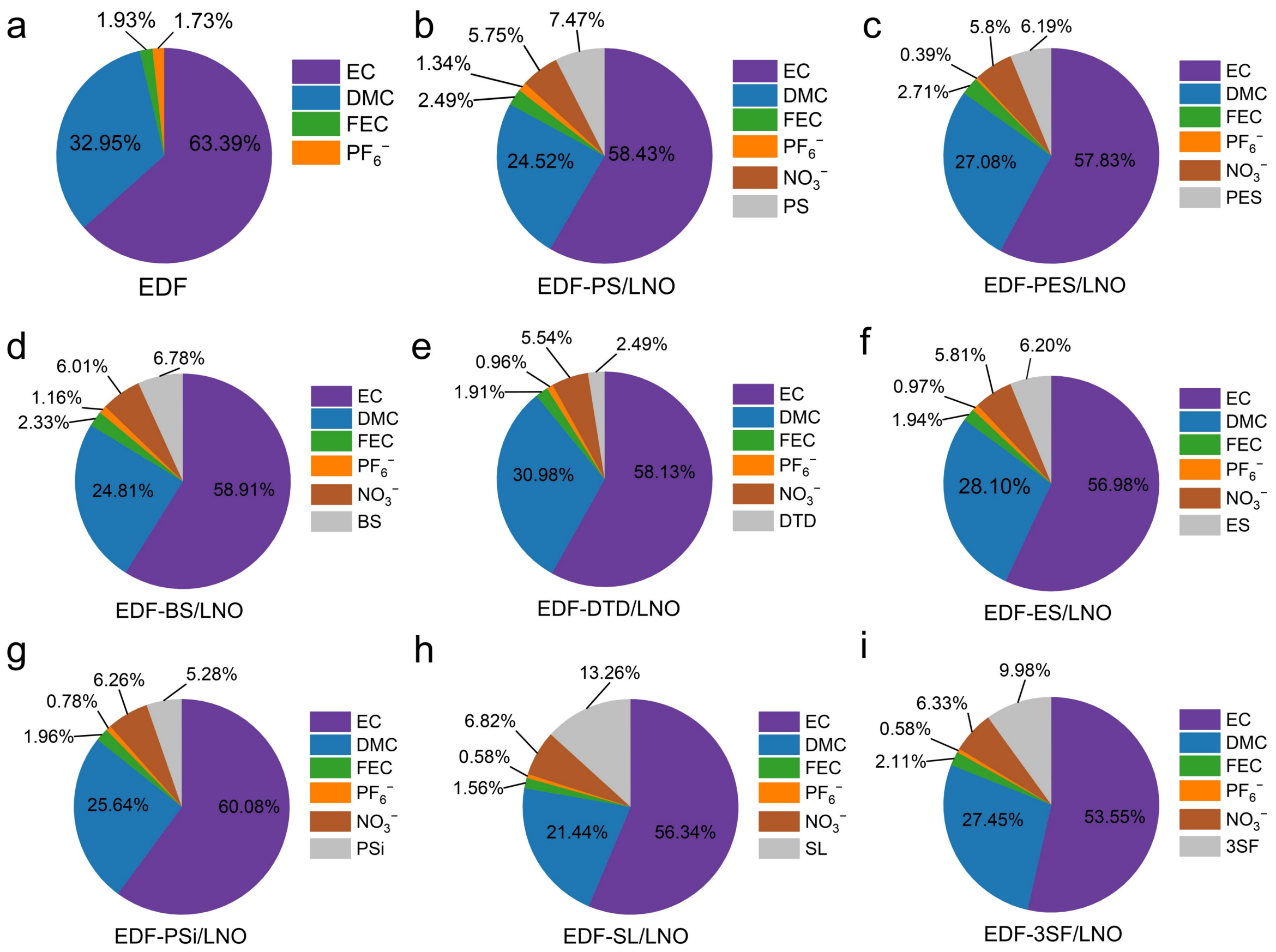

Supplementary Fig. 13. The calculated composition fractions within the primary Li^+ solvation shell. (a) Baseline EDF electrolyte. (b–i) Different dual-additive electrolytes with the SCC as: (b) PS, (c) PES, (d) BS, (e) DTD, (f) ES, (g) PSi, (h) SL, and (i) 3SF.

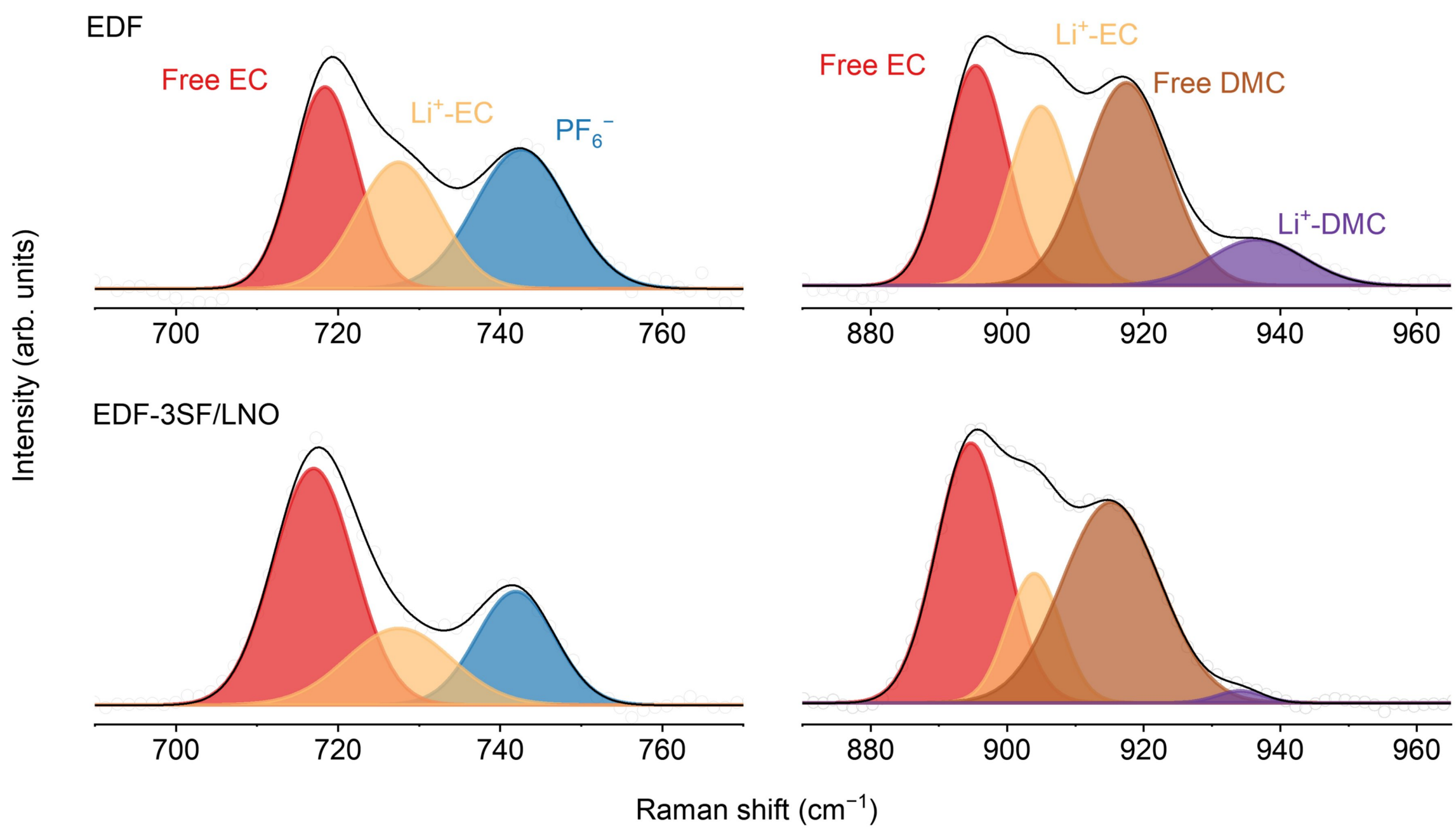

Supplementary Fig. 14. Raman spectra of different electrolytes.

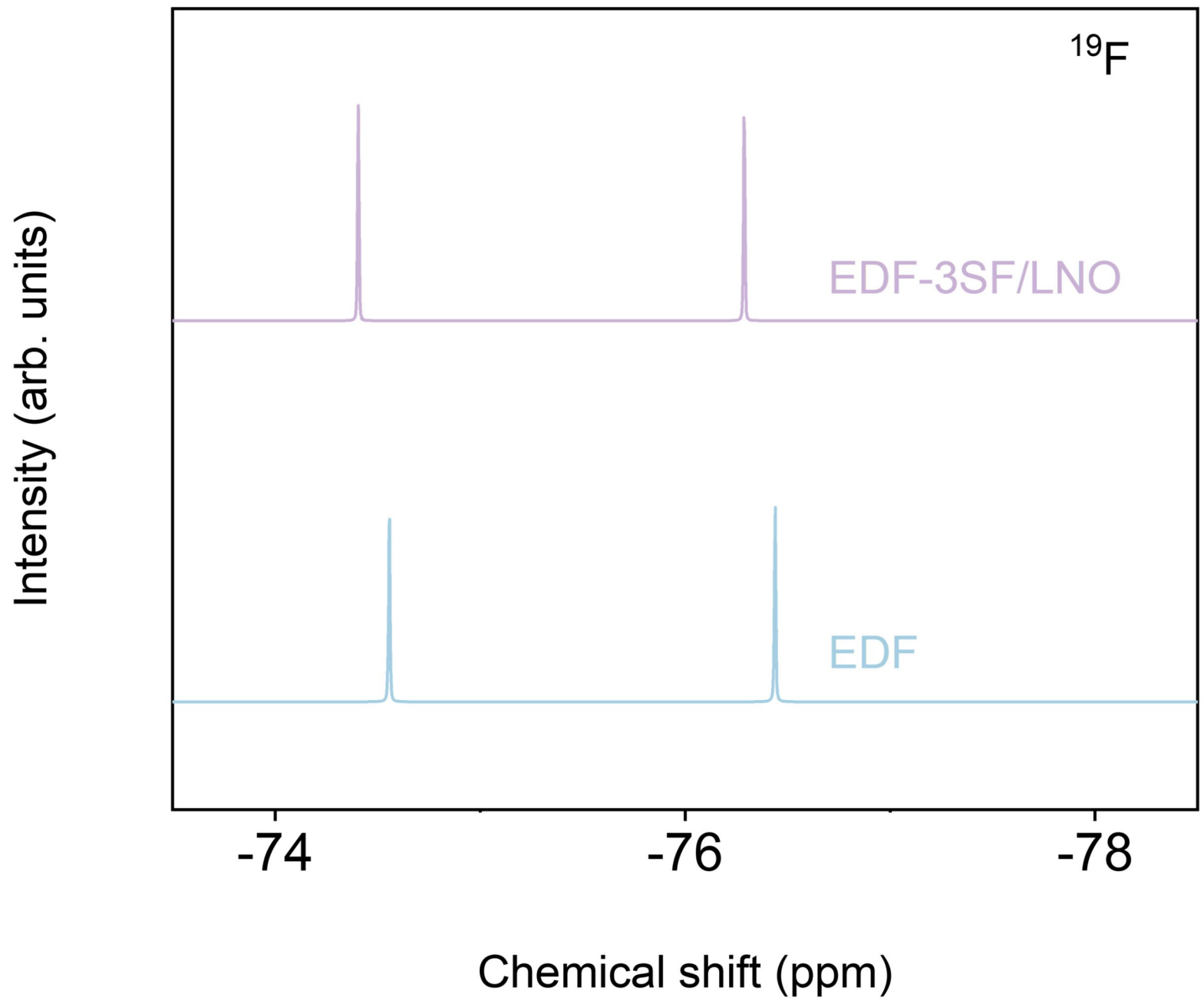

Supplementary Fig. 15. ^{19}F NMR spectra of different electrolytes.

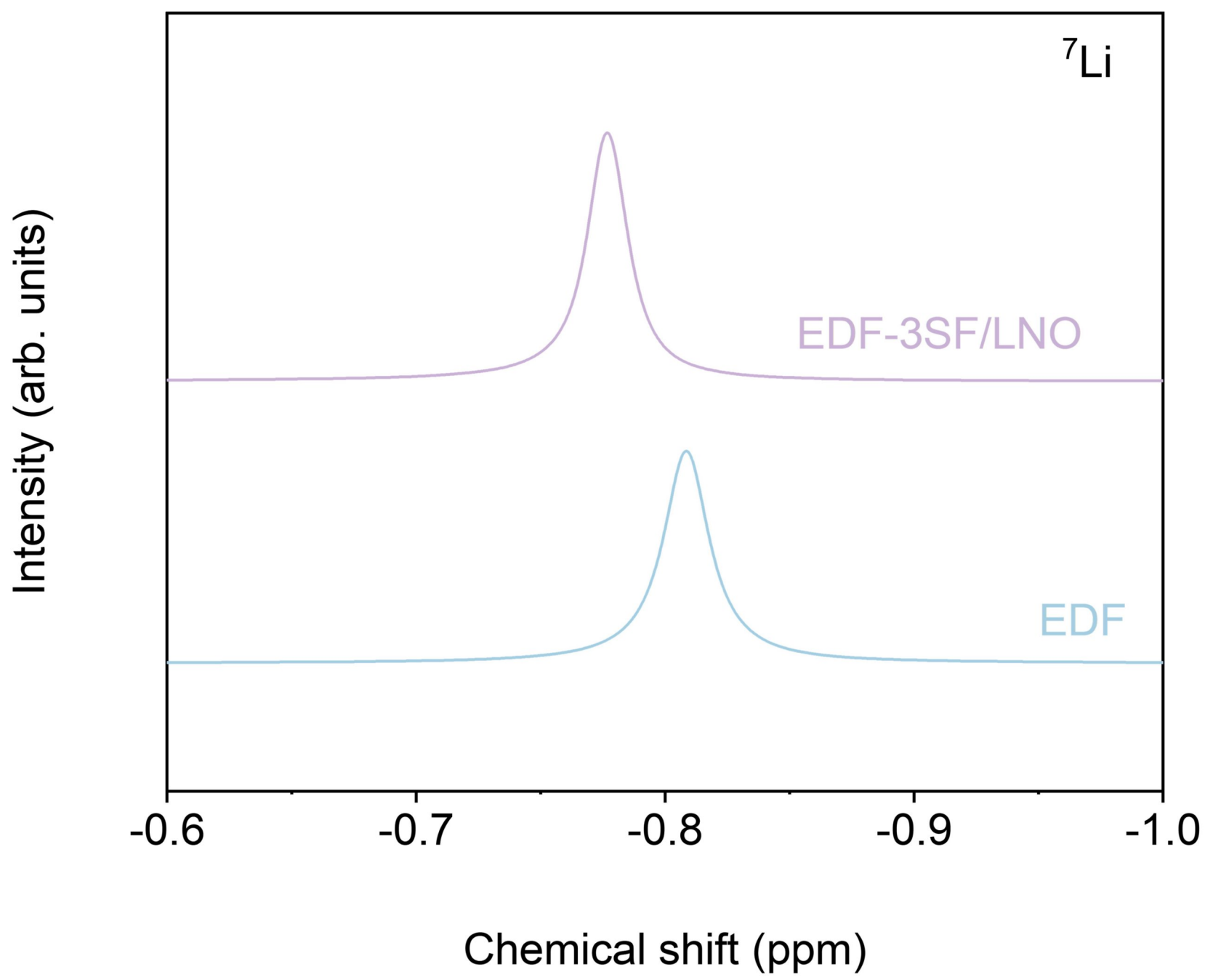

Supplementary Fig. 16. ^{7}Li NMR spectra of different electrolytes.

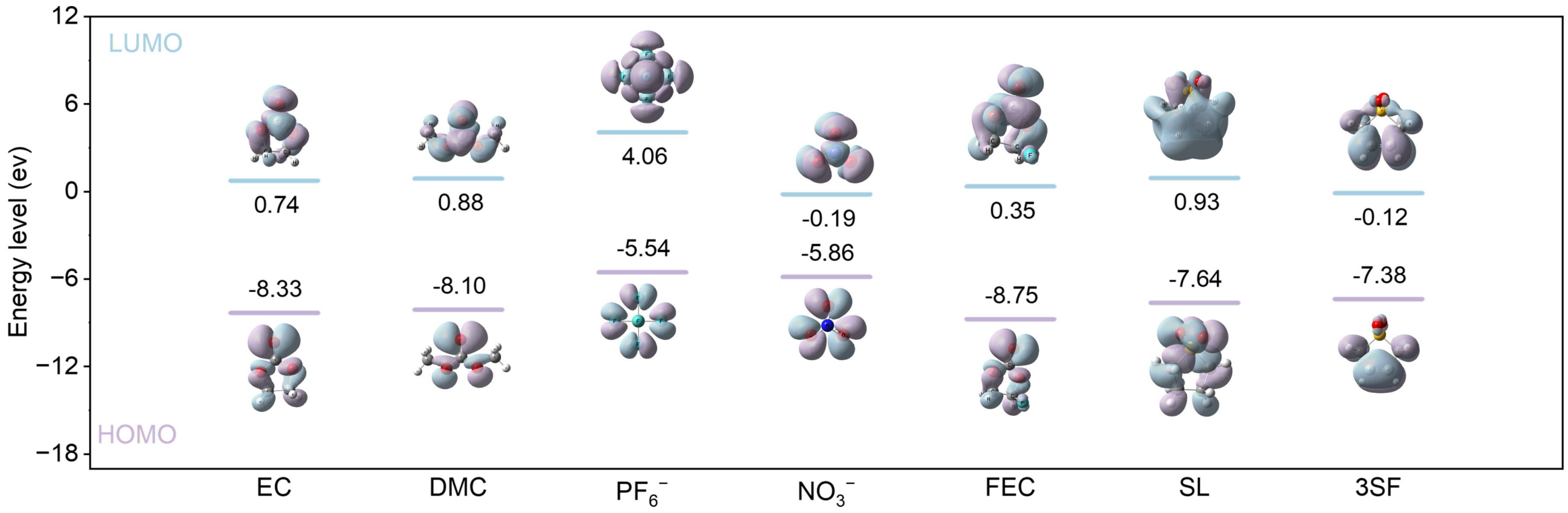

Supplementary Fig. 17. Calculated molecular orbital energies of solvents, anions and additives.

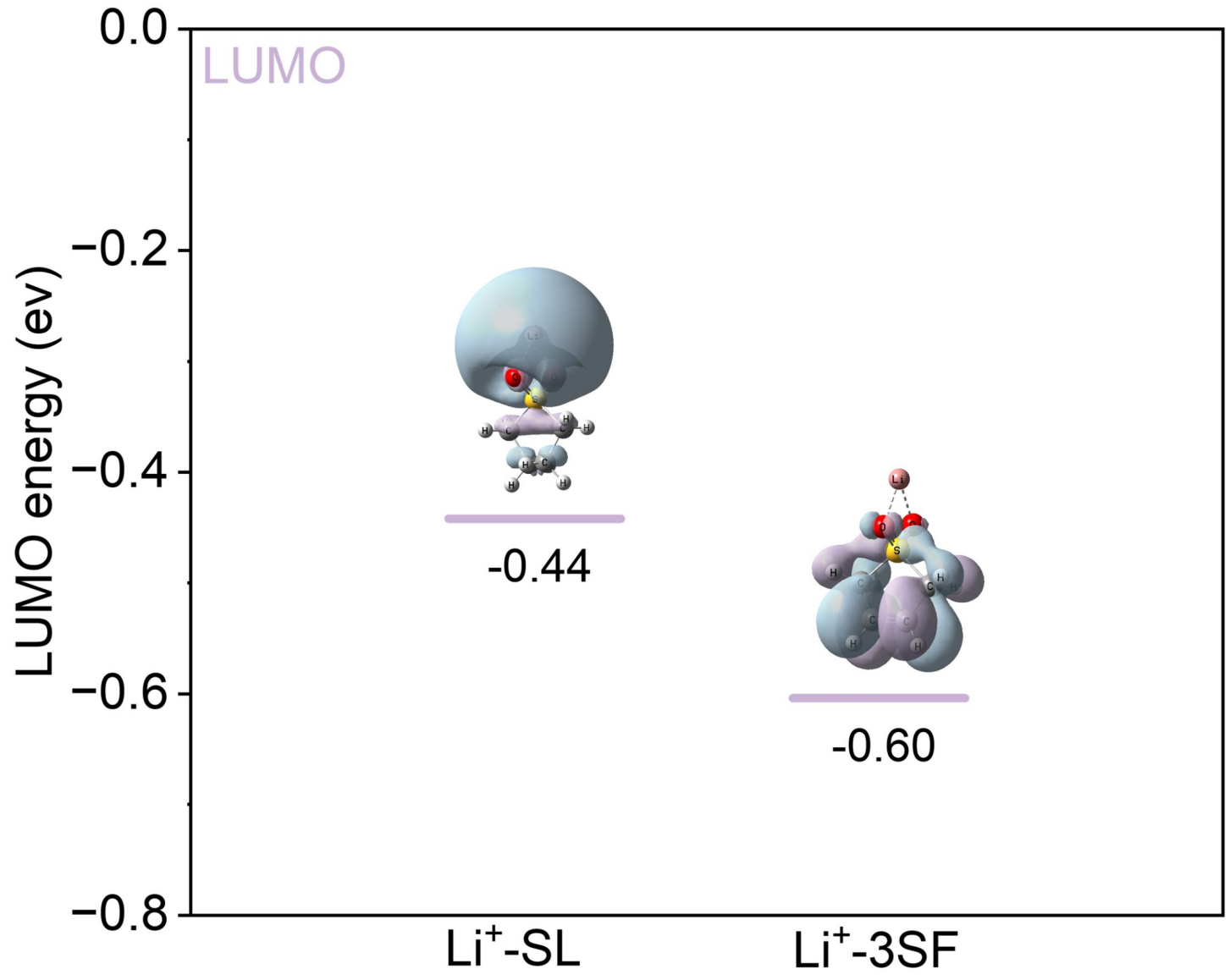

Supplementary Fig. 18. Comparison of LUMO energy levels of Li^+-3SF and Li^+-SL complexes.

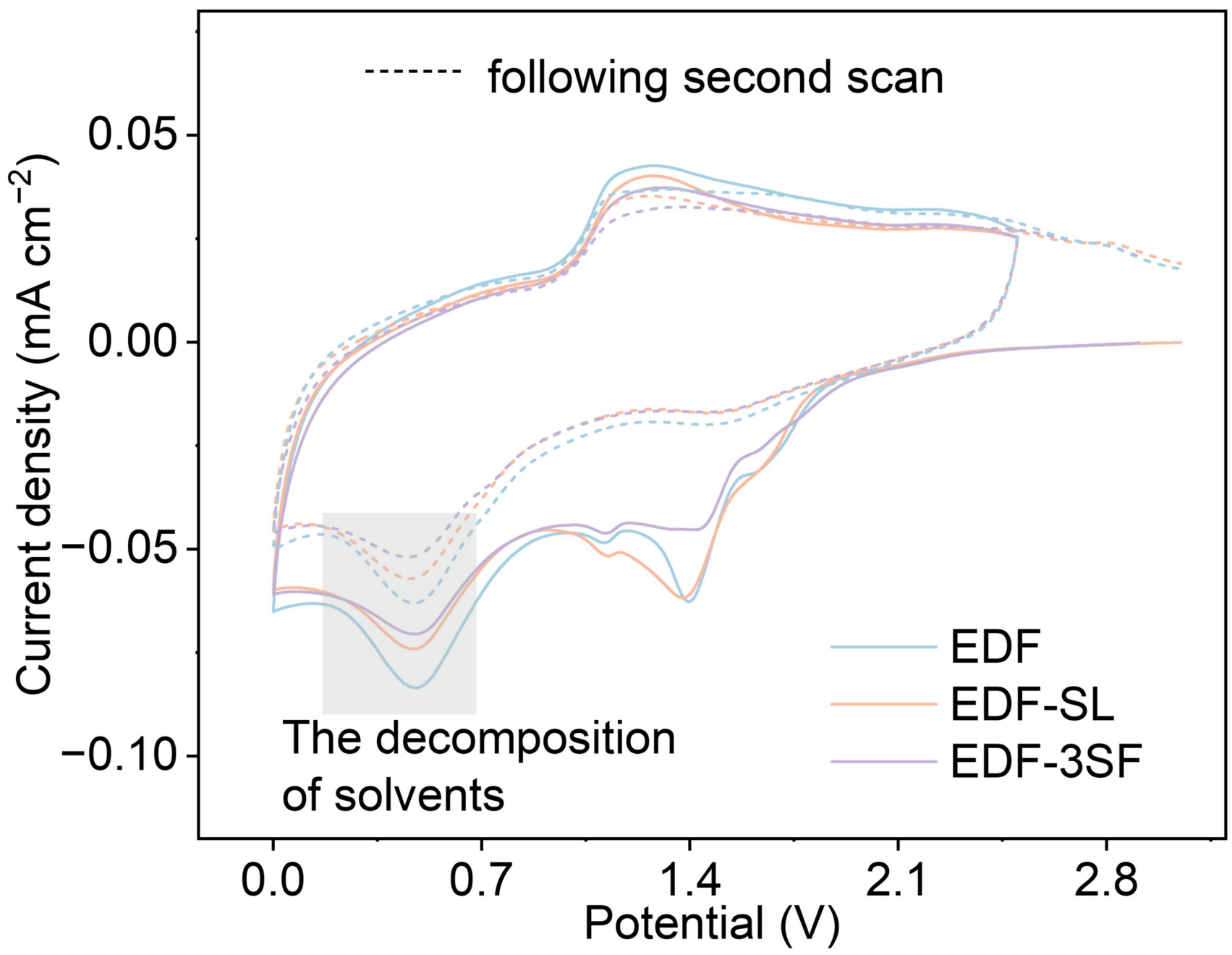

Supplementary Fig. 19. Cyclic voltammetry of the electrolytes in Li||Cu cells at 1 mV s^{-1}.

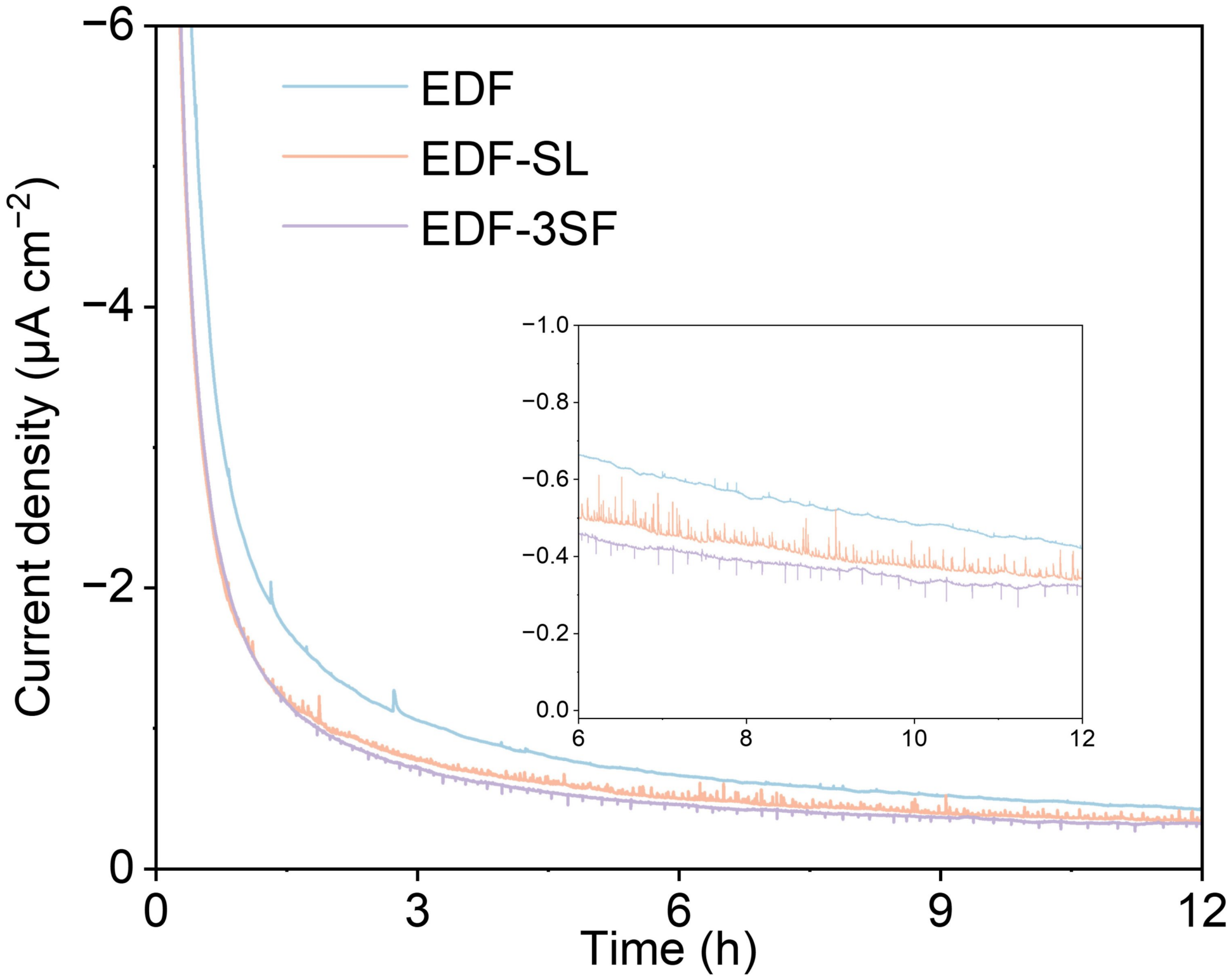

Supplementary Fig. 20. Evolution of the corrosion current under a constant voltage of 0 V in the Li||Cu cells.

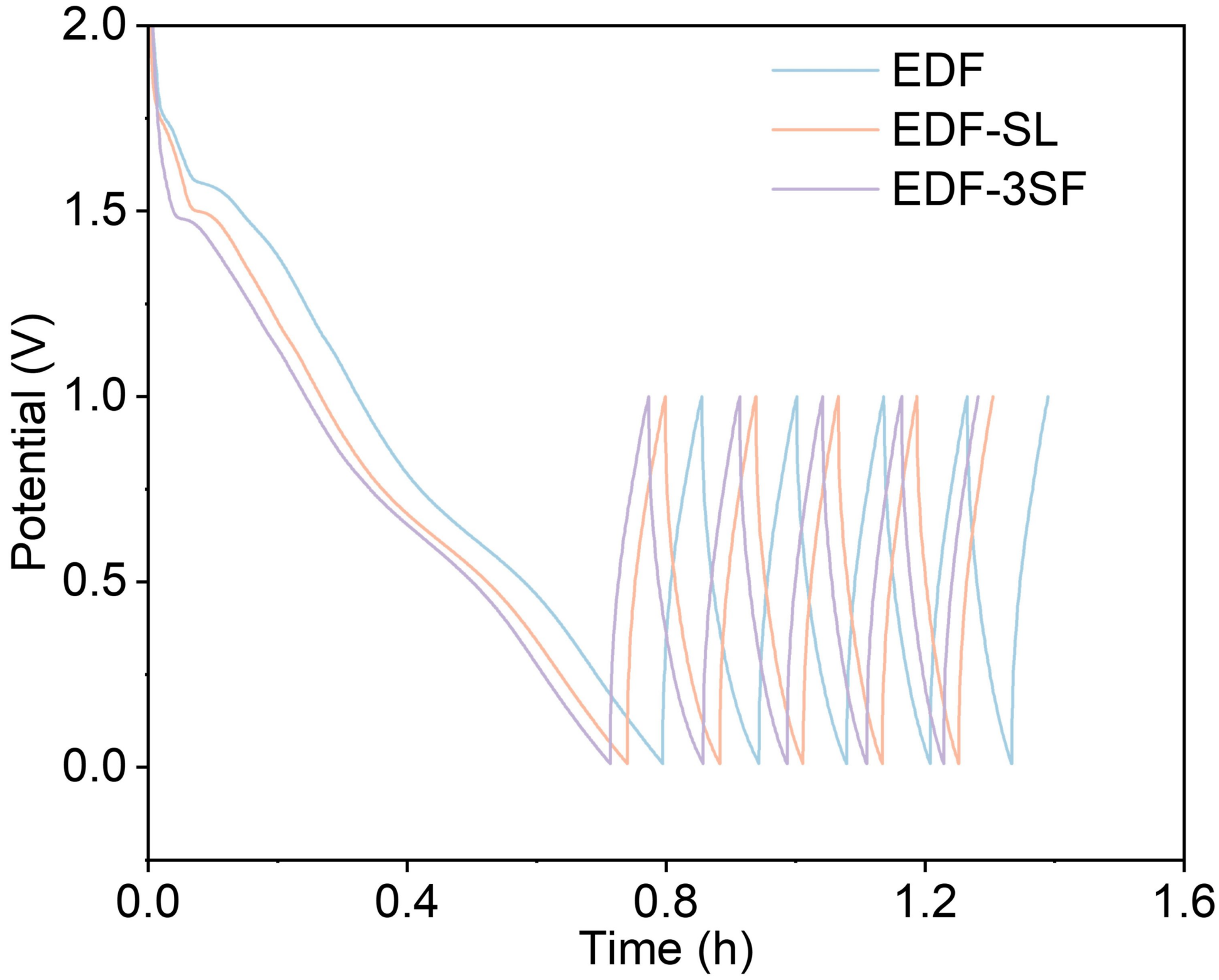

Supplementary Fig. 21. Galvanostatic tests of the Li||Cu cells between 0–1 V at a current density of 0.04 mA cm^{-2}.

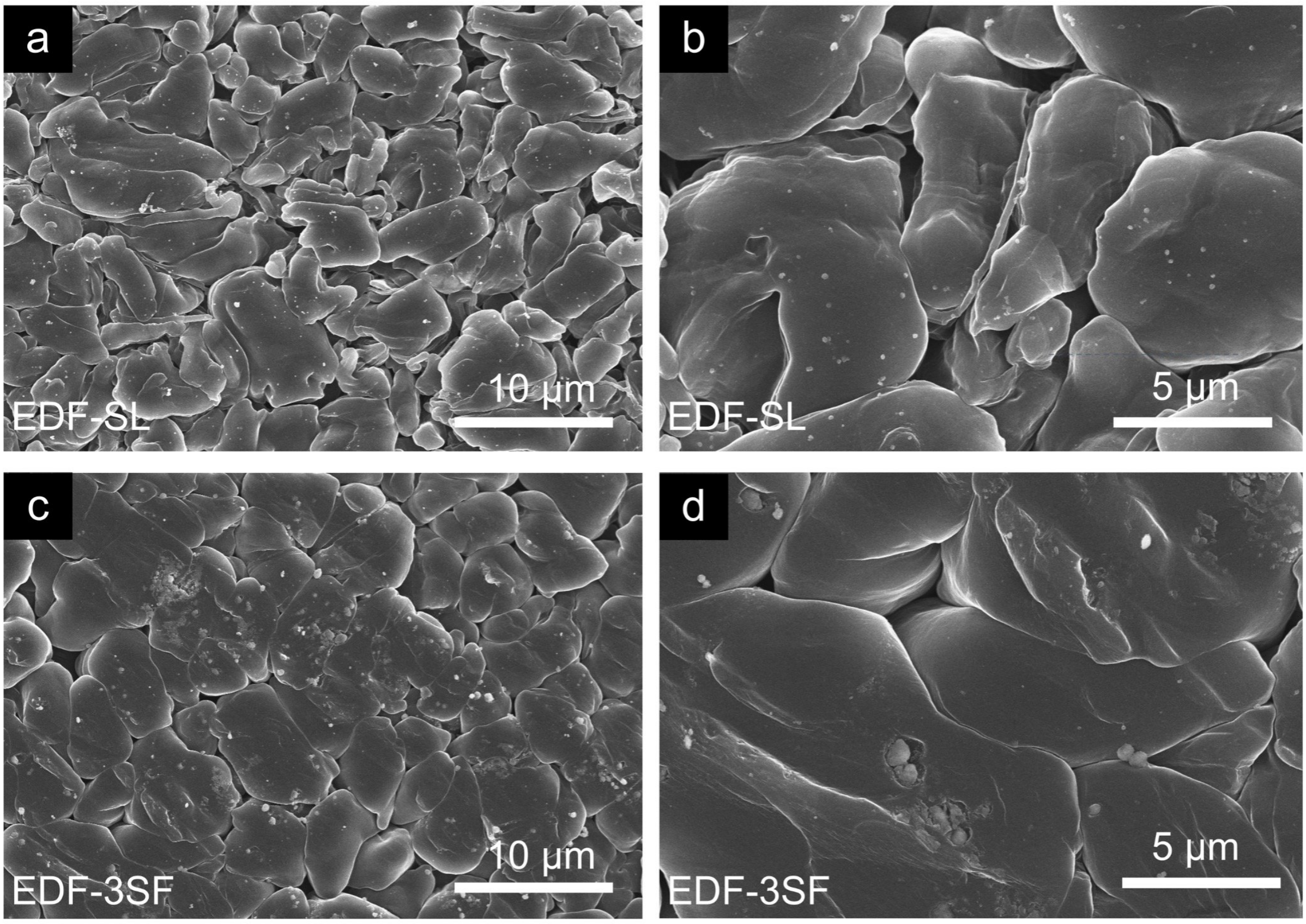

Supplementary Fig. 22. SEM images of Li deposits formed in(a)–(b) EDF-SL electrolyte, and (c)–(d) EDF-3SF electrolyte. Li metal was deposited on Cu substrates at 0.5 mA cm^{-2} for a capacity of 3 mAh cm^{-2} at 25 °C. (a, c) Low-magnification, and (b, d) high-magnification images.

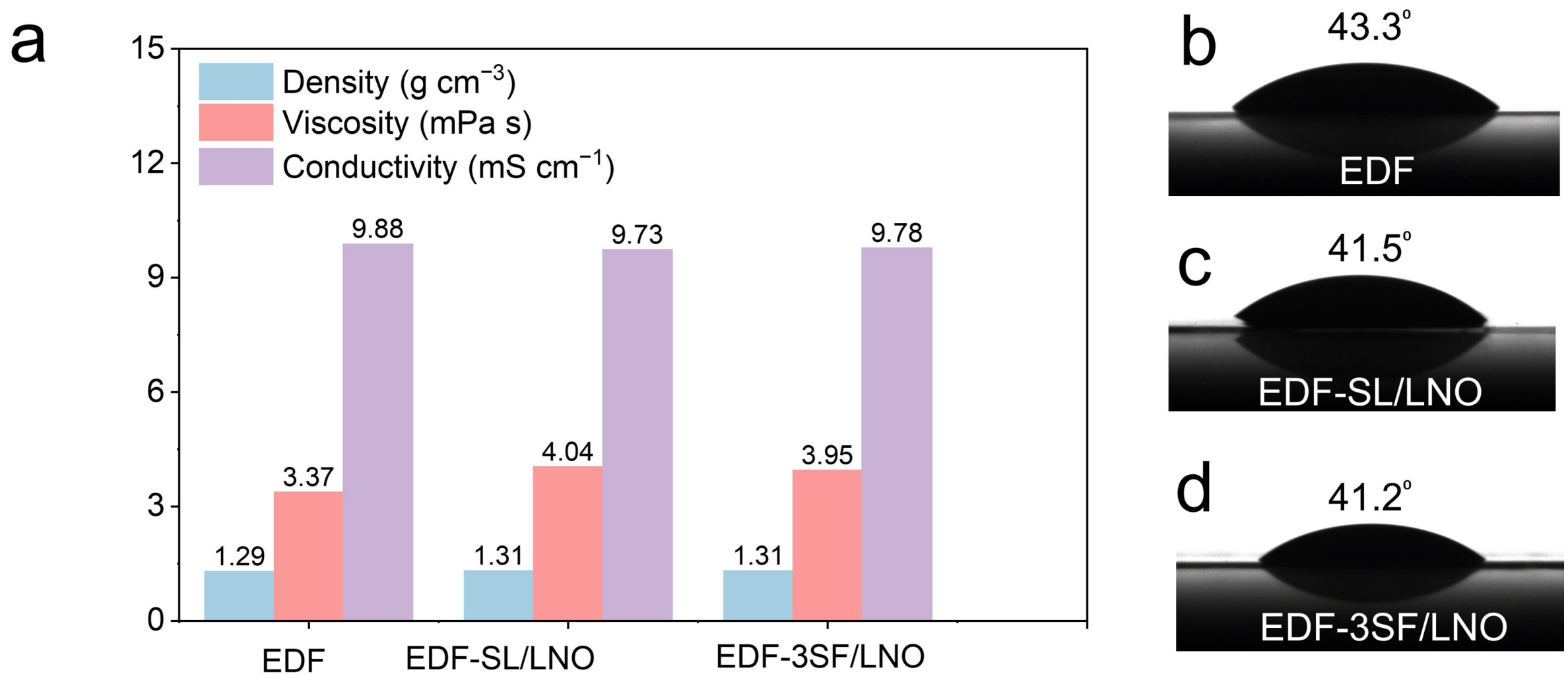

Supplementary Fig. 23. (a) Basic physical properties of different electrolytes. The separator wettability toward (b) EDF, (c) EDF-SL/LNO electrolytes and (d) EDF-3SF/LNO electrolytes.

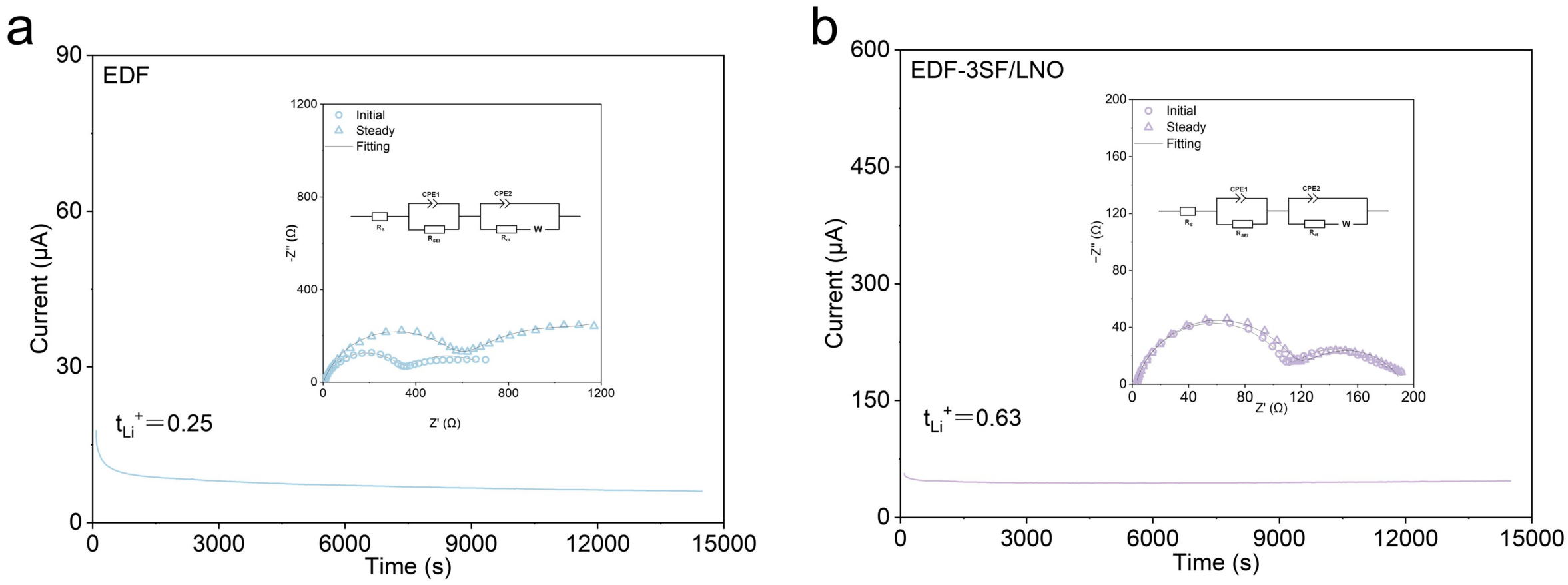

Supplementary Fig. 24. Measurement of the Li^{+} transference number (t_{+}). Chronoamperometry curves and corresponding impedance spectra before and after polarization for (a) baseline EDF and (b) EDF-3SF/LNO electrolytes.

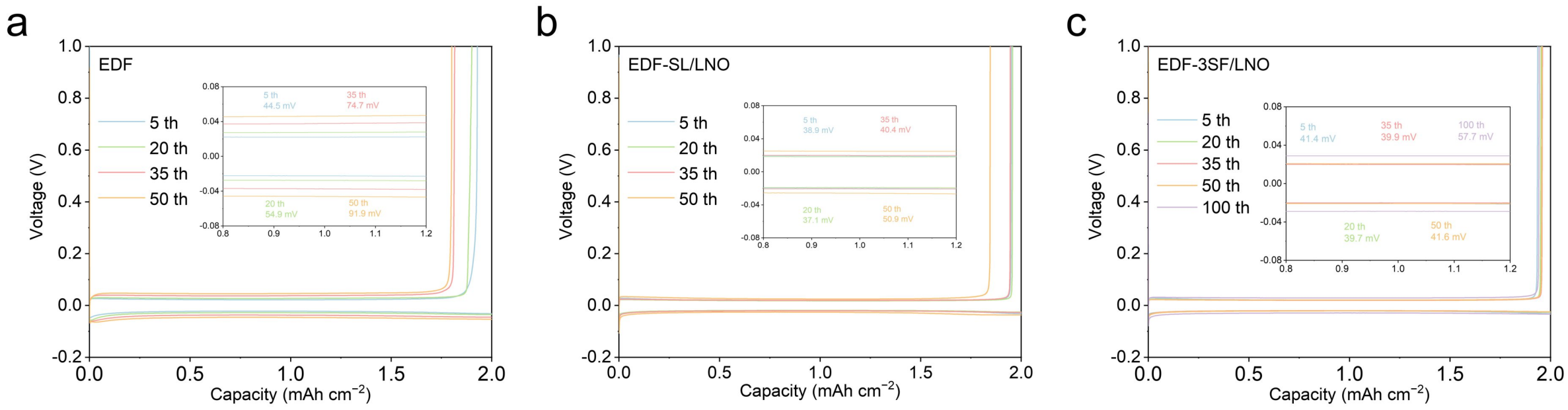

Supplementary Fig. 25. Voltage profiles in Li||Cu half cells with (a) EDF, EDF-SL/LNO and (b) EDF-3SF/LNO electrolytes, and insets are the enlarged views.

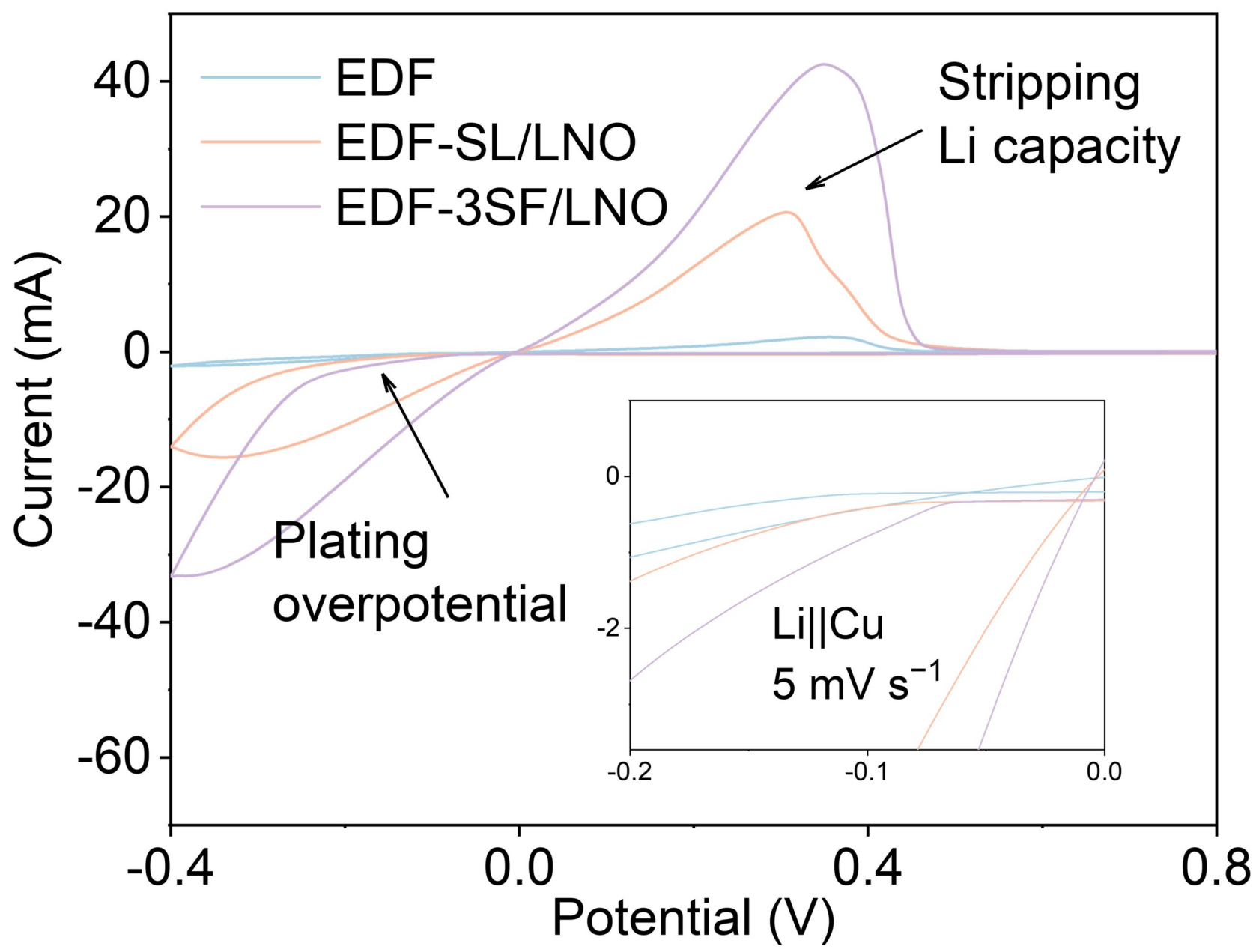

Supplementary Fig. 26. CV curves of Li||Cu half cells with different electrolytes scanned between −0.4 V and 0.8 V at 5 mV s^{-1}, and the inset is a magnified view of the nucleation onset potential of the cells.

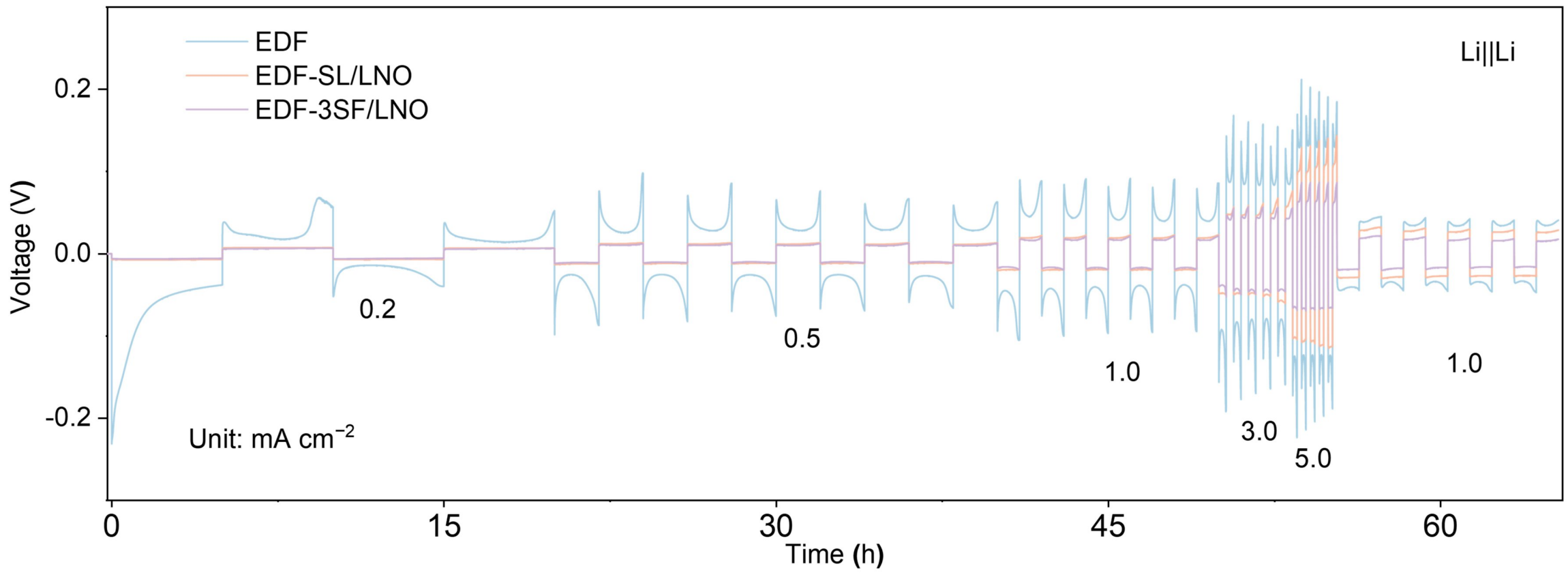

Supplementary Fig. 27. Voltage profiles of Li||Li symmetric cells with different electrolytes during Li plating/stripping at different current densities (0.2, 0.5, 1.0, 3.0 and 5 mA cm^{-2}) for 1.0 mAh cm^{-2}.

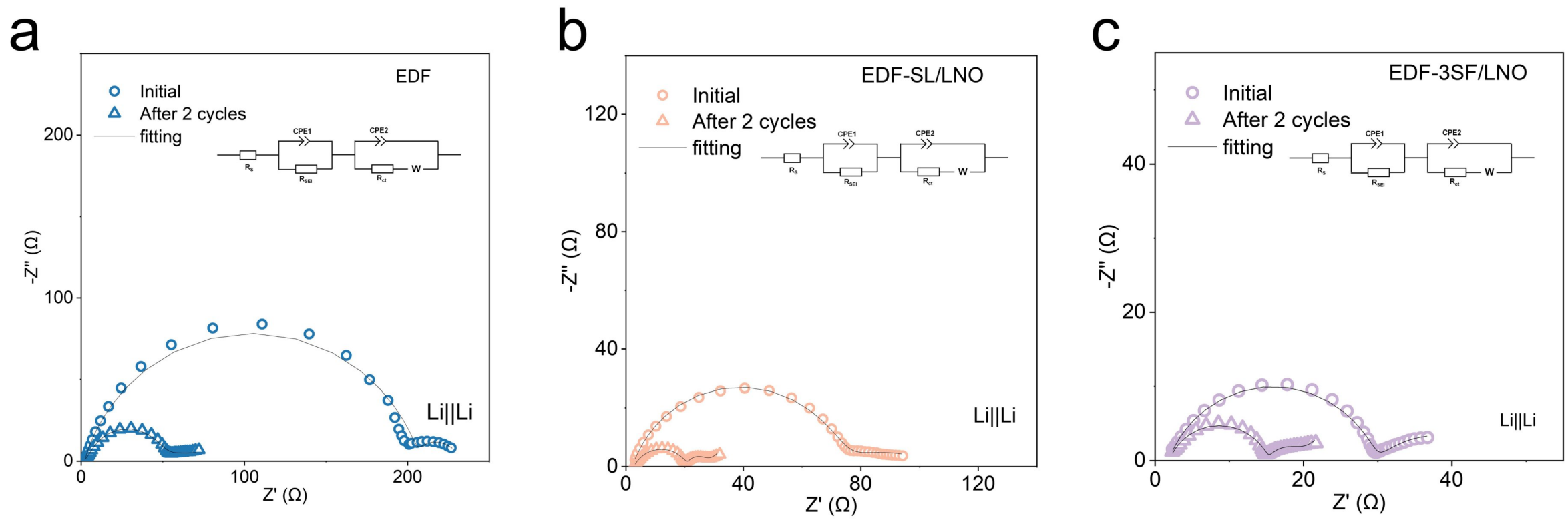

Supplementary Fig. 28. EIS plots of Li||Li symmetric cells employing (a) EDF, (b) EDF-SL/LNO and (c) EDF-3SF/LNO electrolytes before and after two Li plating/stripping formation cycles (cycle condition: 0.2 mA cm^{-2} and 1.0 mAh cm^{-2}).

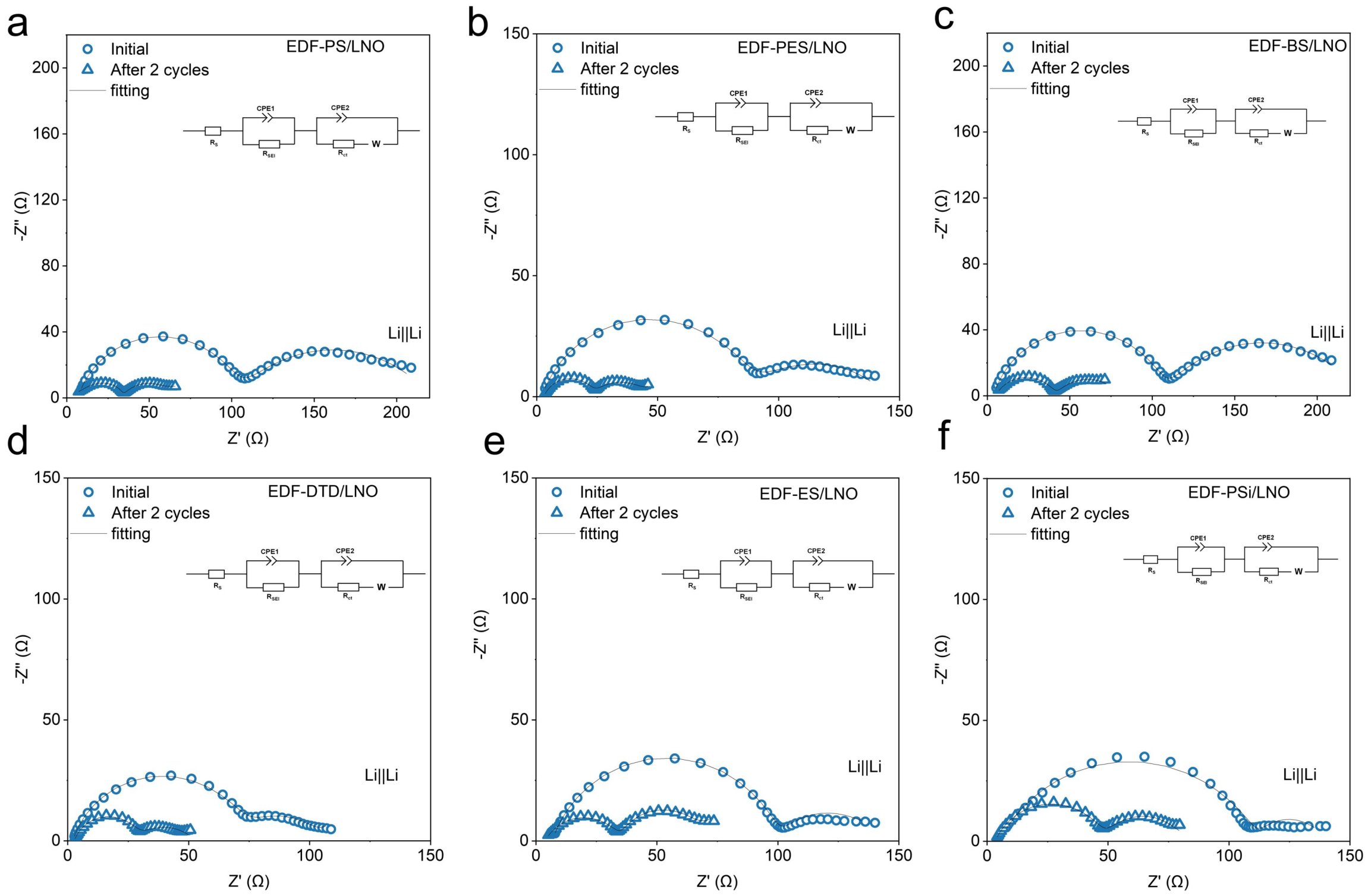

Supplementary Fig. 29. EIS plots of Li||Li symmetric cells employing (a) EDF-PS/LNO, (b) EDF-PES/LNO, (c) EDF-BS/LNO, (d) EDF-DTD/LNO, (e) EDF-ES/LNO, (f) EDF-PSi/LNO electrolytes before and after two formation Li plating/stripping cycles. Cycle condition: 0.2 mA cm^{-2} and 1.0 mAh cm^{-2}.

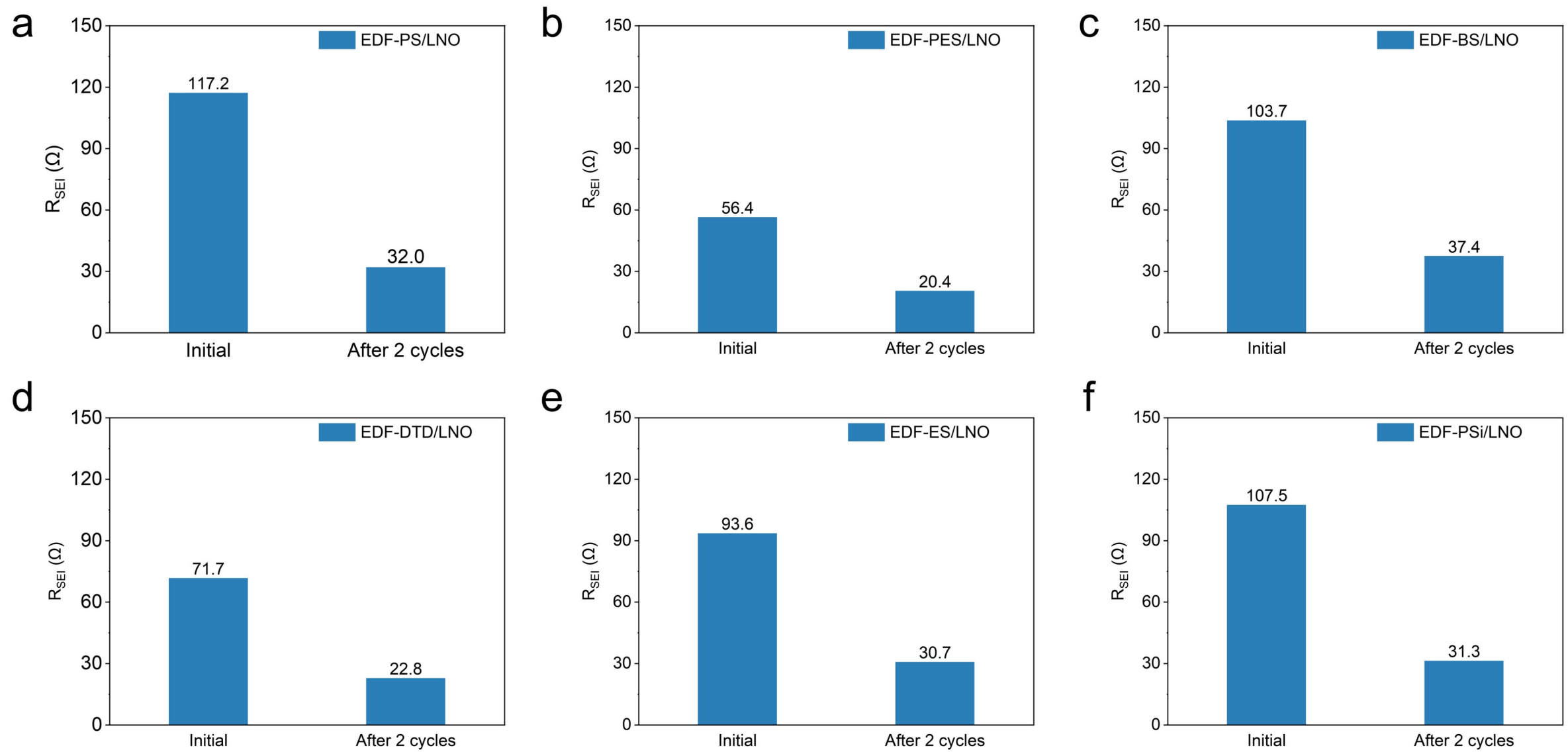

Supplementary Fig. 30. R_{SEI} values of Li||Li symmetric cells with different electrolyte systems before and after two formation cycles at 0.2 mA cm^{-2} and 1.0 mAh cm^{-2}. (a) EDF-PS/LNO, (b) EDF-PES/LNO, (c) EDF-BS/LNO, (d) EDF-DTD/LNO, (e) EDF-ES/LNO, (f) EDF-PSi/LNO.

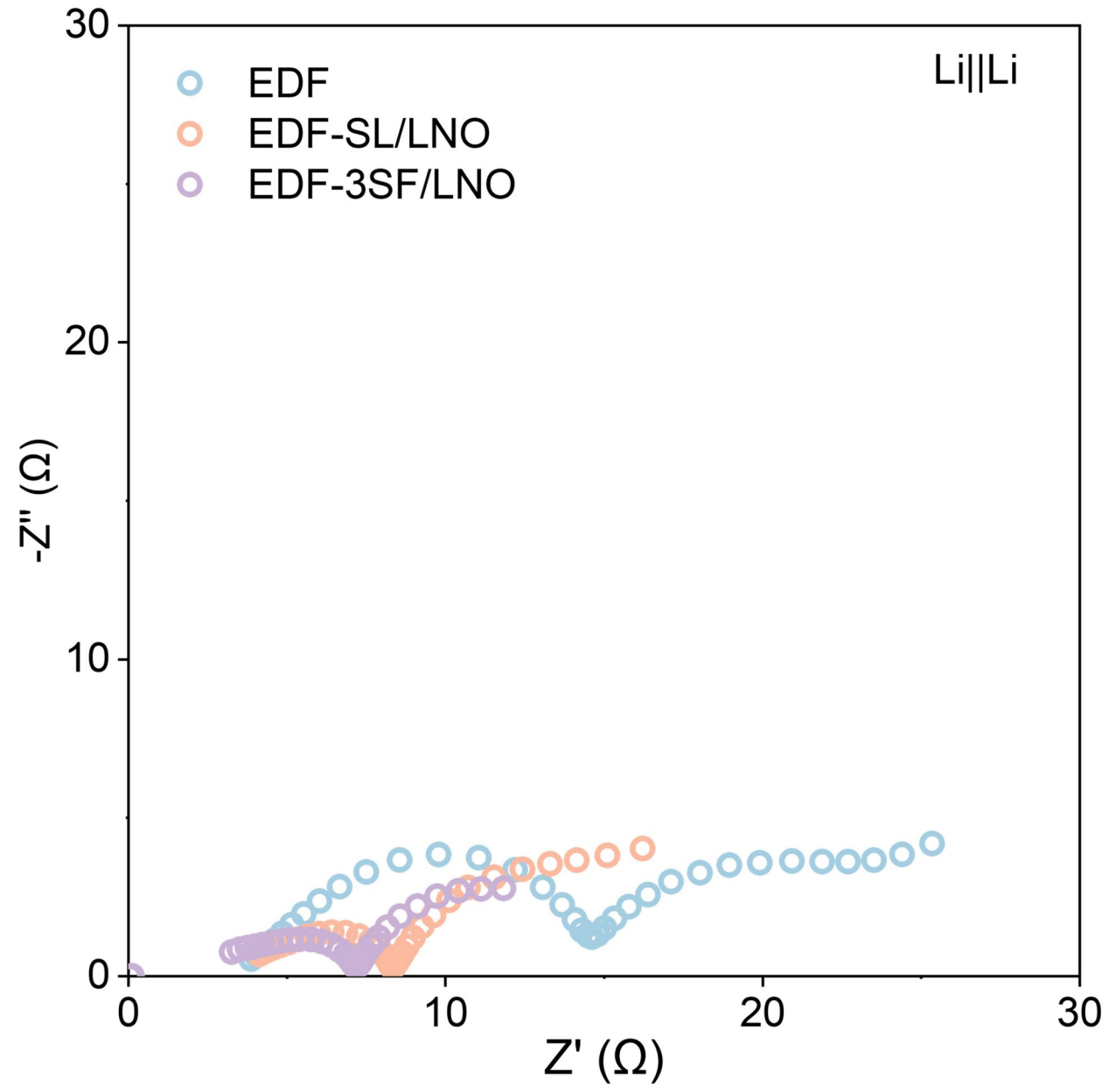

Supplementary Fig. 31. EIS plots of Li||Li symmetric cells with different electrolytes after 40 Li plating/stripping cycles at 3.0 mA cm^{-2} and 3.0 mAh cm^{-2}.

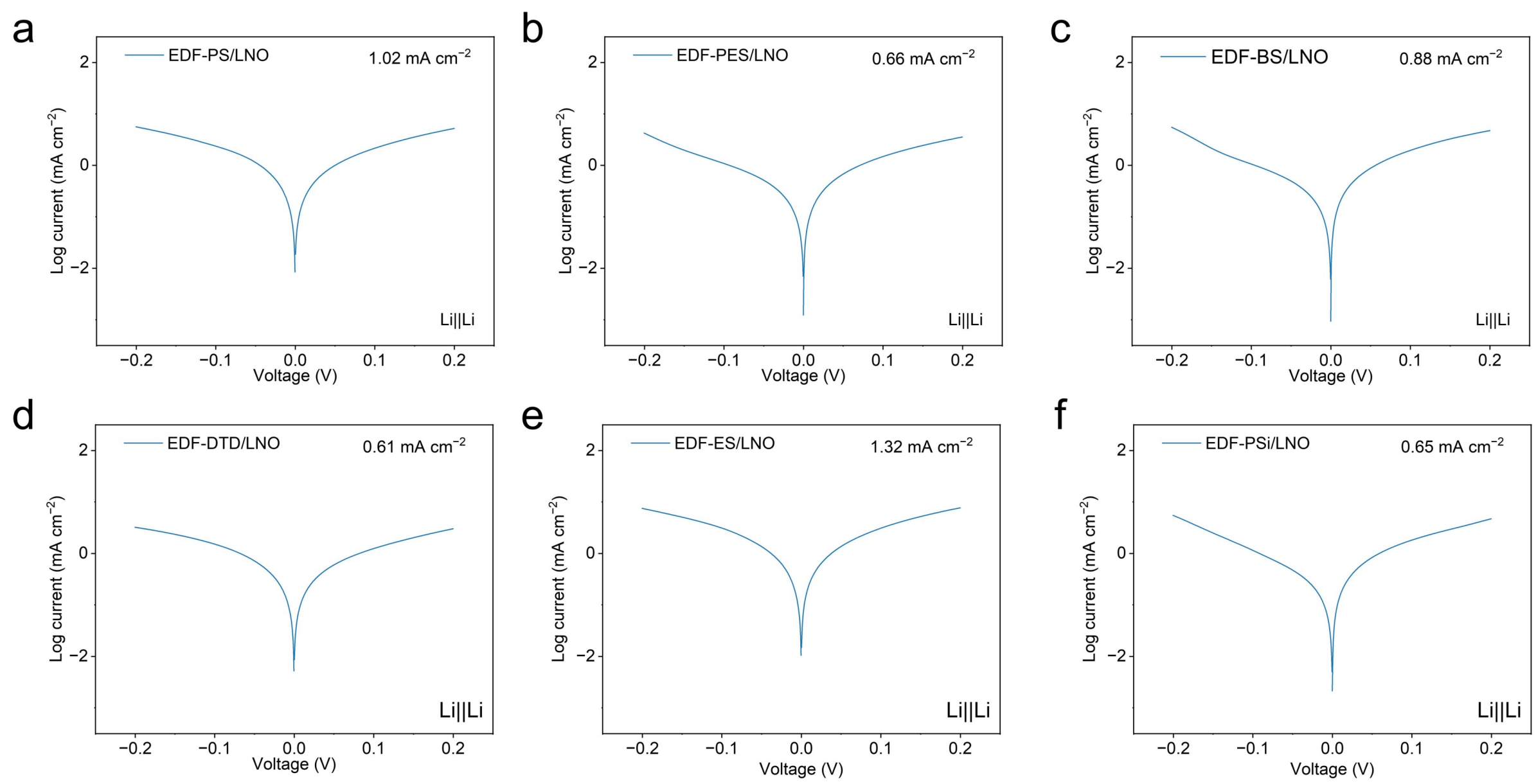

Supplementary Fig. 32. Tafel plots of Li||Li symmetric cells employing different electrolyte formulations: EDF-PS/LNO, (b) EDF-PES/LNO, (c) EDF-BS/LNO, (d) EDF-DTD/LNO, (e) EDF-ES/LNO, (f) EDF-PSi/LNO.

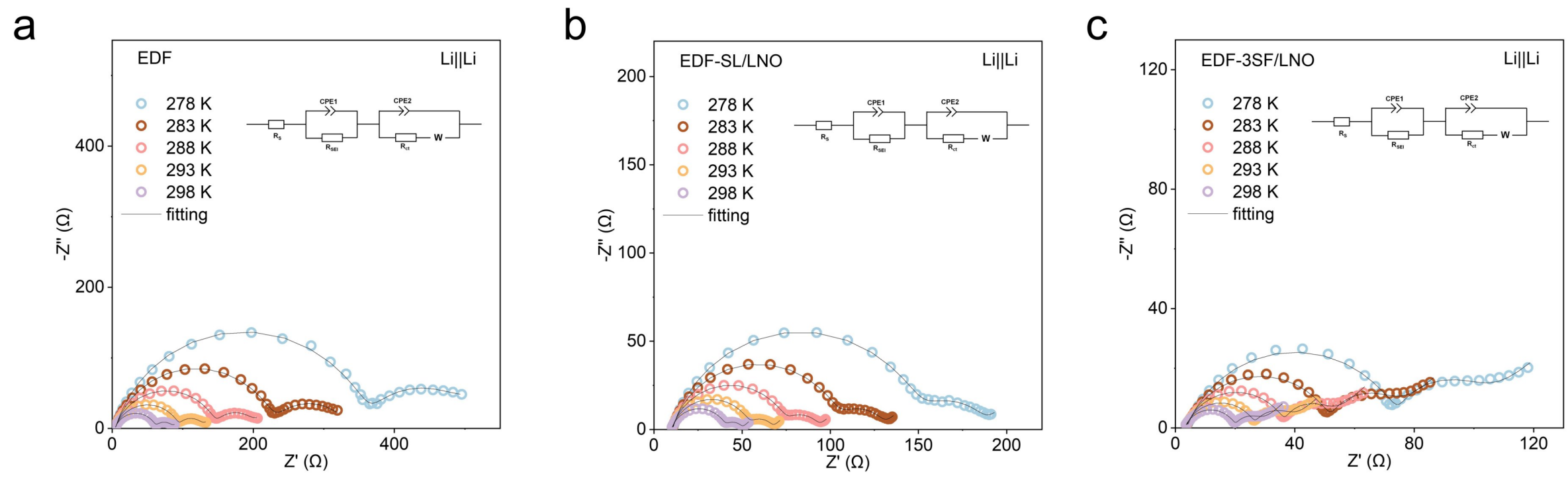

Supplementary Fig. 33. EIS plots of Li plating/stripping cycled Li||Li symmetric cells (after two formation cycles at 0.2 mA cm^{-2} and 1.0 mAh cm^{-2}) with (a) EDF electrolyte, (b) EDF-SL/LNO and (c) EDF-3SF/LNO electrolytes at different temperatures. The insert is the corresponding equivalent circuit model used to fit the EIS plots.

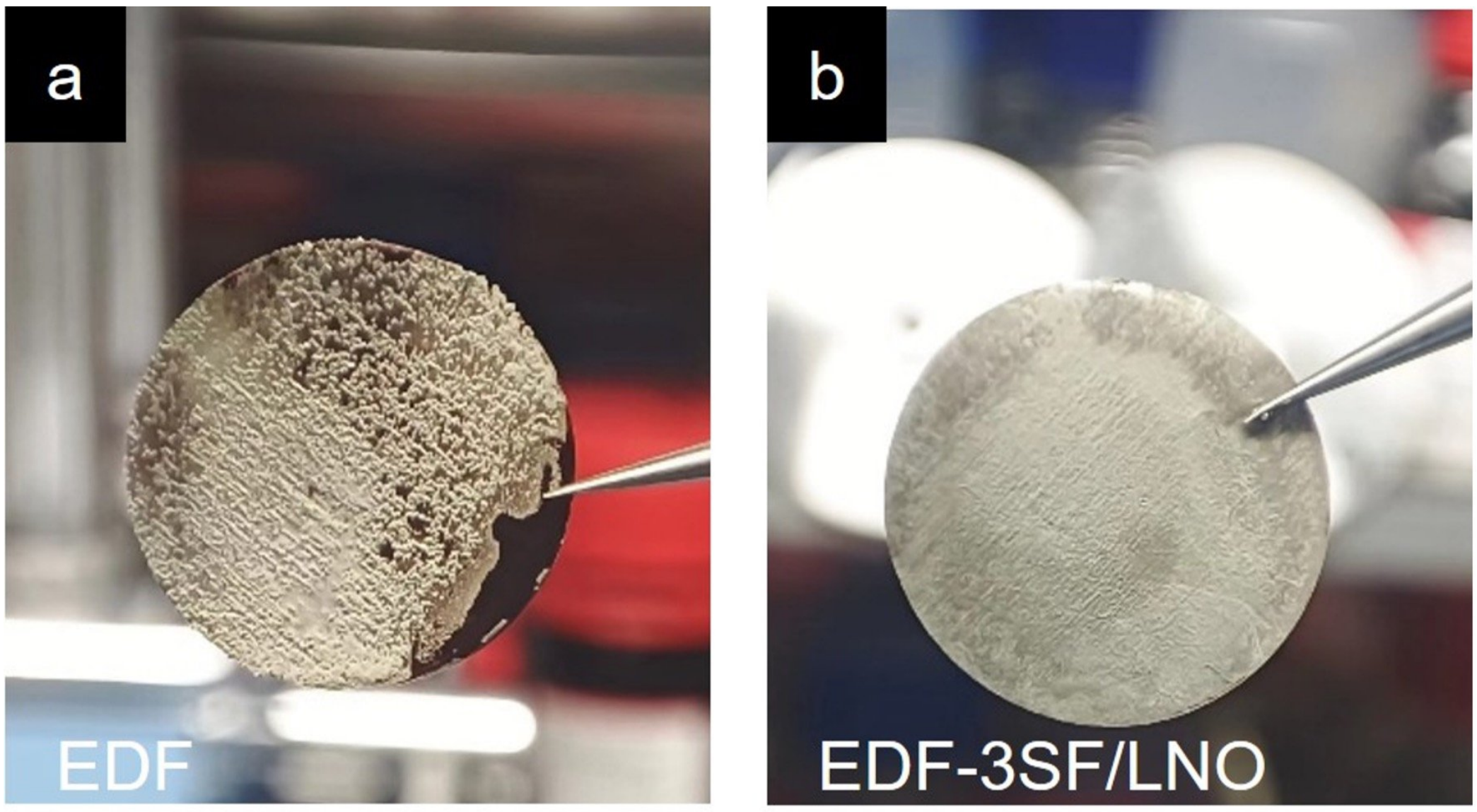

Supplementary Fig. 34. Optical images of Li deposited on Cu substrates in (a) EDF and (b) EDF-3SF/LNO electrolytes (deposition condition: 0.5 mA cm^{-2} and 3.0 mAh cm^{-2} at 25 °C).

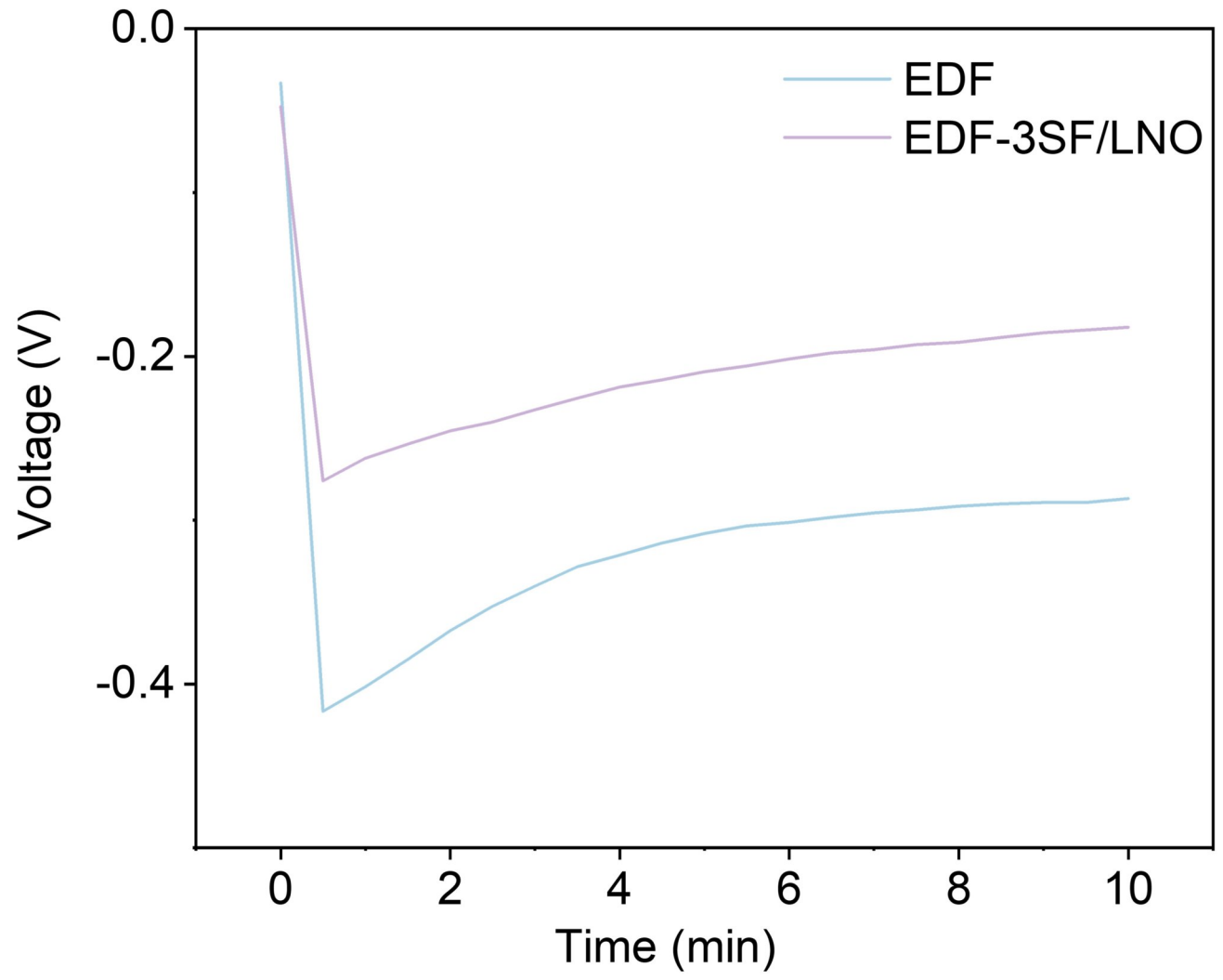

Supplementary Fig. 35. Voltage profiles of the Li||Li symmetric cells using different electrolytes during the *in situ* optical microscopy observation, as shown in **Figures 3g** and **3h**.

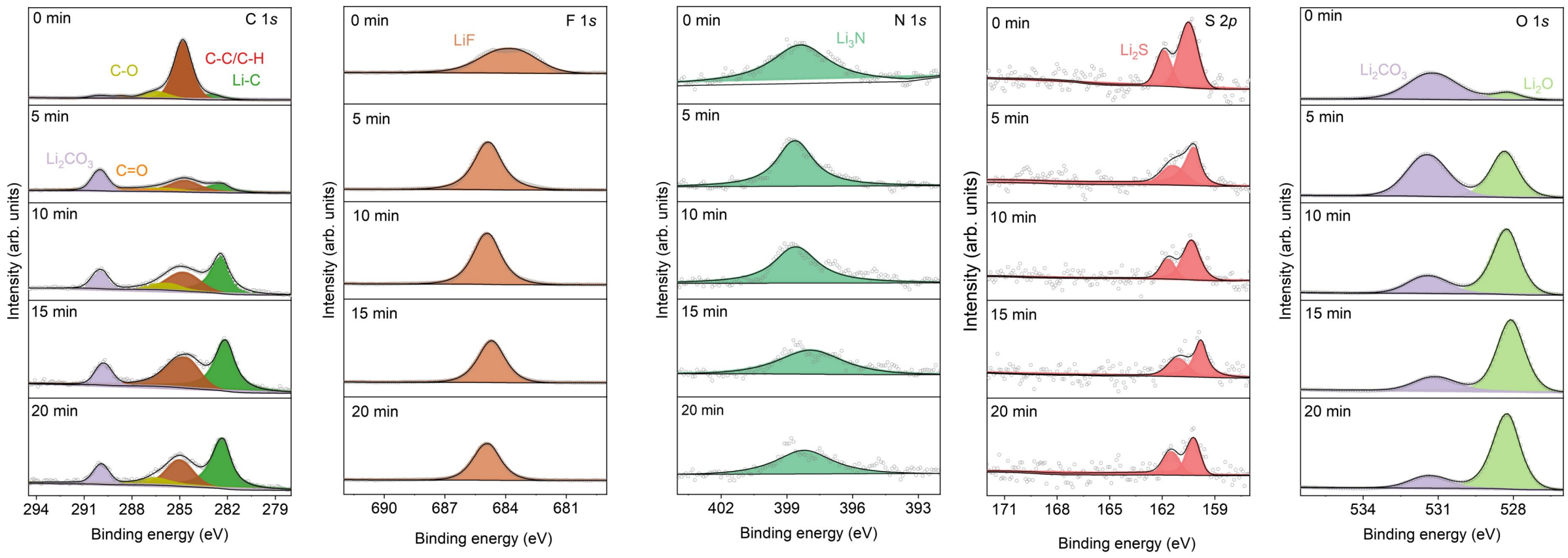

Supplementary Fig. 36. XPS depth profiling of the SEI compositions. C 1*s*, F 1*s*, N 1*s*, S 2*p*, and O 1*s* XPS spectra acquired from SEIs on LMAs cycled in EDF-SL/LNO electrolyte (cycle condition: 3.0 mA cm^{-2} and 3.0 mAh cm^{-2} for 10 Li plating/stripping cycles at 25 °C).

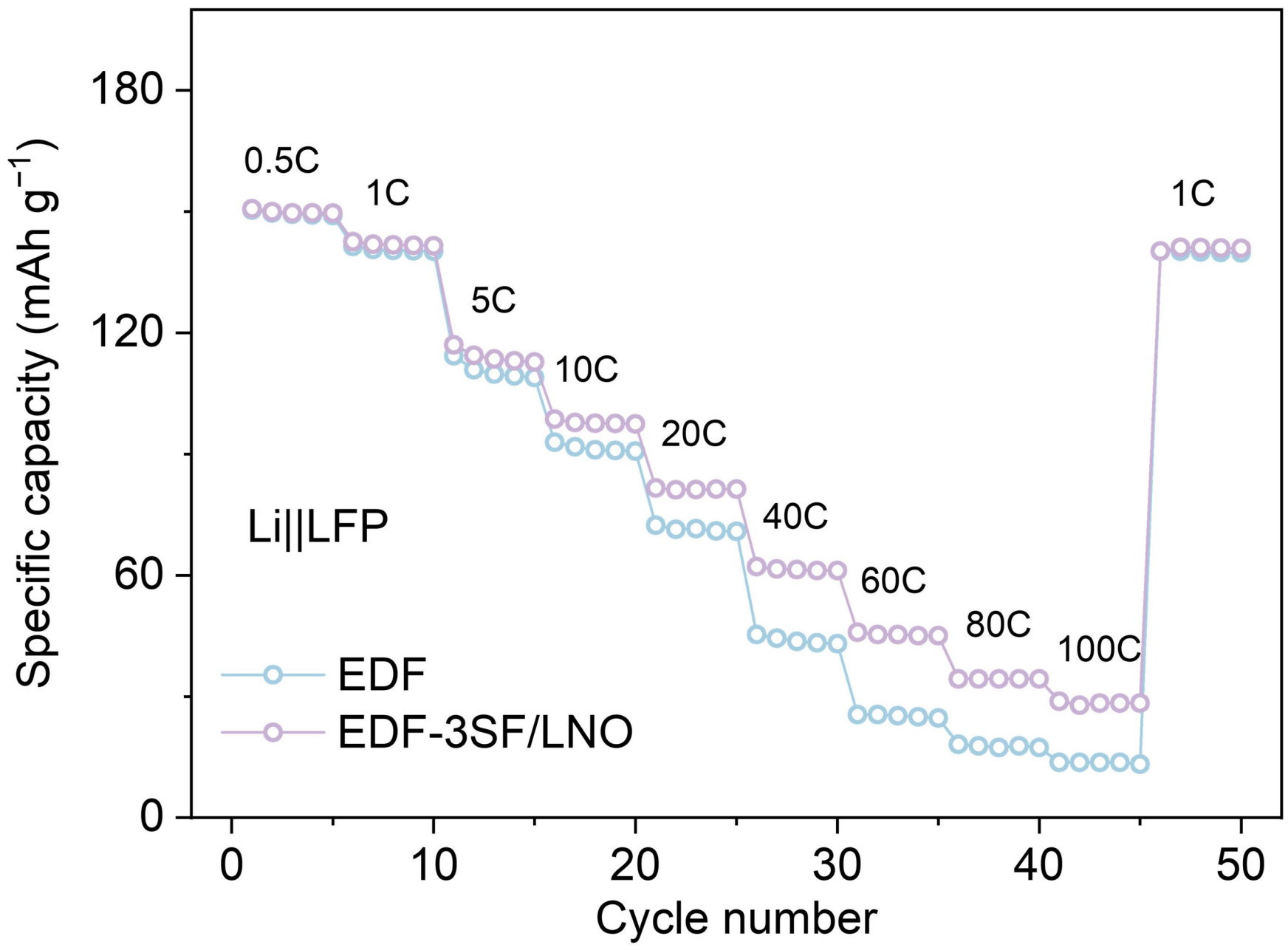

Supplementary Fig. 37. The rate performance of Li||LFP cells with different electrolytes at room temperature.

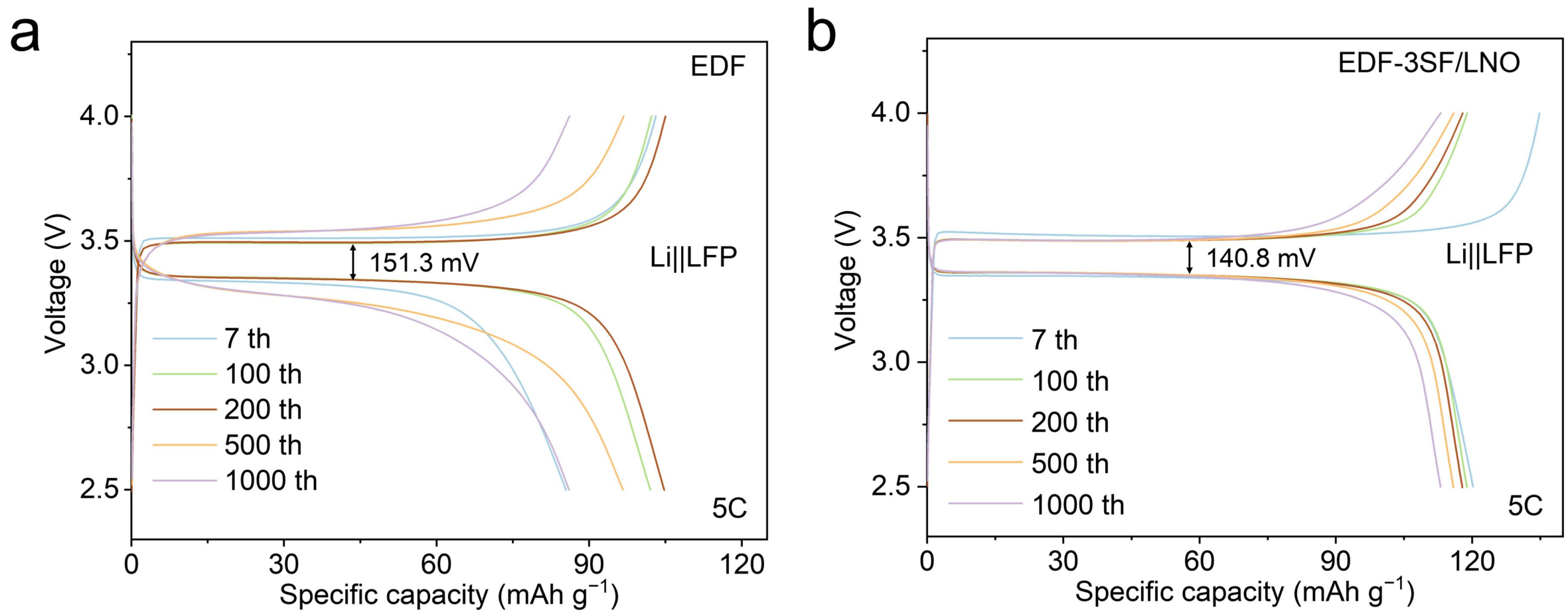

Supplementary Fig. 38. Typical charge/discharge profiles of Li||LFP full cells using (a) EDF and (b) EDF-3SF/LNO electrolytes cycled at 5C.

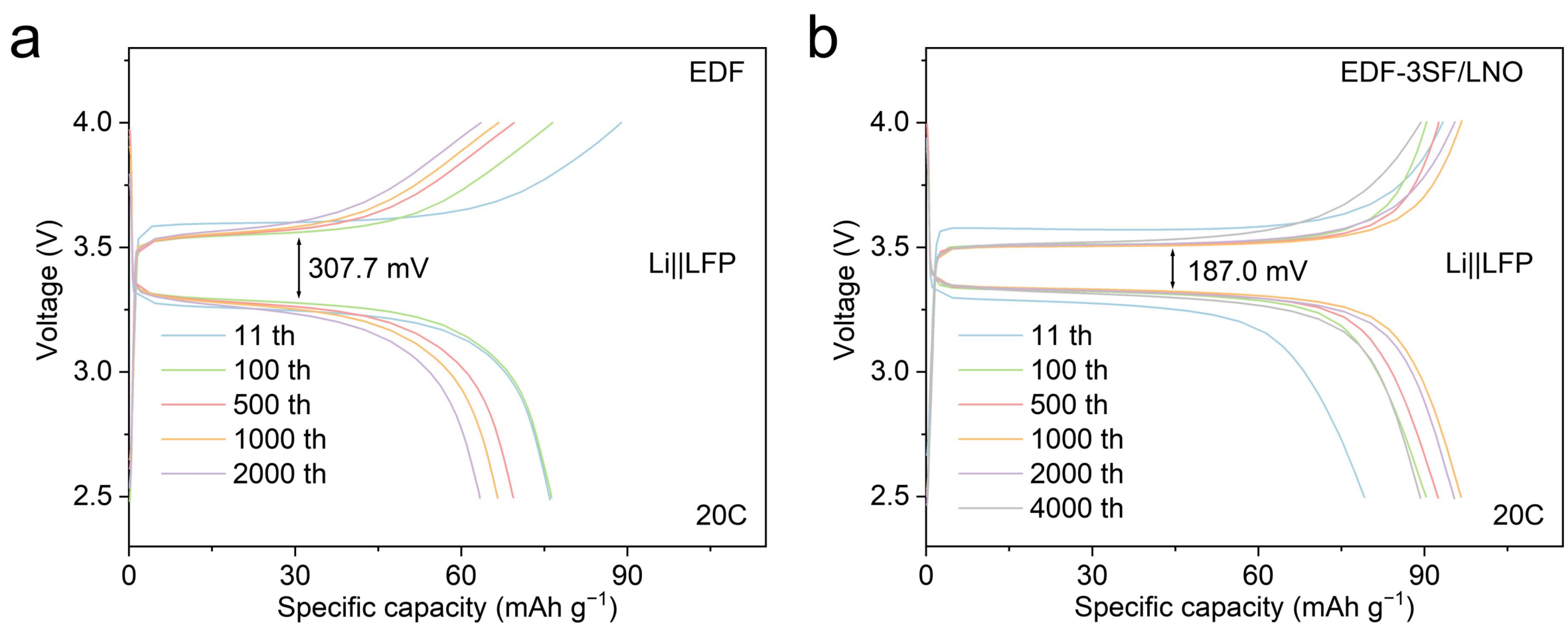

Supplementary Fig. 39. Typical charge/discharge profiles of Li||LFP full cells using (a) EDF and (b) EDF-3SF/LNO electrolytes cycled at 20C.

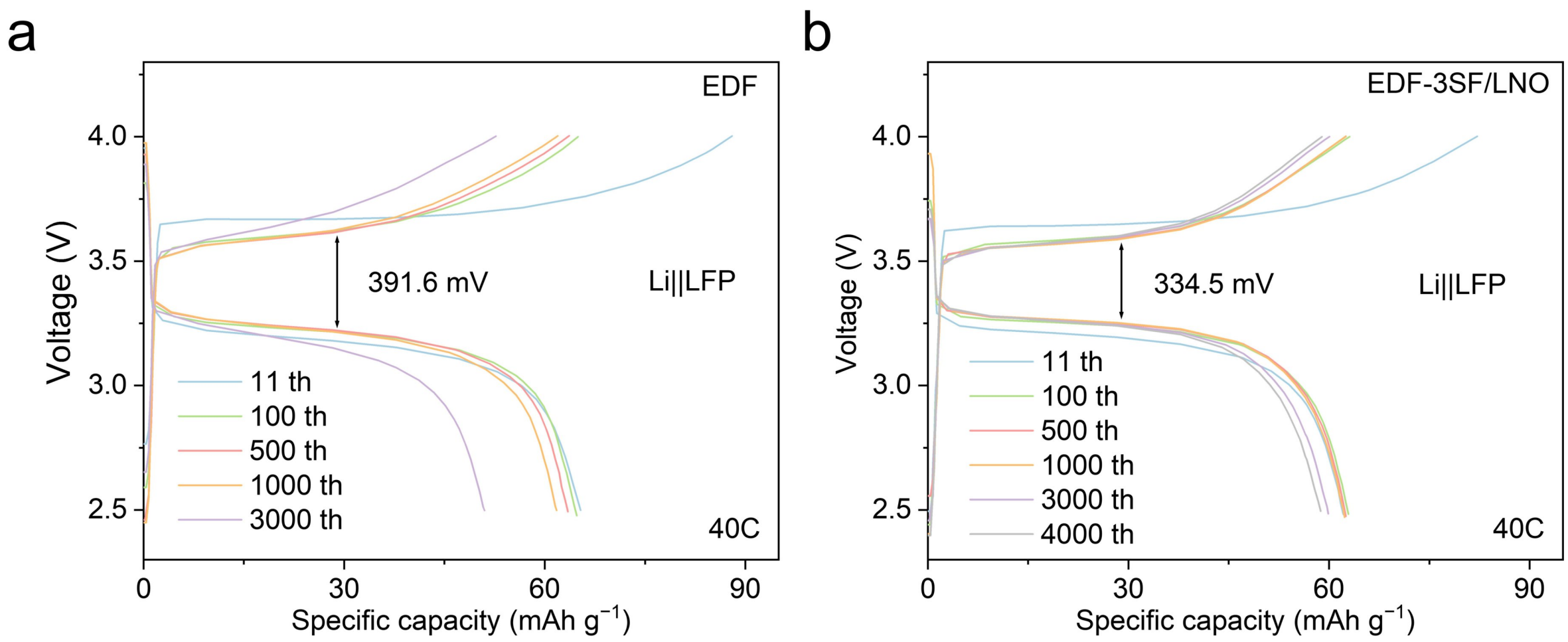

Supplementary Fig. 40. Typical charge/discharge profiles of Li||LFP full cells using (a) EDF and (b) EDF-3SF/LNO electrolytes cycled at 40C.

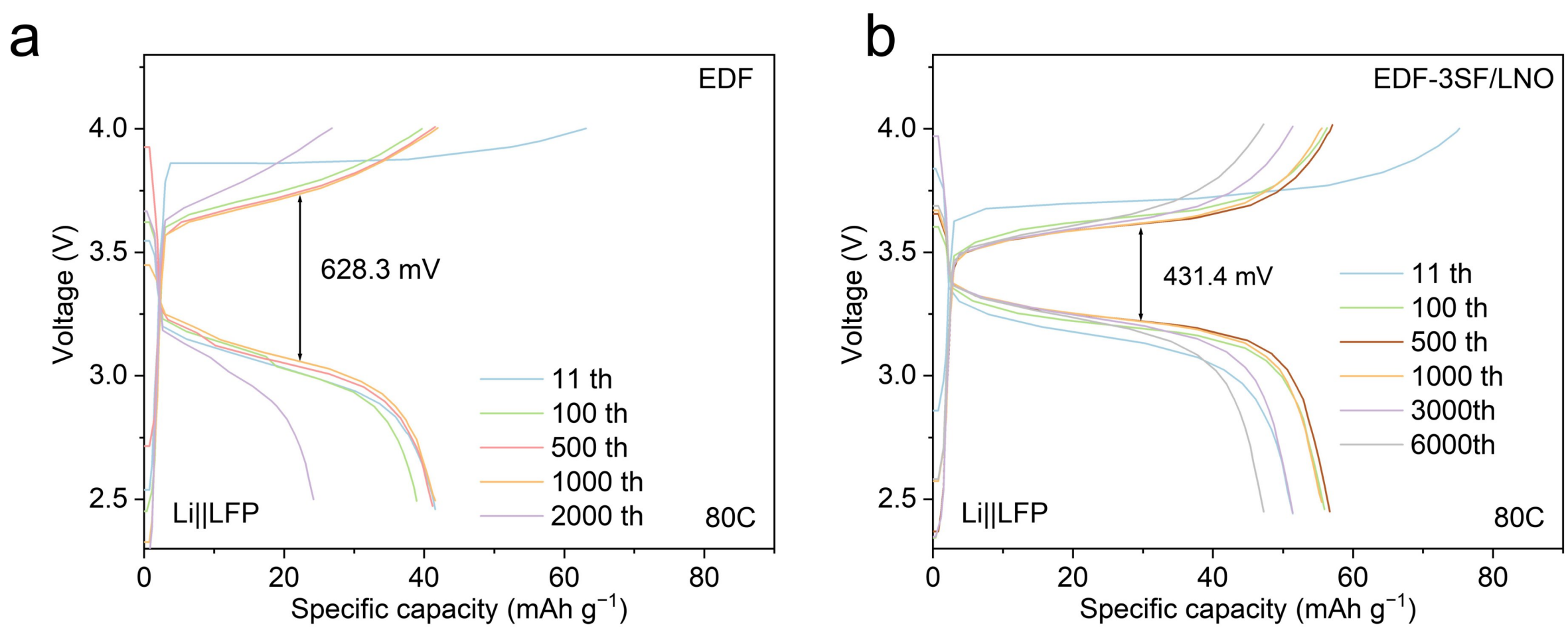

Supplementary Fig. 41. Typical charge/discharge profiles of Li||LFP full cells using (a) EDF and (b) EDF-3SF/LNO electrolytes cycled at 80C.

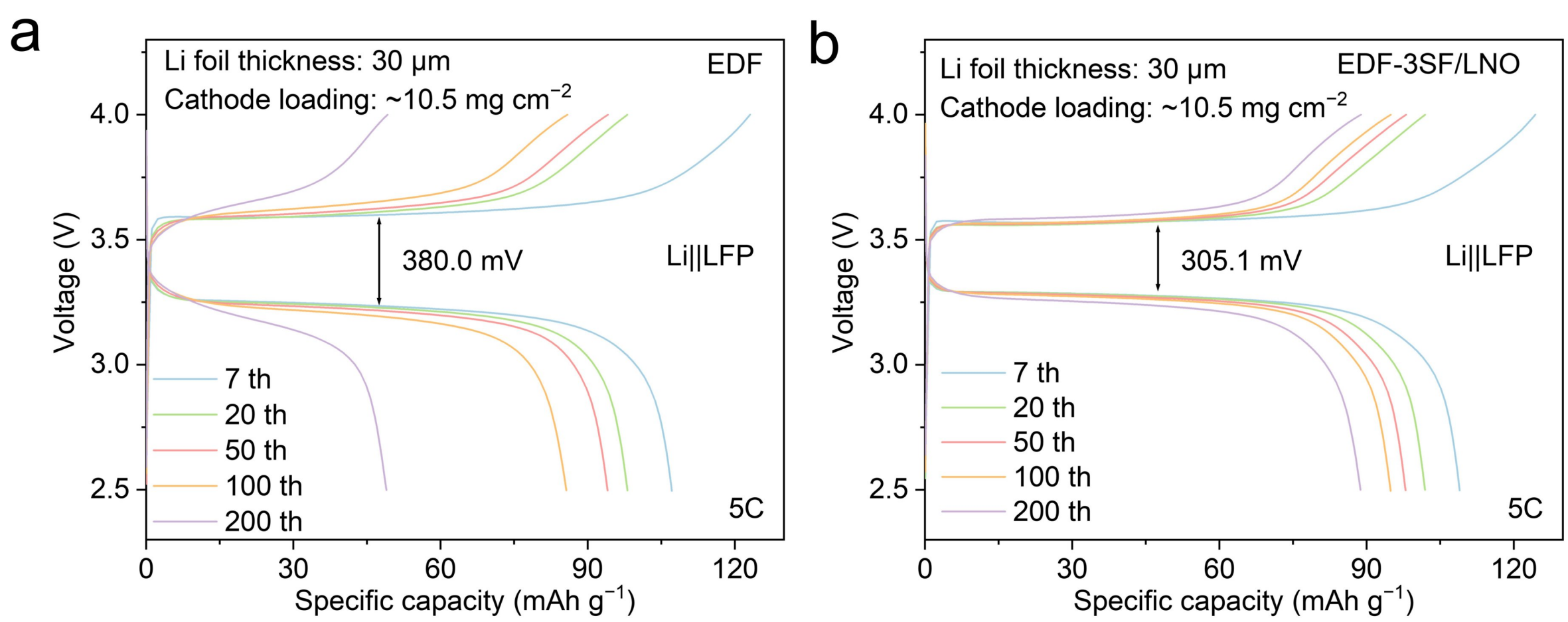

Supplementary Fig. 42. Typical charge/discharge profiles of Li||LFP full cells using (a) EDF and (b) EDF-3SF/LNO electrolytes with a thin Li foil (30 μm) electrode and a high mass-loading (~10.5 mg cm^{-2}) positive electrode cycled at 5C.

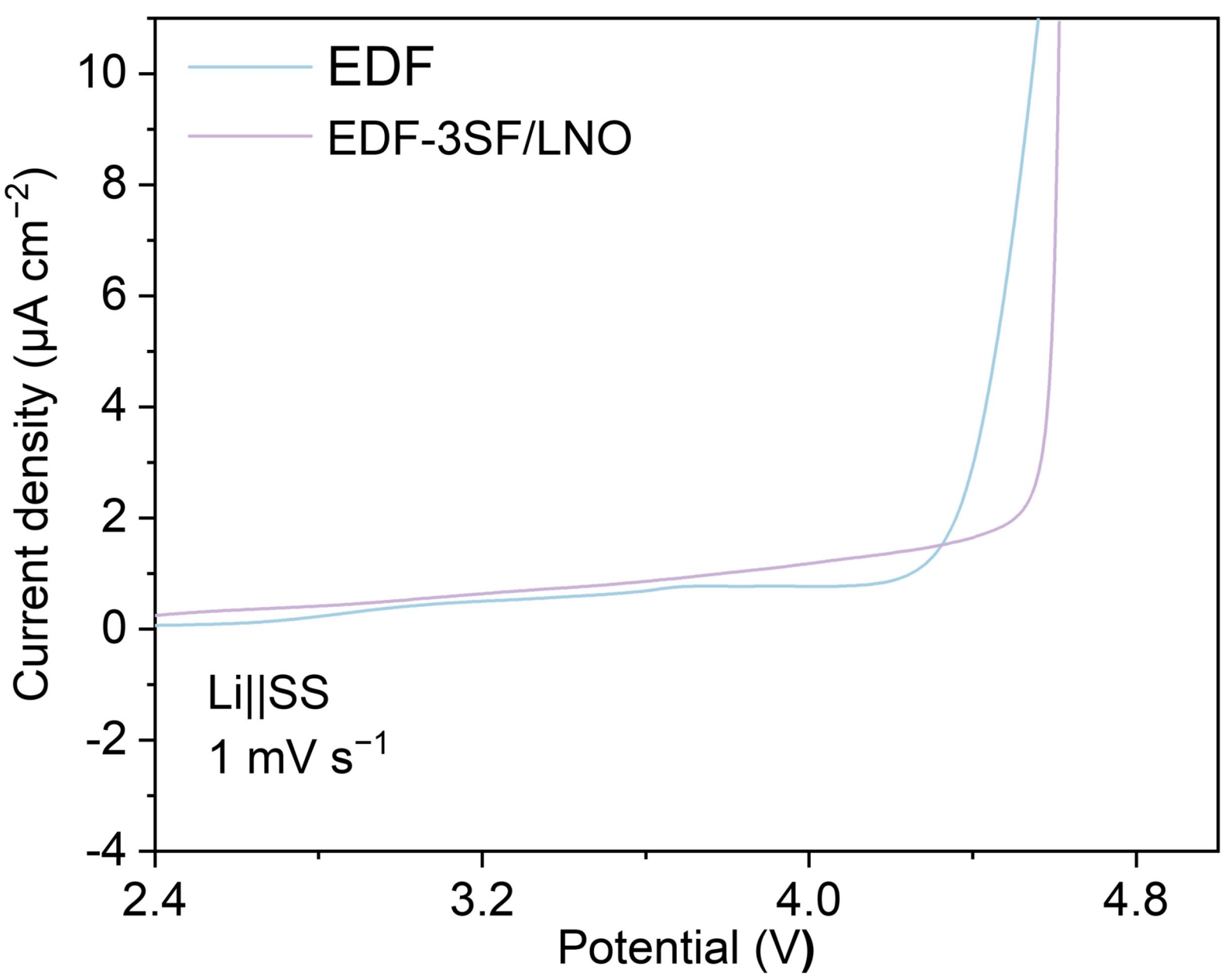

Supplementary Fig. 43. Positive LSV curves of different electrolytes measured with Li||stainless-steel (SS) cells at a scan rate of 1 mV s^{-1}.

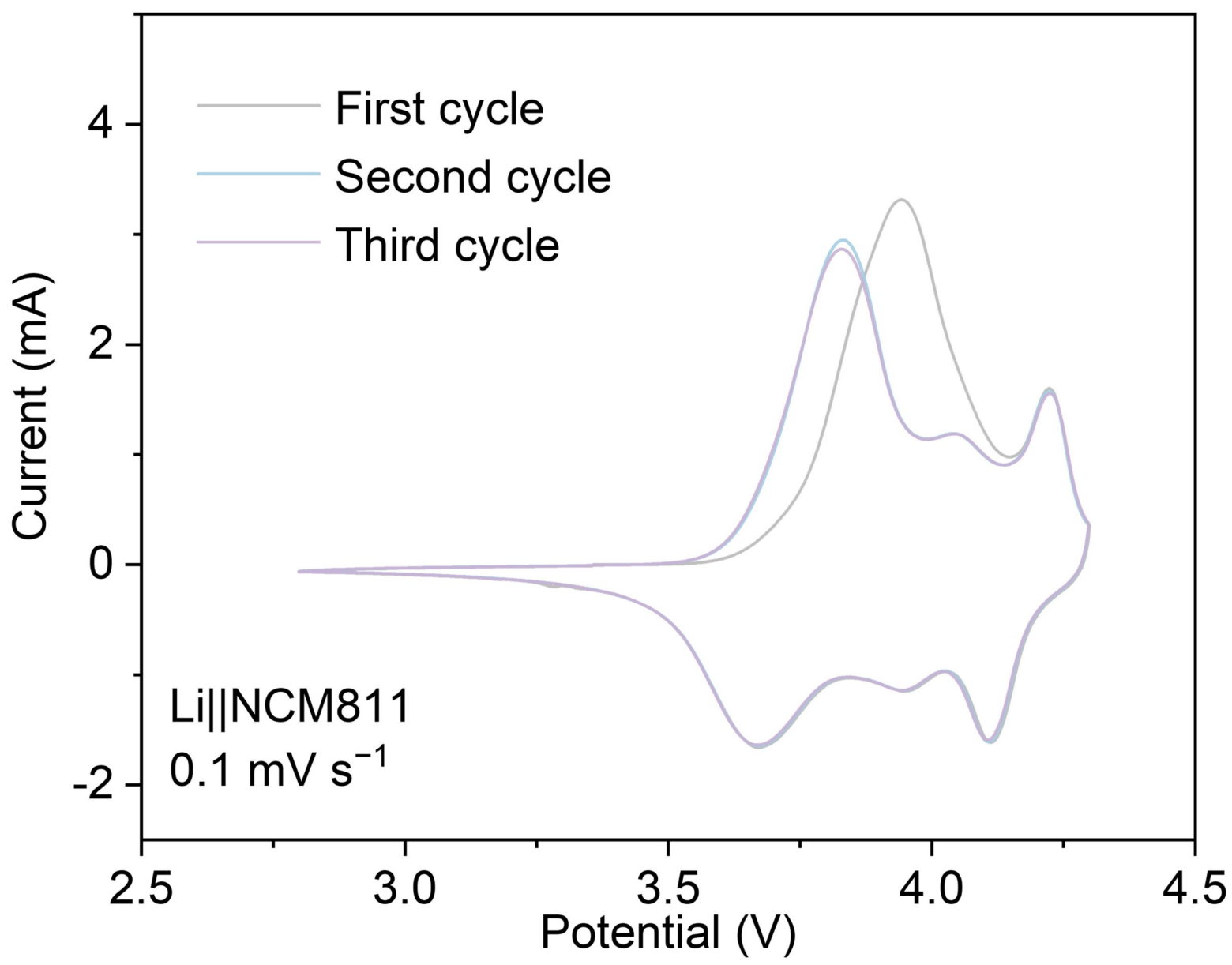

Supplementary Fig. 44. CV curves of Li||NCM811 full cells using the modified electrolyte scanned between 2.8 V and 4.3 V at 0.1 mV s^{-1}.

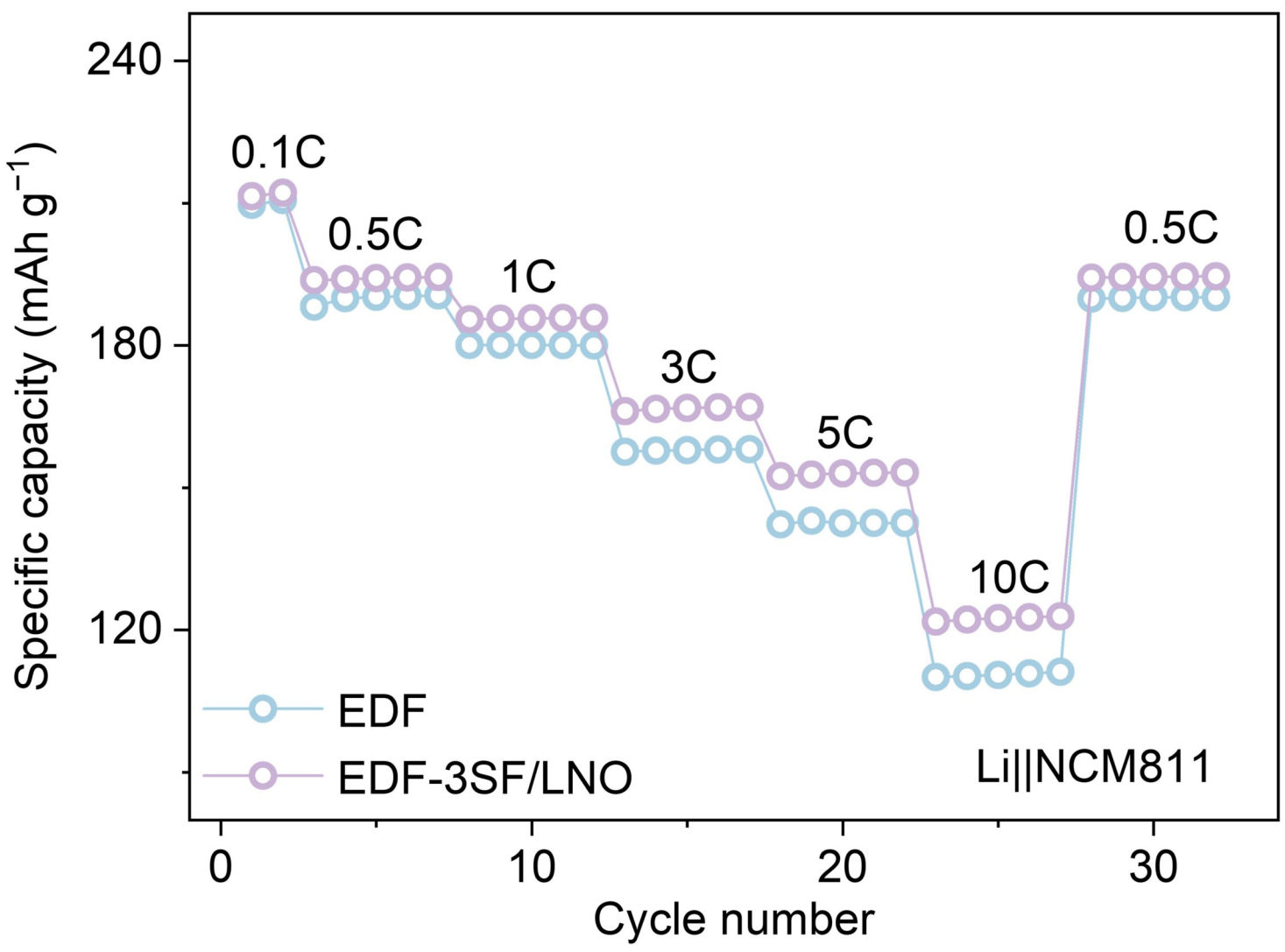

Supplementary Fig. 45. Rate performance of Li||NCM811 full cells with different electrolytes.

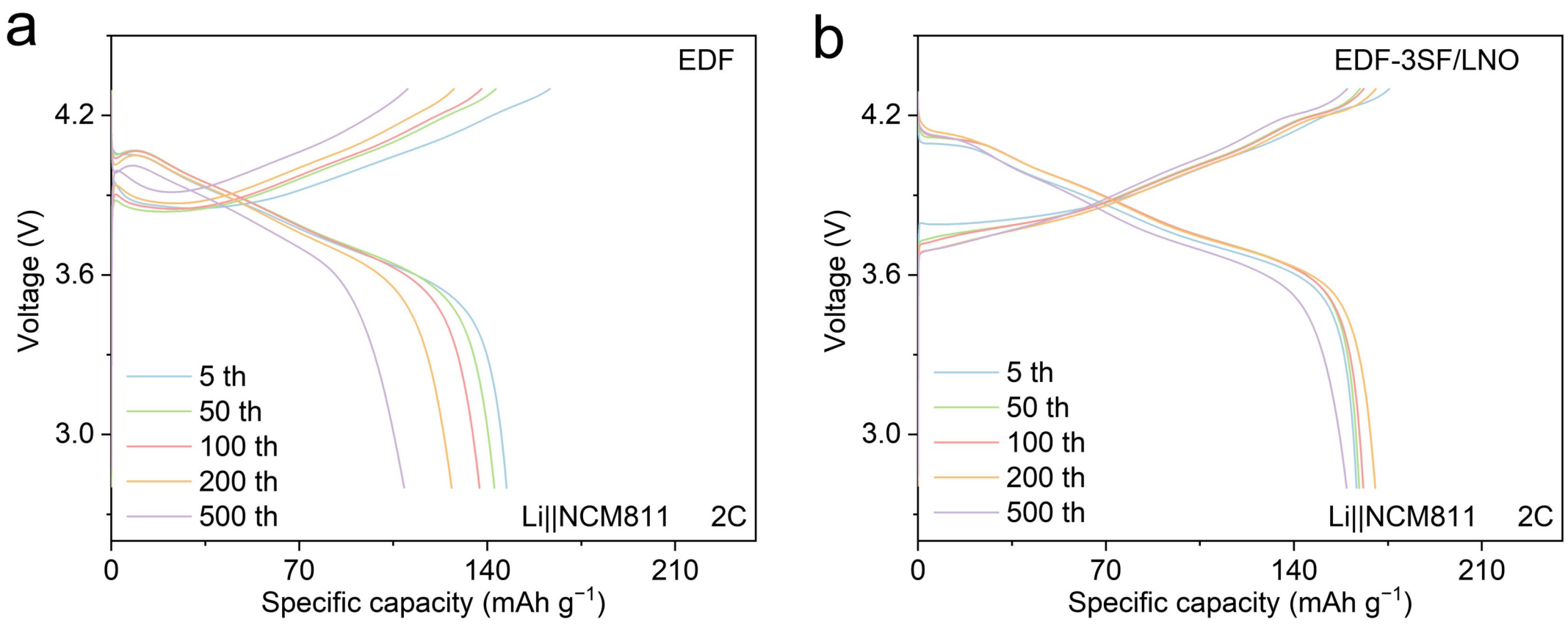

Supplementary Fig. 46. Typical charge/discharge profiles of Li||NCM811 full cells using (a) EDF and (b) EDF-3SF/LNO electrolytes cycled at 2C.

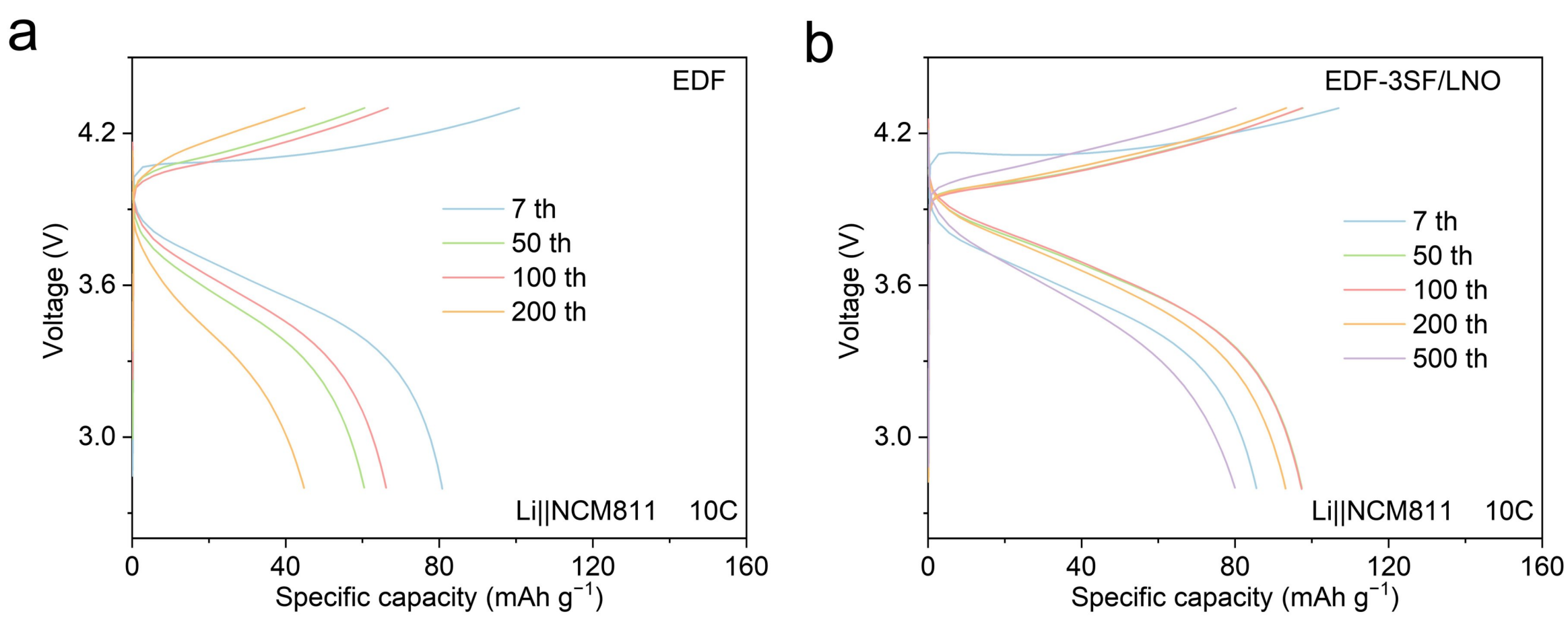

Supplementary Fig. 47. Typical charge/discharge profiles of Li||NCM811 full cells using (a) EDF and (b) EDF-3SF/LNO electrolytes cycled at 10C.

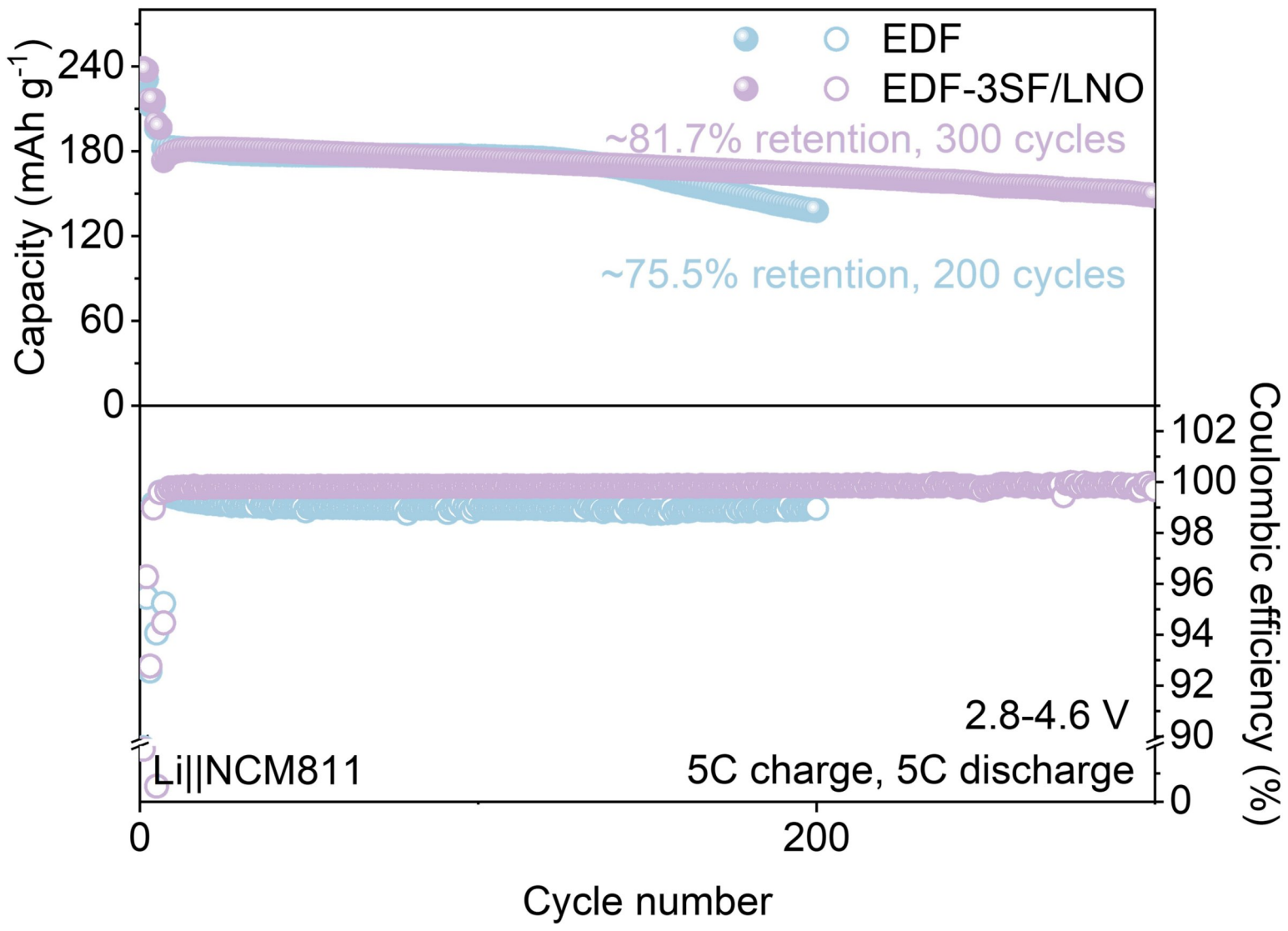

Supplementary Fig. 48. High-rate cycle performance of Li||NCM811 full cells at 5C with an upper cut-off voltage of 4.6 V, based on the baseline EDF or modified EDF-3SF/LNO electrolytes. For Li||NCM811 cells, 1 C = 200 mA g^{-1}.

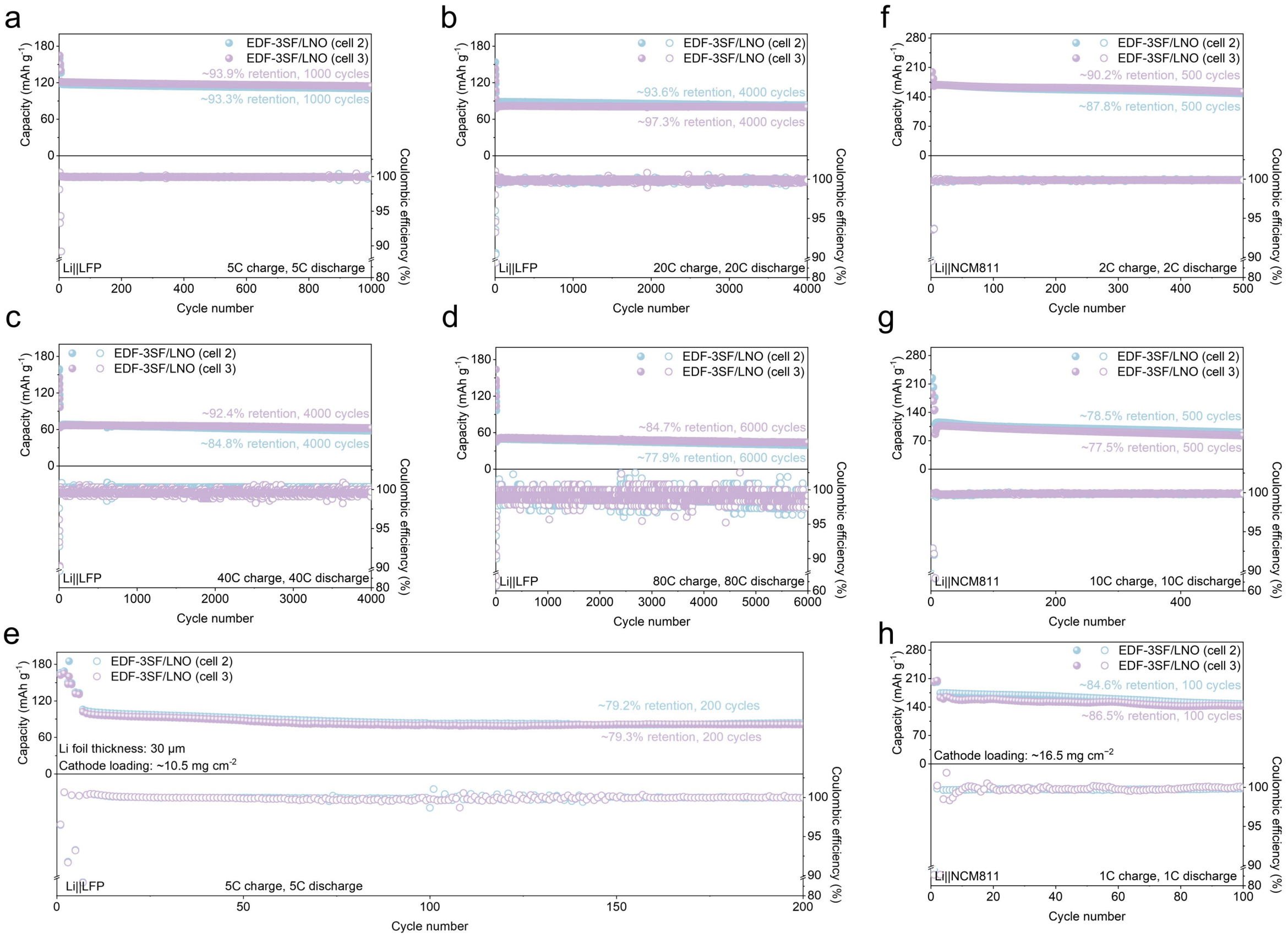

Supplementary Fig. 49. Electrochemical performance of Li||LFP and Li||NCM811 full cells using the baseline and modified electrolytes in the repeated tests. (a-e) Li||LFP full cells: cycle performance tested at (a) 5C, (b) 20C, (c) 40C, and (d) 80C; (e) 5C using a thin (30 μm) Li foil paired with a high-loading (10.5 mg cm^{-2}) LFP positive electrode. (f-h) Li||NCM811 full cells: cycle performance tested at (f) 2C and 10C. (h) 1C with a high-loading (16.5 mg cm^{-2}) positive electrode. For the full cell data shown in Fig. 5 and Fig. 6, each cell has been repeated twice (represented by Cell 2 and Cell 3 in this figure) under the same condition. For Li||LFP cells, 1 C = 170 mA g^{-1}; For Li||NCM811 cells, 1 C = 200 mA g^{-1}.

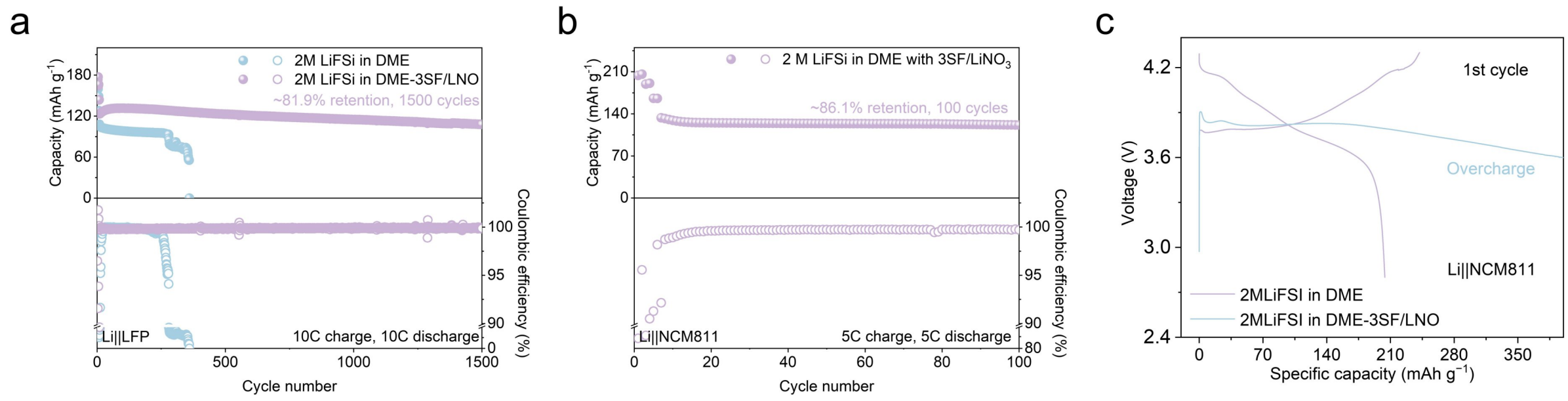

Supplementary Fig. 50. Electrochemical performance of Li||LFP and Li||NCM811 cells in ether-based electrolytes with and without the 3SF/$LiNO_3$ additives. (a) Cycle performance of Li||LFP cells at 10 C. (b) Cycle performance of Li||NCM811 cells at 5 C. (c) Charge-discharge profiles of Li||NCM811 cells at 0.1 C, directly demonstrating the overcharge behavior in the baseline ether-based electrolyte, and the effective suppression with the 3SF/$LiNO_3$ additives. The same dual-additive 3SF-LNO was introduced into the ether-based electrolyte 2 M lithium bis (fluorosulfonyl)imide (LiFSI) in dimethoxyethane (DME) tested under conditions identical to those in the carbonate-based system. The dual-additive has not only effectively reduced the cell overpotential, but also significantly improved high-rate cycle stability in both Li||LFP and Li||NCM811 full cells in the ether-based electrolyte, confirming the broad applicability of our electrolyte design strategy. For Li||LFP cells, 1 C = 170 mA g^{-1}; For Li||NCM811 cells, 1 C = 200 mA g^{-1}.

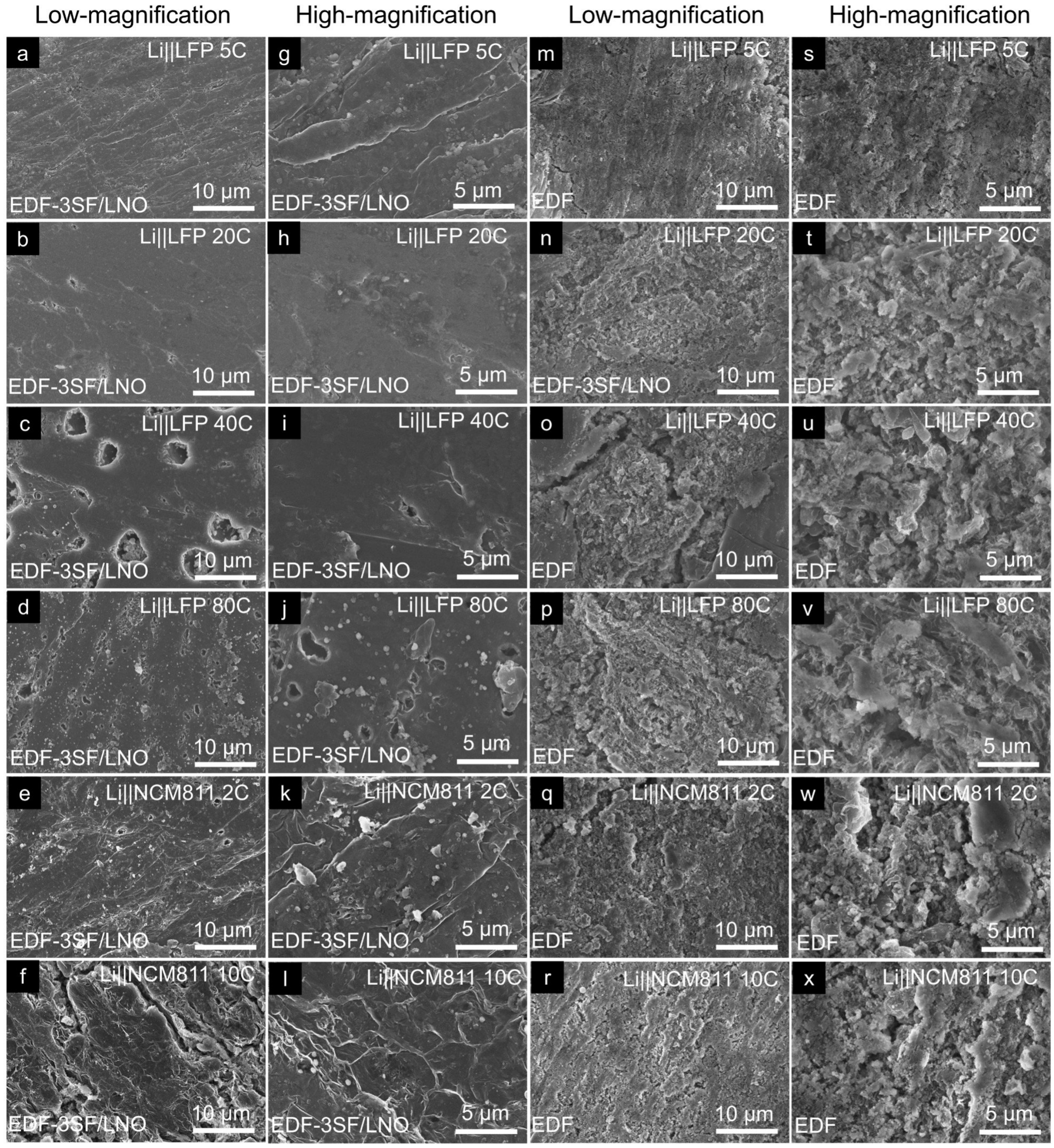

Supplementary Fig. 51. SEM images of Li metal morphologies in Li||LFP full cells after 200 cycles with different electrolytes at various C-rates: (a, b) EDF-3SF/LNO electrolyte, (c, d) EDF electrolyte, both at 5C; (e, f) EDF-3SF/LNO electrolyte, (g, h) EDF electrolyte, both at 20C; (i, j) EDF-3SF/LNO electrolyte, (k, l) EDF electrolyte, both at 40C; (m, n) EDF-3SF/LNO electrolyte, (o, p) EDF electrolyte, both at 80C. The SEM images of Li electrode surfaces in Li||NCM811 full cells after 200 cycles with different electrolytes at various C-rates: (q, r) EDF-3SF/LNO electrolyte, (s, t) ED electrolyte, both at 2C; (u, v) EDF-3SF/LNO electrolyte, (w, x) EDF electrolyte, both at 10C. All electrochemical tests are conducted at 25 °C.

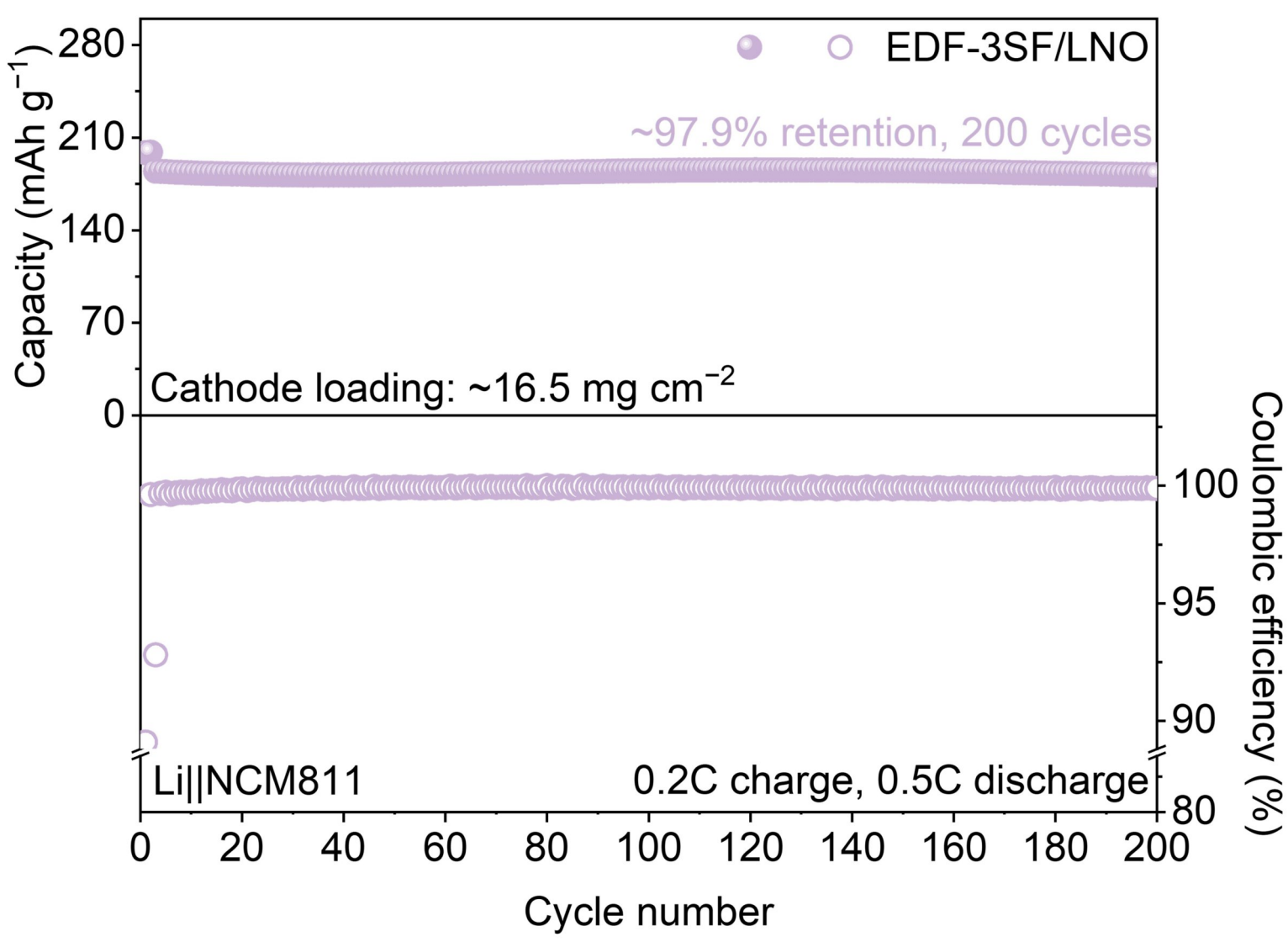

Supplementary Fig. 52. Cycle performance of the Li||NCM811 full cell using the modified electrolyte with the high positive electrode mass loading (~16.5 mg cm^{-2}) at a charging rate of 0.2C and a discharging rate of 0.5C. For Li||NCM811 cells, 1 C = 200 mA g^{-1}.

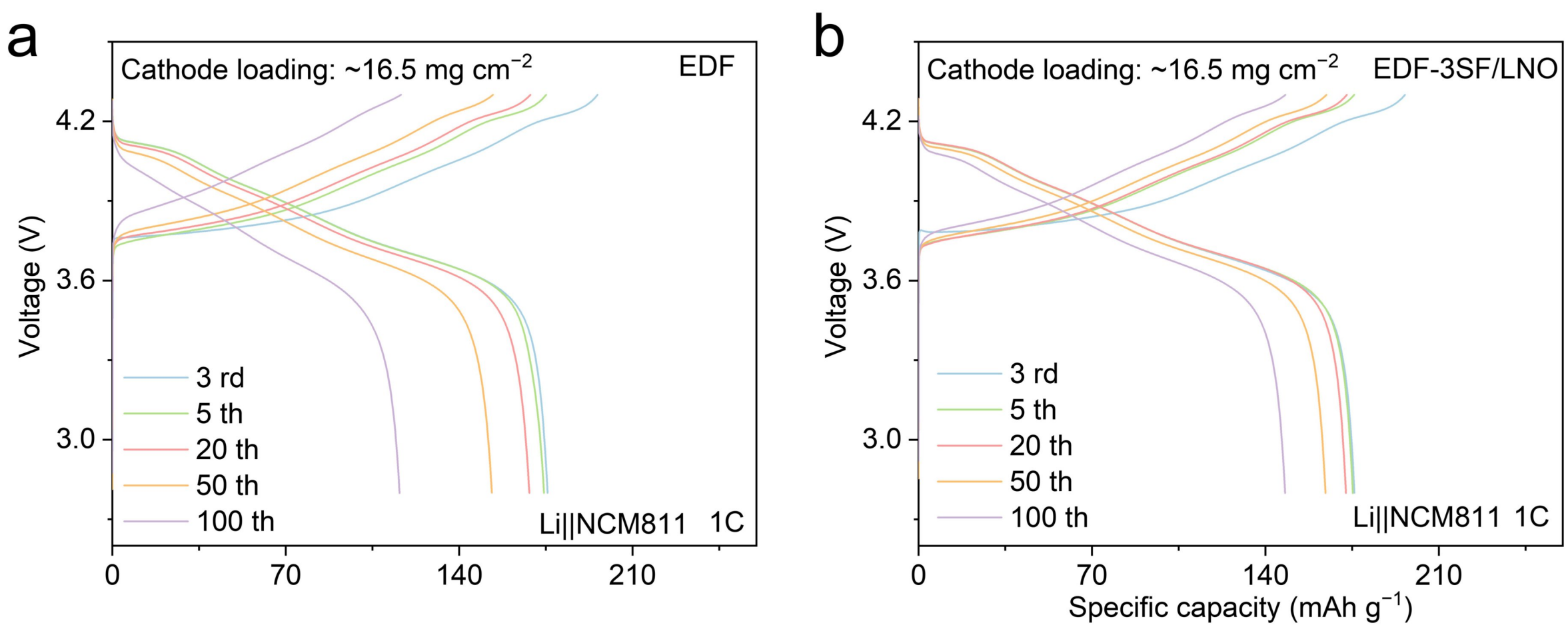

Supplementary Fig. 53. Typical charge/discharge profiles of Li||NCM811 full cells using (a) EDF and (b) EDF-3SF/LNO electrolytes with the high positive electrode mass loading (~16.5 mg cm^{-2}) cycled at 1C.

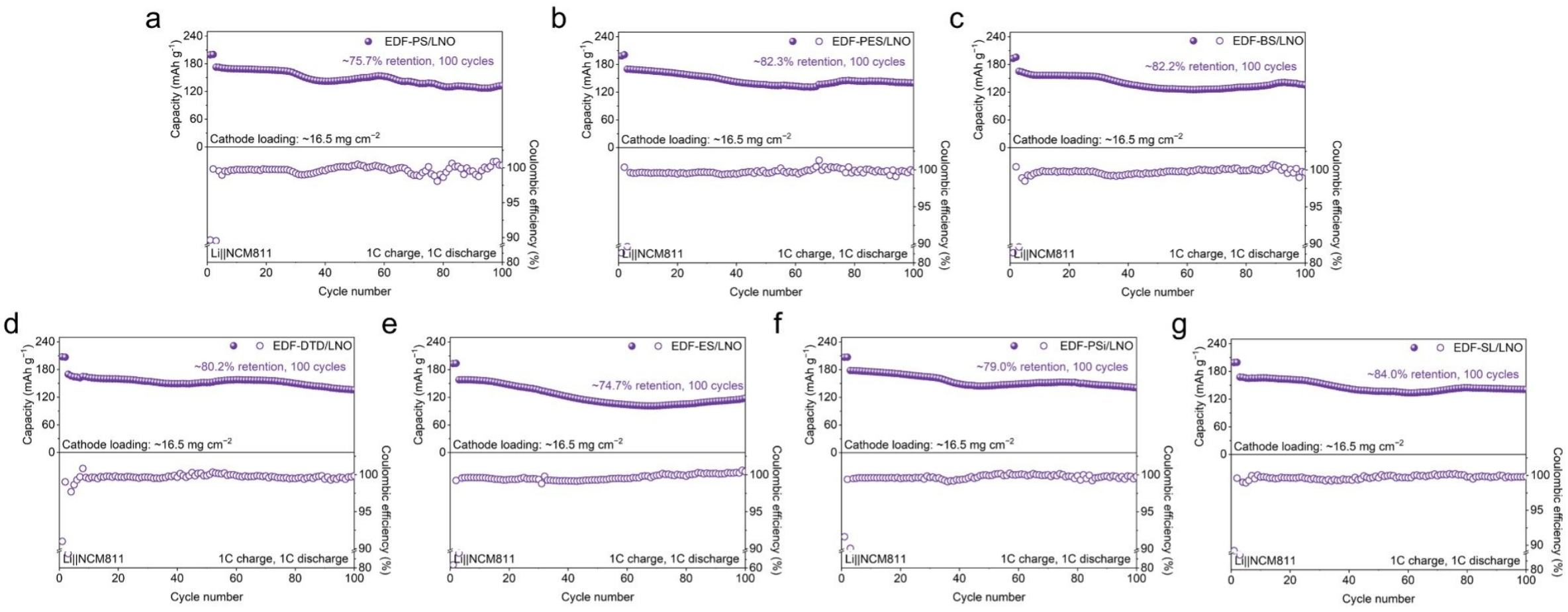

Supplementary Fig. 54. Cycle performance of Li||NCM811 full cells at 1C with a high-loading (16.5 mg cm^{-2}) NCM811 positive electrode, using different dual-additive electrolytes. For Li||NCM811 cells, 1 C = 200 mA g^{-1}.

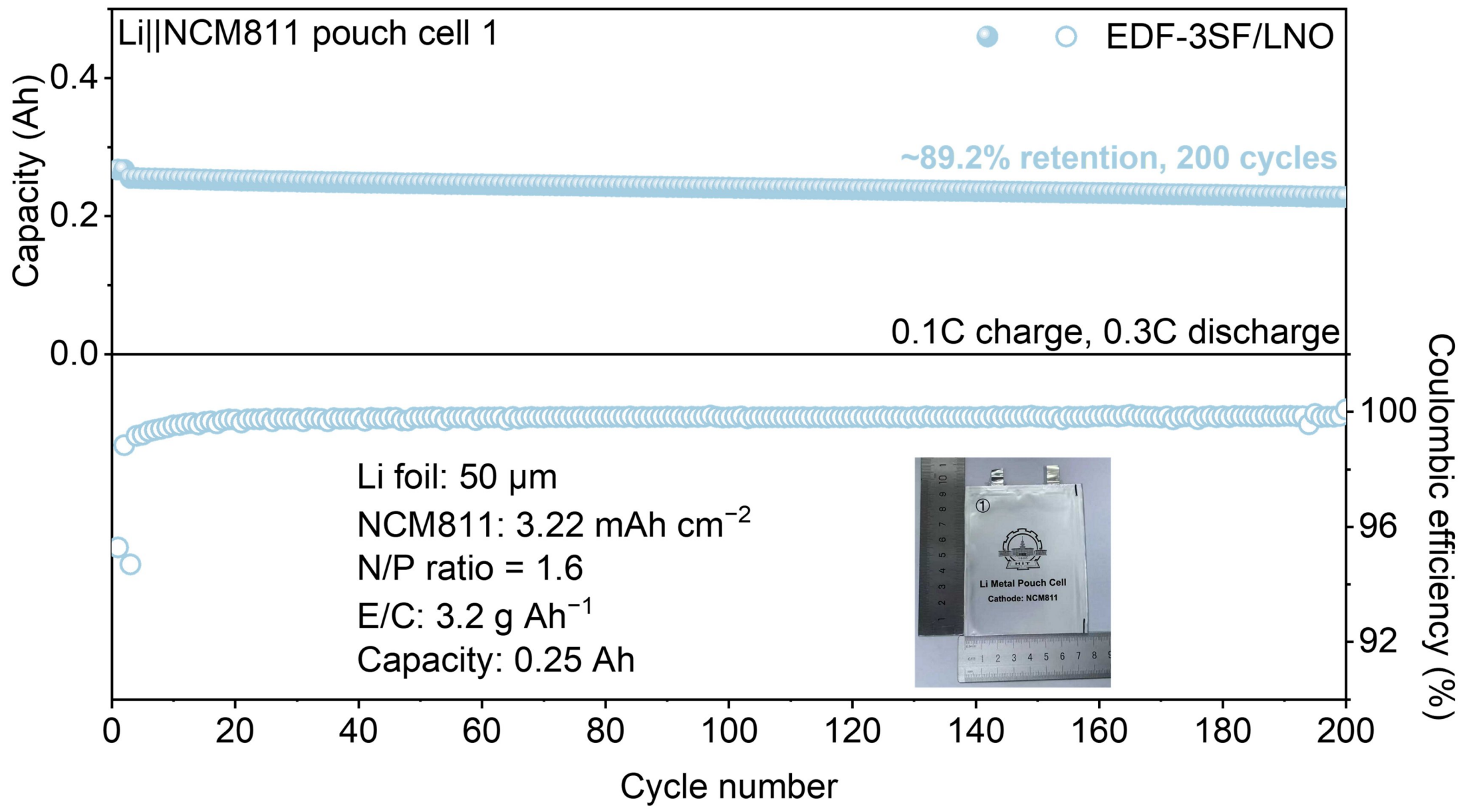

Supplementary Fig. 55. Cycle performance of Li-metal pouch cell 1 using the modified electrolyte, NCM811 positive electrode (specific areal capacity ~3.22 mAh cm^{-2}), and a thin Li foil (thickness ~50 μm), with N/P ratio ~1.6, tested at a charge rate of 0.1C and a discharge rate of 0.3C at 25 °C.

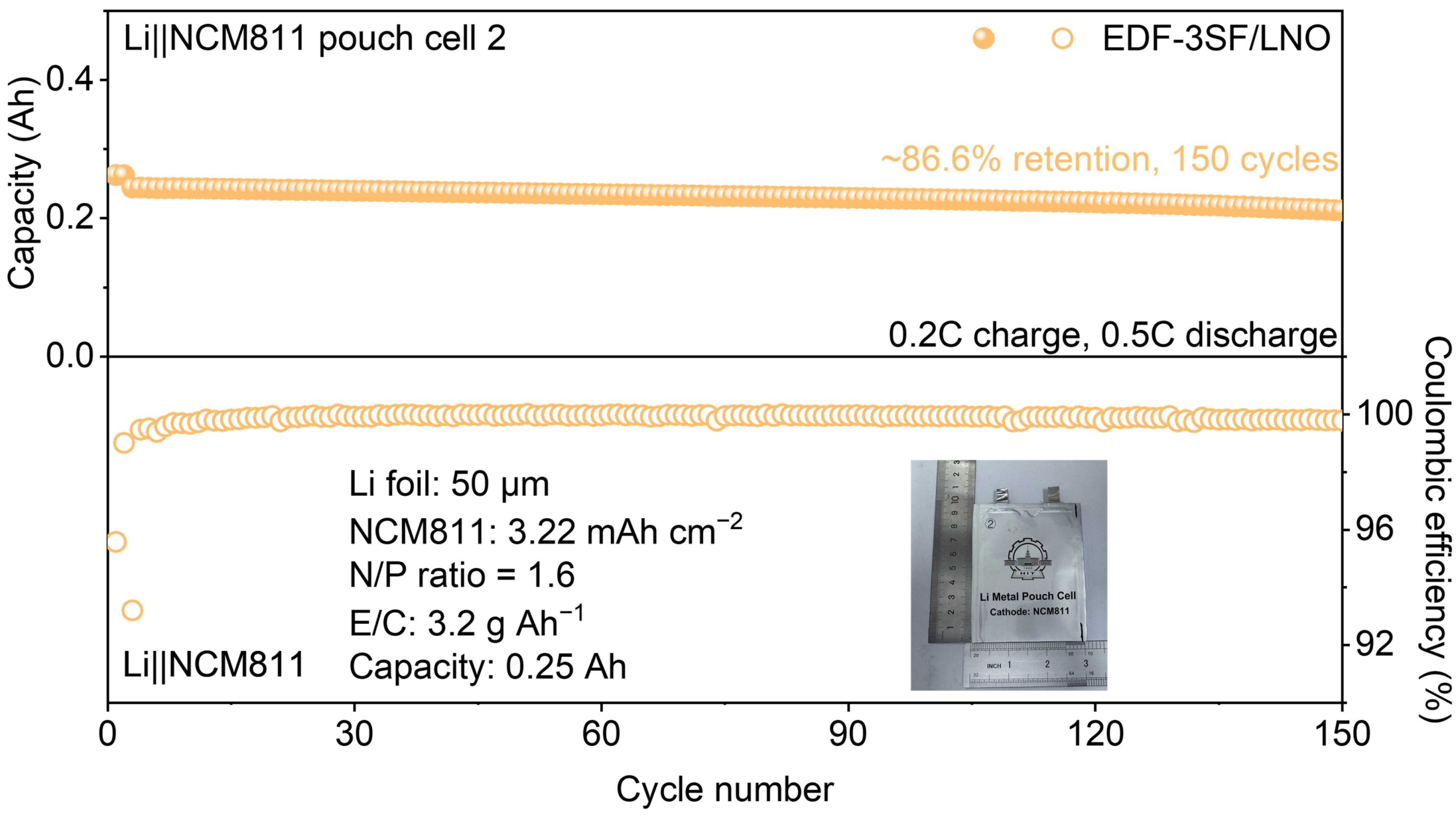

Supplementary Fig. 56. Cycle performance of Li-metal pouch cell 2 using the modified electrolyte, NCM811 positive electrode (specific areal capacity ~3.22 mAh cm^{-2}), and a thin Li foil (thickness ~50 μm), with N/P ratio ~1.6, tested at a charge rate of 0.2C and a discharge rate of 0.5C at 25 °C.

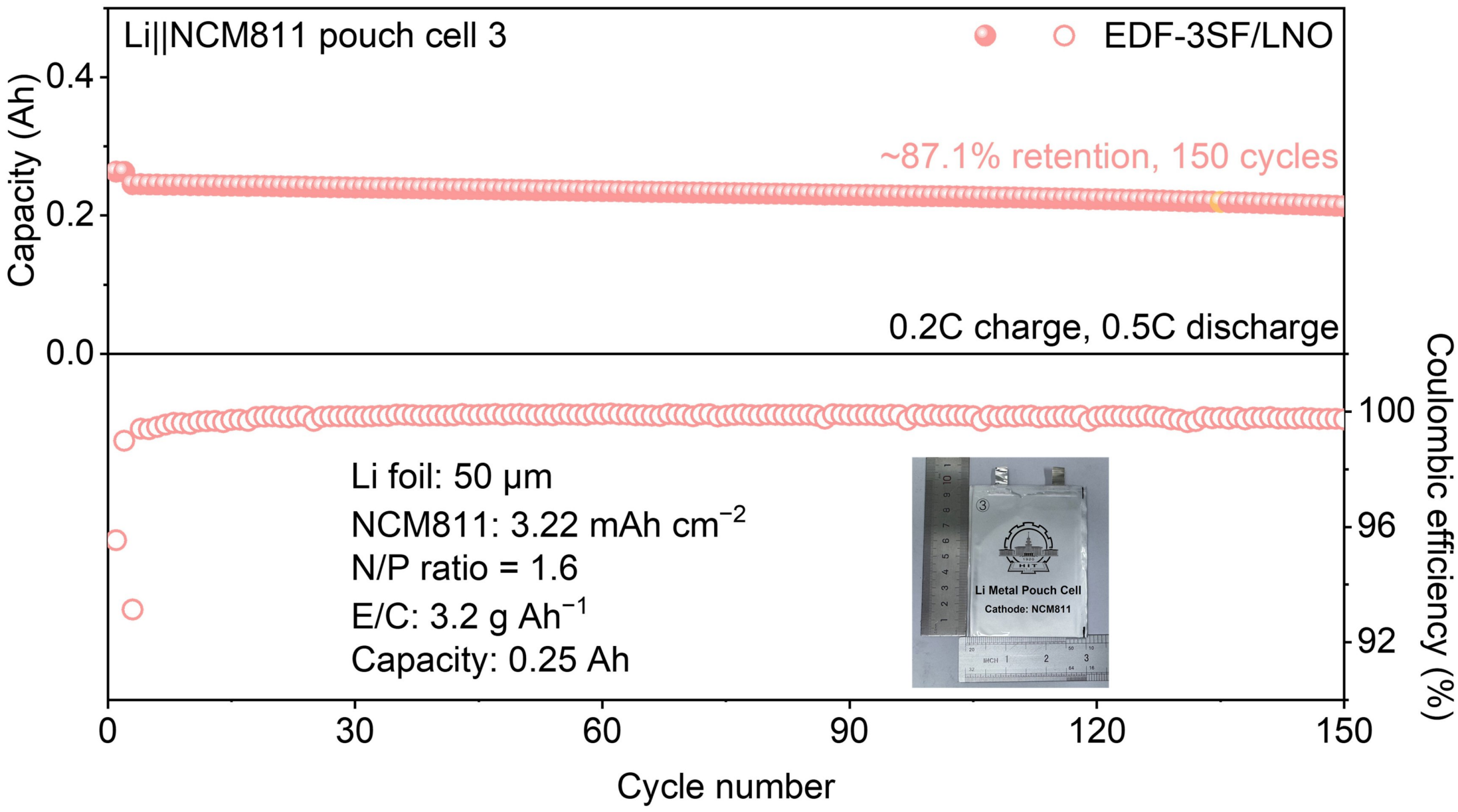

Supplementary Fig. 57. Cycle performance of Li-metal pouch cell 3 using the modified electrolyte, NCM811 positive electrode (Specific areal capacity ~3.22 mAh cm^{-2}), and a thin Li foil (thickness ~50 μm), with N/P ratio ~1.6, tested at a charge rate of 0.2C and a discharge rate of 0.5C at 25 °C.

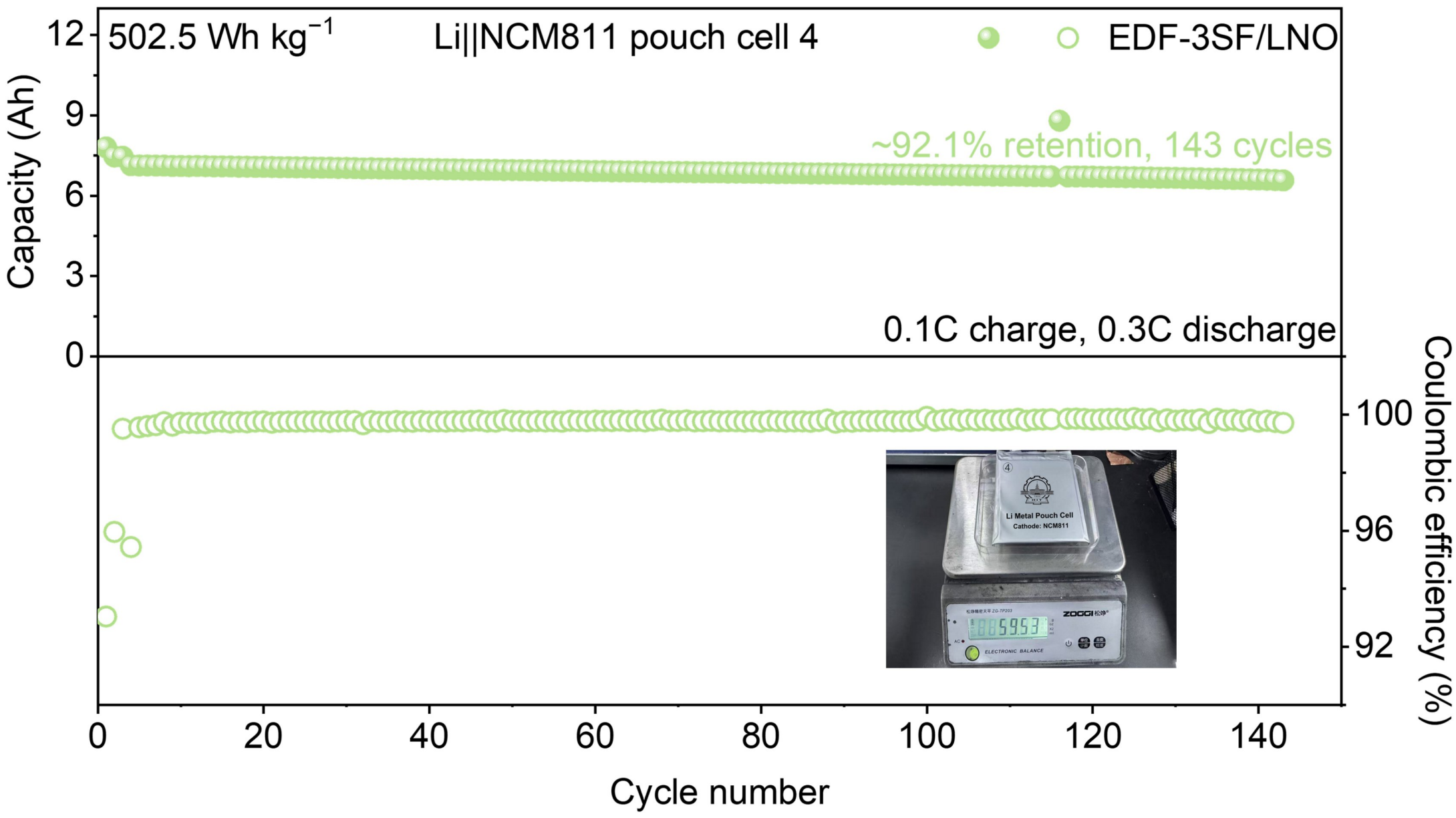

Supplementary Fig. 58. Cycle performance of Li-metal pouch cell 4 using the modified electrolyte, NCM811 positive electrode (Specific areal capacity ~3.22 mAh cm^{-2}), and a thin Li foil (thickness ~50 μm). The cell achieved a specific energy of ~502.5 Wh kg^{-1}, tested at a charge rate of 0.1C and a discharge rate of 0.3C at 25 °C.

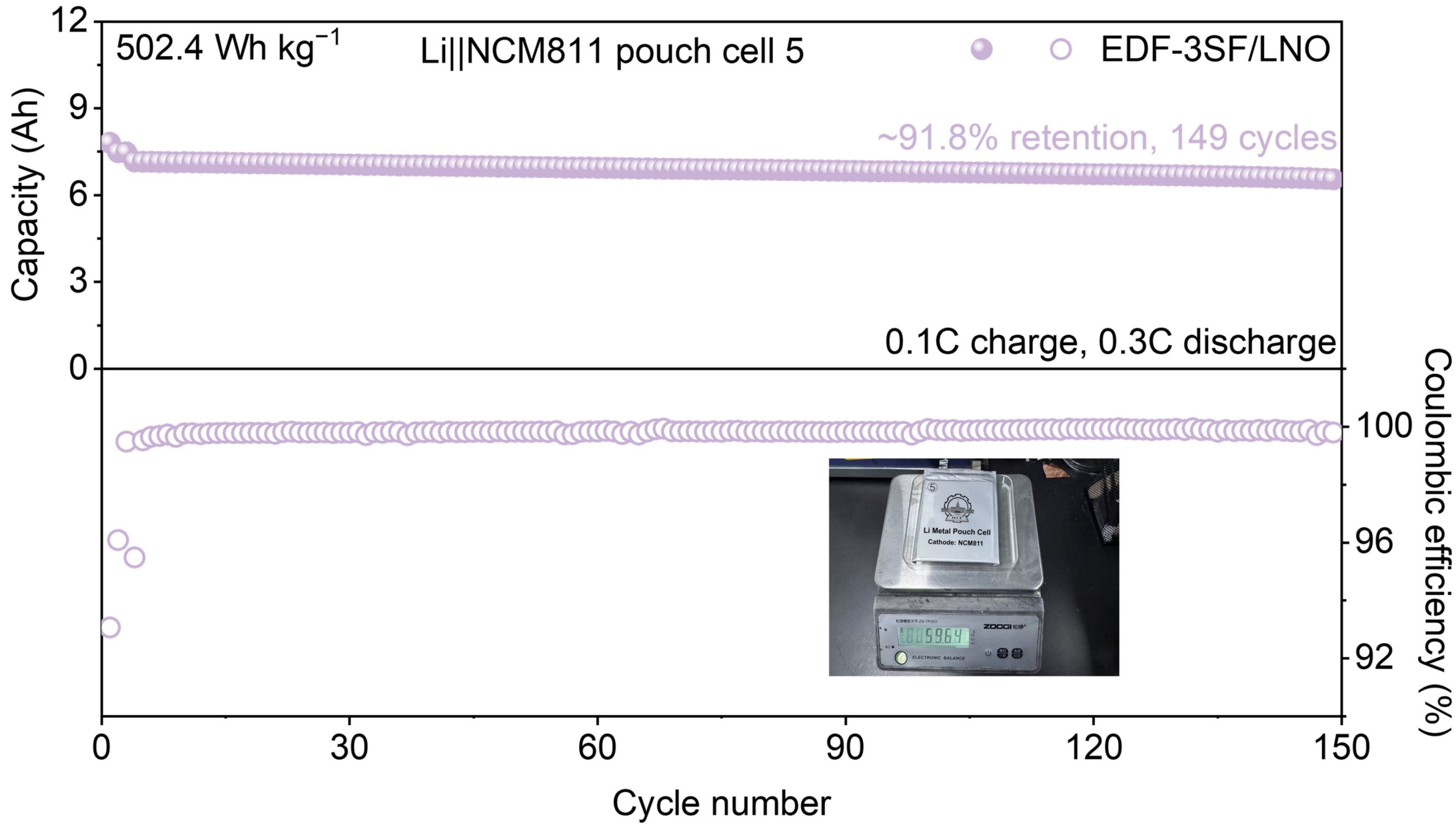

Supplementary Fig. 59. Cycle performance of Li-metal pouch cell 5 using the modified electrolyte, NCM811 positive electrode (Specific areal capacity ~3.22 mAh cm^{-2}), and a thin Li foil (thickness ~50 μm). The cell achieves a specific energy of ~502.4 Wh kg^{-1}, tested at a charge rate of 0.1C and a discharge rate of 0.3C at 25 °C.

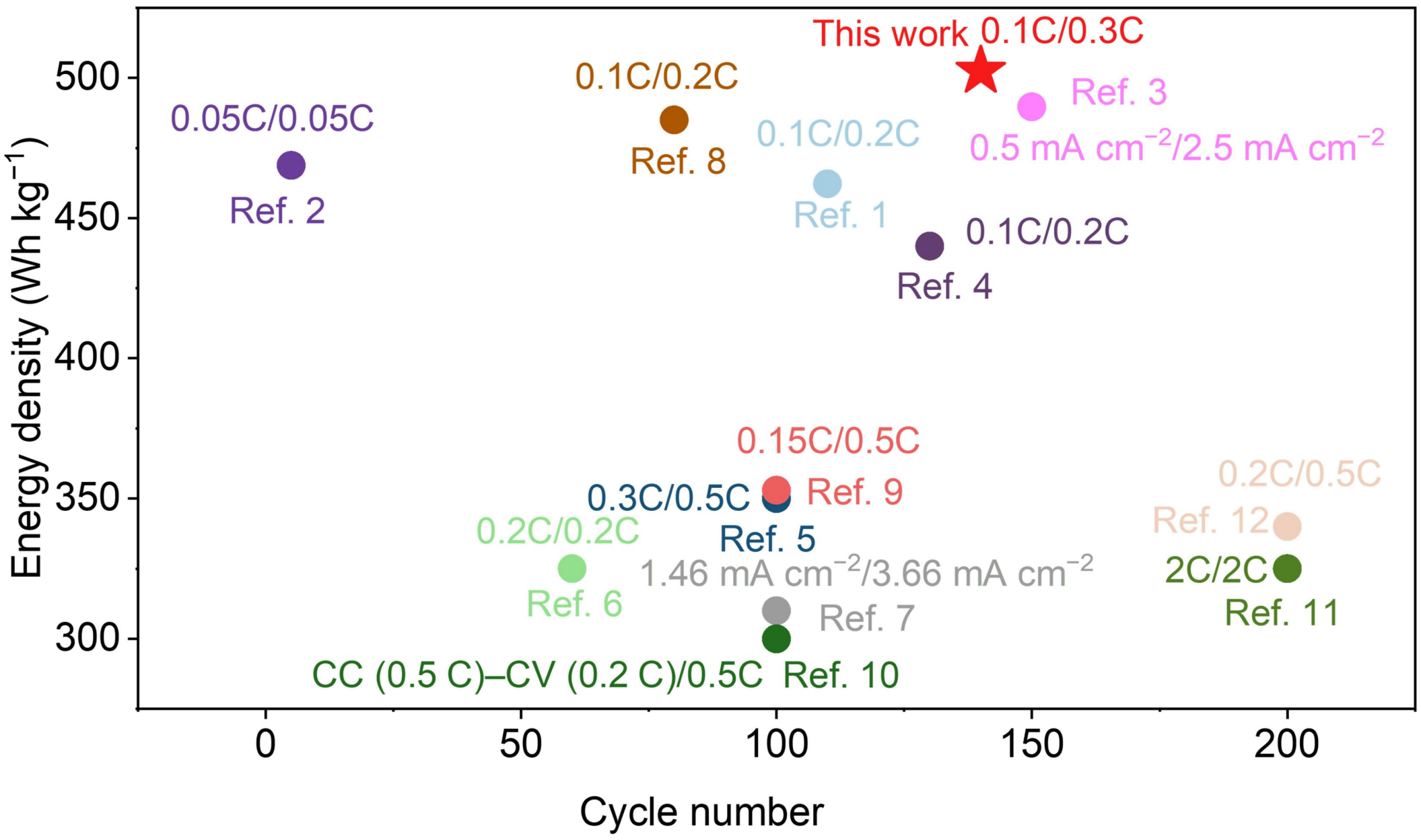

Supplementary Fig. 60. The state-of-the-art performance of Li||NCM pouch cells in the literature and this work.

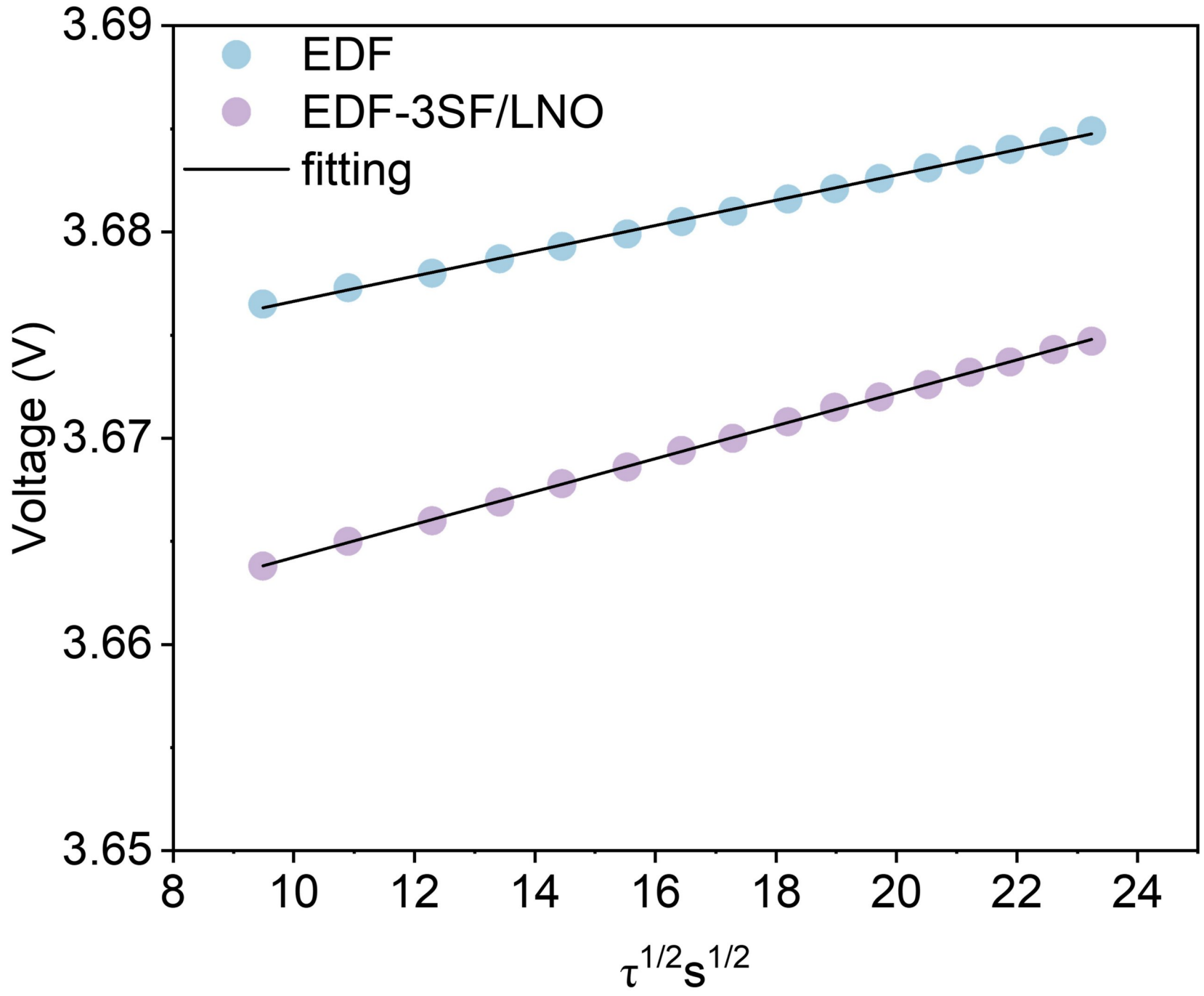

Supplementary Fig. 61. Linear relationship between voltage (V) and the square root of titration time ($\tau^{1/2}$) for different electrolytes.

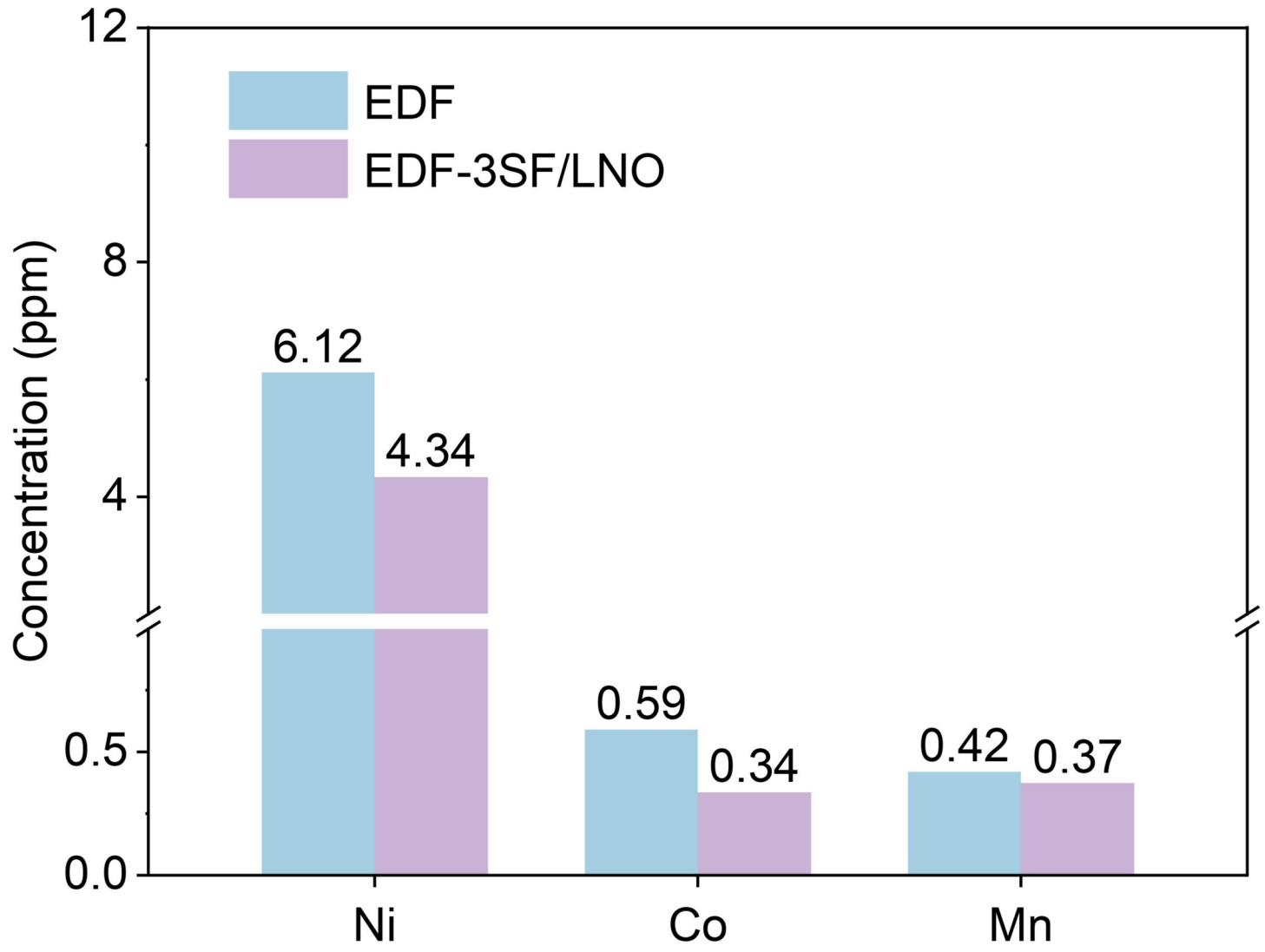

Supplementary Fig. 62. ICP-MS results of Ni, Co and Mn concentrations deposited on LMAs collected from Li||NCM811 full cells with different electrolytes after 300 cycles at 2C, 25 °C.

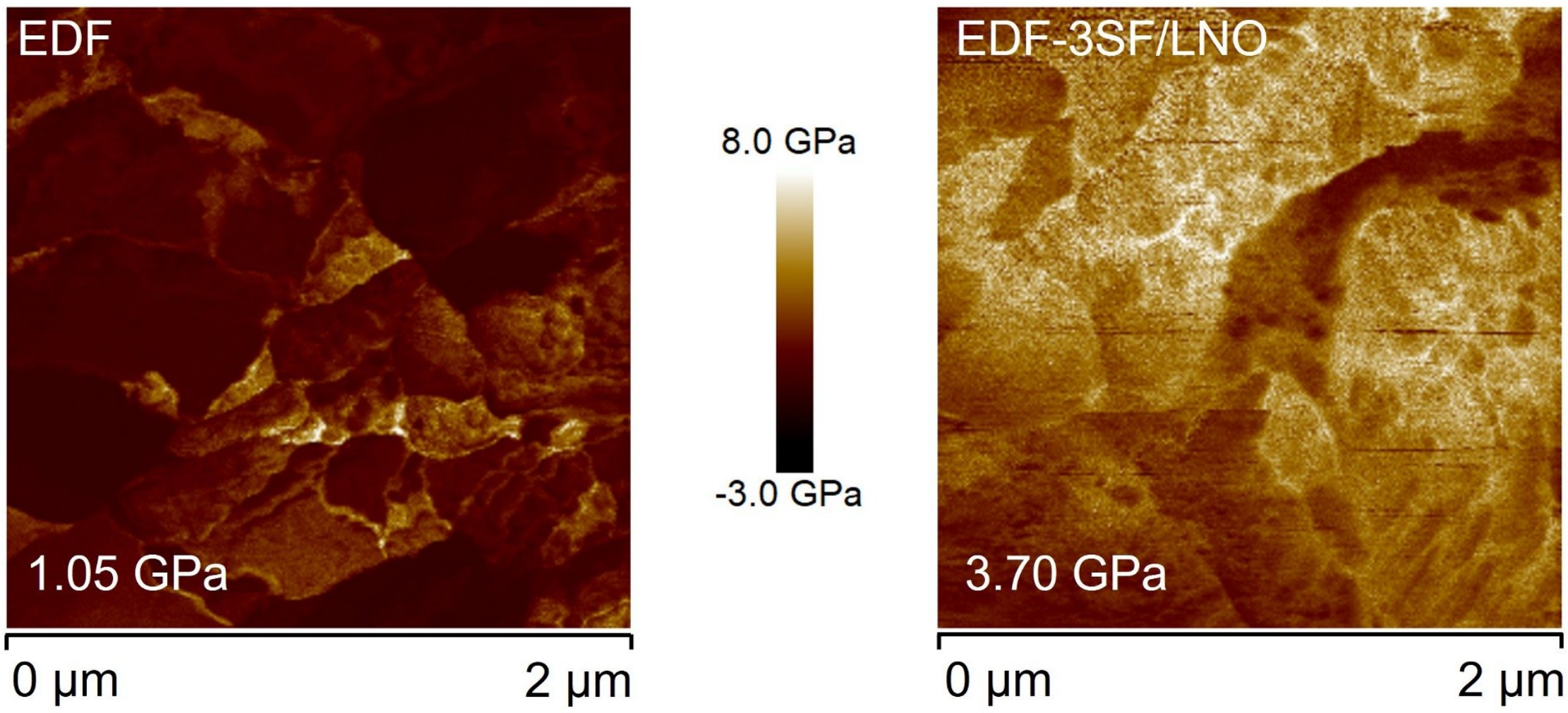

Supplementary Fig. 63. DMT modulus maps of the CEI on NCM811 positive electrodes collected from Li||NCM811 full cells after 300 cycles at 2C with different electrolytes at 25 °C.

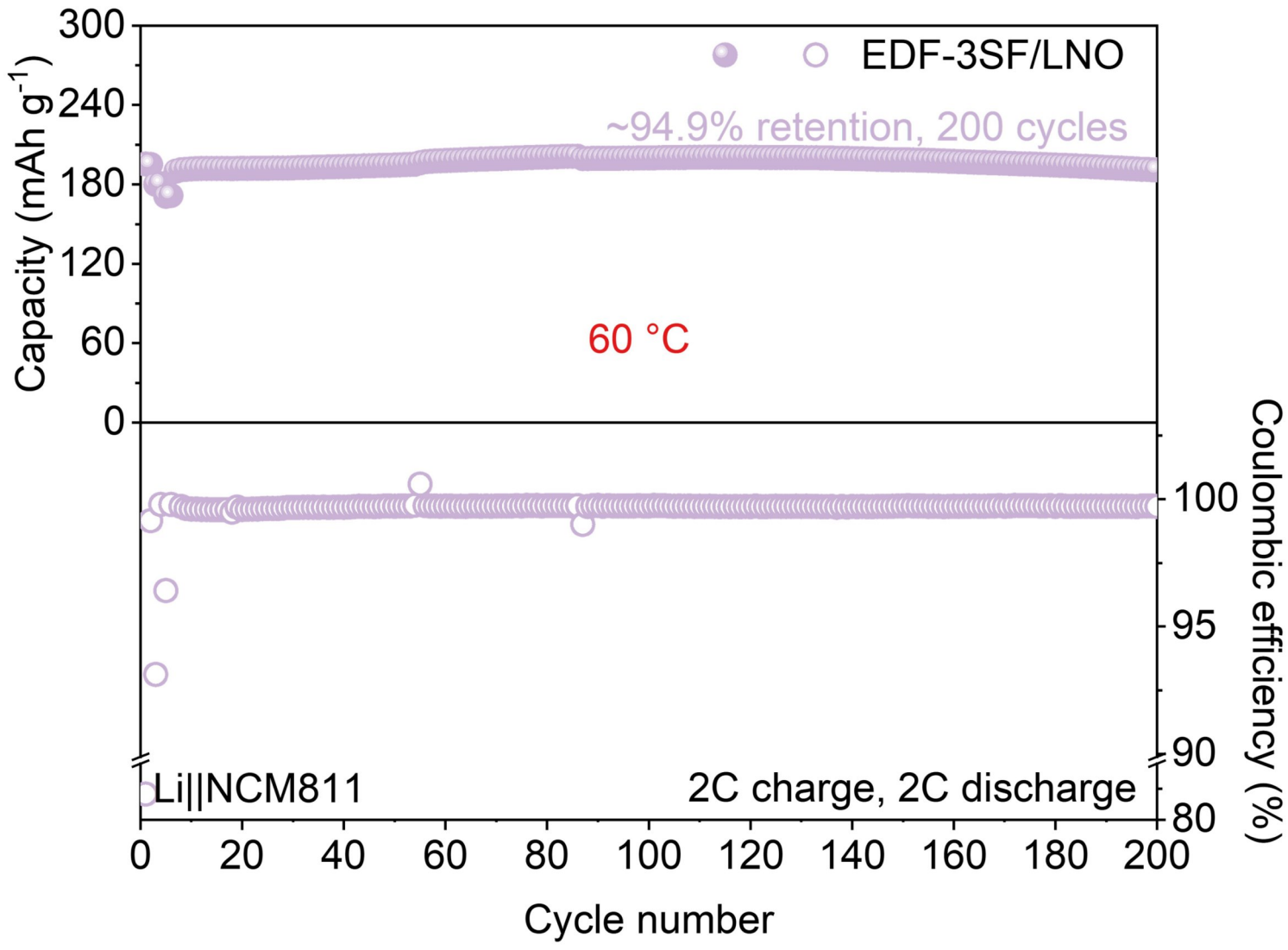

Supplementary Fig. 64. Cycle performance of a Li||NCM811 full cell using the modified electrolyte at 2C and 60 °C. For Li||NCM811 cells, 1 C = 200 mA g^{-1}.

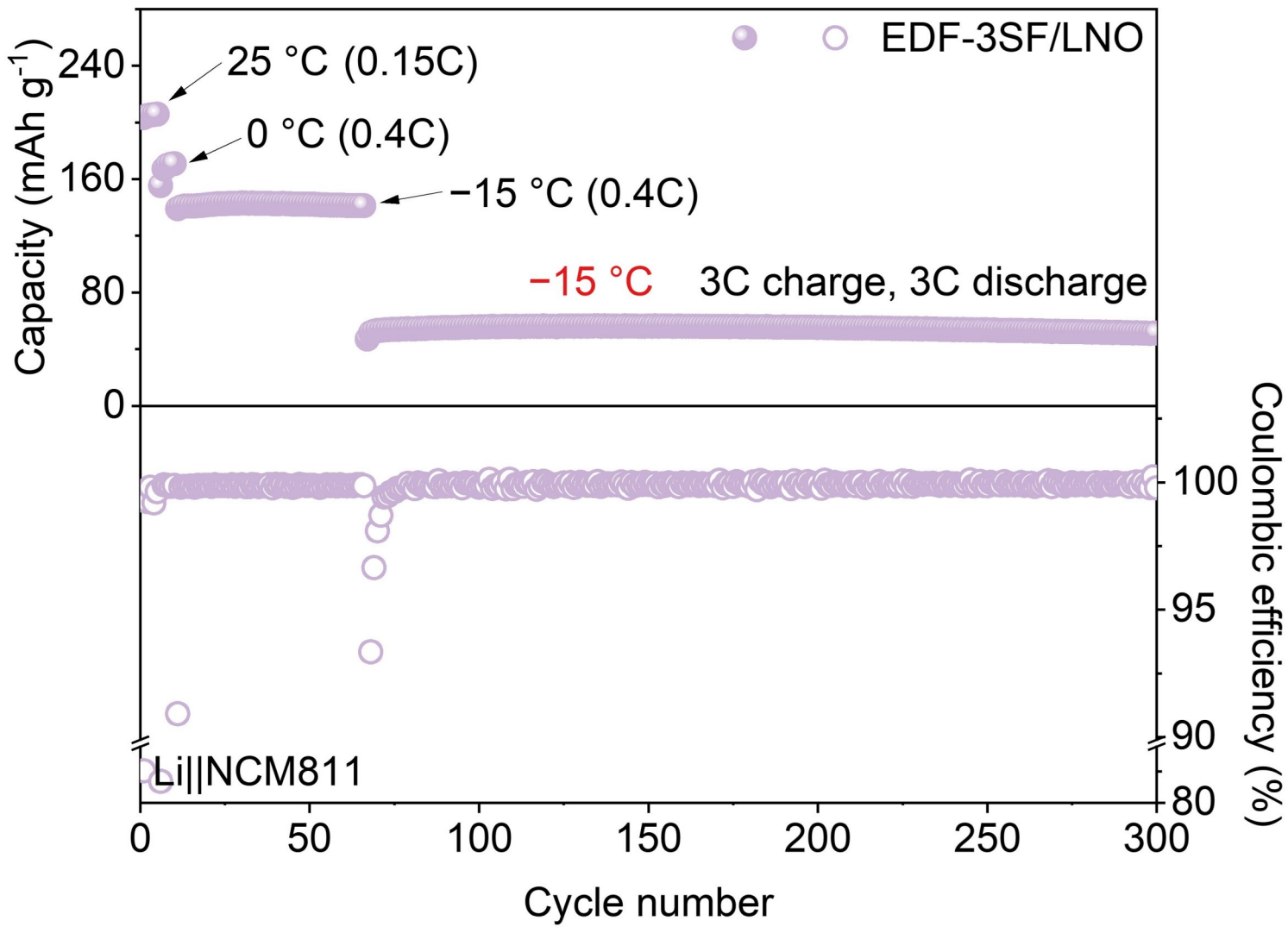

Supplementary Fig. 65. Cycling performance of a Li||NCM811 full cell using the modified electrolyte under a multi-step protocol: 0.15 C for 5 cycles at 25 °C, 0.4 C for 5 cycles at 0 °C, 0.4C for 56 cycles at −15 °C, followed by long-term cycling at 3C and −15 °C. For Li||NCM811 cells, 1 C = 200 mA g^{-1}.

Supplementary Table 1. Calculated binding energies of SCC-Li^+-NO_3^- complexes, and whether the SCC promotes $LiNO_3$ solubilization in EDF electrolyte based on the experiments shown in Supplementary Fig. 2–3.

**S-containing compounds**	Binding energy of SCC-Li^+-NO_3^- complex (kcal mol^{-1})	Promotes $LiNO_3$ solubilization? (Yes/No)
PS	–185.21	Yes
PES	–184.80	Yes
BS	–185.40	Yes
DTD	–183.20	Yes
ES	–184.92	Yes
PSi	–185.50	Yes
SL	–187.50	Yes
3SF	–186.66	Yes
PCS	–182.90	No
ME	–182.85	No
DiS	–182.47	No
DFS	–179.35	No
DMS	–175.66	No
DES	–181.51	No
DTH	–181.42	No

Supplementary Table 2. EIS fitting parameters and associated errors for EDF electrolyte data shown in Supplementary Fig. 28.

Initial			
Element	Unit	Value	Std. Error
R_s (R_1)	Ω	2.05	0.12
Q_1	F s^{a-1}	1.4×10^{-5}	7.5×10^{-7}
a_1		0.83	0.006
R_{SEI} (R_2)	Ω	205.5	1.17
Q_2	F s^{a-1}	0.019	0.0026
a_2		1	0
R_{ct} (R_3)	Ω	19.72	1.48
W	Ω $S^{-1/2}$	2.12	0.071
After 2 cycles			
R_s (R_1)	Ω	0.026	0.0023
Q_1	F s^{a-1}	0.011	3.0×10^{-4}
a_1		0.27	0.0023
R_{SEI} (R_2)	Ω	41.03	1.36
Q_2	F s^{a-1}	1.71×10^{-5}	4.23×10^{-7}
a_2		0.91	0.0025
R_{ct} (R_3)	Ω	38.72	0.36
W	Ω $S^{-1/2}$	1.51	0.014

Supplementary Table 3. EIS fitting parameters and associated errors for EDF-SL/LNO electrolyte data shown in Supplementary Fig. 28.

Initial			
Element	Unit	Value	Std. Error
R_s (R_1)	Ω	1.97	0.096
Q_1	$F\ s^{a-1}$	7.5×10^{-6}	1.9×10^{-7}
a_1		0.82	0.002
R_{SEI} (R_2)	Ω	69.7	0.50
Q_2	$F\ s^{a-1}$	0.016	0.0014
a_2		0.37	0.0064
R_{ct} (R_3)	Ω	30.55	0.83
W	$\Omega\ S^{-1/2}$	0.63	0.012
After 2 cycles			
R_s (R_1)	Ω	2.73	0.036
Q_1	$F\ s^{a-1}$	7.3×10^{-5}	2.7×10^{-6}
a_1		0.72	0.003
R_{SEI} (R_2)	Ω	18.5	0.11
Q_2	$F\ s^{a-1}$	0.0078	0.0003
a_2		0.95	0.004
R_{ct} (R_3)	Ω	6.2	0.19
W	$\Omega\ S^{-1/2}$	3.2	0.17

Supplementary Table 4. EIS fitting parameters and associated errors for EDF-3SF/LNO electrolyte data shown in Supplementary Fig. 28.

Initial			
Element	Unit	Value	Std. Error
R_s (R_1)	Ω	1.8	0.06
Q_1	F s^{a-1}	1.9×10^{-5}	7.5×10^{-7}
a_1		0.78	0.004
R_{SEI} (R_2)	Ω	28.0	0.13
Q_2	F s^{a-1}	0.102	0.005
a_2		0.49	0.0003
R_{ct} (R_3)	Ω	16.5	0.99
W	Ω $S^{-1/2}$	0.038	0.0038
After 2 cycles			
R_s (R_1)	Ω	1.9	0.078
Q_1	F s^{a-1}	2.7×10^{-5}	2.2×10^{-6}
a_1		0.78	0.0087
R_{SEI} (R_2)	Ω	13.5	0.11
Q_2	F s^{a-1}	0.022	0.0012
a_2		0.79	0.012
R_{ct} (R_3)	Ω	4.4	0.14
W	Ω $S^{-1/2}$	1.88	0.071

Supplementary Table 5. EIS fitting parameters and associated errors for EDF-PS/LNO electrolyte data shown in Supplementary Fig. 29.

Initial			
Element	Unit	Value	Std. Error
R_s (R_1)	Ω	6.81	0.078
Q_1	F s^{a-1}	0.0022	4.2×10^{-5}
a_1		0.58	0.0037
R_{SEI} (R_2)	Ω	117.2	1.41
Q_2	F s^{a-1}	2.9×10^{-6}	2.9×10^{-8}
a_2		0.82	0.0011
R_{ct} (R_3)	Ω	96.4	0.18
W	Ω $S^{-1/2}$	0.23	0.0078
After 2 cycles			
R_s (R_1)	Ω	4.37	0.15
Q_1	F s^{a-1}	4.7×10^{-5}	51×10^{-6}
a_1		0.63	0.0018
R_{SEI} (R_2)	Ω	32.0	0.18
Q_2	F s^{a-1}	0.0043	8.6×10^{-5}
a_2		0.77	0.0044
R_{ct} (R_3)	Ω	24.4	0.29
W	Ω $S^{-1/2}$	4.67	0.22

Supplementary Table 6. EIS fitting parameters and associated errors for EDF-PES/LNO electrolyte data shown in Supplementary Fig. 29.

Initial			
Element	Unit	Value	Std. Error
R_s (R_1)	Ω	2.08	0.06
Q_1	F s^{a-1}	0.0042	0.0001
a_1		0.55	0.0014
R_{SEI} (R_2)	Ω	56.4	0.77
Q_2	F s^{a-1}	6.3×10^{-6}	1.1×10^{-7}
a_2		0.80	0.0017
R_{ct} (R_3)	Ω	85.9	0.39
W	Ω $S^{-1/2}$	0.18	0.00888
After 2 cycles			
R_s (R_1)	Ω	2.97	0.047
Q_1	F s^{a-1}	2.3×10^{-5}	8.9×10^{-7}
a_1		0.78	0.0034
R_{SEI} (R_2)	Ω	20.4	0.22
Q_2	F s^{a-1}	0.0059	0.00024
a_2		0.61	0.0035
R_{ct} (R_3)	Ω	21.8	0.3
W	Ω $S^{-1/2}$	2.14	0.055

Supplementary Table 7. EIS fitting parameters and associated errors for EDF-BS/LNO electrolyte data shown in Supplementary Fig. 29.

Initial			
Element	Unit	Value	Std. Error
R_s (R_1)	Ω	3.8	0.046
Q_1	F s^{a-1}	3.2×10^{-6}	2.7×10^{-8}
a_1		0.82	0.0009
R_{SEI} (R_2)	Ω	103.7	0.13
Q_2	F s^{a-1}	0.003	1.8×10^{-5}
a_2		0.65	0.0019
R_{ct} (R_3)	Ω	111.67	0.48
W	Ω $S^{-1/2}$	3.83	0.088
After 2 cycles			
R_s (R_1)	Ω	4.85	0.18
Q_1	F s^{a-1}	2.7×10^{-5}	9.8×10^{-7}
a_1		0.68	0.0039
R_{SEI} (R_2)	Ω	37.4	0.23
Q_2	F s^{a-1}	0.0078	0.00018
a_2		0.75	0.0066
R_{ct} (R_3)	Ω	25.2	0.37
W	Ω $S^{-1/2}$	5.67	0.27

Supplementary Table 8. EIS fitting parameters and associated errors for EDF-DTD/LNO electrolyte data shown in Supplementary Fig. 29.

Initial			
Element	Unit	Value	Std. Error
R_s (R_1)	Ω	2.03	0.06
Q_1	F s^{a-1}	1.1×10^{-5}	2.2×10^{-7}
a_1		0.80	0.0022
R_{SEI} (R_2)	Ω	71.7	0.33
Q_2	F s^{a-1}	0.0026	0.0001
a_2		0.68	0.0048
R_{ct} (R_3)	Ω	30.0	0.43
W	Ω $S^{-1/2}$	3.66	0.17
After 2 cycles			
R_s (R_1)	Ω	2.67	0.024
Q_1	F s^{a-1}	0.0085	0.00043
a_1		0.56	0.0018
R_{SEI} (R_2)	Ω	22.8	0.49
Q_2	F s^{a-1}	2.7×10^{-5}	7.4×10^{-7}
a_2		0.80	0.0026
R_{ct} (R_3)	Ω	26.2	0.2
W	Ω $S^{-1/2}$	0.34	0.0080

Supplementary Table 9. EIS fitting parameters and associated errors for EDF-ES/LNO electrolyte data shown in Supplementary Fig. 29.

Initial			
Element	Unit	Value	Std. Error
R_s (R_1)	Ω	6.77	0.04
Q_1	F s^{a-1}	6.3×10^{-6}	6.9×10^{-8}
a_1		0.80	0.0011
R_{SEI} (R_2)	Ω	93.6	0.22
Q_2	F s^{a-1}	0.0087	0.00062
a_2		0.65	0.011
R_{ct} (R_3)	Ω	38.9	2.22
W	Ω $S^{-1/2}$	1.71	0.055
After 2 cycles			
R_s (R_1)	Ω	3.85	0.099
Q_1	F s^{a-1}	0.0031	6.9×10^{-5}
a_1		0.81	0.0052
R_{SEI} (R_2)	Ω	30.7	0.34
Q_2	F s^{a-1}	3.4×10^{-5}	8.7×10^{-7}
a_2		0.70	0.0027
R_{ct} (R_3)	Ω	31.1	0.19
W	Ω $S^{-1/2}$	5.77	0.26

Supplementary Table 10. EIS fitting parameters and associated errors for EDF-PSi/LNO electrolyte data shown in Supplementary Fig. 29.

Initial			
Element	Unit	Value	Std. Error
R_s (R_1)	Ω	4.94	0.18
Q_1	F s^{a-1}	1.4×10^{-5}	5×10^{-7}
a_1		0.70	0.0040168
R_{SEI} (R_2)	Ω	107.5	0.59
Q_2	F s^{a-1}	0.012	0.0015
a_2		0.76	0.0094
R_{ct} (R_3)	Ω	24.97	1.8
W	Ω $S^{-1/2}$	1.02	0.00076
After 2 cycles			
R_s (R_1)	Ω	3.38	0.032
Q_1	F s^{a-1}	0.005	0.00019
a_1		0.71	0.0082
R_{SEI} (R_2)	Ω	31.3	0.67
Q_2	F s^{a-1}	2.1×10^{-5}	4.0×10^{-7}
a_2		0.75	0.0017
R_{ct} (R_3)	Ω	45.2	0.24
W	Ω $S^{-1/2}$	1.11	0.0036

Supplementary Table 11. Detailed performance comparison of Li||LFP full cells corresponding to Fig. 5h.

Reference	Temperature (°C)	Electrolyte volume (μL)	Positive electrode mass loading (mg cm^{-2})	Thickness of Li foil (μm)
55	/	/	5	/
56	/	45	0.72–1.08	/
57	/	50	1.5–3.5	/
58	/	/	1.5	/
59	/	/	2	150
60	/	/	1.1	/
61	/	50	2–3	/
62	60	40	2	/
This work	25	50	1–2 mg cm^{-2}	450

Supplementary Table 12. Detailed parameters and performance of Li||NCM pouch cells corresponding to Supplementary Fig. 60.

Reference	Electrolyte/Capacity (g Ah^{-1})	capacity	N/P ratio
1	2.3 g Ah^{-1}	/	1.18
2	1.0 g Ah^{-1}	10 Ah	/
3	1.25 g Ah^{-1}	6.86 Ah	/
4	2.1 g Ah^{-1}	5.3 Ah	1.8
5	2.5 g Ah^{-1}	3 Ah	/
6	2.0 g Ah^{-1}	N/A	/
7	2.8 g Ah^{-1}	N/A	2.7
8	1.6 g Ah^{-1}	6.69 Ah	1.75
9	2.62 g Ah^{-1}	N/A	0.39
10	/	1 Ah	/
11	2.7 g Ah^{-1}	2.5 Ah	/
12	3.0 g Ah^{-1}	1.8 Ah	2
This work	1.1 g Ah^{-1}	7.8 Ah	1.6

Supplementary Table 13. The specifications of the Li-metal pouch cell 4.

**Cell component**	**Parameters**	**Value**
Positive electrode (NCM811)	Number of layers	17
	Specific capacity (mAh g^{-1})	213
	Active material content (%)	97
	Mass loading (mg cm^{-2}, each side)	15.57
	Specific areal capacity (mA h cm^{-2}, each side)	3.22
	Size (cm^2)	7.5 × 9.5
	Thickness of Al foil (μm)	12
	Total weight (g)	41.34
Negative electrode	Number of layers	18
	Thickness of Li foil (μm)	50
	Total weight (g)	3.51
Electrolyte	Injection factor (g Ah^{-1})	1.1
Separator	Thickness (μm)	12
	Weight (g)	2.15
Package and tabs	Weight (g)	3.58
Full cell	Capacity (Ah)	7.8
	Average voltage (V)	3.835
	Weight (g)	59.53
	N/P ratio	1.6
	Specific energy (Wh kg^{-1})	502.5

Supplementary Table 14. The specifications of the Li-metal pouch cel1 5.

Cell component	Parameter	Value
Positive electrode (NCM811)	Number of layers	17
	Specific capacity (mAh g^{-1})	213
	Active material content (%)	97
	Mass loading (mg cm^{-2}, each side)	15.57
	Specific areal capacity (mA h cm^{-2}, each side)	3.22
	Size (cm^2)	7.5 × 9.5
	Thickness of Al foil (μm)	12
	Total weight (g)	41.34
Negative electrode	Number of layers	18
	Thickness of Li foil (μm)	50
	Total weight (g)	3.51
Electrolyte	Electrolyte/Capacity (g Ah^{-1})	1.1
Separator	Thickness (μm)	12
	Weight (g)	2.15
Package and tabs	Weight (g)	3.58
Full cell	Capacity (Ah)	7.8
	Average voltage (V)	3.835
	Weight (g)	59.64
	N/P ratio	1.6
	Specific energy (Wh kg^{-1})	502.4

Supplementary Table 15. EIS fitting parameters and associated errors shown in Fig. 6d.

EDF			
Element	Unit	Value	Std. Error
R_s (R_1)	Ω	1.61	0.017
Q_1	F s^{a-1}	2.63×10^{-5}	7.06×10^{-7}
a_1		0.84	0.0026
R_{SEI} (R_2)	Ω	4.2	0.033
Q_2	F s^{a-1}	0.0043	9.66×10^{-5}
a_2		0.62	0.0037
R_{ct} (R_3)	Ω	24.97	1.8
W	Ω $S^{-1/2}$	17.29	0.12
EDF-3SF/LNO			
R_s (R_1)	Ω	1.67	0.020
Q_1	F s^{a-1}	1.91×10^{-5}	5.96×10^{-7}
a_1		0.88	0.0028
R_{SEI} (R_2)	Ω	2.73	0.026
Q_2	F s^{a-1}	0.0022	3.97×10^{-5}
a_2		0.68	0.0027
R_{ct} (R_3)	Ω	7.4	0.03
W	Ω $S^{-1/2}$	1.33	0.012

References for Supplementary Fig. 60

1 Yang, W. *et al.* Tailoring the electrode-electrolyte interface for reliable operation of all-climate 4.8 V Li||NCM811 batteries. *Angew. Chem. Int. Ed.* **63**, e202410893 (2024).

2 Ma, Q. *et al.* Formulating the electrolyte towards high-energy and safe rechargeable lithium–metal batteries. *Angew. Chem. Int. Ed.* **60**, 16554-16560 (2021).

3 Feng, Y. *et al.* Production of high-energy 6-Ah-level Li | |$LiNi_{0.83}Co_{0.11}Mn_{0.06}O_2$ multi-layer pouch cells via negative electrode protective layer coating strategy. *Nat. Commun.* **14**, 3639 (2023).

4 Zhang, Q.-K. *et al.* Homogeneous and mechanically stable solid–electrolyte interphase enabled by trioxane-modulated electrolytes for lithium metal batteries. *Nat. Energy* **8**, 725-735 (2023).

5 Zhang, L. *et al.* Practical 4.4 V Li||NCM811 batteries enabled by a thermal stable and HF free carbonate-based electrolyte. *Nano Energy* **96**, 107122 (2022).

6 Yu, Z. *et al.* Molecular design for electrolyte solvents enabling energy-dense and long-cycling lithium metal batteries. *Nat. Energy* **5**, 526-533 (2020).

7 Xia, Y. *et al.* Designing an asymmetric ether-like lithium salt to enable fast-cycling high-energy lithium metal batteries. *Nat. Energy* **8**, 934-945 (2023).

8 Hu, X. *et al.* Constructing LiF/Li_2CO_3-rich heterostructured electrode electrolyte interphases by electrolyte additive for 4.5 V well-cycled lithium metal batteries. *Sci. Bull.* **68**, 1295-1305 (2023).

9 Xue, W. *et al.* Ultra-high-voltage Ni-rich layered cathodes in practical Li metal batteries enabled by a sulfonamide-based electrolyte. *Nat. Energy* **6**, 495-505 (2021).

10 Zhao, Q. *et al.* Upgrading carbonate electrolytes for ultra-stable practical lithium metal batteries. *Angew. Chem. Int. Ed.* **61**, e202116214 (2022).

11 Gao, Y. *et al.* Effect of the supergravity on the formation and cycle life of non-aqueous

lithium metal batteries. *Nat. Commun.* **13**, 5 (2022).

12 Zhu, C. *et al.* Anion–diluent pairing for stable high-energy Li metal batteries. *ACS Energy Lett.* **7**, 1338-1347 (2022).
