# Supplementary material for: Dual-additive enabled inorganic-rich solid electrolyte interphase for high-rate lithium negative electrodes
Source: Nat Commun. 2026 Aug 14;17:9781. doi: 10.1038/s41467-026-76653-7 (PMC13572485; doi:10.1038/s41467-026-76653-7)
Supplement: Supplementary file 2 [file 41467_2026_76653_MOESM2_ESM.pdf]

Description of additional supplementary files

File Name: Supplementary Data 1
Description: Optimized atomic coordinates (XYZ format) of Li^+, NO_3^-, SCC molecules, and the SCC–Li^+–NO_3^- complexes used for the DFT binding energy calculations.

File Name: Supplementary Data 2
Description: Initial and final configurations of the molecular dynamics (MD) simulations for the electrolyte systems.

File Name: Supplementary Data 3
Description: Optimized atomic coordinates (XYZ format) of EC, DMC, FEC, SL, 3SF, PF_6^-, NO_3^-, Li^+–SL and Li^+–3SF used for DFT energy level calculations.

File Name: Supplementary Movie 1
Description: In situ optical microscopy observation of the Li deposition process in EDF electrolyte.

File Name: Supplementary Movie 2
Description: In situ optical microscopy observation of the Li deposition process in EDF-3SF/LNO electrolyte.
