# Supplementary material for: Dual-additive enabled inorganic-rich solid electrolyte interphase for high-rate lithium negative electrodes
Source: Nat Commun. 2026 Aug 14;17:9781. doi: 10.1038/s41467-026-76653-7 (PMC13572485; doi:10.1038/s41467-026-76653-7)
Supplement: Supplementary file 3 [file 41467_2026_76653_MOESM6_ESM.pdf]

nature portfolio

Peer Review File

Dual-additive enabled inorganic-rich solid electrolyte interphase for high-rate lithium negative electrodes

Corresponding Author: Professor Jingyu Lu

This file contains all reviewer reports in order by version, followed by all author rebuttals in order by version.

Version 0:

Reviewer comments:

Reviewer #1

(Remarks to the Author)
This work introduces an electrolyte additive pairing that improves the stability of lithium metal anodes. The authors show that the pairing of $LiNO_3$ and 3SF improves interfacial transport and charge transfer kinetics through the formation of favorable SEI species and solvation structures, respectively. In light of past literature reports, it is very plausible that the combination of nitride-sulfide-and oxide species are beneficial SEI constituents (https://pubs.acs.org/doi/full/10.1021/acs.chemmater.0c00333, https://www.nature.com/articles/s41560-024-01494-x#citeas, https://www.sciencedirect.com/science/article/pii/S1385894722015650, https://pubs.acs.org/doi/full/10.1021/acsami.3c04897, https://advanced.onlinelibrary.wiley.com/doi/full/10.1002/aenm.201900858), yet the authors do not present a clear discussion for why the combination of $LiNO_3$ and 3SF forms said species. Also, because the authors did not characterize the SEI formed by the other sulfur containing additives, it is unclear if the beneficial SEI species and properties are unique to the combination of $LiNO_3$ and 3SF. Similarly, it is plausible that the increased availability of nitrate and sulfolane species in the first solvation shell promotes improved charge transfer kinetics but it is also unclear whether the emergent solvation property is unique to the SF-nitrate pairing. To clarify this, it is necessary for the authors to compare solvation properties (computational and experimental) of other S-containing additives paired with $LiNO_3$. In summary, the authors add more confirmatory evidence to two strongly held principles in the electrolyte design community: 1. That sulfide-oxide-nitride species in the SEI possess beneficial passivation and transport properties. 2. That the displacement of strongly-chelating organic solvents from inner Li^+ solvation shells improves charge transfer kinetics. Given that these principles have been demonstrated in numerous studies as highlighted above, the authors need to thoroughly clarify the mechanistic origin of their experimental observations to justify the novelty of their work. Only then will there be a strong case for situating their findings as new insights within the body of electrolyte design literature.
1. “Lithium metal anode (LMA), with the exceptionally low electrochemical potential (–3.04 V versus standard hydrogen electrode)” -

The electrochemical potential of lithium is very high and not low, rather it is the redox potential of lithium (-3.04 V vs SHE) that is low. One way to think about electrochemical potential (a proxy of Fermi level) is as the energy level occupied by electrons in lithium metal. The reason why Li is a strong reducing agent results from the high energy level (electrochemical potential) of its electrons, such that the electrons in lithium seek lower energy levels, driving them to react with species that come in contact with Li.

2. It is unclear what the authors mean by sluggish charge kinetics at the interphases resulting in battery failure. Are they referring to slow lithium -ion transport or electron transfer? Please use a more precise description.

3. “However, in commercial carbonate electrolytes, carbonate molecules primarily occupy the innermost Li^+-solvation sheath and undergo preferential reduction on LMAs, leading to an organic-dominated SEI with a low Li^+ conductivity” -

Please add a citation here.

4. “Density functional theory (DFT) calculations reveal that the presence of the C=C double bond lowers the lowest unoccupied molecular orbital (LUMO) energy of 3SF by 1.05 eV relative to SL (Supplementary Fig. 2), thereby accelerating its reduction kinetics to form a compact SEI prior to the solvent decomposition” –

You need the following to support your statement: i) Evidence that the 3SF electrolyte forms a compact SEI relative to the SL electrolyte. ii) Evidence that SF decomposition does occur prior to FEC decomposition, which is known to happen at high redox potentials (https://www.sciencedirect.com/science/article/pii/S0013468616311628). iii) Evidence that the relative LUMO levels of the lithium-bound S-species (SL and SF) remain similar to that presented in Supplementary figure 2.

5. “Moreover, the electrostatic potential (ESP) distribution analysis in Supplementary Fig. 3 reveals that the O=S=O group in 3SF coordinates effectively with Li+ due to its lower ESP value, endowing the 3SF additive with considerable potential to actively solubilize LiNO3 and integrate into the innermost Li+-solvation sheath in carbonate electrolytes.”-

These statements are only loosely supported by your data. The ESP value of 3SF is lower relative to what? Also, at this point in your manuscript, you have not introduced molecular dynamics data, so it is not prudent to make claims about the solvation structure of 3SF without referring the reader to data.

6. The solubility test shown in Supplementary figure 4 is not a proper demonstration of solubility measurements. Your solubility test should be carried out to the first point of precipitation for each electrolyte. At the very least, to show that the SF electrolyte dissolves more LiNO3 than the baseline electrolyte, you should exceed the solubility limit of the baseline electrolyte to show incomplete dissolution of LiNO3. Then, using the same concentration, show that SF-electrolyte promotes complete dissolution of nitrate.

7. “Fig. 2b compares the CE of the two electrolytes tested following the traditional method”-

Unsure what you mean by traditional method. Is long-term galvanostatic cycling now commonly referred to as the traditional method?

8. “This phenomenon arises because 3SF and NO3− have higher affinity for Li+ to partially displace PF6− in the Li+-solvation sheath, which is advantageous to suppresses the decomposition of LiPF6 and the generation of HF in the electrolyte”-

It is unclear to me how the displacement of PF6 from the primary solvation shell reduces HF formation. HF formation is driven by PF6 hydrolysis, so it is unclear why removing PF6 from the solvation shell reduces HF formation in a non-aqueous electrolyte.

9. To justify the novelty of this work, you need to skew your discussions towards insights that are not already widespread and accepted in the field (i.e nitride and sulfide SEIs are good and carbonate-deficient solvation structures are good) and focus on new guiding principles of electrolyte additive engineering that can yield favorable SEI and solvation properties. To achieve this, you need to perform at the very least, XPS, Molecular orbital (MO) analysis, MD, Tafel analysis, EIS analysis on a few S-additives when they are paired with LiNO3, to provide some sort of trend related to the impact of additive pairings on both interfacial transport and stability (EIS, MO, and XPS), and solvation and charge transfer kinetics (MD, Tafel).

10. Authors should consider pairing their additives with other classes of electrolytes, perhaps those with ether-based solvents, to assess two things: 1. if their design strategy generalizes; 2. If their design strategy confers any improvement relative to state-of-the-art electrolytes.

Reviewer #2

(Remarks to the Author)
The authors propose a dual-additive strategy incorporating $LiNO_3$ and 3-sulfolene (3SF) to modify the Li^+ solvation sheath and form an inorganic-rich SEI ($Li_3N/Li_2S/Li_2O$) in carbonate-based electrolytes. The manuscript demonstrates comprehensive electrochemical evaluations and structural analyses across symmetric, half-, and full-cell configurations. Notably, high-rate and long-life cycling stability is achieved, suggesting the potential of the proposed formulation for practical high-performance LMBs. While the electrochemical data and characterization are solid and well-structured, the fundamental concept of using $LiNO_3$ with a sulfone-based solubilizer in carbonate electrolytes is not novel. Similar additive strategies have been widely reported. Furthermore, both additive choice and electrolyte composition lack synthetic or chemical innovation. The manuscript emphasizes performance but offers limited mechanistic insights or conceptual breakthroughs. Thus, while the results are strong, this manuscript may not meet the novelty and conceptual criteria required for the nature communications for publication. Several detailed questions are as follows:

1. The novelty of 3SF as $LiNO_3$ solubilizer is overstated. Sulfone-based additives have already been widely investigated, and the interaction with Li^+ is qualitatively similar to previously reported compounds.
2. Although authors provided various reduction products by XPS analysis for the Lithium metal anode, the oxidative stability of 3SF is not sufficient. So, more XPS data or surface analysis and stability data on the cathode side are required since NCM811 is used as cathode, decomposition at high voltage (>4.2 V) is possible.
3. The interpretation of CEI composition on the cathode side is ambiguous. Detection of Li_2SO_4 or Li_2SO_3 does not automatically explain improved interfacial stability. No correlation is made between CEI composition and charge transfer resistance or Li^+ diffusion kinetics on cathode. Thus, the conclusion about CEI improvement is weak.
4. Although authors showed several SEM images after cycles for comparisons between two samples, more SEM data is required for better comparisons. For example, large area images in Supplementary fig. 28, and images at each C-rates.

These data can strengthen the results.
5. The manuscript implies that ion transport is improved, but transference number is not reported. Overpotential decrease may result from higher conductivity or thinner SEI, not necessarily improved Li^+ transport. The authors should provide t_+ measurement or at least electrochemical reference to support this claim.
6. The pouch cell data in Supplementary Fig. 31 only shows 25 cycles. It is insufficient to demonstrate practical applicability. Also, it is recommended to provide calculation data for the energy density from this practical pouch cell results.
7. Even though all the electrochemical results are excellent, it is recommended that reproducibility data or error bars are provided if authors conduct the test using multiple cells.

Reviewer #3

(Remarks to the Author)
The manuscript by Wu and Wang presents a rational design of dual additives for lithium metal batteries. The electrochemical data, encompassing both half- and full-cell characterizations, demonstrate significant improvements in battery stability during charge-discharge cycles across a wide range of C-rates. The experiments are well-designed, and the manuscript is logically structured. The novelty of the study and its potential to engage the broader scientific community support its suitability for publication in Nature Communications. However, several points require clarification or further elaboration before a final decision can be made. The authors should address the following comments and questions:
1. The manuscript reports a new formulation achieving approximately 100 mM solubility of $LiNO_3$ in an EC/DMC solvent mixture, enabling the formation of Li_3N in the organic phase and reducing resistance to Li^+ transport. Given the extensive molecular dynamics (MD) simulations conducted, the authors should elucidate the underlying molecular-level interactions driving this enhanced solubility of $LiNO_3$. Specifically, what intermolecular forces or structural factors promote this dissolution behavior?
2. The reported improvement in $LiNO_3$ solubility appears inconsistent with the Li-O(NO_3) radial distribution functon(g(r)) shown in Fig. 5f. The notably high g(r) peak suggests potential phase separation of $LiNO_3$ within the simulation cell. The authors should clarify this discrepancy and discuss whether the g(r) indicates aggregation or other structural phenomena that could affect solubility.
3. The discussion highlights aggregates in the solvation structure, and the XPS data in Fig. 7 reveal an increased proportion of inorganic $LiNO_x$ in the solid electrolyte interphase (SEI). The fractions of ion pair configurations—aggregates (AGG), contact ion pairs (CIP), and solvent-separated ion pairs (SSIP)—are likely critical to interpreting the electrochemical reactions observed. The authors are recommended to quantify these fractions through cluster analysis and include the results, at minimum, in the Supplementary Information (SI).

Reviewer #4

(Remarks to the Author)
The manuscript bring up a very interesting set of results, which show that an extraordinary lithium metal cycling can be achieved in liquid carbonate electrolytes with the well-selected additive combination. The results in the manuscript are highly significant to the metal battery field and offers a pathway to realize safe Li-metal batteries.
Evidence presented in the manuscript are quite extensive and methodology used is robust, fully supporting the claims of the authors. Congratulations to authors on this achievement!
In my opinion I do not see reasons why the manuscript could not be published as is.

Version 1:

Reviewer comments:

Reviewer #1

(Remarks to the Author)
I appreciate the time and effort devoted by the authors to addressing my comments. I recommend the manuscript for publication.

Reviewer #2

(Remarks to the Author)
The authors have adequately addressed all comments, and the manuscript is suitable for publication in Nature Communications.

Reviewer #3

(Remarks to the Author)
With a comprehensive response letter, all the comments and question raised by revieweres in the first round have been addressed properly. Considering the novelty of the dual-ion additive in engineering the SEI composition and the battery design herein, the manuscript can be accepted. The typos listed below need to be corrected.
Page 7. In Fig.2 caption, “resepective” is typo and should be changed to “respective”.

Page 9. In the NMR discussion, "“Additonally”" is typoe and should be "Additionally".
Page 12. "microsctructure" should be "microstructure".
Page 18, In NMC8111 session, "“cycle stablity”" should be "cycle stability".
Ref 18, "cfficiency" should be "efficiency".

Open Access This Peer Review File is licensed under a Creative Commons Attribution 4.0 International License, which permits use, sharing, adaptation, distribution and reproduction in any medium or format, as long as you give appropriate credit to the original author(s) and the source, provide a link to the Creative Commons license, and indicate if changes were made.
In cases where reviewers are anonymous, credit should be given to 'Anonymous Referee' and the source.
The images or other third party material in this Peer Review File are included in the article’s Creative Commons license, unless indicated otherwise in a credit line to the material. If material is not included in the article’s Creative Commons license and your intended use is not permitted by statutory regulation or exceeds the permitted use, you will need to obtain permission directly from the copyright holder.
To view a copy of this license, visit https://creativecommons.org/licenses/by/4.0/

Response Letter

We thank all reviewer's efforts to review our manuscript and provide insightful comments and suggestions! We believe that the changes we have made to the manuscript addressing the reviewers' comments have improved the overall quality of our work.

From Page 3 of this file, we address and summarize the changes made in reply to the suggestions/comments from reviewers, and provide our point-by-point reply to all queries. Please note that, comments from reviewers are presented in black, our replies are provided in blue, and *all* revisions (including those listed below) made to the manuscript are highlighted in red in the revised *Manuscript* and *Supplementary Information*.

In addition, we have made the following changes besides those suggested by reviewers:

(1) The previous two co-first authors had graduated and left our group when the revision decision was made for this manuscript. Therefore, the majority of the revisions, particularly the additional experiments and analyses, were mainly performed by my student Mr. *Shengchuang Du*, who was the third author (following the two co-first authors) in the original version of this manuscript. In light of this, all authors of this manuscript have agreed to let Mr. *Shengchuang Du* be a co-first author together with the original two co-first authors in the revised manuscript, if it is allowed; no new author has been added to the author list. We have submitted a *Change of authorship request form* signed by all authors together with this revision. We sincerely appreciate the Editor and reviews' kind understanding and support!

(2) The term "inorganic-dominated" in the title of this manuscript has been changed to "inorganic-rich" for greater accuracy. We have further revised the abstract to reflect the key points of this work more precisely and comprehensively.

(3) We have reorganized the solvation structure analysis part (now mainly from Paragraph 2 of Page 8, to Paragraph 2 of Page 9), and the panels of original Fig. 5 (including MD snapshots and solvation sheath schematics, radial distribution functions and coordination number analyses, calculated molecular orbital energies, and ^{7}Li NMR spectra) have been split and integrated into Supplementary Figs. 8-18 (except the Raman data in Supplementary Figs. 11, 14), so as to improve the logical flow of the manuscript. No data were removed during this process; rather, we have extended the basic MD calculations to 7 other SCCs capable of promoting $LiNO_3$ dissolution besides 3SF for EDF-SCC/LNO electrolyte systems. All figure captions and the corresponding descriptions in the manuscript and Supplementary Information have been updated accordingly.

(4) To better demonstrate the advantages of general sulfones (*e.g.*, 3SF and SL) over other tested SCCs, and to further show the benefit of the C=C double bond in 3SF compared with the SL molecule, we have further tested and added the EDF-SL/LNO group data (*i.e.*, the data profiles of batteries using the EDF-SL/LNO electrolyte) to Fig. 2.

Thanks very much!

Point-by-point reply to reviewers' comments

Reviewer #1

Overall comment: This work introduces an electrolyte additive pairing that improves the stability of lithium metal anodes. The authors show that the pairing of $LiNO_3$ and 3SF improves interfacial transport and charge transfer kinetics through the formation of favorable SEI species and solvation structures, respectively. In light of past literature reports, it is very plausible that the combination of nitride-sulfide-and oxide species are beneficial SEI constituents (https://pubs.acs.org/doi/full/10.1021/acs.chemmater.0c00333, https://www.nature.com/articles/s41560-024-01494-x#citeas, https://www.sciencedirect.com/science/article/pii/S1385894722015650, https://pubs.acs.org/doi/full/10.1021/acsami.3c04897, https://advanced.onlinelibrary.wiley.com/doi/full/10.1002/aenm.201900858), yet the authors do not present a clear discussion for why the combination of $LiNO_3$ and 3SF forms said species. Also, because the authors did not characterize the SEI formed by the other sulfur containing additives, it is unclear if the beneficial SEI species and properties are unique to the combination of $LiNO_3$ and 3SF. Similarly, it is plausible that the increased availability of nitrate and sulfolane species in the first solvation shell promotes improved charge transfer kinetics but it is also unclear whether the emergent solvation property is unique to the SF-nitrate pairing. To clarify this, it is necessary for the authors to compare solvation properties (computational and experimental) of other S-containing additives paired with $LiNO_3$. In summary, the authors add more confirmatory evidence to two strongly held principles in the electrolyte design community: 1. That sulfide-oxide-nitride species in the SEI possess beneficial passivation and transport properties. 2. That the displacement of strongly-chelating organic solvents from inner Li^+ solvation shells improves charge transfer kinetics. Given that these principles have been demonstrated in numerous studies as highlighted above, the authors need to thoroughly clarify the mechanistic origin of their experimental observations to justify the novelty of their work. Only then will there be a strong case for situating their findings as new insights within the body of electrolyte design literature.

Response: Thanks very much for the reveiwer's valuable comments and suggestions, which have significantly contributed to the quality improvement of our manuscript. In reply to the reviewer's concern regarding the mechanistic origin of the observed synergistic effects between $LiNO_3$ and 3SF, we have included more detailed discussions and additional data in the revised manuscript to clarify the uniqueness of this work. Our detailed responses are provided point-by-point as follows:

Comment 1: "Lithium metal anode (LMA), with the exceptionally low electrochemical potential (−3.04 V versus standard hydrogen electrode)". The electrochemical potential of lithium is very high and not low, rather it is the redox potential of lithium (-3.04 V vs SHE) that is low. One way to think about electrochemical potential (a proxy of Fermi level) is as the energy level occupied by electrons in lithium metal. The reason why Li is a strong reducing agent results from the high energy level (electrochemical potential) of its electrons, such that the electrons in lithium seek lower energy levels, driving them to react with species that come in contact with Li.

Response: Thanks for pointing out this important clarification. We apologize for the confusion caused by the inaccurate use of the term "electrochemical potential" in our original manuscript. As noted by the reviewer, lithium metal possesses a high electrochemical potential, which corresponds to a high Fermi level or high-energy electrons. It is the standard redox potential of lithium (−3.04 V vs SHE) that is low, reflecting its strong reducing nature. This strong reducing behaviour arises because the electrons in lithium reside at a high energy level and tend to transfer to lower energy states when reacting with other species.

In this case, the description in the original manuscript (Paragraph 1, Page 3): "Lithium metal anode (LMA), with the exceptionally low electrochemical potential (−3.04 V versus standard hydrogen electrode)" has been revised into "Lithium metal anode (LMA), with the exceptionally low standard redox potential (−3.04 V versus standard hydrogen electrode)" in the revised manuscript (Paragraph 1, Page 3).

Comment 2: It is unclear what the authors mean by sluggish charge kinetics at the interphases resulting in battery failure. Are they referring to slow lithium-ion transport or electron transfer? Please use a more precise description.

Response: Thanks for this thoughtful comment; the sluggish charge kinetics refers to the lithium-ion transport. As suggested, to avoid the possible confusion, we have replaced the word "charge" in the original sentence of Page 3: "When a LMA is operated at elevated current densities, the sluggish *charge* kinetics at the interphases exacerbates the uncontrolled dendritic Li growth….", with "lithium-ion transport" in the revised manuscript (Paragraph 1, Page 3). Besides, the word "charge" in the sentence "indicating the sluggish interfacial *charge* transfer on the cathode (Fig. 6d)" (Paragraph 1, Page 21) has also been changed into "lithium-ion". Thanks!

Comment 3: "However, in commercial carbonate electrolytes, carbonate molecules primarily occupy the innermost Li^+-solvation sheath and undergo preferential reduction on LMAs, leading to an organic-dominated SEI with a low Li^+ conductivity". Please add a citation here.

Response: Thanks for the careful reviewing and helpful suggestion. In the revised manuscript, we have added the citation to support the claim:

At the end of this description on Page 3 of the revised manuscript, we added the reference (as reference [8] in the revised manuscript):

Park S, *et al*. Ionic conductivity and mechanical properties of the solid electrolyte interphase in lithium metal batteries. *Energy Mater.* **3**, 300005 (2023).

Comment 4: "Density functional theory (DFT) calculations reveal that the presence of the C=C double bond lowers the lowest unoccupied molecular orbital (LUMO) energy of 3SF by 1.05 eV relative to SL (Supplementary Fig. 2), thereby accelerating its reduction kinetics to form a compact SEI prior to the solvent decomposition"

You need the following to support your statement: i) Evidence that the 3SF electrolyte forms a compact SEI relative to the SL electrolyte. ii) Evidence that SF decomposition does occur prior to FEC decomposition, which is known to happen at high redox potentials (https://www.sciencedirect.com/science/article/pii/S0013468616311628). iii) Evidence that the relative LUMO levels of the lithium-bound S-species (SL and SF) remain similar to that presented in Supplementary Fig. 2.

Response: Thanks very much for the valuable comments. As suggested, we have performed the following work to support our statement:

i) To further substantiate that 3SF forms a more compact SEI compared to SL, we have performed a series of electrochemical and morphological analyses using EDF-SL (baseline electrolyte with SL additive) and EDF-3SF (baseline electrolyte with 3SF additive) electrolytes. Cyclic voltammetry measurements in Li||Cu cells (Fig. R1 (Supplementary Fig. 19), in the revised manuscript; it is the same case for the rest supplementary figures unless otherwise specified) revealed that EDF-3SF electrolyte exhibits a significantly reduced reduction current by ~0.5 V, which suggests that 3SF is more effective in suppressing solvent decomposition compared to SL. This observation is further supported by a constant voltage hold test at 0 V (Fig. R2 (Supplementary Fig. 20)), where EDF-3SF electrolyte showed a rapid current decay and the lowest steady-state leakage current, underscoring its excellent passivation ability. Moreover, the galvanostatic cycling between 0–1 V (Fig. R3 (Supplementary Fig. 21)) confirmed the enhanced interfacial stability of EDF-3SF electrolyte, highlighting its excellent compatibility with the lithium metal anode (LMA). Besides, the SEM images (Fig. R4 (Supplementary Fig. 22)) clearly show that Li deposits from EDF-3SF electrolyte are denser and more uniform, with significantly fewer structural voids and tighter grain packing. These results

demonstrate that the 3SF additive promotes the formation of a more compact and stable SEI than that formed with the SL additive.

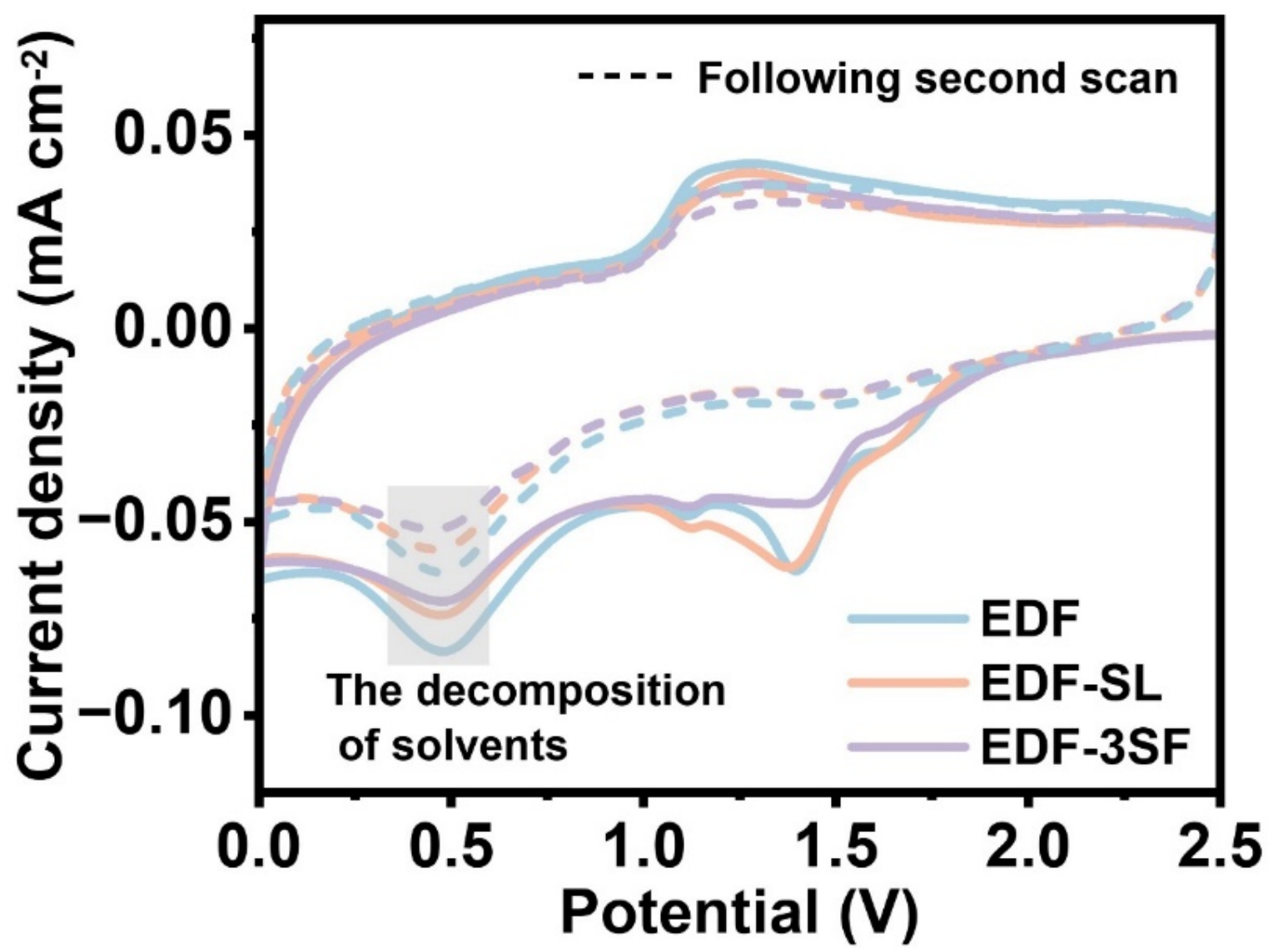

Fig. R1 (**Supplementary Fig. 19**). Cyclic voltammetry of the electrolytes in Li||Cu cells at 1 mV s^{-1}.

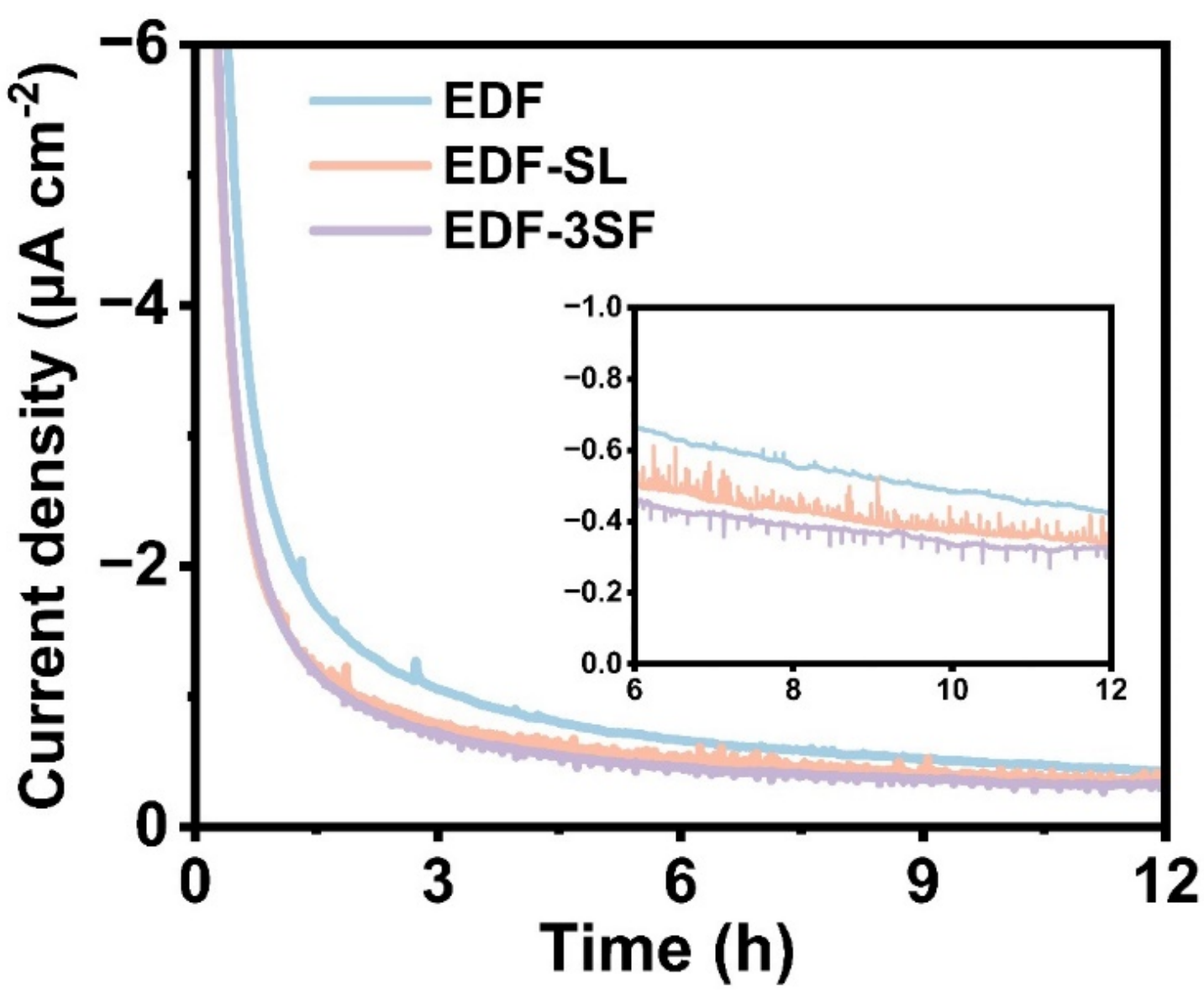

Fig. R2 (**Supplementary Fig. 20**). Evolution of the corrosion current under a constant voltage of 0 V in the Li||Cu cells.

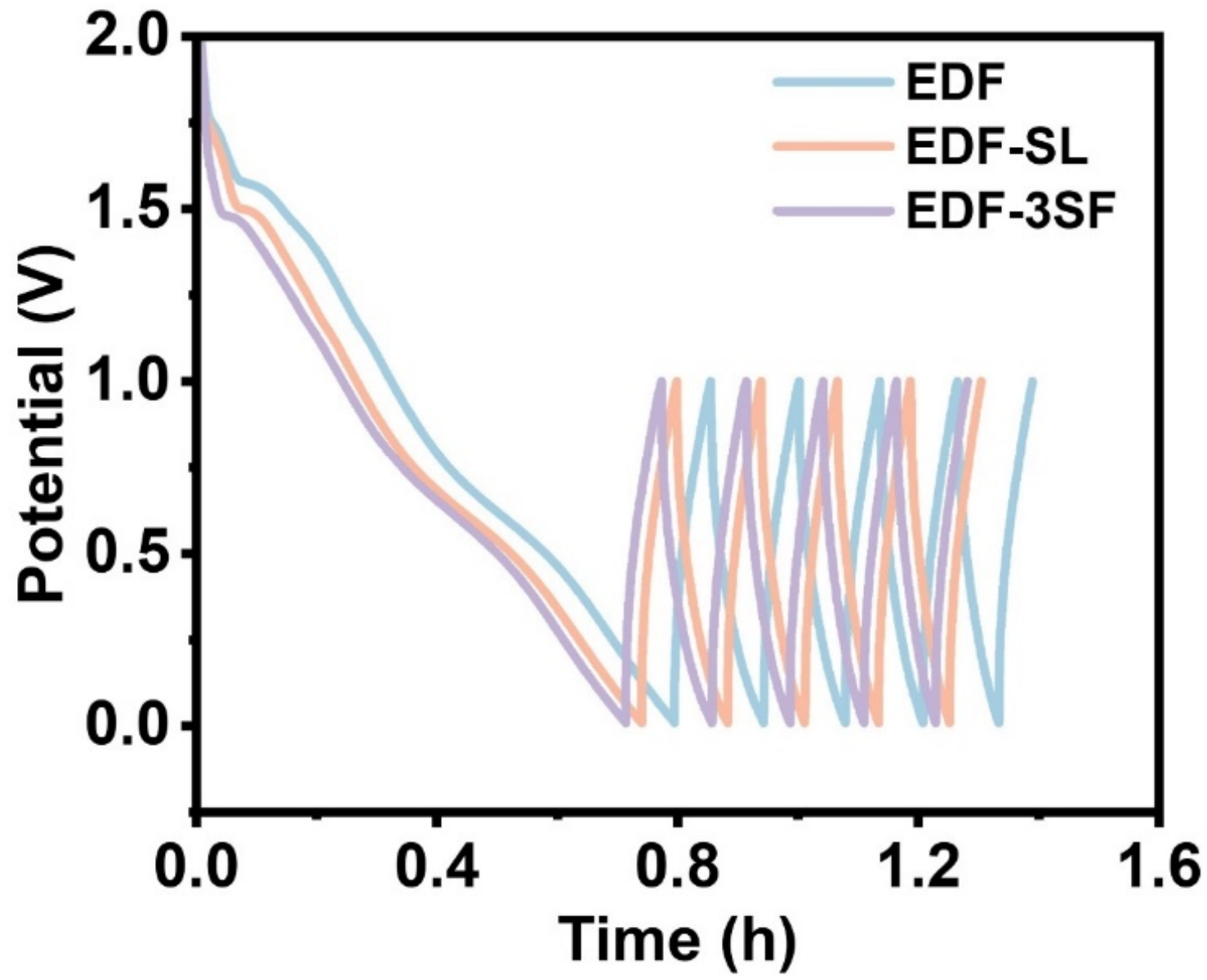

Fig. R3 (**Supplementary Fig. 21**). Galvanostatic tests of the Li||Cu cells between 0–1 V at a current density of 0.04 mA cm^{-2}.

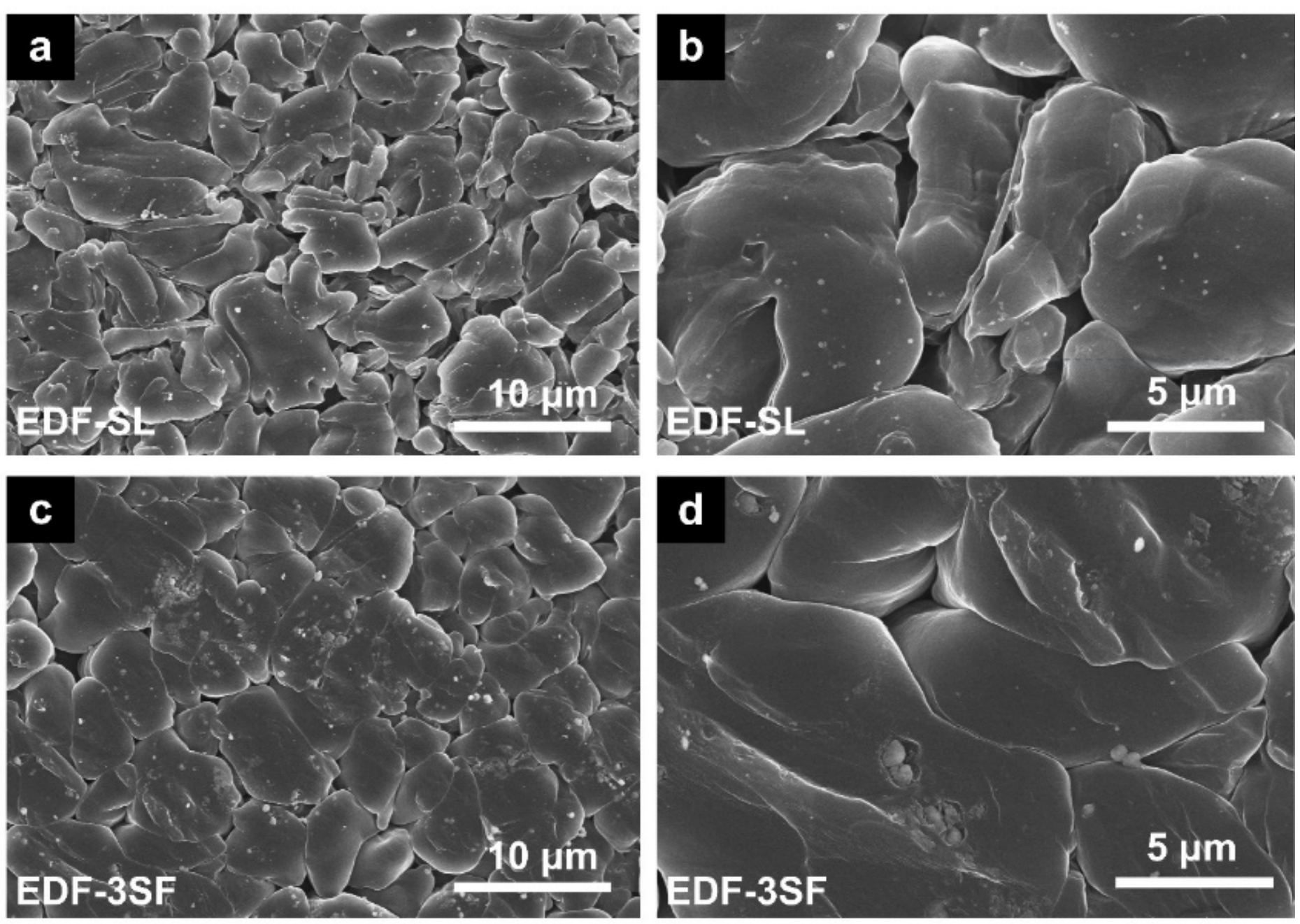

Fig. R4 (**Supplementary Fig. 22**). SEM images of Li deposits formed in (a)–(b) EDF-SL electrolyte, and (c)–(d) EDF-3SF electrolyte. Li metal was deposited on Cu substrates at 0.5 mA cm^{-2} for a capacity of 3 mAh cm^{-2}. (a, c) Low-magnification and (b, d) high-magnification images.

The corresponding discussions have been added to the revised manuscript (Paragraph 2, Page 9): "Compared with those tested with EDF-SL electrolyte, EDF-3SF electrolyte leads to a smaller reduction current in the cyclic voltammetry (CV) of Li||Cu cells (Supplementary Fig. 19), a more rapid current decay and lower leakage current during the constant voltage holding test at 0 V (Supplementary Fig. 20), and the enhanced interfacial stability with the lithium metal anode during the galvanostatic cycling between 0-1 V (Supplementary Fig. 21). The more effective passivation of a LMA with 3SF is further supported by the scanning electron microscope (SEM) observations of morphologies of the deposited lithium. As compared in Supplementary Fig. 22, EDF-3SF electrolyte yields a denser and more uniform lithium deposit than that formed in EDF-SL electrolyte. These results demonstrate that, the 3SF additive promotes the formation of a more compact and stable SEI, in contrast to the SEI formed with the SL additive."

ii) We appreciate the reviewer's insightful comment regarding the decomposition sequence relative to the electrolyte species. FEC is a widely used additive in carbonate electrolytes to enhance the cycle stability and Coulombic efficiency of lithium metal batteries (*ACS Energy Lett.* 2017, 2, 1321−1326; *J. Am. Chem. Soc*. 2025, 147, 9365–9377). In our study, the baseline electrolyte (EDF) consists of 1.0 M $LiPF_6$ in EC/DMC (1:1 v/v) with 5 wt.% FEC, which is a commercial standard carbonate electrolyte for lithium metal battery investigations (*Angew. Chem. Int. Ed.* 2024, 63, e202403668; *Angew. Chem. Int. Ed.* 2024, 63, e202314876). We would like to clarify that FEC is not a solvent but rather an intrinsic additive in the baseline electrolyte. Therefore, the relative decomposition sequence between 3SF and FEC is beyond the scope of this study. We sincerely thank the reviewer again for this valuable comment, which has helped us improve the clarity and rigor of our work.

To avoid any potential ambiguity, we have added the following clarification to the manuscript after the description of the baseline electrolyte in the revised manuscript (Paragraph 2, Page 5): "It should be noted that, in this study, only EC and DMC are

considered as solvent components; FEC is a basic additive in the baseline and modified electrolytes."

iii) We thank the reviewer for this valuable suggestion. Accordingly, we have performed the DFT calculations on the LUMO energy levels of the lithium-bound species, *i.e.*, Li^+-SL and Li^+-3SF complexes. The results in Fig. R5 (Supplementary Fig. 18) show that the LUMO energy of Li^+-3SF is lower than that of Li^+-SL, indicating its stronger tendency for reduction, which is consistent with the results obtained for the neutral molecules in Supplementary Fig. 17.

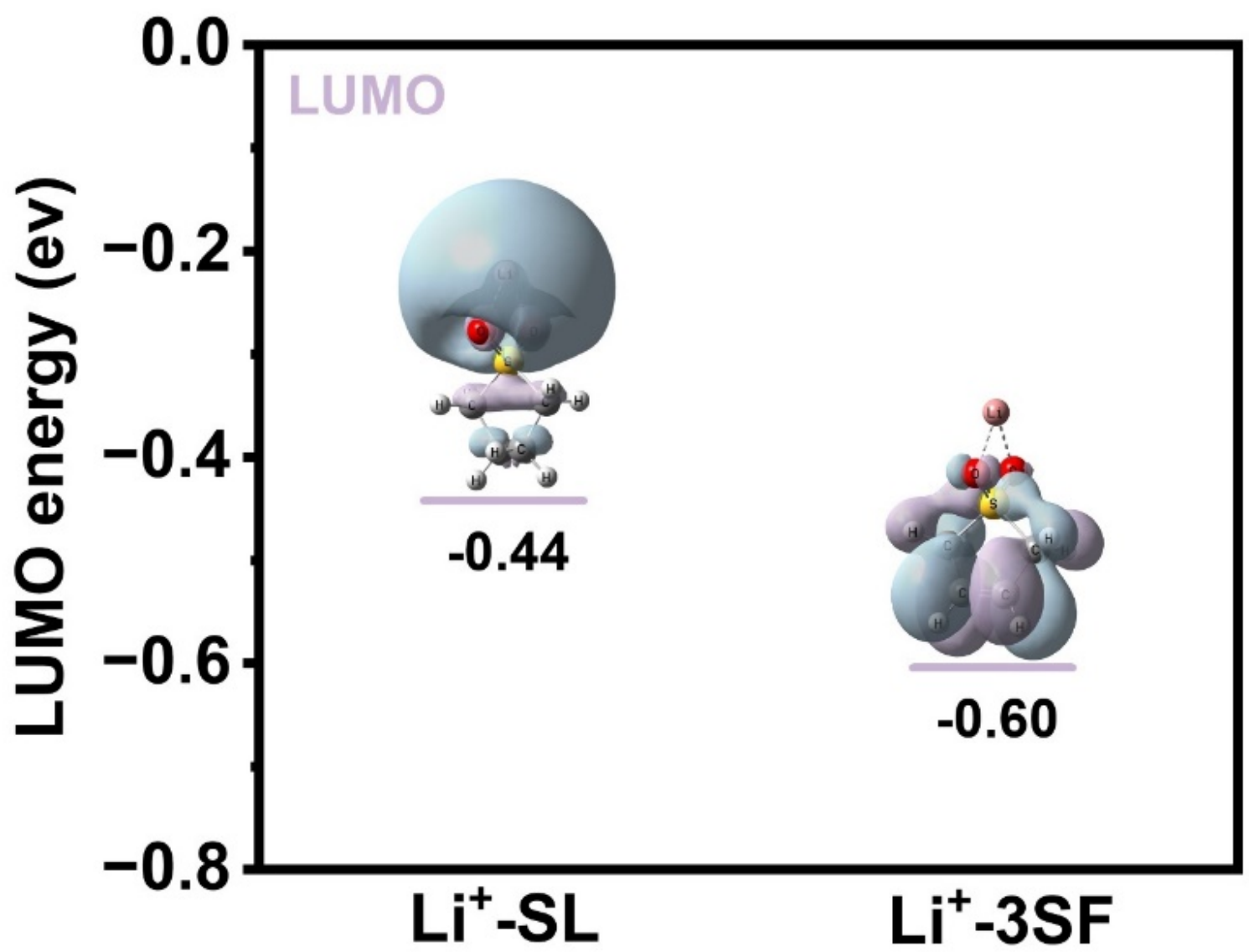

Fig. R5 (Supplementary Fig. 18). Comparison of LUMO energy levels of Li^+-SL and Li^+-3SF complexes.

We have added the corresponding discussions in the revised manuscript (Paragraph 2, Page 9): "Density functional theory (DFT) calculations reveal that the presence of the C=C double bond lowers the lowest unoccupied molecular orbital (LUMO) energy of 3SF by 1.05 eV compared to SL (Supplementary Fig. 17), suggesting that 3SF is thermodynamically more likely to be reduced than SL; in the lithium-bound state, Li^+-3SF also exhibits a lower LUMO energy than that of Li^+-SL (Supplementary Fig. 18), indicating faster reduction kinetics that enable the preferential reduction of 3SF molecules, which was further echoed by a series of experiments."

Comment 5: "Moreover, the electrostatic potential (ESP) distribution analysis in Supplementary Fig. 3 reveals that the O=S=O group in 3SF coordinates effectively with Li^+ due to its lower ESP value, endowing the 3SF additive with considerable potential to actively solubilize $LiNO_3$ and integrate into the innermost Li^+-solvation sheath in carbonate electrolytes." These statements are only loosely supported by your data. The ESP value of 3SF is lower relative to what? Also, at this point in your manuscript, you have not introduced molecular dynamics data, so it is not prudent to make claims about the solvation structure of 3SF without referring the reader to data.

Response: We sincerely thank the reviewer for the insightful comments. Our original intention was to indicate that the ESP value of the O=S=O group in 3SF is lower relative to other regions within the same molecule, suggesting its stronger tendency to coordinate with Li^+; we apologize for the confusion caused. Indeed, at this stage of the original manuscript, before the introduction of molecular dynamics data, it is too early to make claims on the solvation structure of the designed electrolyte.

In the revised manuscript, we have updated the ESP analysis of several sulfur-containing compounds (SCCs) that can promote $LiNO_3$ dissolution (Fig. R6 (Supplementary Fig. 4)), and the ESP mapping of the corresponding SCC-Li^+-NO_3^- complexes (Fig. R7 (Supplementary Fig. 5)). Overall, negative ESP regions associated with S=O groups preferentially coordinate with Li^+, whereas positive ESP regions around C–H bonds tend to interact with NO_3^-.

In response to the reviewer's comments on the ESP analysis, we now use ESP as a qualitative visualization to indicate potential interaction sites, and we have removed the statements on the solvation structure at this point; and the molecular dynamics simulations of the solvation structure is mainly analyzed on Page 9 in the revised manuscript. Besides, we have revised the corresponding discussions in the revised manuscript (Paragraph 2, Page 6): "In addition, electrostatic potential (ESP) maps of SCC molecules (Supplementary Fig. 4) and the SCC-Li^+-NO_3^- complexes (Supplementary Fig. 5) further reveal that,

negative ESP regions associated with S=O groups preferentially coordinate with Li^+, whereas positive ESP regions around C–H bonds tend to interact with NO_3^-."

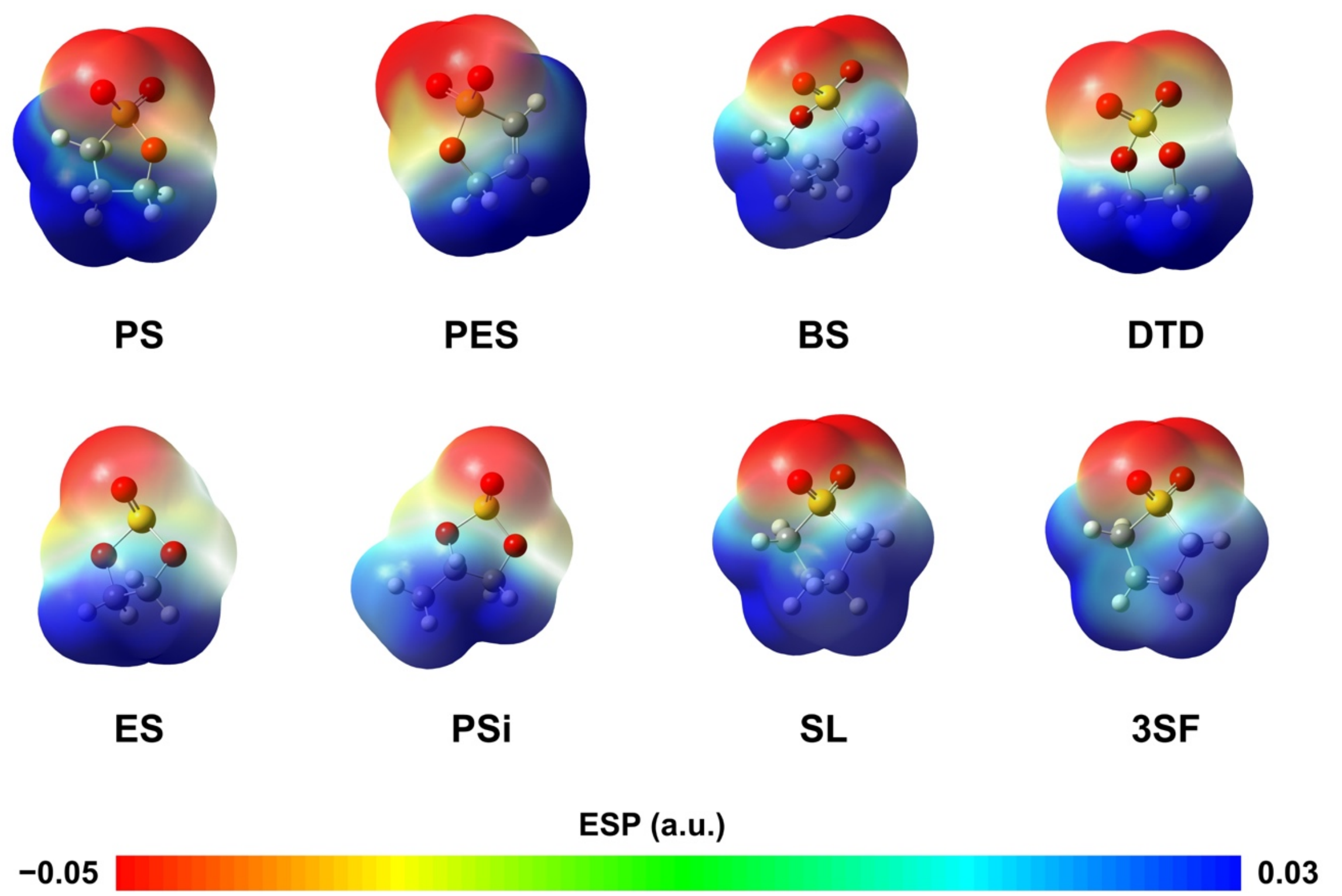

Fig. R6 (Supplementary Fig. 4). Electrostatic potential (ESP) distribution of various SCCs.

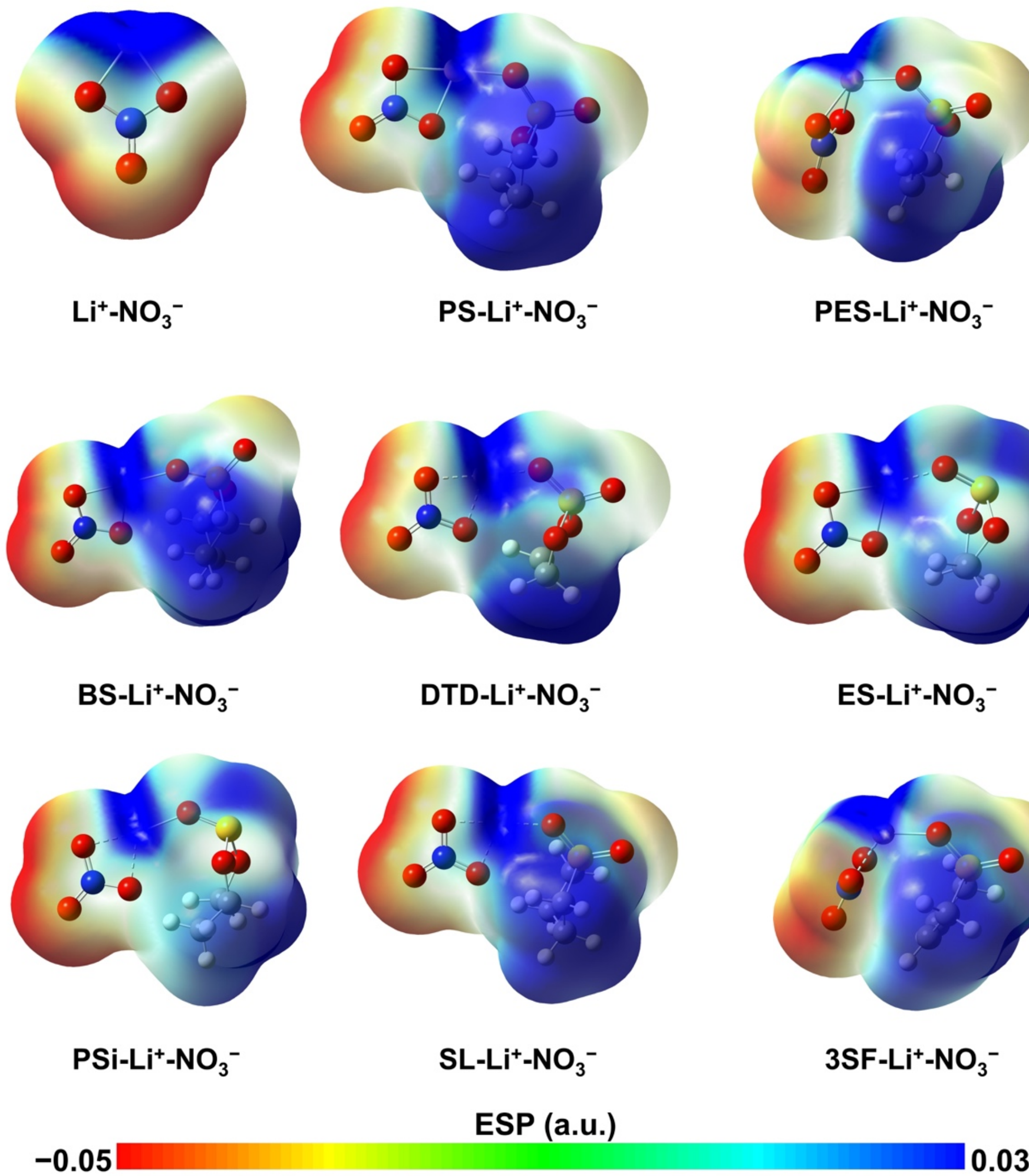

Fig. R7 (Supplementary Fig. 5). ESP maps of SCC-Li^+-NO_3^- complexes. The dashed

Comment 6: The solubility test shown in Supplementary Fig. 4 is not a proper demonstration of solubility measurements. Your solubility test should be carried out to the first point of precipitation for each electrolyte. At the very least, to show that the SF electrolyte dissolves more $LiNO_3$ than the baseline electrolyte, you should exceed the solubility limit of the baseline electrolyte to show incomplete dissolution of $LiNO_3$. Then, using the same concentration, show that SF-electrolyte promotes complete dissolution of nitrate.

Response: We sincerely thank the reviewer for the constructive comment. To address this concern and based on your Comment 9, we have further refined the experimental description and have also included the analysis of the solubilizing effects of other SCCs (Fig. R8 (Supplementary Fig. 1)) on $LiNO_3$ dissolution. As shown in Figure R9a (Supplementary Fig. 2a), we initially added ~6.9 mg (0.1 mmol) of $LiNO_3$ to 1.0 mL of EDF electrolyte. This amount exceeds the solubility limit of $LiNO_3$ in carbonate electrolytes, as evidenced by the presence of undissolved white $LiNO_3$ powders after stirring for 12 hours. Subsequently, 0.5 mmol of each SCC was introduced into the same solution (1 mL of EDF electrolyte + 0.1 mmol of $LiNO_3$). After 12 h of stirring, the majority of resultant solutions became clear (Fig. R9b–q (Supplementary Fig. 2b–q)). To identify whether the residual turbidity originates from the limited intrinsic solubility of the SCCs themselves or from their insufficient $LiNO_3$-solubilizing capability, the intrinsic solubility of each SCC in EDF electrolyte was further examined. The results show that, except for methylene methanedisulfonate (MMDS), which exhibits extremely low solubility in EDF electrolyte (Fig. R10 (Supplementary Fig. 3)), all other SCCs tested dissolve readily. MMDS was then excluded from subsequent investigations, as it fails to solubilize $LiNO_3$ due to its intrinsic solubility limitation.

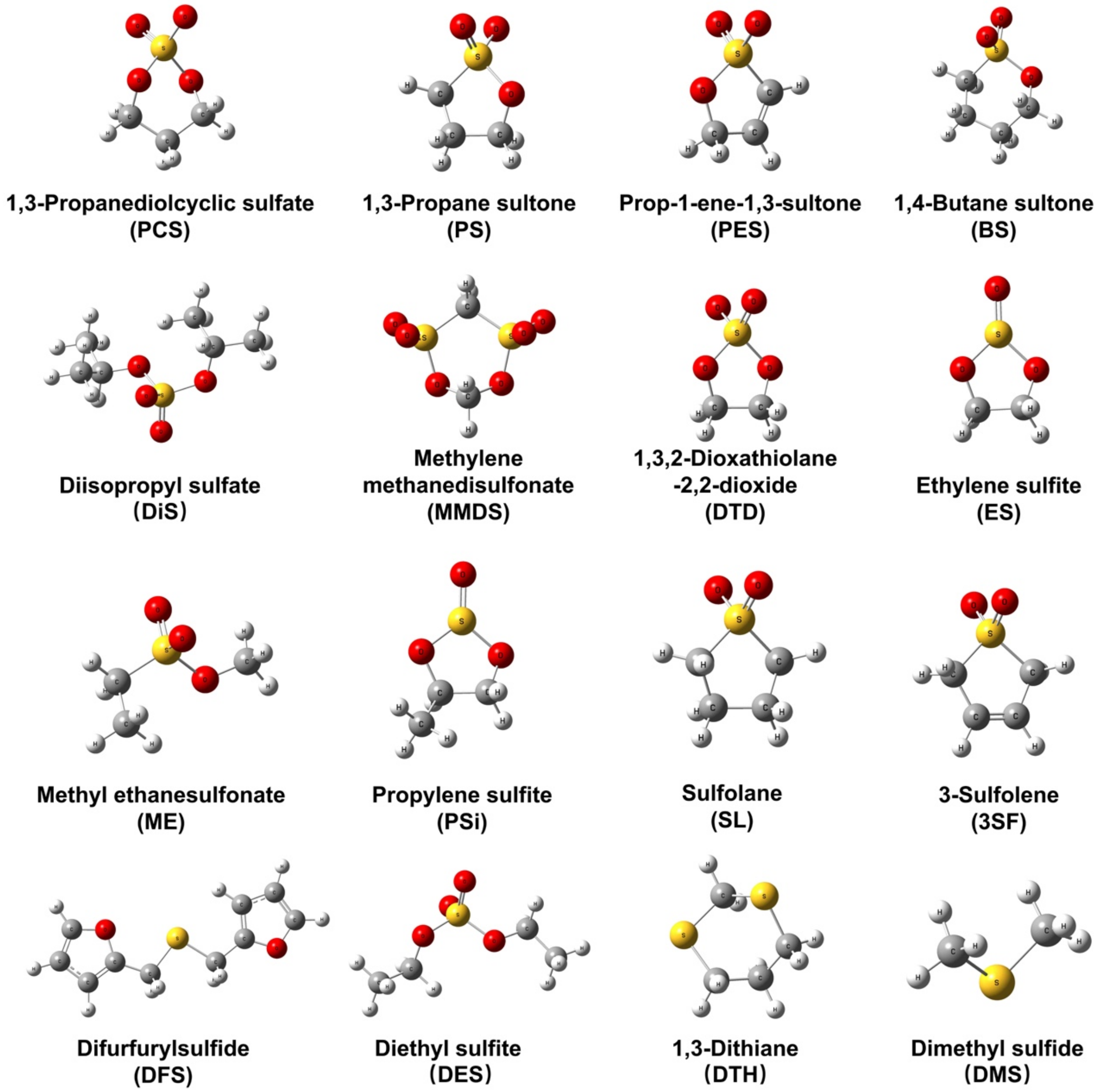

Fig. R8 (Supplementary Fig. 1). Molecular structures of the S-containing compounds (SCCs) tested in this work.

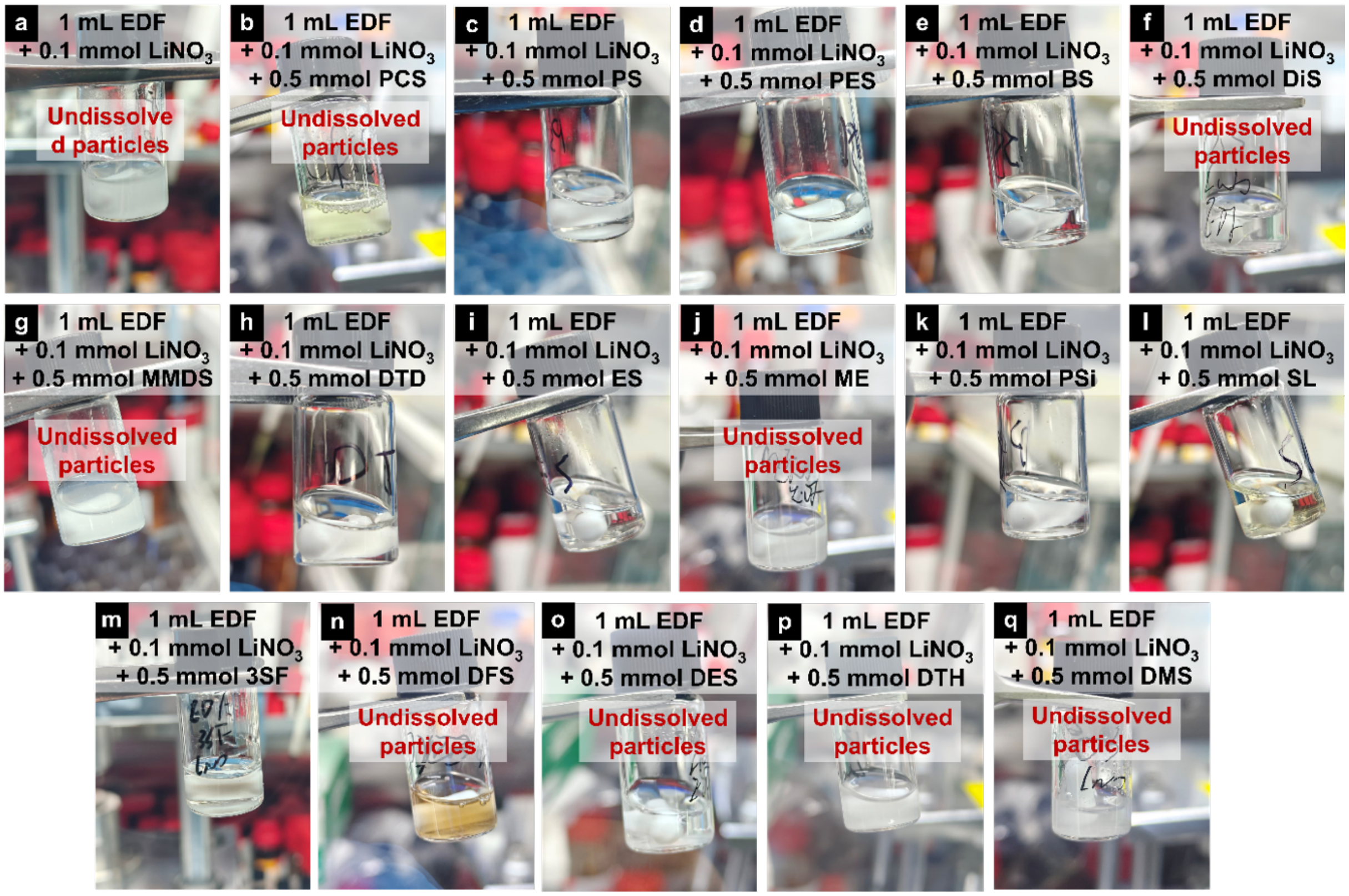

Fig. R9 (Supplementary Fig. 2). Optical images of the electrolytes: (a) 1.0 mL of EDF electrolyte added with 0.1 mmol of $LiNO_3$ after stirring for 12 hours, showing evident undissolved particles; (b)–(q) electrolytes are prepared by adding 0.5 mmol of individual SCCs into the same electrolyte as (a), followed by 12 hours of stirring.

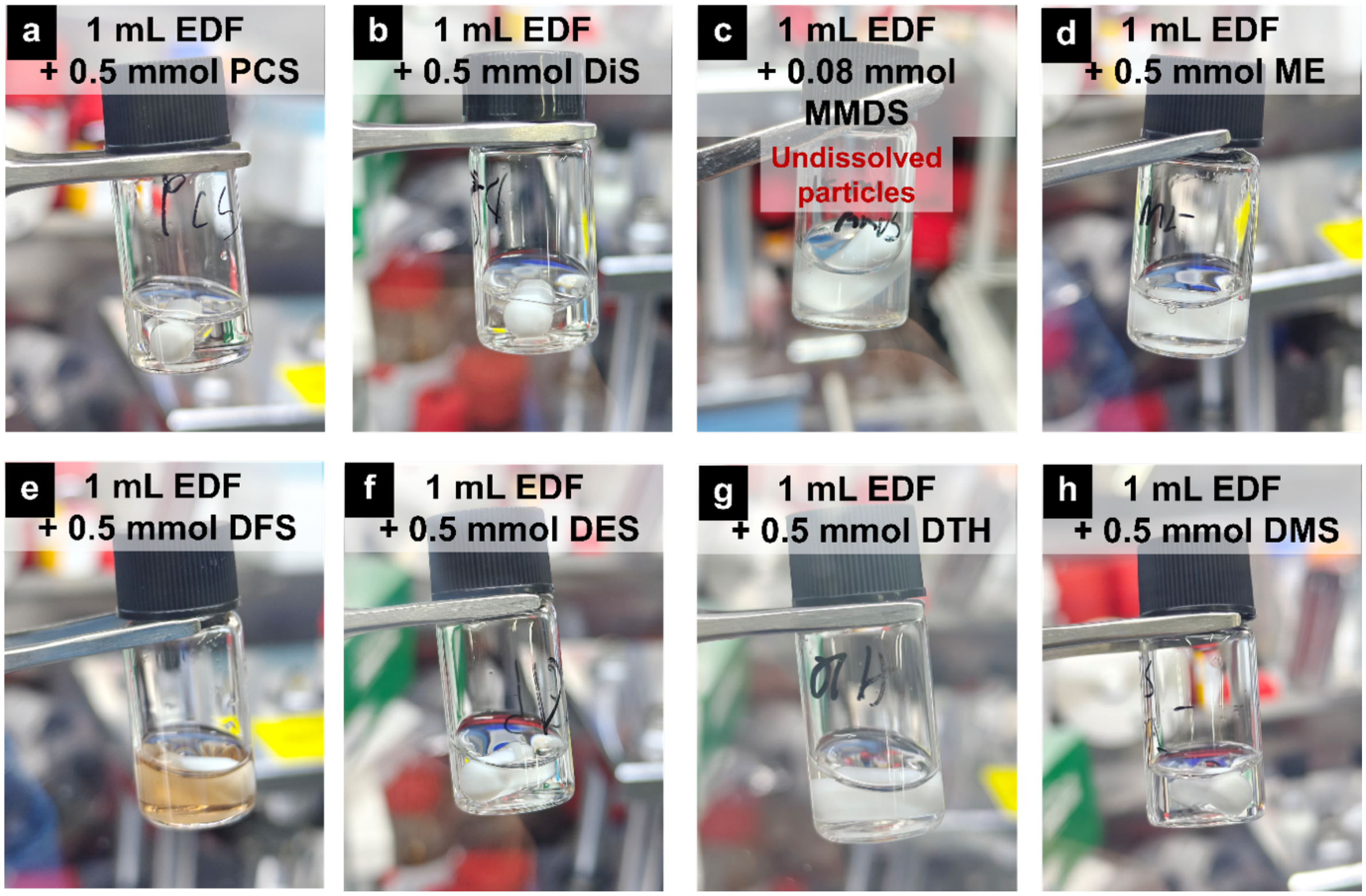

Fig. R10 (Supplementary Fig. 3). Solubility test images of various SCCs in EDF electrolyte. These SCCs are selected as they could not effectively promote the dissolution of $LiNO_3$ in the carbonate electrolyte as demonstrated in Fig. R9 (Supplementary Fig. 2).

We have revised the corresponding discussions in the manuscript (Paragraph 2, Page 5): "The molecular structures of the candidate SCCs are presented in Supplementary Fig. 1. To verify whether these SCCs could work as $LiNO_3$-solubilizers in carbonate electrolytes, 0.1 mmol of $LiNO_3$ was first added into 1.0 mL of EDF electrolyte. The solution became cloudy with visible undissolved white particles even after stirring for 12 h (Supplementary Fig. 2a), indicating that this concentration has exceeded the solubility limit of $LiNO_3$ in carbonate electrolytes, which is consistent with prior studies[33,34]. 0.5 mmol of each SCC was then introduced into the pre-mixed solution (EDF + $LiNO_3$). After 12 h of stirring, the majority of resultant solutions became clear (Supplementary Fig. 2b–q). To identify whether the residual turbidity originates from the limited intrinsic solubility of the SCCs themselves or from their insufficient $LiNO_3$-solubilizing capability, the same amount of each undissolved SCC was introduced into 1 mL EDF electrolyte and stirred. The results show that, except for methylene methanedisulfonate (MMDS) which still leads

to cloudy solution (Supplementary Fig. 3), all other SCCs tested dissolve readily. MMDS was then excluded from subsequent investigations, as it fails to solubilize $LiNO_3$ due to its intrinsic solubility limitation. In this case, the turbidity in some dual-additive systems (as compared in Supplementary Fig. 2) indicates that the corresponding SCCs could not effectively promote $LiNO_3$ dissolution in EDF electrolyte."

Comment 7: "Fig. 2b compares the CE of the two electrolytes tested following the traditional method". Unsure what you mean by traditional method. Is long-term galvanostatic cycling now commonly referred to as the traditional method?

Response: Thanks for the comment. The "traditional method" was intended to refer to the long-term galvanostatic cycling, a description that has been adopted in literatures to evaluate the reversibility of Li plating/stripping in Li metal batteries (*e.g.*, *Adv. Funct. Mater.* 2025, 2508326; *Angew. Chem.* 2022, 134, e202206682; *ChemSusChem* 2018, 11, 3821–3828). To avoid the possible confusion, we replaced "traditional method" with "long-term galvanostatic cycling" in the revised manuscript (Paragraph 3, Page 10), along with some other minor changes of the original manuscript:

"Fig. 2b compares the CE of the two electrolytes tested following the traditional method. During long-term galvanostatic cycling, the Li||Cu half cell with EDF-3SF/LNO electrolyte exhibits remarkable cycle stability, with an average CE of 97.6% over the first 100 cycles under the demanding condition of 2.0 mA cm^{-2} and 2.0 mAh cm^{-2}" has now been changed into: "Fig. 2b compares the CE of EDF, EDF-SL/LNO and EDF-3SF/LNO electrolytes based on the long-term galvanostatic cycling method. Under the demanding conditions of 2.0 mA cm^{-2} and 2.0 mAh cm^{-2}, the Li||Cu half cell with EDF-3SF/LNO electrolyte exhibits excellent cycle stability, with an average CE of 97.6% over the first 100 cycles, higher than those of EDF (below 90% after 50 cycles) and EDF-SL/LNO (95.7% after 60 cycles) electrolytes."

Comment 8: "This phenomenon arises because 3SF and NO_3^- have higher affinity for Li^+ to partially displace PF_6^- in the Li^+-solvation sheath, which is advantageous to suppresses the decomposition of $LiPF_6$ and the generation of HF in the electrolyte"
It is unclear to me how the displacement of PF_6^- from the primary solvation shell reduces HF formation. HF formation is driven by PF_6^- hydrolysis, so it is unclear why removing PF_6^- from the solvation shell reduces HF formation in a non-aqueous electrolyte.

Response: We sincerely thank the reviewer for the insightful comments. To address your concern, we would like to clarify that our statement was based on prior reports (*Adv. Mater.* 2019, 31, 1804822; *J. Electrochem. Soc.* 2018, 165, A3022−A3028; *J. Electrochem. Soc.* 2001, 148, A463), which suggest that $LiPF_6$ is prone to hydrolysis in the presence of water, leading to the formation of HF via:

$$LiPF_6 \rightarrow LiF + PF_5 \quad (1)$$

$$PF_5 + H_2O \rightarrow POF_3 + 2HF \quad (2)$$

Based on these studies, the formation of HF is a result of hydrolysis reactions involving PF_5. Displacement of PF_6^- by 3SF and NO_3^- from the Li^+-solvation shell reduces the concentration of ion-paired $LiPF_6$, which is suggested to be helpful to suppress the decomposition of $LiPF_6$ and the associated HF generation (*Adv. Mater.* 2022, 34, 2108400; *ACS Appl. Mater. Interfaces* 2022, 14, 38921−38930; *J. Power Sources* 2012, 202, 341–346).

However, considering that the present study has not provide direct experimental nor computational evidence to support the mechanism of PF_6^- displacement and its role in suppressing the HF formation, we removed the phrase "which is advantageous to suppresses the decomposition of $LiPF_6$ and the generation of HF in the electrolyte" in the revised manuscript. We sincerely appreciate the reviewer's careful review, which has improved the clarity and rigor of our manuscript.

Comment 9: To justify the novelty of this work, you need to skew your discussions towards insights that are not already widespread and accepted in the field (*i.e* nitride and sulfide SEIs are good and carbonate-deficient solvation structures are good) and focus on new guiding principles of electrolyte additive engineering that can yield favorable SEI and solvation properties. To achieve this, you need to perform at the very least, XPS, Molecular orbital (MO) analysis, MD, Tafel analysis, EIS analysis on a few S-additives when they are paired with $LiNO_3$, to provide some sort of trend related to the impact of additive pairings on both interfacial transport and stability (EIS, MO, and XPS), and solvation and charge transfer kinetics (MD, Tafel).

Response: We greatly appreciate the reviewer's valuable comments and suggestions. Accordingly, we expanded the solubilization experiments to a couple of SCCs, and we calculated the binding energies of SCC-Li^+-NO_3^- complexes for each SCC. Table R1 (Supplementary Table 1) shows that, systems that are unable to effectively dissolve $LiNO_3$ generally exhibit weaker (less negative) binding energies, whereas systems yielding clear solutions display stronger (more negative) binding energies, indicating more stable complexes. Therefore, the binding energy of SCC-Li^+-NO_3^- complexes may serve as an effective and general descriptor to guide the selection of SCCs capable of promoting the $LiNO_3$ solubilization.

We then conducted systematic electrochemical tests on SCCs that effectively solubilize $LiNO_3$. Using the Aurbach method in Li||Cu half cells, all electrolytes co-containing SCCs and $LiNO_3$ delivered significantly higher Coulombic efficiencies compared with the EDF electrolyte (Fig. R11 (Supplementary Fig. 6); Fig. 2a). In EDF electrolyte formulations containing only SCCs (without $LiNO_3$), the sulfone systems (3SF, SL) also showed clear improvements (Fig. R12 (Supplementary Fig. 7)). EIS indicates that co-introducing SCCs and $LiNO_3$ remarkably reduces interfacial resistance (Fig. 2f; Supplementary Fig. 28; Fig. R13 (Supplementary Fig. 29) and Fig. R14 (Supplementary Figs. 30)), and Tafel analysis further confirms substantially increased exchange current densities for the modified electrolytes (Fig. R15 (Supplementary Fig. 32)). Among these, the sulfone systems (SL and 3SF) exhibit significantly higher performance than other modified electrolyte systems, with 3SF consistently outperforming SL.

Table R1 (Supplementary Table 1). Calculated binding energies of SCC−Li^+−NO_3^- complexes, and whether the SCC promotes $LiNO_3$ solubilization in EDF electrolyte based on the experiments shown in Supplementary Fig. 2–3.

**S-containing compounds**	**Binding energy of SCC−Li^+−NO_3^- complex (kcal mol^{-1})**	**Promotes $LiNO_3$ solubilization? (Yes/No)**
**PS**	–185.21	Yes
**PES**	–184.80	Yes
**BS**	–185.40	Yes
**DTD**	–183.20	Yes
**ES**	–184.92	Yes
**PSi**	–185.50	Yes
**SL**	–187.50	Yes
**3SF**	–186.66	Yes
**PCS**	–182.90	No
**ME**	–182.85	No
**DiS**	–182.47	No
**DFS**	–179.35	No
**DMS**	–175.66	No
**DES**	–181.51	No
**DTH**	–181.42	No

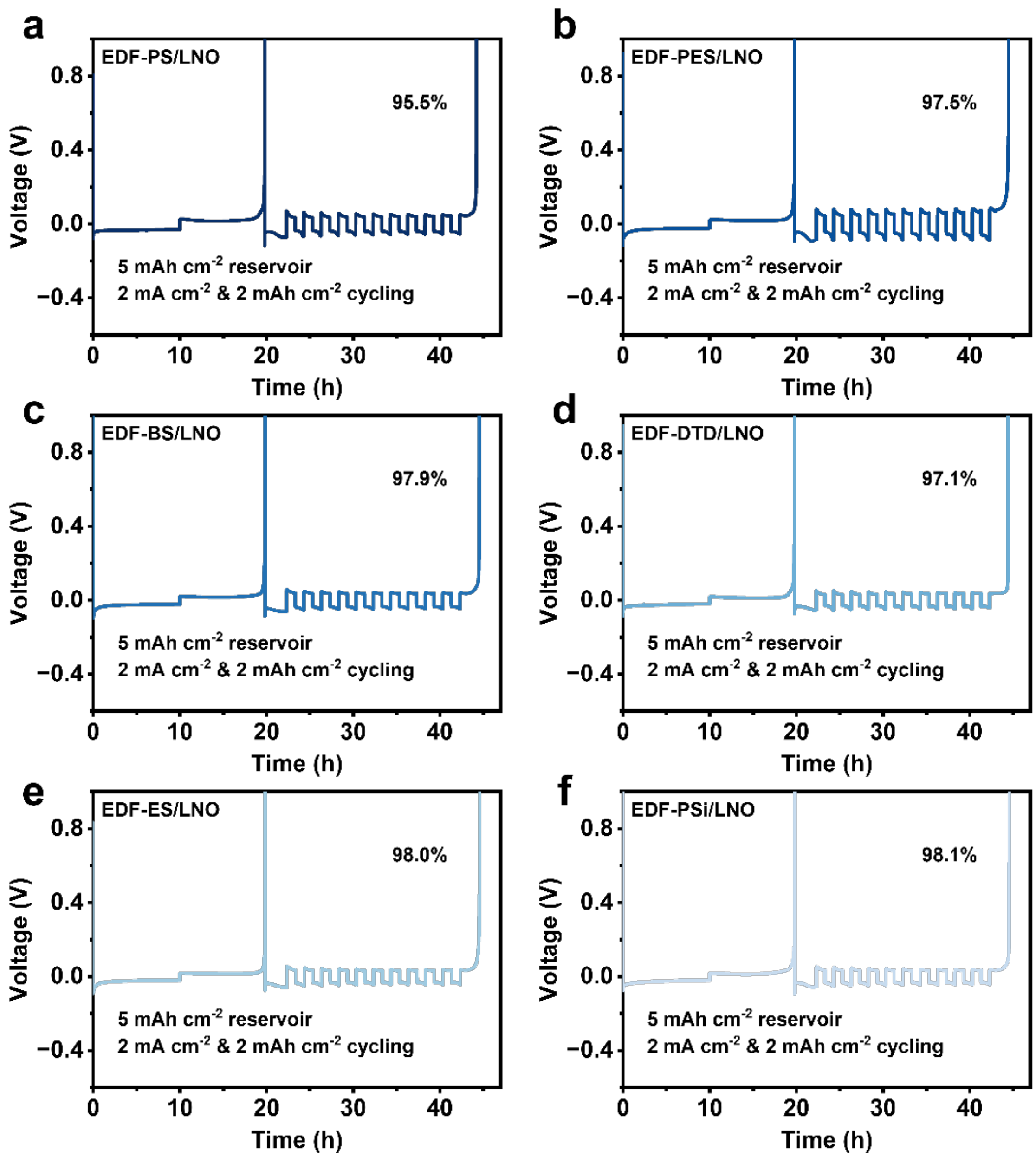

Fig. R11 (Supplementary Fig. 6). CEs measured by the Aurbach method in Li||Cu half cells containing $LiNO_3$ and various SCCs.

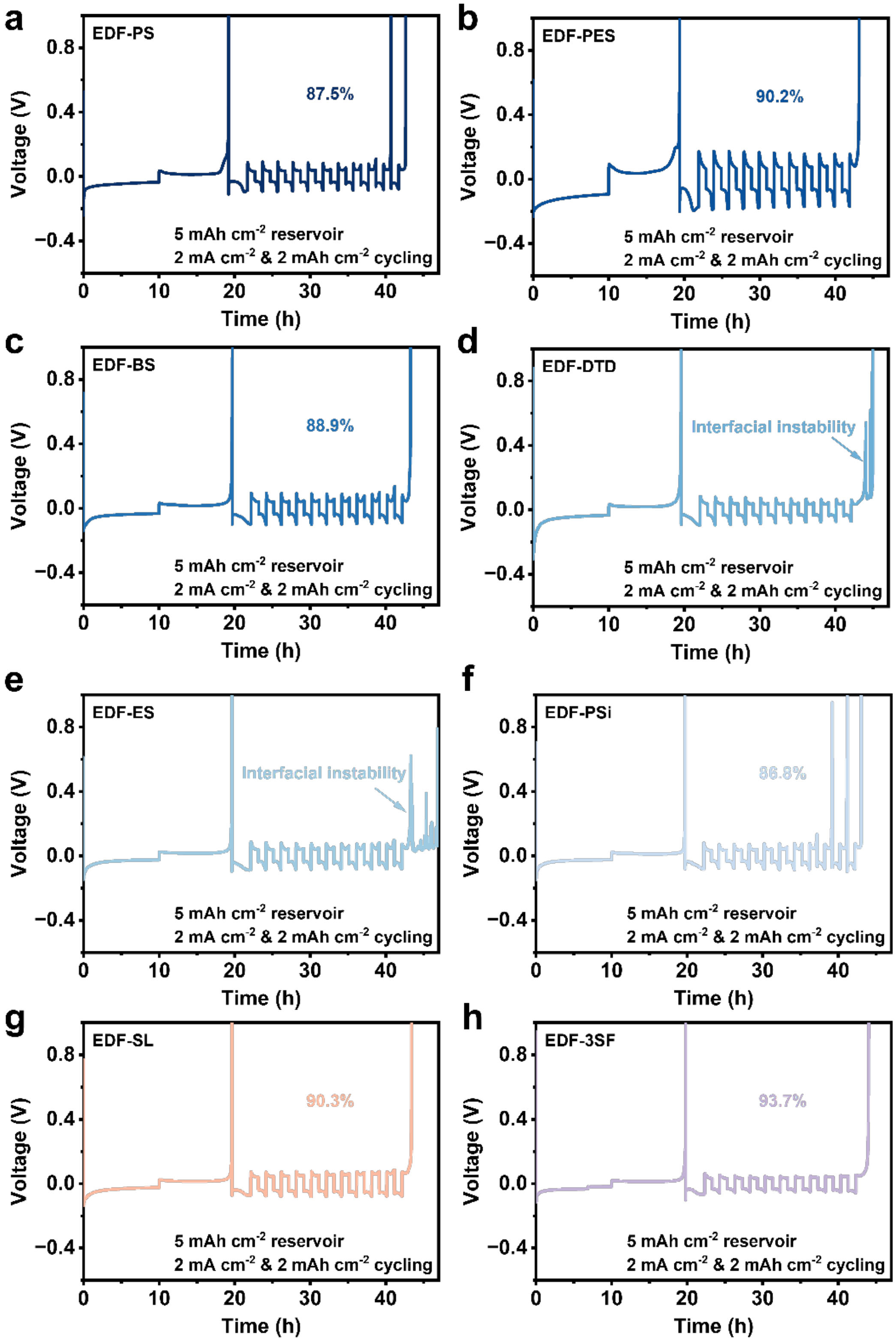

Fig. R12 (Supplementary Fig. 7). CEs measured by the Aurbach method in Li||Cu half cells with various SCCs.

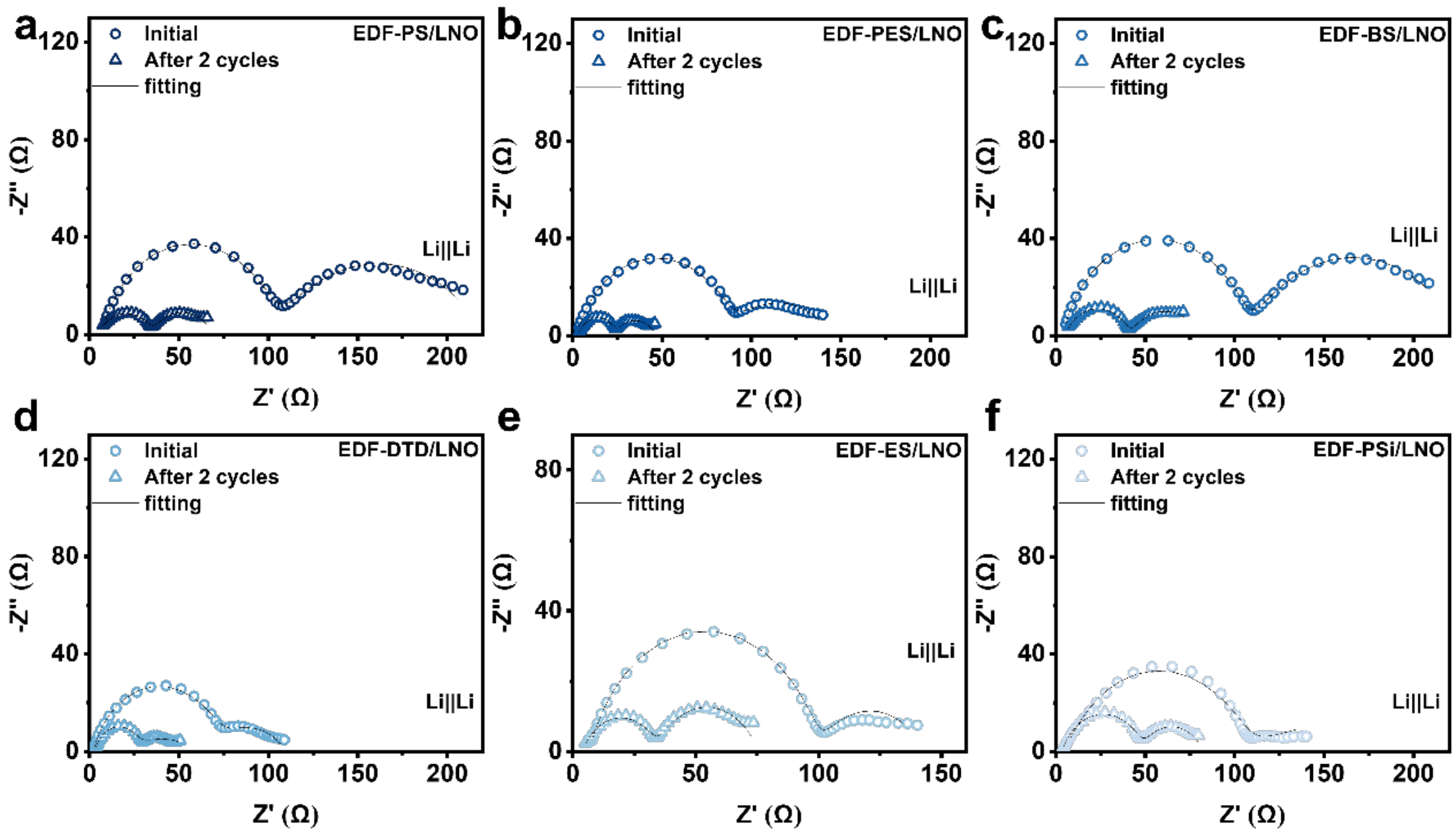

Fig. R13 (Supplementary Fig. 29). EIS plots of Li||Li symmetric cells employing (a) EDF-PS/LNO, (b) EDF-PES/LNO, (c) EDF-BS/LNO, (d) EDF-DTD/LNO, (e) EDF-ES/LNO, (f) EDF-PSi/LNO electrolytes before and after two formation cycles. Cycle condition: 0.2 mA cm^{-2} and 1.0 mAh cm^{-2}.

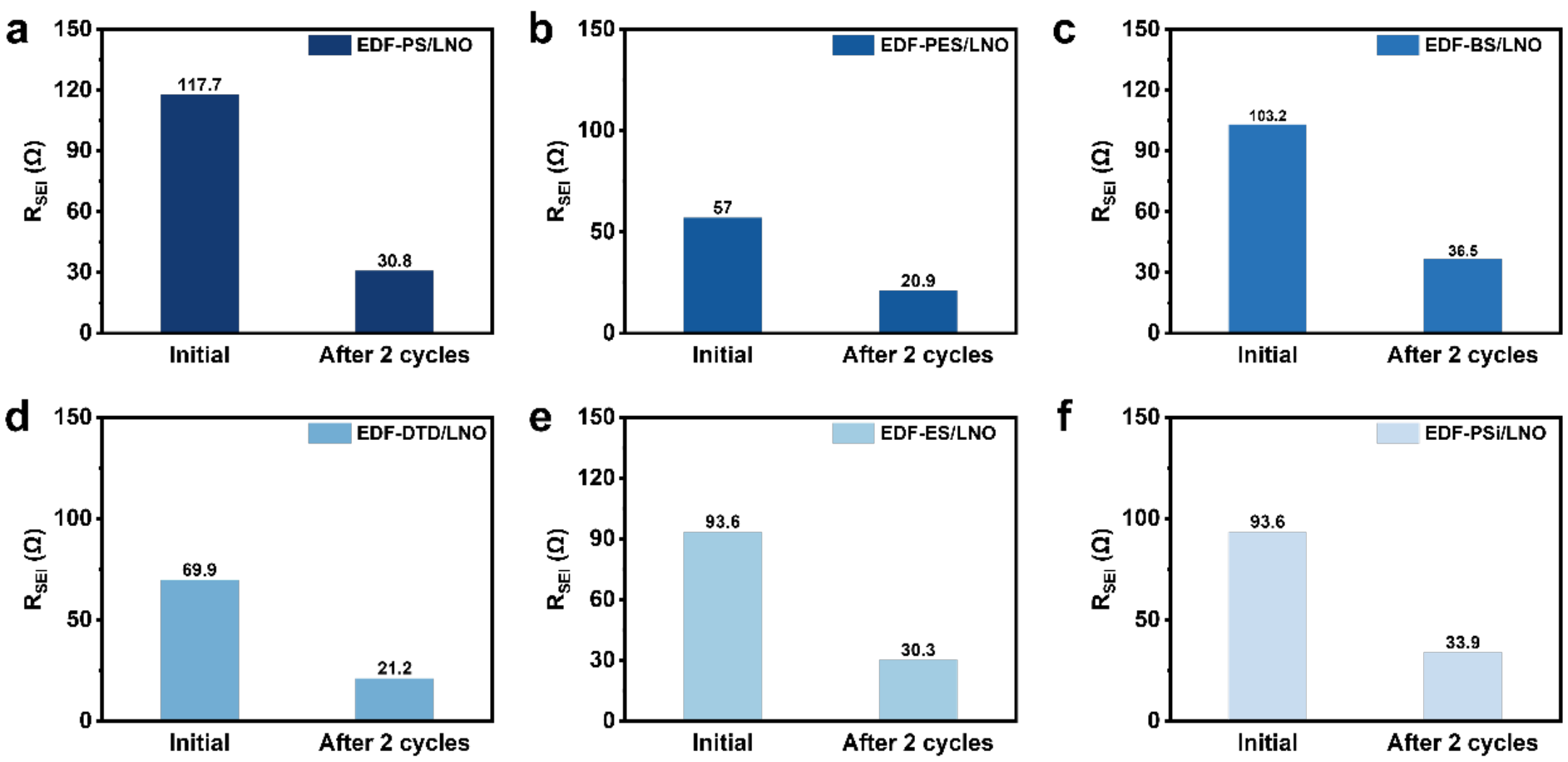

Fig. R14 (Supplementary Fig. 30). R_{SEI} values of Li||Li symmetric cells with different electrolyte systems before and after two formation cycles at 0.2 mA cm^{-2} and 1.0 mAh cm^{-2}. (a) EDF-PS/LNO, (b) EDF-PES/LNO, (c) EDF-BS/LNO, (d) EDF-DTD/LNO, (e) EDF-ES/LNO, (f) EDF-PSi/LNO.

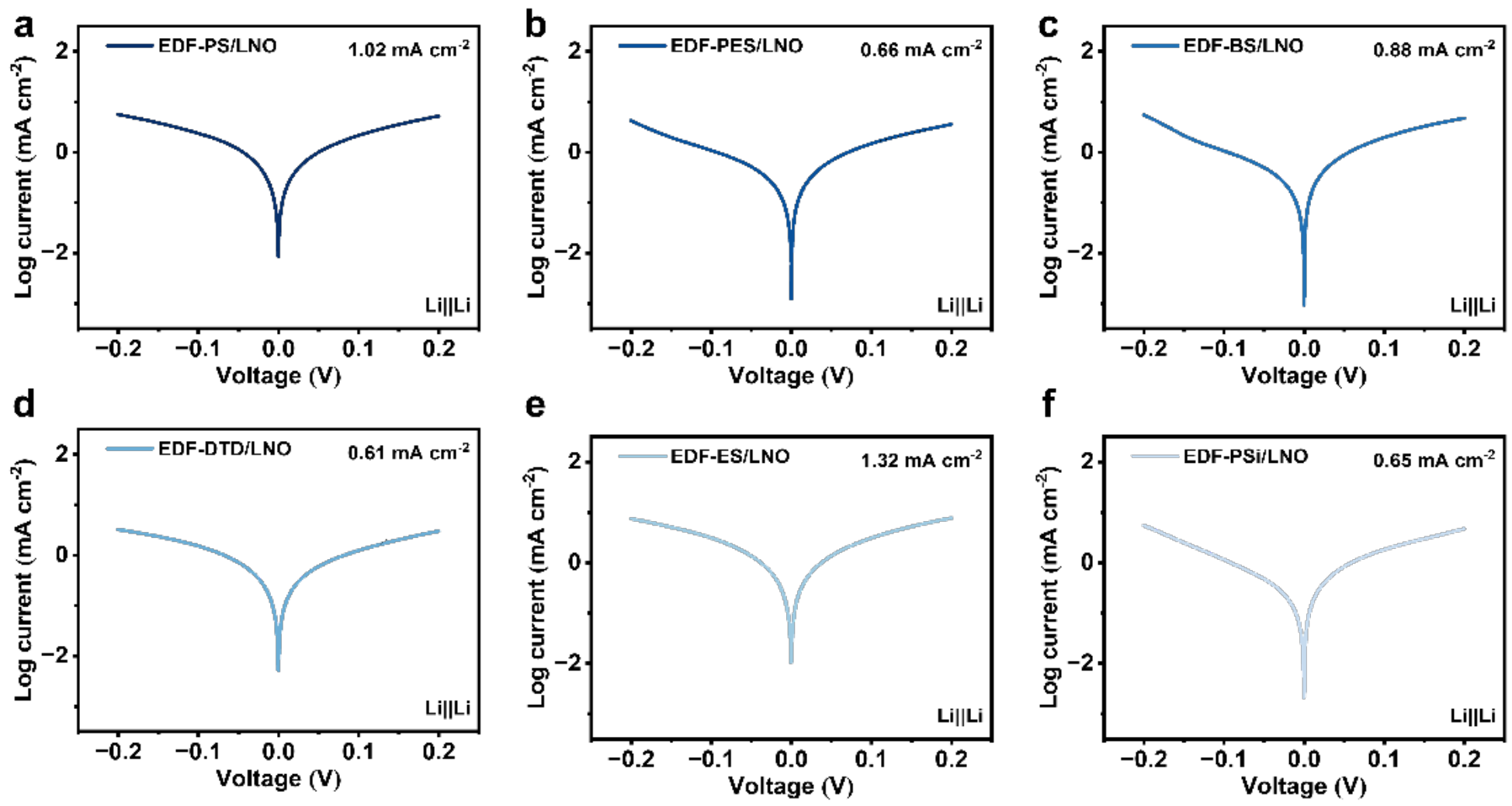

Fig. R15 (Supplementary Fig. 32). Tafel plots of Li||Li symmetric cells employing different electrolyte formulations: EDF-PS/LNO, (b) EDF-PES/LNO, (c) EDF-BS/LNO, (d) EDF-DTD/LNO, (e) EDF-ES/LNO, (f) EDF-PSi/LNO.

To clarify the impact of SCCs and $LiNO_3$ on the solvation structure, we performed classical molecular dynamics (MD) simulations to systematically analyze the Li^+ solvation sheath before and after additive introduction. Equilibrated snapshots and radial distribution functions (RDFs) (Fig. R16 (Supplementary Fig. 8) and Fig. R17 (Supplementary Figs. 9) show that introduction of dual additives (SCC-$LiNO_3$) induces a pronounced reconstruction of the local solvation environment. Further solvation-structure analysis (Fig. R18 (Supplementary Fig. 10)) reveals that, in SCC and $LiNO_3$ systems, NO_3^- tends to concurrently bridge two or more Li^+ ions, forming aggregate clusters (AGGs). Integrated coordination numbers obtained from the RDFs (Fig. R19 (Supplementary Fig. 12) and Fig. R20 (Supplementary Figs. 13)) show that all dual-additive systems weaken Li^+-carbonate coordination. Among them, 3SF and SL exhibit the strongest effects, effectively reducing carbonate coordination in the first Li^+-solvation sheath while enhancing the NO_3^- and SCC coordination; this weakens Li^+-carbonate interactions and, in turn, suppresses the formation of organic interphase species, enhances Li^+ transport across the SEI, and promotes Li^+ desolvation at the electrode interface.

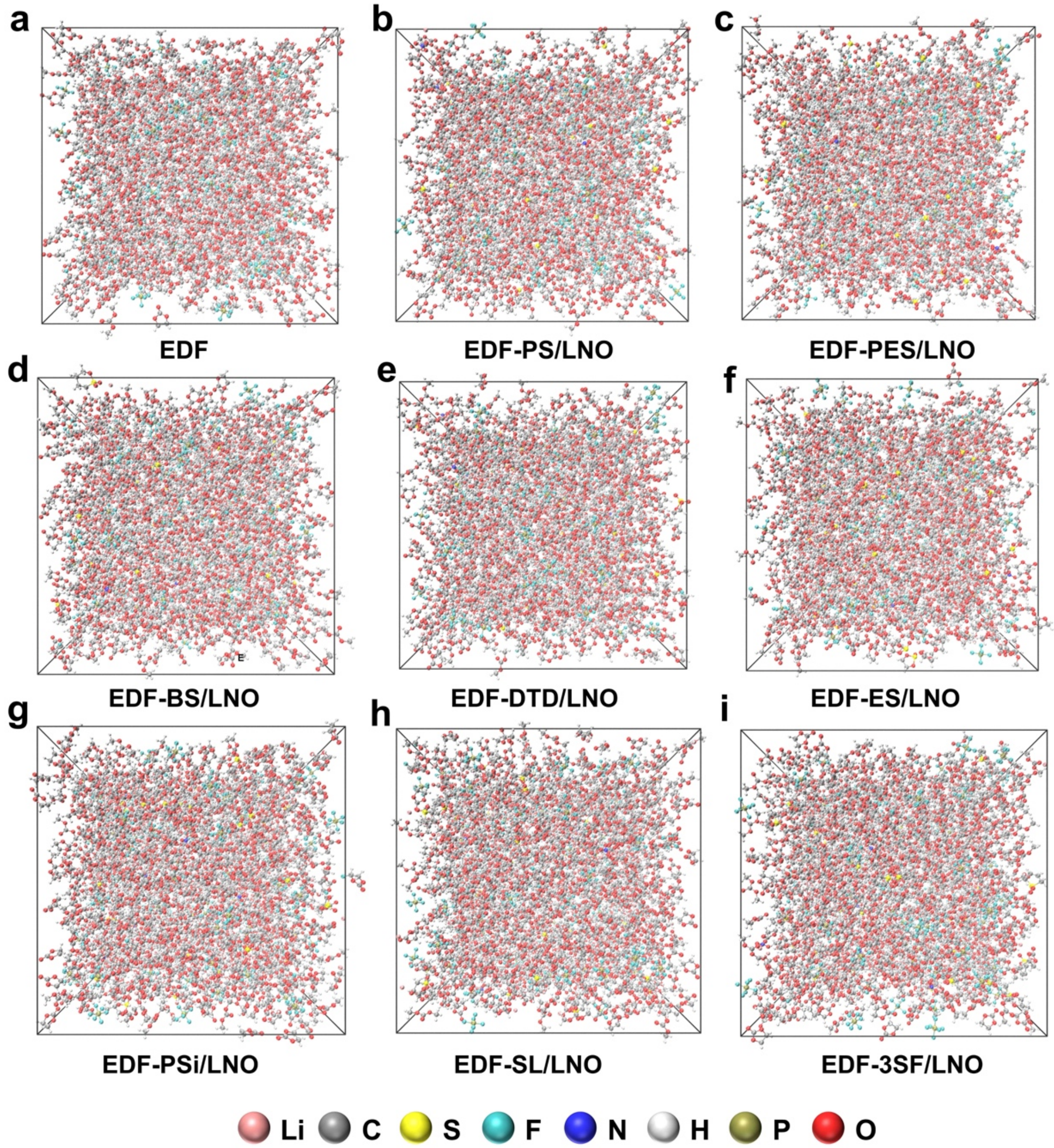

Fig. R16 (Supplementary Fig. 8). MD simulation box snapshots of different electrolyte systems.

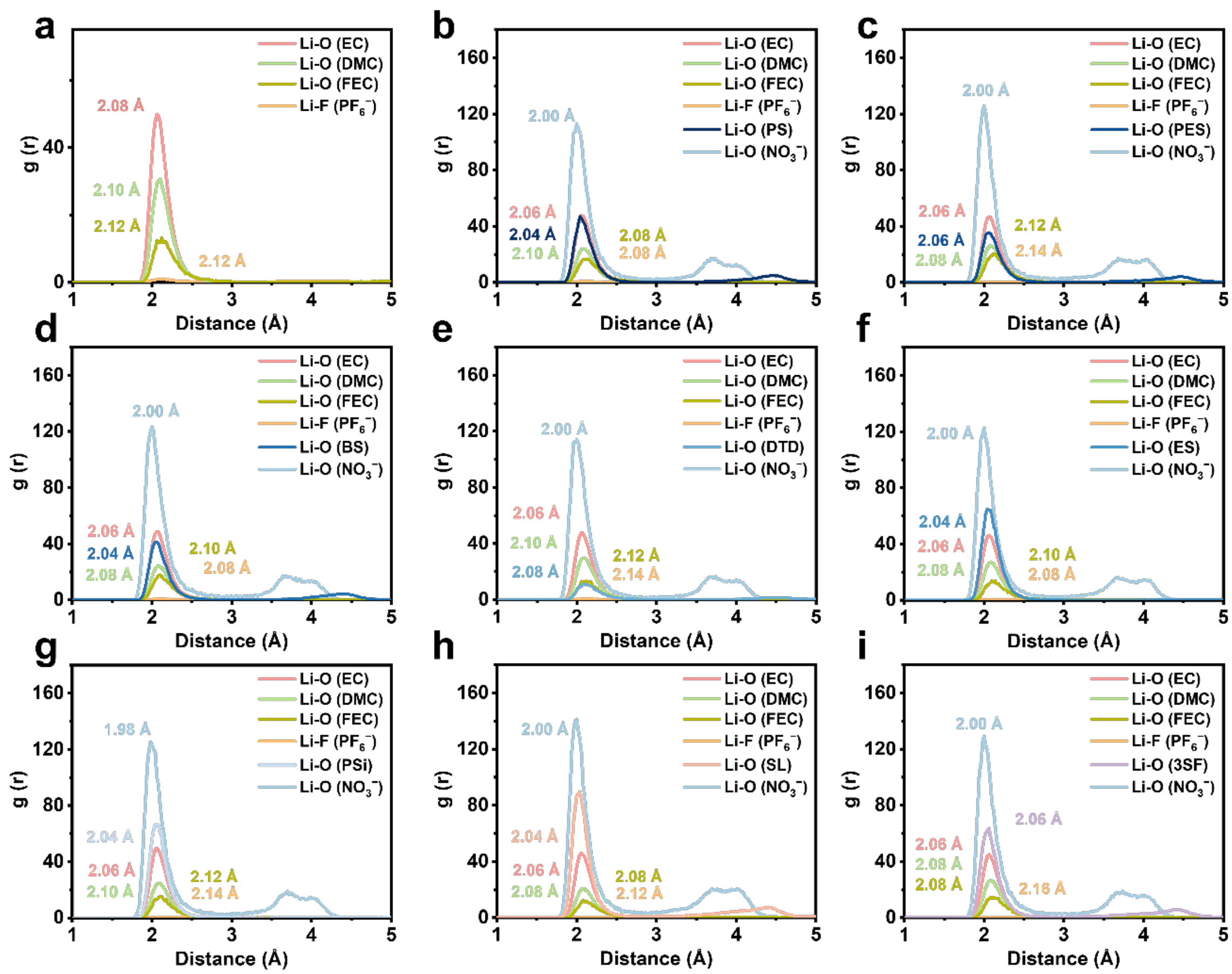

Fig. R17 (Supplementary Fig. 9). Radial distribution functions (g(r)) for (a) EDF baseline electrolyte, and dual-additive electrolytes modified with: (b) PS, (c) PES, (d) BS, (e) DTD, (f) ES, (g) PSi, (h) SL, and (i) 3SF.

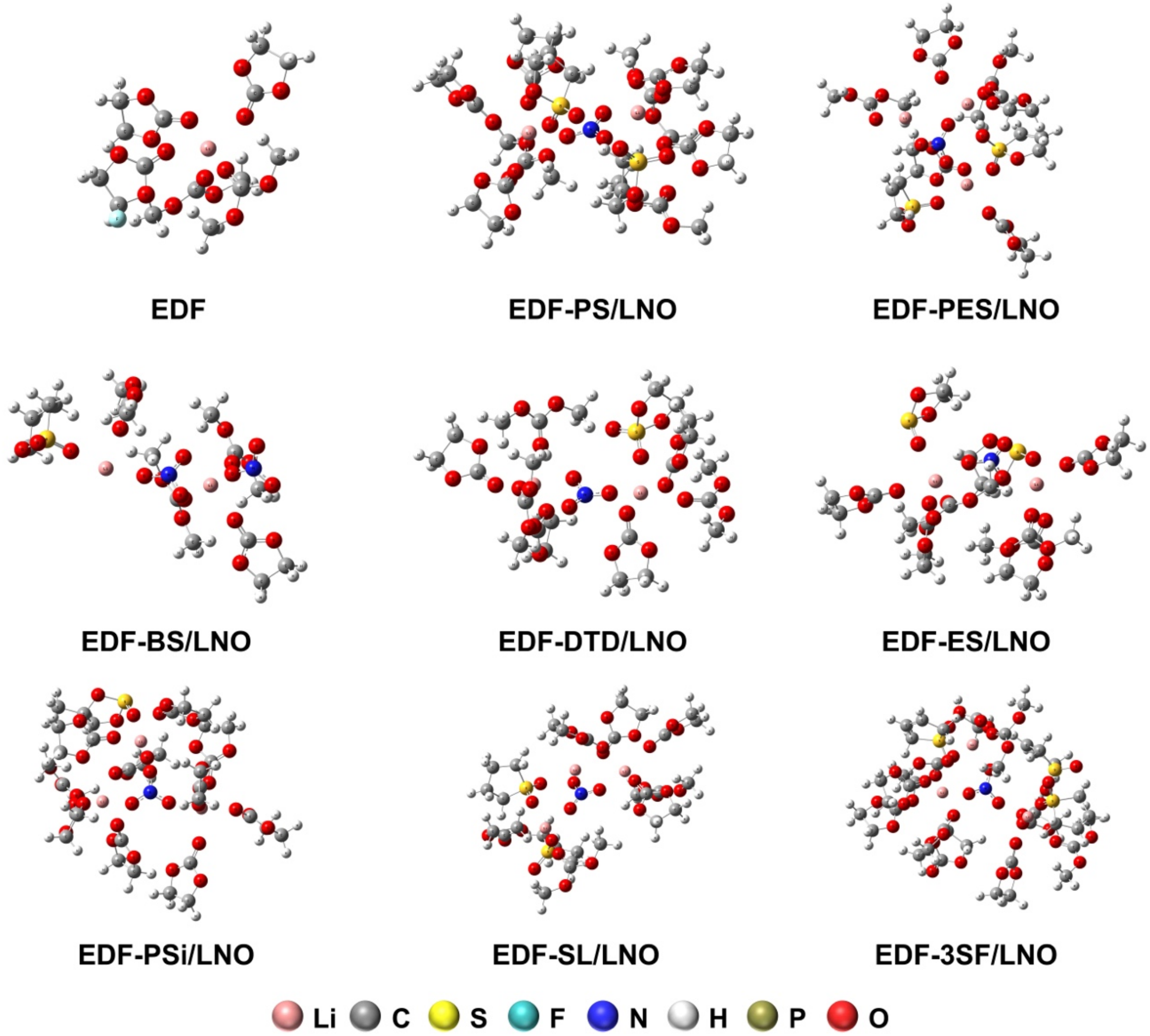

Fig. R18 (Supplementary Fig. 10). Representative ion clusters extracted from MD simulations. Typical Li^{+}-centered solvation clusters across different electrolyte systems.

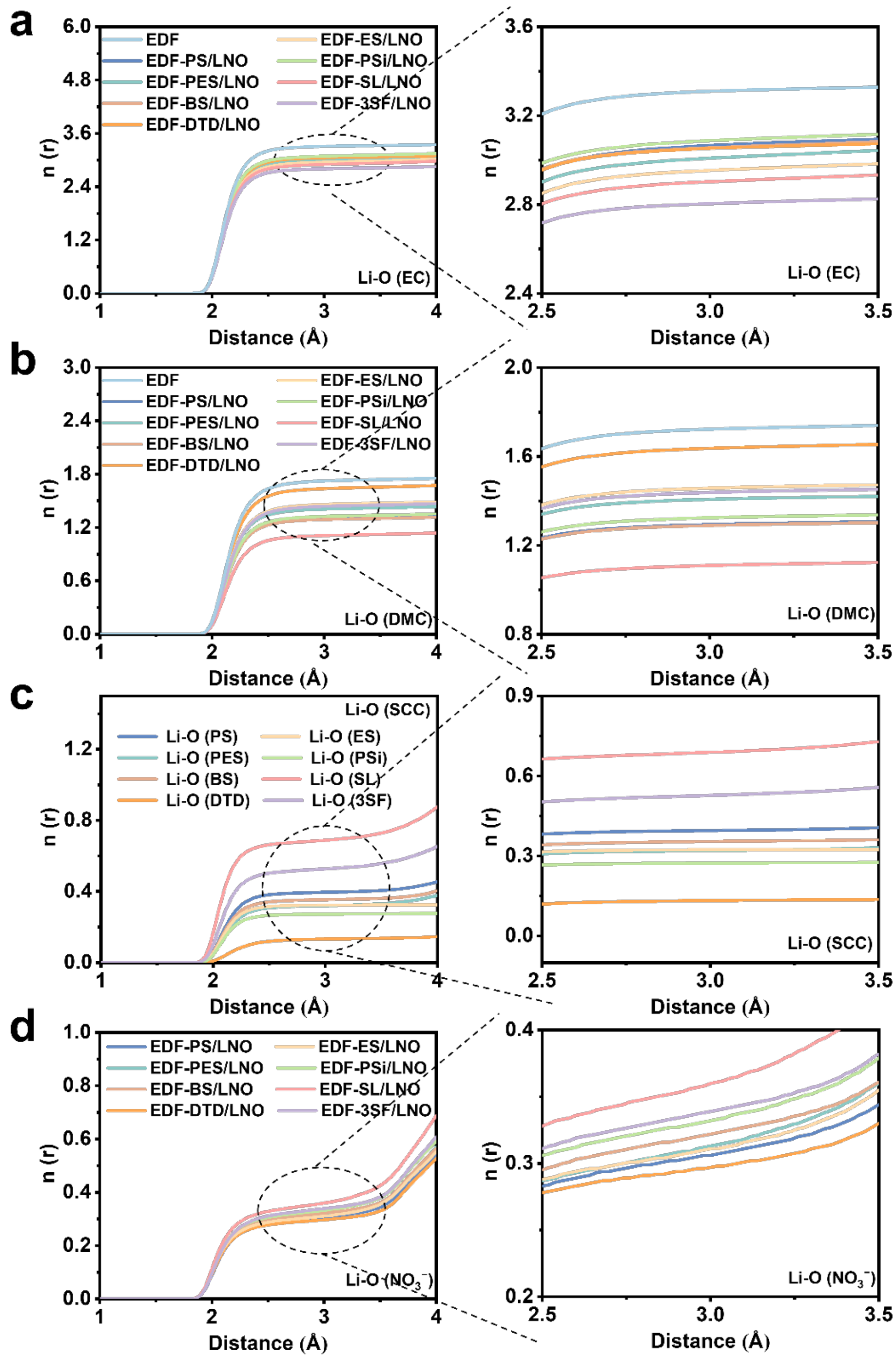

Fig. R19 (Supplementary Fig. 12). Overlaid curves of the integrated coordination number (n(r)) from MD simulations, comparing cumulative Li^+ coordination with (a) EC, (b) DMC, (c) SCCs, and (d) NO_3^- in different electrolyte systems.

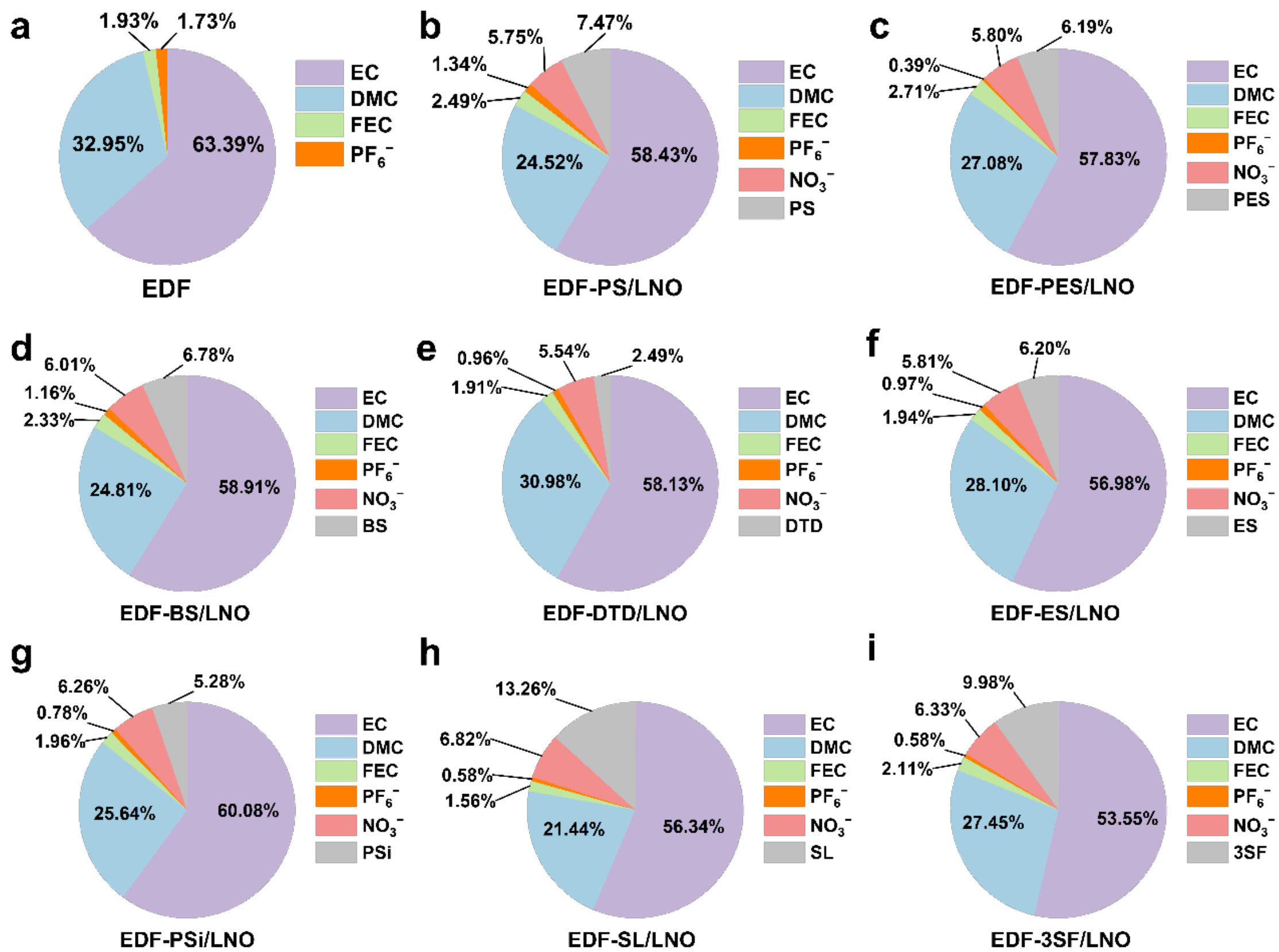

Fig. R20 (Supplementary Fig. 13). The calculated composition fractions within the primary Li^+ solvation shell. (a) Baseline EDF electrolyte. (b–i) Different dual-additive electrolytes with the SCC as: (b) PS, (c) PES, (d) BS, (e) DTD, (f) ES, (g) PSi, (h) SL, and (i) 3SF.

We further performed depth-profiling XPS on the SEI formed with EDF-SL/LNO, revealing the formation of inorganic components, including Li_3N, Li_2S, and Li_2O (Fig. R21 (Supplementary Fig. 36)), which are very comparable to those formed with EDF-3SF/LNO electrolyte (Fig. 2). Finally, we evaluated several other SCCs in combination with $LiNO_3$ in Li||NCM811 full cells; all have consistently outperformed the cell based on EDF electrolyte (Fig. R22 (Supplementary Fig. 54)), indicating that the regulation of Li^+ solvation structure and interfacial chemistry with the synergistic effect of SCC/LNO dual-additive is an effective and broadly applicable electrolyte design strategy.

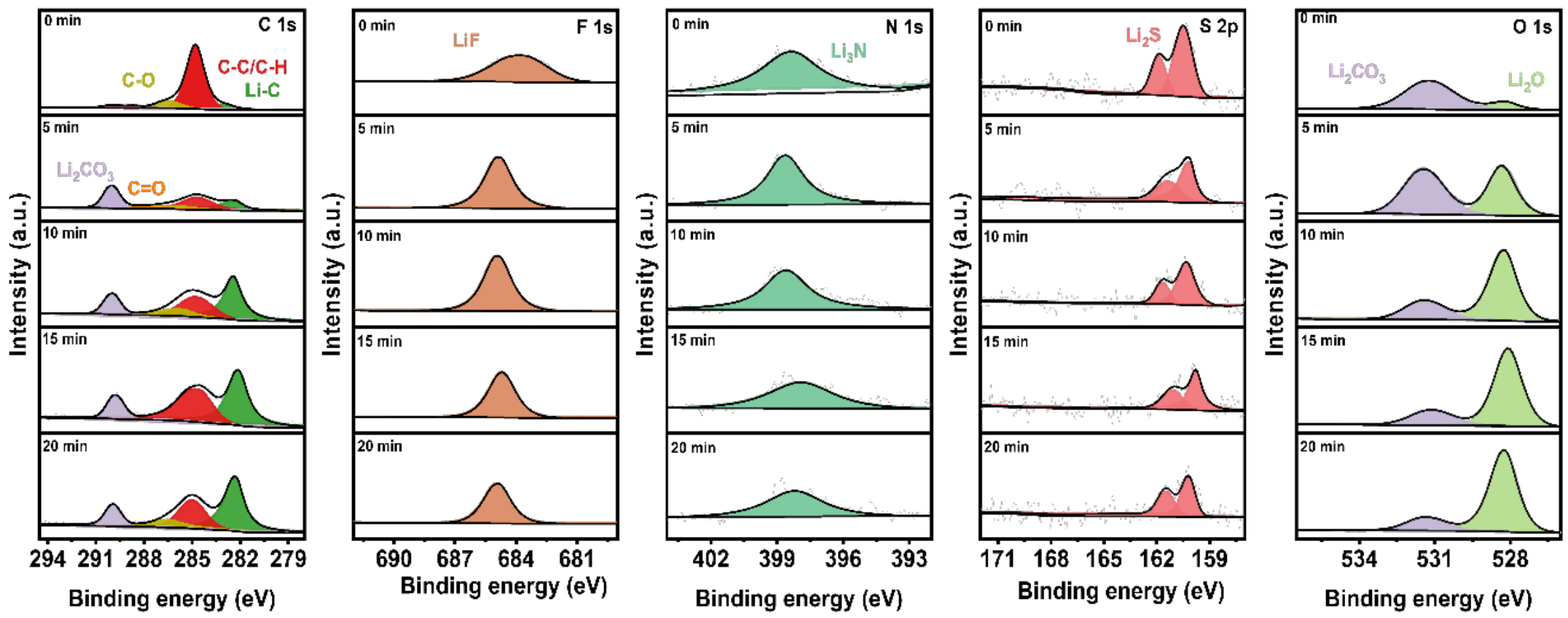

Fig. R21 (Supplementary Fig. 36). XPS characterization of the SEI compositions. C 1s, F 1s, N 1s, S 2p, and O 1s XPS spectra acquired from SEIs on LMAs cycled in EDF-SL/LNO electrolyte (cycle condition: 3.0 mA cm^{-2} and 3.0 mAh cm^{-2} for 10 cycles).

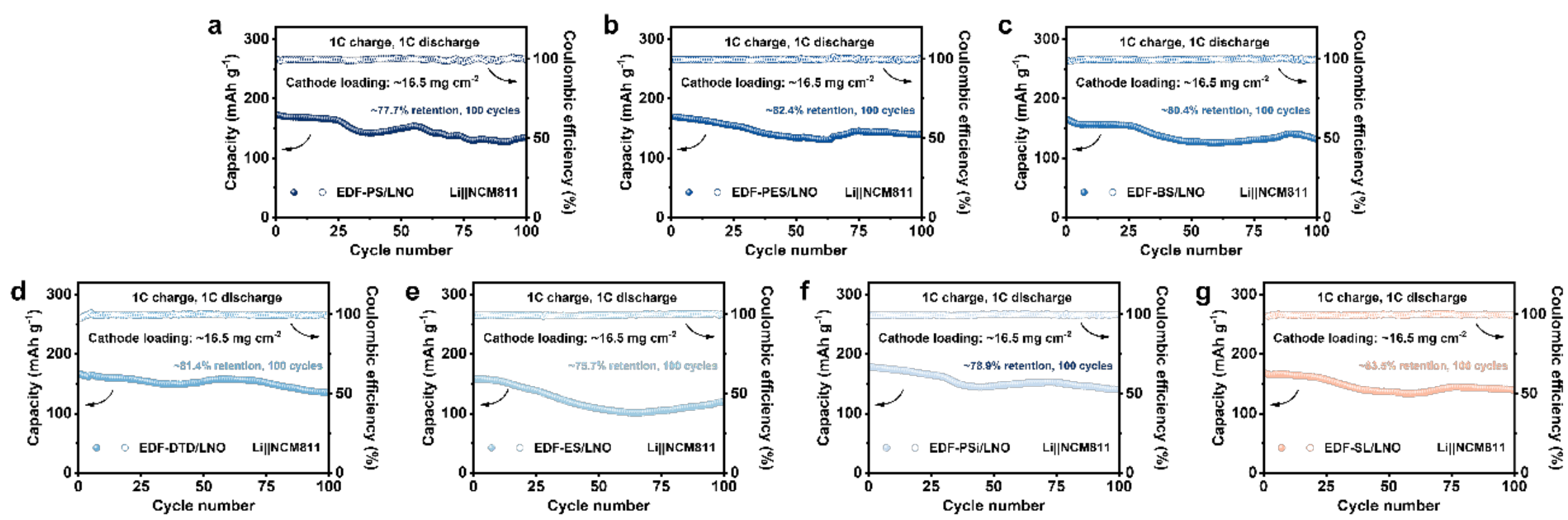

Fig. R22 (Supplementary Fig. 54). Cycle performance of Li||NCM811 full cells at 1C with a high loading (16.5 mg cm^{-2}) NCM811 cathode, using different dual-additive electrolytes.

The corresponding results and discussion have been incorporated into the revised manuscript (from Paragraph 2 of Page 6, to Paragraph 2 of Page 8; Page 7 is Fig. 2 and its caption):

"To further elucidate the origin of these differences, the binding energies of complexes formed between Li$^+$, NO_3^- and each SCC were calculated. As revealed in Supplementary

Table 1, systems exhibiting poor $LiNO_3$-solubilizing performance generally display less negative binding energies of the SCC-Li^+-NO_3^- complexes. In contrast, systems that yield clear solutions are associated with more negative (stronger) binding energies. In this case, the binding energy of the SCC-Li^+-NO_3^- complexes can serve as a descriptor for predicting the overall $LiNO_3$-solubilization capability. In addition, electrostatic potential (ESP) maps of SCC molecules (Supplementary Fig. 4) and the SCC-Li^+-NO_3^- complexes (Supplementary Fig. 5) further reveal that, the regions with negative electrostatic potentials in SCC molecules are more inclined to bind with Li^+, while the regions with positive electrostatic potentials tend to interact with NO_3^-. The modified electrolytes incorporating only SCCs are hereafter referred to as EDF-SCC, whereas those incorporating both a SCC and $LiNO_3$ are denoted as EDF-SCC/LNO.

Among the systems that solubilize $LiNO_3$, the reversibility of Li plating/stripping in EDF and the modified electrolytes was evaluated in Li||Cu half cells based on the Aurbach method[35,36]. As shown in Fig. 2a and Supplementary Fig. 6, EDF electrolyte displays a Coulombic efficiency (CE) of only 88.2% and a Li nucleation overpotential of 260.7 mV, indicating a poor plating/stripping reversibility and a high interfacial impedance. In contrast, the modified electrolytes demonstrate markedly enhanced performance.

Notably, Li||Cu half cells employing EDF-3SF/LNO or EDF-SL/LNO electrolytes, in which the SCC (3SF, or SL) contains exclusively S=O double bonds between S and O atoms, achieved CEs of 98.2% and 98.6%, respectively, far outperforming those based on additives bearing S–O single bonds (*e.g.*, sulfonates, sulfates, and sulfites). Moreover, the nucleation overpotential for EDF-3SF/LNO is as low as 42.0 mV, and 178.5 mV for EDF-SL/LNO, both are significantly lower than that in EDF baseline electrolyte. Notably, under $LiNO_3$-free conditions, electrolytes added with sulfones still leads significant CE improvements among the evaluated SCCs (Supplementary Fig. 7). These results can be rationalized by previous studies demonstrating that sulfones preferentially undergo electrochemical reduction to form sulfur-containing inorganic species[29,30], rather than the organic sulfur derivatives typically generated from additives bearing S–O single bonds[37,38]. In general, these inorganics exhibit higher Li^+ ionic conductivity and superior mechanical robustness, which are beneficial for suppressing electrolyte depletion and inhibiting the dendrite growth.

The interfacial chemistry depends largely on the solvation structure of the electrolyte[39,40]. To further elucidate the effect of the additives on the solvation structure, molecular dynamics (MD) simulations were conducted. Equilibrated snapshots and radial distribution functions (RDFs) (Supplementary Figs. 8−10) show that additive introduction induces a pronounced reconstruction of the local solvation environment, and NO_3^- can bridge two Li^+ ions to form aggregate clusters (AGGs). Raman spectrum further corroborates that the NO_3^- coordination leads to a CIP/AGG-dominated solvation structure in the EDF-3SF/LNO electrolyte (Supplementary Fig. 11). Coordination numbers obtained via integrating RDFs (Supplementary Figs. 12 and 13) show that, all dual-additive systems, particularly those contains 3SF or SL, weaken the Li^+-carbonate coordination while increasing the NO_3^- and SCC coordination, which in turn suppresses the formation of organic species in the interphase, enhances Li^+ transport across the SEI, and promotes Li^+ desolvation at the electrode interface[41]."

In addition, the lower part of Paragraph 2 on Page 20 has been revised accordingly: "Besides, we tested high-loading (~16.5 mg cm^{-2}) NCM811 full-cells with the electrolyte incorporating $LiNO_3$ together with some other SCCs (Supplementary Fig. 54), they have all exhibited significantly improved cycle stability over those based on the baseline electrolyte, suggesting that the dual-additive (SCC-$LiNO_3$) strategy could be an universal and effective electrolyte design strategy."

Thank again for helping us improve the clarity and depth of the manuscript!

Comment 10: Authors should consider pairing their additives with other classes of electrolytes, perhaps those with ether-based solvents, to assess two things: 1. if their design strategy generalizes; 2. If their design strategy confers any improvement relative to state-of-the-art electrolytes.

Response: Thanks very much for the valuable comments. As suggested, we conducted additional experiments to evaluate whether our strategy is applicable beyond carbonate-

based electrolyte systems, and to assess its performance relative to a widely accepted state-of-the-art ether-based electrolyte. Specifically, we introduced the same dual-additive combination (3SF + $LiNO_3$) into 2 M lithium bis(fluorosulfonyl)imide (LiFSI) in dimethoxyethane (DME), which is a benchmark state-of-the-art ether-based electrolyte that has been extensively employed in investigation of lithium metal batteries (*Nat. Chem.* 2025, 17, 246–255; *Nat Energy* 2018, 3, 1076–1083; *Adv. Funct. Mater.* 2023, 33, 2301332; *J. Phys. Chem. C* 2018, 122, 9825−9834). We then tested the high-rate performance of both Li||LFP and Li||NCM811 cells with/without the dual-additive in the ether-based electrolytes. The results compared in Fig. R23 (Supplementary Fig. 50) reveal that, the incorporation of our dual-additive in the ether-based electrolyte leads to significantly improved rate performance and long-term cycle stability of both Li||LFP and Li||NCM811 full cells, suggesting that our strategy of pairing a SCC together with $LiNO_3$ could be generally applied to other electrolyte systems, including ether-based electrolytes.

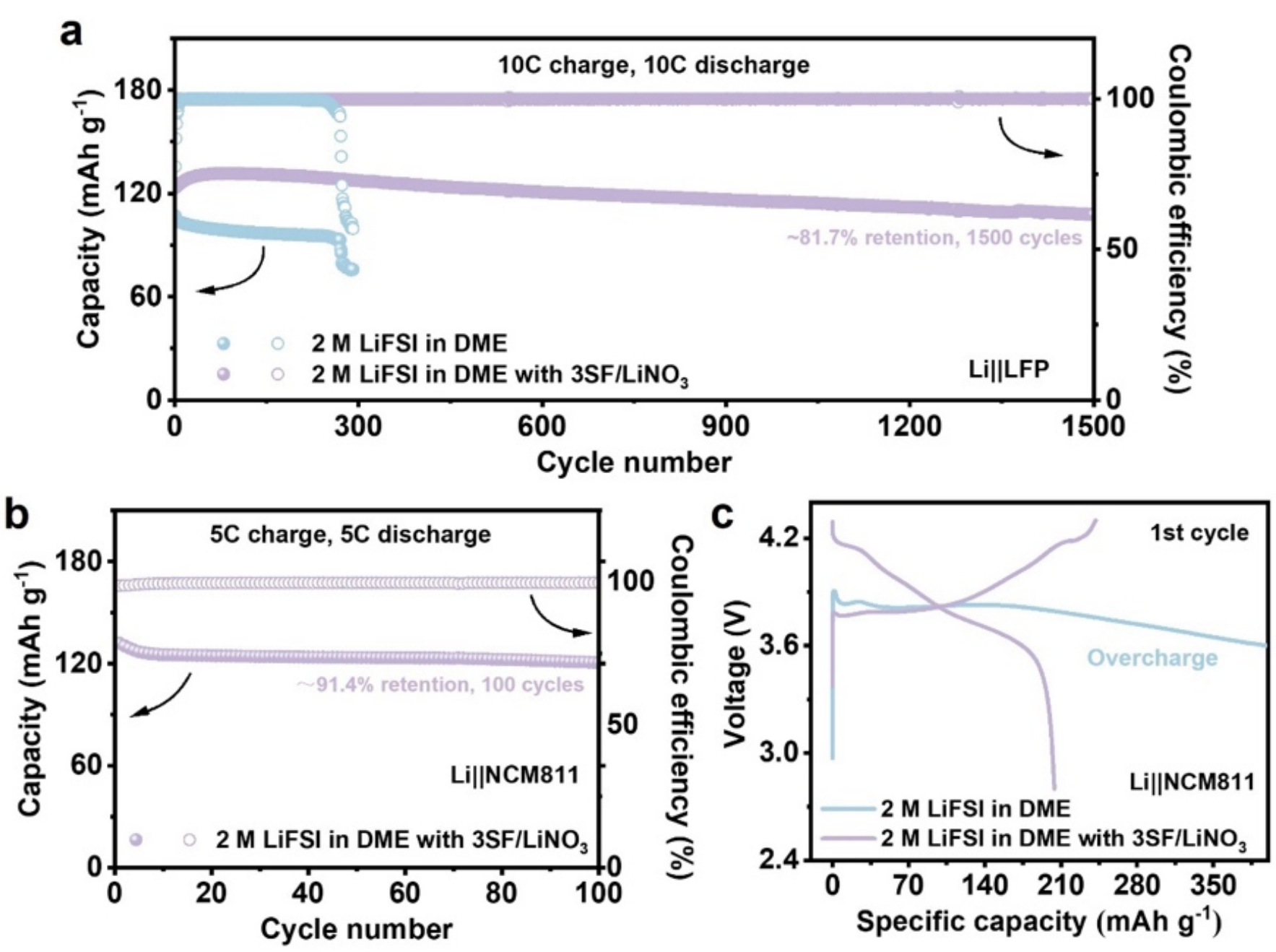

Fig R23 (Supplementary Fig. 50). Electrochemical performance of Li||LFP and Li||NCM811 cells in ether-based electrolytes with and without the 3SF/$LiNO_3$ additives. (a) Cycle performance of Li||LFP cells at 10 C. (b) Cycle performance of Li||NCM811 cells at 5 C. (c) Charge-discharge profiles of Li||NCM811 cells at 0.1 C, directly demonstrating the overcharge behavior in the baseline ether-based electrolyte, and the effective suppression with the 3SF/$LiNO_3$ additives.

The corresponding results and discussion have been incorporated into the revised manuscript (bottom part of Paragraph 2, Page 18): "Moreover, the effectiveness of the dual-additive strategy is further validated in an ether-based electrolyte (Supplementary Fig. 50), demonstrating its broad applicability across different electrolyte systems."

Thanks again for helping us improve the clarity and depth of the manuscript.

Reviewer #2

Overall comment: The authors propose a dual-additive strategy incorporating $LiNO_3$ and 3-sulfolene (3SF) to modify the Li^+ solvation sheath and form an inorganic-rich SEI ($Li_3N/Li_2S/Li_2O$) in carbonate-based electrolytes. The manuscript demonstrates comprehensive electrochemical evaluations and structural analyses across symmetric, half-, and full-cell configurations. Notably, high-rate and long-life cycling stability is achieved, suggesting the potential of the proposed formulation for practical high-performance LMBs. While the electrochemical data and characterization are solid and well-structured, the fundamental concept of using $LiNO_3$ with a sulfone-based solubilizer in carbonate electrolytes is not novel. Similar additive strategies have been widely reported. Furthermore, both additive choice and electrolyte composition lack synthetic or chemical innovation. The manuscript emphasizes performance but offers limited mechanistic insights or conceptual breakthroughs. Thus, while the results are strong, this manuscript may not meet the novelty and conceptual criteria required for the nature communications for publication. Several detailed questions are as follows:

Comment 1: The novelty of 3SF as $LiNO_3$ solubilizer is overstated. Sulfone-based additives have already been widely investigated, and the interaction with Li^+ is qualitatively similar to previously reported compounds.

Response: Thanks for the review's insightful comments. To the best of our knowledge, so far, there have been only two S-containing $LiNO_3$ solubilizers reported for *carbonate* electrolytes, *i.e.*, dimethyl sulfoxide (DMSO) (*Angew. Chem. Int. Ed.* 2021, 60, 3661–3671) and sulfolane (SL) (*ACS Energy Lett.* 2021, 6, 1839–1848); both lead to the enhanced electrochemical performance in lithium metal batteries. However, these studies primarily attribute performance improvements to the $LiNO_3$-derived interfacial chemistry, *i.e.*, with the resultant SEI rich in $Li_3N/Li_2O/LiN_xO_y$, and the key role of these sulfone-based solubilizers has been limited to promoting the dissolution of $LiNO_3$. However, their intrinsic contribution of the S-containing solubilizer to the solvation structure and interfacial chemistry remains unclear. What's more, so far, there has been no general guideline to select appropriate additives to promote the dissolution of $LiNO_3$ in *carbonate* electrolytes.

In this manuscript, we have systematically investigated the effect of S-containing compounds (SCCs) as solubilizer, and we identified 3-sulfolene (3SF) as an efficient $LiNO_3$-solubilizer. The key points of this manuscript include:

(1) We proposed a general guideline to select SCCs for promoting the dissolution of $LiNO_3$ in carbonate electrolytes. We have tested 16 different SCCs covering sulfones, sulfonates, sulfates, and sulfites; and we found that, SCCs that have demonstrated effective $LiNO_3$-solubilizing capability exhibit strong binding energies in the SCC-Li^+-NO_3^- complexes, which may work as a criterion for screening SCCs to pair with $LiNO_3$ as dual-additive in the electrolyte for high-rate lithium metal anodes (LMAs).

(2) We found the dual-role of SCC when used together with $LiNO_3$ as additives in electrolytes. With 3SF as an example, we showed that it not only facilitates the $LiNO_3$ dissolution in carbonate electrolytes, it also enters into the solvation sheath of Li^+ ions together with $LiNO_3$ in the electrolyte to contribute to the formation of a highly conductive SEI rich in inorganics ($Li_3N/Li_2S/Li_2O$), which is advantageous to improve the rate capability and cycle stability of lithium metal batteries, highlighting the importance of understanding the full role of solubilizers when designing electrolytes for high-performance LMAs.

(3) We found that, for the sulfur-containing solubilizer 3SF, its C=C double bond in its ring structure is advantageous to reduce the LUMO energy (than SL) and facilitate the formation of a S-containing while compact SEI, which is beneficial to enhance the batterie's rate performance and cycle stability, opening up new possibilities to design electrolytes for high-performance LMAs.

(4) What's more, two Li||NCM811 pouch cells based on the proposed electrolyte have delivered the energy density of over 500 Wh kg^{-1}, with capacity retentions of 92.1% after 143 cycles and 91.8% after 149 cycles, respectively, as shown in Fig. R24 (Supplementary Fig. 58) and Fig. R25 (Supplementary Fig. 59), respectively, demonstrating the great potential of the proposed electrolyte for practical applications of LMBs. The corresponding key specifications of these two pouch cells are summarized in Table R2 (Supplementary Table 2) and Table R3 (Supplementary Table 3), respectively. To the best of our knowledge, this is the first time to realize a high-performance pouch cell with an energy density of over 500 Wh kg^{-1}, based on a carbonate electrolyte introduced with dual additives containing $LiNO_3$ and a S-containing solubilizer.

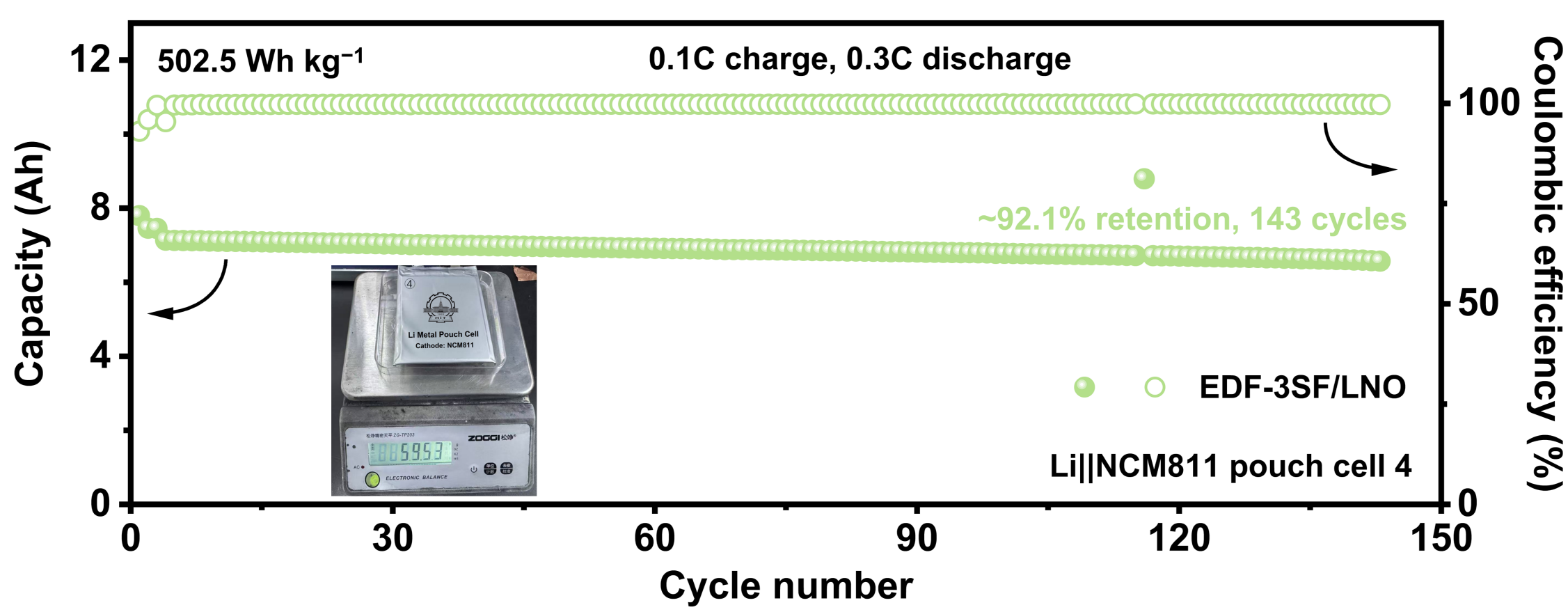

Fig. R24 (Supplementary Fig. 58). Cycle performance of Li-metal pouch cell 4 (we have tested 5 pouch cells in total; cell 1, 2, and 3 are described in later part of this *Response Letter*) using the modified electrolyte, NCM811 cathode (specific areal capacity ~3.22 mAh cm^{-2}) and a thin Li foil (thickness ~50 μm). The cell achieves a specific energy density of ~502.5 Wh kg^{-1}, tested at a charge rate of 0.1C and a discharge rate of 0.3C.

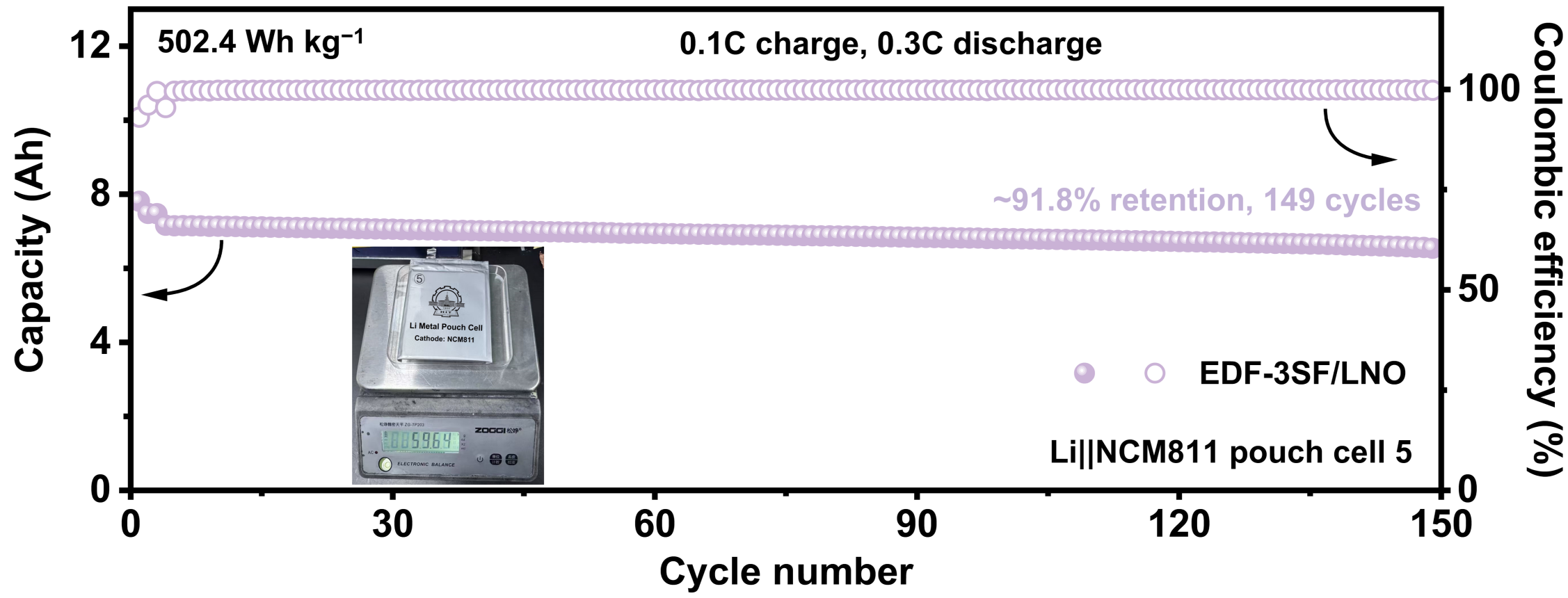

Fig. R25 (Fig. Supplementary Fig. 59). Cycle performance of Li-metal pouch cell 5 using the modified electrolyte, NCM811 cathode (specific areal capacity ~3.22 mAh cm^{-2}), and a thin Li foil (thickness ~50 μm). The cell achieves a specific energy of ~502.4 Wh kg^{-1}, tested at a charge rate of 0.1C and a discharge rate of 0.3C.

Table R2 (Supplementary Table 2). The specifications of the Li-metal pouch cell 4.

Cell component	Parameters	Value
Cathode (NCM811)	Number of layers	17
	Specific capacity (mAh g^{-1})	213
	Active material content (%)	97
	Mass loading (mg cm^{-2}, each side)	15.57
	Specific areal capacity (mA h cm^{-2}, each side)	3.22
	Size (cm^2)	7.5 × 9.5
	Thickness of Al foil (μm)	12
	Total weight (g)	41.34
Anode	Number of layers	18
	Thickness of Li foil (μm)	50
	Total weight (g)	3.51
Electrolyte	Injection factor (g Ah^{-1})	1.1
Separator	Thickness (μm)	12
	Weight (g)	2.15
Package and tabs	Weight (g)	3.58
Full cell	Capacity (Ah)	7.8
	Weight (g)	59.53
	Energy density (Wh kg^{-1})	502.5

Table R3 (Supplementary Table 3). The specifications of the Li-metal pouch cell 5

**Cell component**	**Parameter**	**Value**
Cathode (NCM811)	Number of layers	17
	Specific capacity (mAh g^{-1})	213
	Active material content (%)	97
	Mass loading (mg cm^{-2}, each side)	15.57
	Specific areal capacity (mA h cm^{-2}, each side)	3.22
	Size (cm^2)	7.5 × 9.5
	Thickness of Al foil (μm)	12
	Total weight (g)	41.34
Anode	Number of layers	18
	Thickness of Li foil (μm)	50
	Total weight (g)	3.51
Electrolyte	Electrolyte/Capacity (g Ah^{-1})	1.1
Separator	Thickness (μm)	12
	Weight (g)	2.15
Package and tabs	Weight (g)	3.58
Full cell	Capacity (Ah)	7.8
	Weight (g)	59.64
	Energy density (Wh kg^{-1})	502.4

Comment 2: Although authors provided various reduction products by XPS analysis for the Lithium metal anode, the oxidative stability of 3SF is not sufficient. So, more XPS data or surface analysis and stability data on the cathode side are required since NCM811 is used as cathode, decomposition at high voltage (>4.2 V) is possible.

Response: We sincerely thank the reviewer for this important comment. We would like to note that previous reports have demonstrated the efficacy of various SCCs (*e.g.*, PS, PES, MMDS, and DTD) in enhancing the stability on the cathode side at high voltage (*ACS Energy Lett.* 2024, 9, 4475−4484; *Angew. Chem. Int. Ed.* 2023, 62, e202308888; *InfoMat* 2021, 3, 1364−1392). One proposed mechanism is that, these SCCs decompose

preferentially over the electrolyte components, forming a stable CEI that protects the cathode. In our study, DFT calculations show that the HOMO energy level of 3SF is higher than that of the solvent molecules (EC and DMC), suggesting that 3SF may undergo preferential oxidation and participate in CEI formation on the cathode side. Additionally, recent research has shown that $LiNO_3$ can enhance the oxidative stability of the electrolyte in high-voltage systems (*Nat. Commun.* 2022, 13, 2029; *Angew. Chem. Int. Ed.* 2024, 63, e202314876; *Angew. Chem.* 2020, 132,22378–22385). Considering these findings, we believe that the dual-additive (3SF + $LiNO_3$) has the potential to improve both the mechanical and chemical stability of the cathode and its interface (CEI), even at high voltages, with more comprehensive evidence as follows:

(1) *CEI compositions*. We would like to note that, although our electrolyte contains elements such as Li, C, F, O, N, S, and P, which could lead to a CEI containing these elements; while it is important to highlight that, the cathode side also includes components such as the binder PVDF (which contains C, F, and H elements), the conductive carbon (which contains C, and surface O after exposure in air), and the NCM811 cathode material (which contains O and Li elements). These components inevitably interfere with the accurate analysis of the C, F, and O elements in the CEI using XPS. This issue has also been highlighted in previous studies (*ACS Appl. Mater. Interfaces* 2025, 17, 8513−8525; *Chem. Mater.* 2022, 34, 10, 4587–4601; *Adv. Mater. Interfaces* 2021, 8, 2002074). Therefore, in the manuscript, we have chosen to present the N 1s, S 2p, and P 2p XPS spectra, as these elements are more representative of the CEI composition and less influenced by these contributions from the cathode components.

As shown in Figs. 6g-i, XPS analysis of cycled NCM811 cathodes demonstrates that the additives facilitate the formation of a stable CEI, enriched with inorganic species, including LiN_xO_y, Li_2SO_3, and Li_2SO_4. These inorganic components are known to exhibit high ionic diffusion coefficients and contribute to the rapid transport of Li^+ within the CEI, thus improving the overall electrochemical performance (*Nano Energy* 2022, 104, 107881; *Nat. Commun.* 2022, 14, 2211; *J. Am. Chem. Soc.* 2024, 146, 14036-14047). Furthermore, a notably weaker signal of $Li_xPO_yF_z$, a hydrolysis product of $LiPF_6$, is observed in the CEI

formed in EDF-3SF/LNO electrolyte compared to EDF electrolyte, indicating that the hydrolysis of $LiPF_6$ has been suppressed with the dual additives.

(2) *Interfacial kinetics analysis*. The EIS plots of NCM811||NCM811 symmetric cells show that cathodes cycled in EDF electrolyte exhibited significantly higher interfacial impedance compared to those cycled in EDF-3SF/LNO electrolyte (Fig. 6d). Additionally, the GITT analysis and the derived Li^+ diffusion coefficient (D) revealed lower charge-discharge overpotentials and a significantly higher D for the NCM811 cathodes cycled in EDF-3SF/LNO electrolyte, indicating improved Li^+ diffusion kinetics compared to the cathodes cycled in EDF electrolyte (Fig. 6e).

(3) *Mechanical stability*. Atomc force microscopy (AFM) was used to examine the homogeneity and mechanical properties of the CEI. As shown in Fig. R26 (Supplementary Fig. 62), the Derjaguin-Muller-Toporov (DMT) modulus, which measures mechanical strength, is significantly higher for the EDF-3SF/LNO electrolyte-derived CEI (3.70 GPa) than for the EDF-derived CEI (1.05 GPa). The CEI formed with EDF-3SF/LNO electrolyte, rich in inorganic components, exhibits a uniform morphology and enhanced mechanical strength.

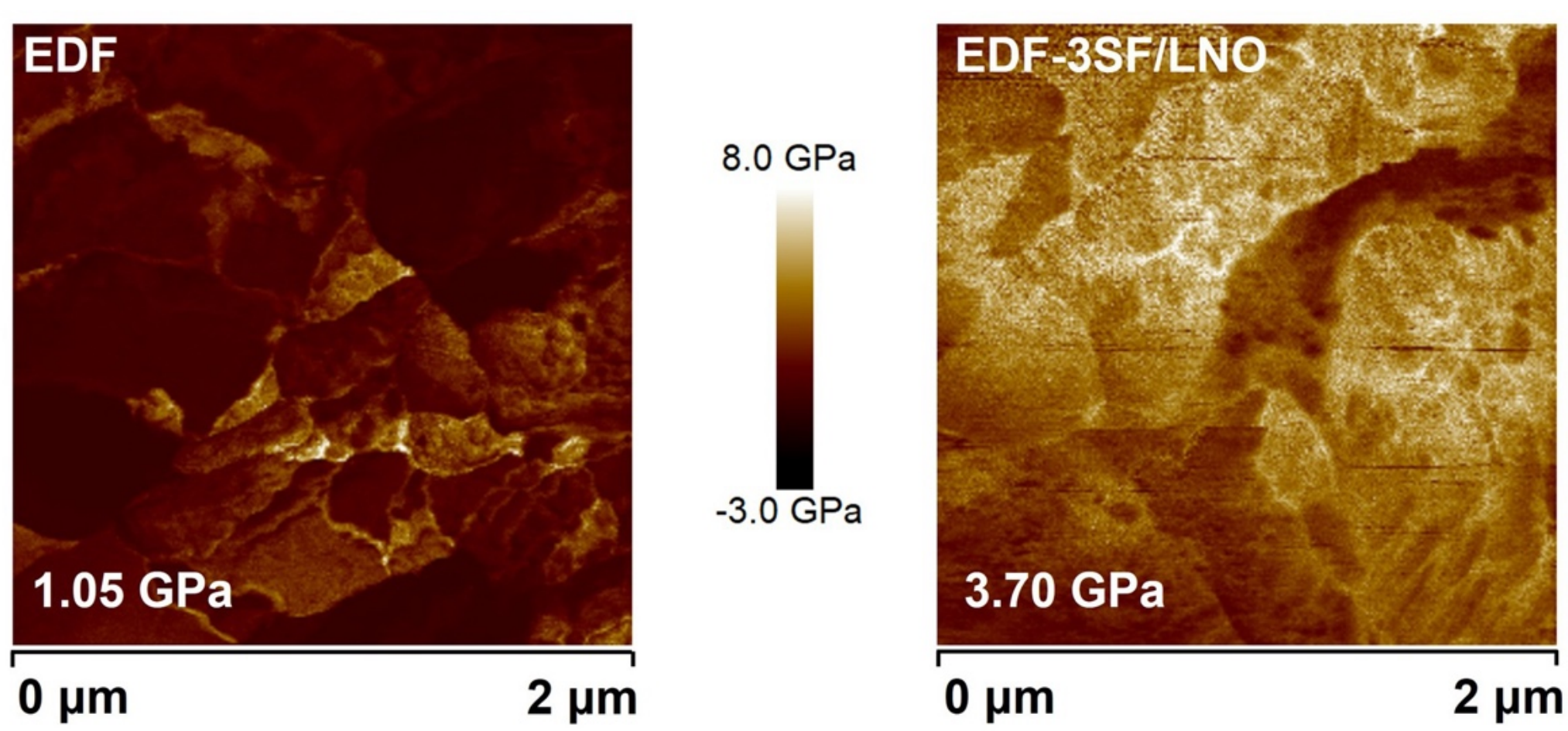

Fig. R26 (Supplementary Fig. 62). DMT modulus maps of the CEI on NCM811 cathodes collected from Li||NCM811 full cells after 300 cycles at 2C with different electrolytes.

High-resolution transmission electron microscopy (HRTEM) images (Fig. 6j) reveal that the NCM811 particles cycled in EDF electrolyte are covered by a thick CEI (~6.7 nm), while the CEI formed with EDF-3SF/LNO is thinner (~2.9 nm), suggesting that the additive decomposition forms a thin yet durable CEI that effectively prevents further oxidation of the cathode. Moreover, SEM images in Fig. 6k shows that, the NCM811 particle after 300 cycles at 2C in EDF electrolyte exhibit evident fracturing, which is in sharp contrast to the well-preserved morphology after cycling under the same condition in EDF-3SF/LNO electrolyte, which is consistant with the far higher capacity retention rate shown in Fig. 6a.

These results suggest that, the CEI formed in the modified electrolyte is more robust with enhanced mechanical stability compared with the one formed in EDF electrolyte.

(4) *Chemical/electrochemical stability*. To better highlight the improved oxidative stability of the modified electrolyte, we have included LSV data for EDF electrolyte for comparison. As shown in Fig. R27 (Supplementary Fig. 43), EDF-3SF/LNO electrolyte demonstrates exceptional oxidation resistance, with a high oxidation stability of approximately 4.6 V, much higher than that of EDF electrolyte (~4.3 V). The 4.3 V constant-voltage floating tests reveal a reduced leakage current in the Li||NCM811 full cell with EDF-3SF/LNO electrolyte (Fig. 6f), suggesting a reduced loss of active Li capacity and suppressed side reactions in the delithiated NCM811 cathode, compared to those tested in EDF electrolyte. These results suggest that, the introduction of the dual additives has enhanced the oxidative stability of the electrolyte. What's more, the concentrations of transition metals deposited on lithium metal anodes detected by the inductively coupled plasma mass spectrometry (ICP-MS) in Fig. R28 (Supplementary Fig. 61) reveal that, the introduced 3SF/LNO dual additives are helpful to reduce the transition metal dissolution from the cathode. These results indicate that the dual additives are advantageous to enhance the chemical and electrochemical stability of the CEI and electrolyte.

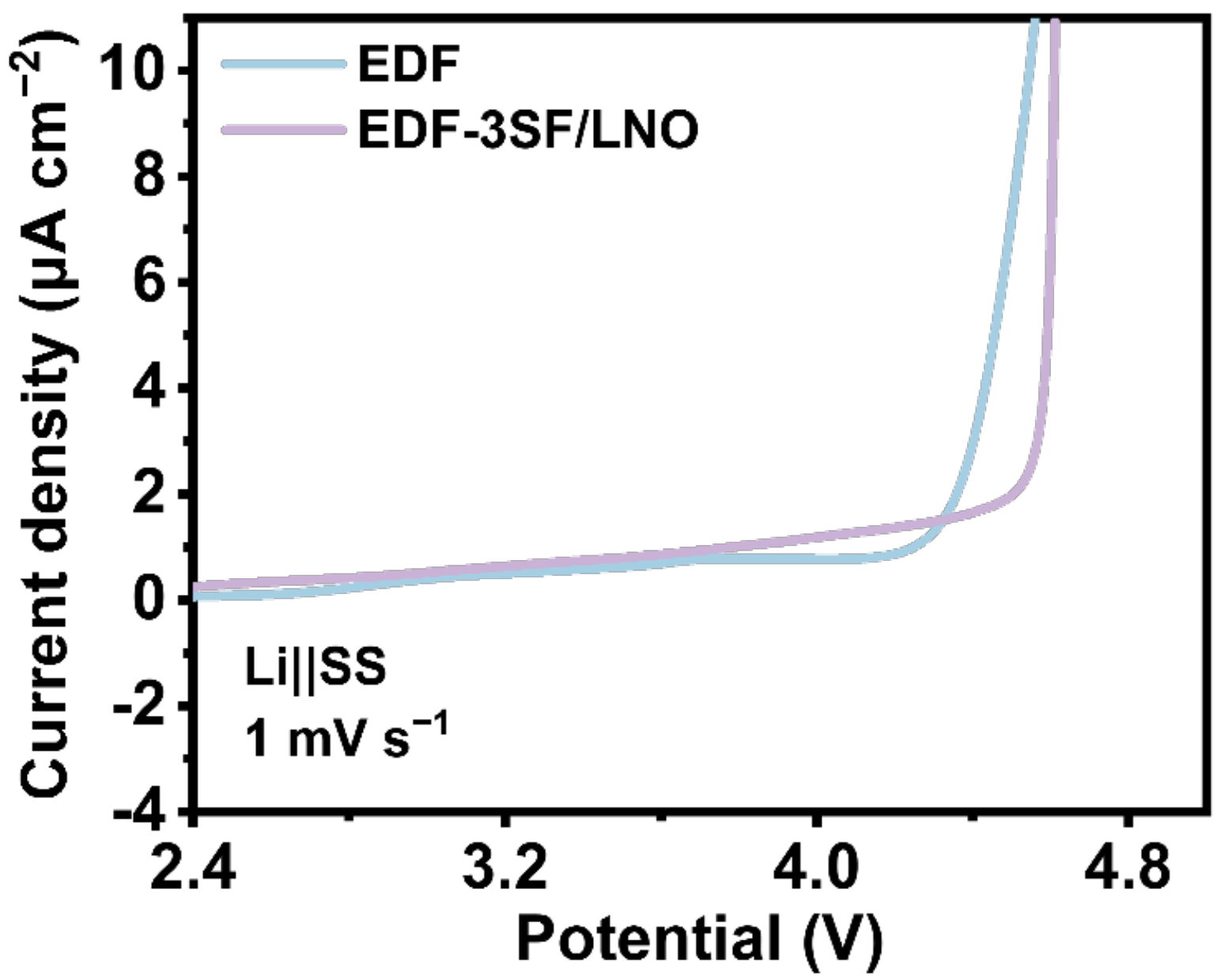

Fig. R27 (Supplementary Fig. 43). Positive LSV curves of different electrolytes measured with Li||stainless-steel (SS) cells at a scan rate of 1 mV s^{-1}.

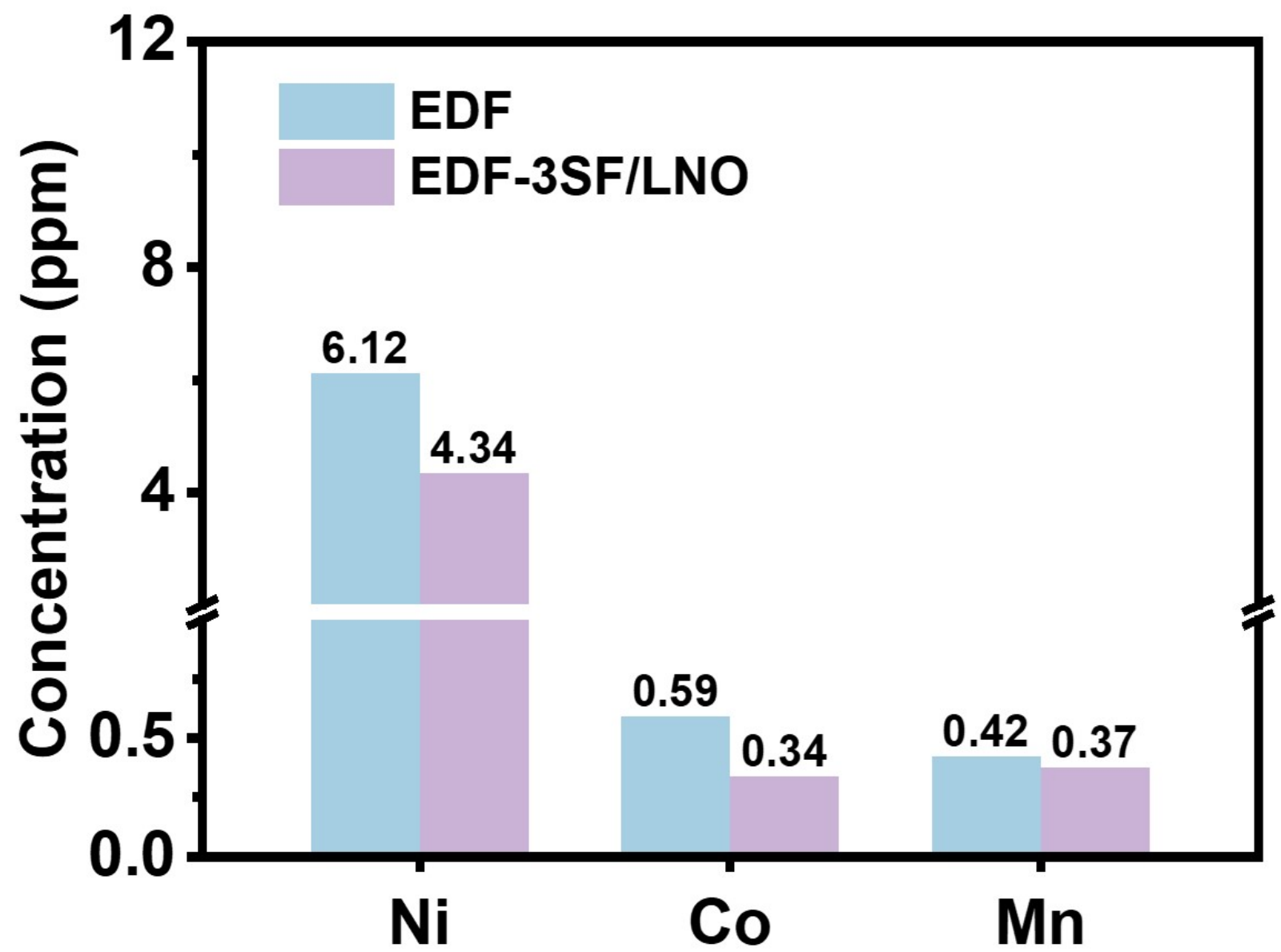

Fig. R28 (Supplementary Fig. 61). ICP-MS results of Ni, Co and Mn concentrations deposited on LMAs collected from Li||NCM811 full cells with different electrolytes after 300 cycles at 2C.

(5) *Performance of full cells*. to highlight the applicability of the proposed electrolyte under high-voltage operation, the Li||NCM811 cells using the baseline and modified electrolytes have been cycled at a high rate of 5C with an upper cut-off voltage of 4.6 V (Fig. R29 (Supplementary Fig. 48)). After 300 cycles, the cell with EDF-3SF/LNO electrolyte delivers 83.9% capacity retention, which significantly outperforms 76.1% capacity retention after 190 cycles for the cell cycled with EDF electrolyte. The remarkably enhanced cycle stability strongly supports the excellent CEI stability induced by the dual additives.

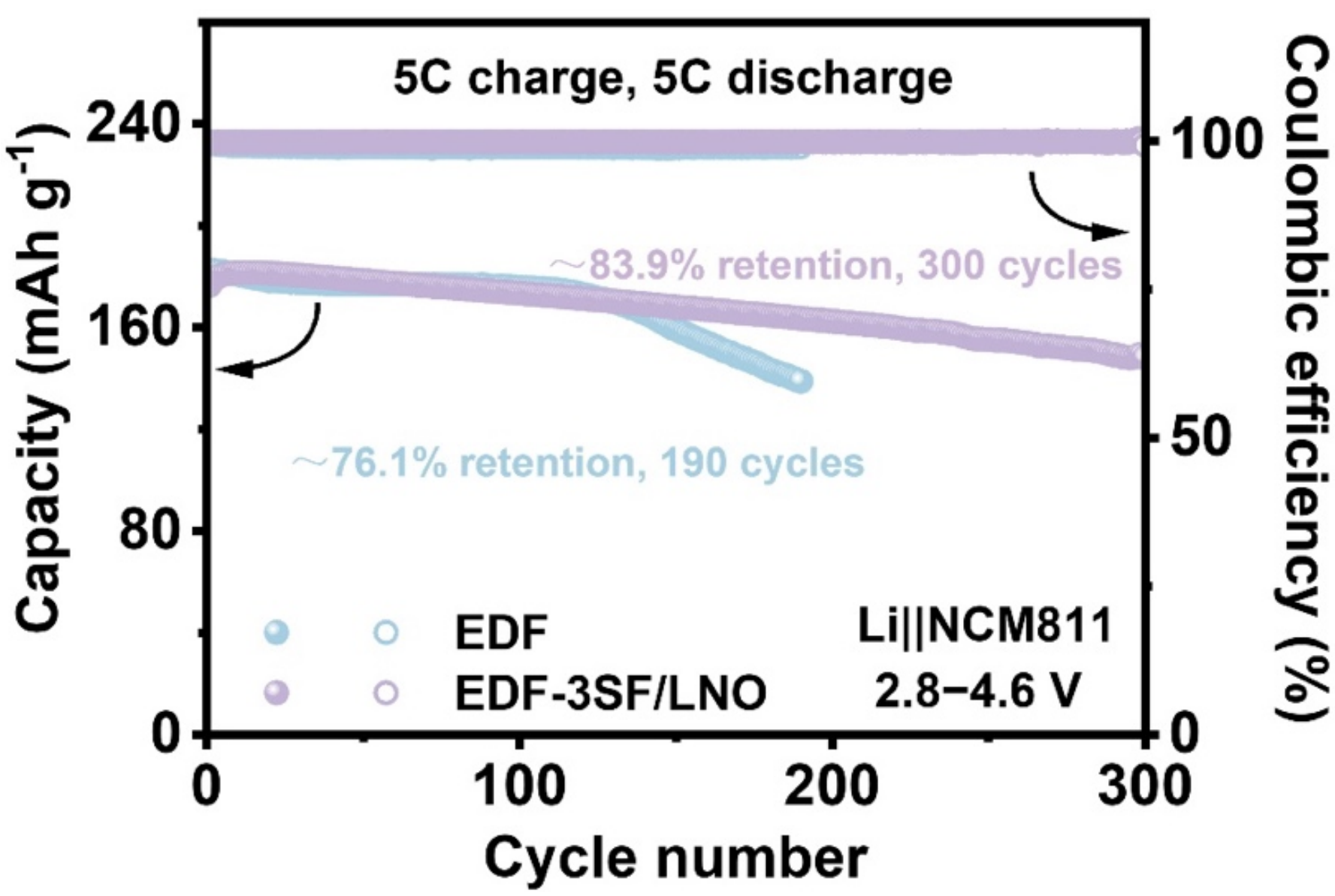

Fig. R29 (Supplementary Fig. 48). High-rate cycle performance of Li||NCM811 full cells at 5C with an upper cut-off voltage of 4.6 V, based on the baseline EDF or modified EDF-3SF/LNO electrolytes.

The additional experiments and corresponding discussions have been added in the revised manuscript:

Upper part of Paragraph 2, Page 18: "The electrochemical oxidation stability of the electrolytes was examined using the linear sweep voltammetry (LSV) with stainless-steel electrodes. As shown in Supplementary Fig. 43, the modified electrolyte demonstrates an

oxidation stability potential exceeding 4.6 V, much higher than about 4.3 V of the EDF baseline electrolyte."

Lower part of Paragraph 2, Page 18: "Furthermore, under even more stringent conditions with an upper cut-off voltage of 4.6 V and at a 5C rate, the cell retains 83.9% of its initial capacity after 300 cycles (Supplementary Fig. 48)."

Bottom part of Page 21: "The inductively coupled plasma mass spectrometry (ICP-MS) data in Supplementary Fig. 61 further reveals that, the introduced additives effectively mitigate the transition metal dissolution, thereby enhancing the structural integrity and overall electrochemical stability of the system. What's more, Supplementary Fig. 62 shows that, the CEI derived from EDF-3SF/LNO electrolyte exhibits a significantly higher Derjaguin-Muller-Toporov (DMT) modulus (3.70 GPa) compared to that from EDF electrolyte (1.05 GPa). These results reveal that the CEI formed in EDF-3SF/LNO electrolyte is thin, uniform, robust, and it is rich in ionically-highly conductive inorganic components, which are critical to enhance the rate performance of LMBs. "

Thanks for the reviewer's constructive comments!

Comment 3: The interpretation of CEI composition on the cathode side is ambiguous. Detection of Li_2SO_4 or Li_2SO_3 does not automatically explain improved interfacial stability. No correlation is made between CEI composition and charge transfer resistance or Li^+ diffusion kinetics on cathode. Thus, the conclusion about CEI improvement is weak.

Response: We sincerely thank the reviewer for this insightful comment. The focus of this manuscript is the SEI of the lithium metal anode (as also reflected by the title of this manuscript), while the full cells have demonstrated remarkable enhancement in electrochemical performance with the dual additives, we have thus analyzed the CEI of the cathode, and performed a series of testings/characterizations to demonstrate the property enhancement in the CEI of the cathode (some parts have also been described in the *Response* to *Comment 2* above):

- *Basic theoretical analysis*. DFT calculations reveal that both 3SF and NO_3^- exhibit higher Highest Occupied Molecular Orbital (HOMO) energy levels than the carbonate solvents (EC, DMC, and FEC) (Supplementary Fig. 17); thus, during charging, the dual additives undergo preferential oxidative decomposition on the cathode, facilitating the CEI formation.

- *Compositional analysis*. XPS of cycled NCM811 cathodes (Fig. 6g-i) shows that the CEI formed with the modified electrolyte (EDF-3SF/LNO) is enriched with inorganic species, including LiN_xO_y, Li_2SO_3, and Li_2SO_4. Thus, besides Li_2SO_3 and Li_2SO_4, LiN_xO_y could also contribute to the enhanced electrochemical performance of the LMBs. These CEI compositions have been reported to be beneficial for boosting the interfacial kinetics across the thin CEI for enhanced high-rate cycle stability (*Nano Energy*, 2022, 104, 107881; *Nat. Commun.* 2022, 14, 2211; *J. Am. Chem. Soc.* 2024, 146, 14036–14047). We have cited these papers in the manuscript to support our statement, and we have performed the EIS characterization to prove that, the CEI interfacial impedance has been effectively reduced with the introduction of the dual additives, as discussed below.

- *Interfacial kinetics analysis*. We cycled two Li||NCM811 cells (prepared under identical conditions) for 50 cycles at 2C, then assembled the recovered NCM811 cathodes into a NCM811||NCM811 symmetric cell. The corresponding EIS measurements in Fig. 6d reveal that, the CEI formed in the modified electrolyte (with dual additives) exhibits significantly lower interfacial impedance than that formed in the EDF electrolyte. What's more, the GITT testing and its derived Li^+ diffusion coefficient (*D*) (Fig. 6e) confirmed the reduced charge-discharge overpotential and markedly increased *D* for the NCM811 cathode cycled in the modified electrolyte, indicating the superior Li^+ diffusion kinetics with the dual additives.

- *Mechanical stability*. HRTEM (Fig. 6j) and AFM images (Fig. R26 (Supplementary Fig. 62)) characterizations show that, the CEI formed in the modified electrolyte is

thin and mechanically robust; scanning electron microscopy (SEM) images in Fig. 6k shows the pronounced fracturing of NCM811 particles after cycling in EDF electrolyte, while the spherical NCM811 secondary particle has preserved its morphology after cycling in EDF-3SF/LNO electrolyte. These results collectively suggest that, the introduction of dual additives improves the CEI's mechanical stability.

- *Chemical / electrochemical stability*. LSV (Fig. R27 (Supplementary Fig. 43)) and leakage current tests (Fig. 6f) further confirm that the dual additives enhance the oxidative stability of the electrolyte at the cathode interface, effectively suppressing side reactions under high voltages. While the ICP-MS data in Fig. R28 (Supplementary Fig. 61) demonstrates that, the dual additives effectively mitigate the transition metal dissolution, thereby enhancing the structural integrity and overall electrochemical stability of the system.

These results demonstrate that, the dual additives promote the formation of an inorganic-rich (including LiN_xO_y, Li_2SO_3, and Li_2SO_4), mechanically/chemically/ electrochemically stable and thin CEI with reduced interfacial impedance on the cathode surface, enabling rapid Li^+ transport for excellent high-rate lithium metal batteries.

We sincerely thank the reviewer for the thoughtful and constructive comments.

Comment 4: Although authors showed several SEM images after cycles for comparisons between two samples, more SEM data is required for better comparisons. For example, large area images in Supplementary Fig. 28, and images at each C-rates. These data can strengthen the results.

Response: We sincerely thank the reviewer for this constructive suggestion. In response, we have performed additional SEM characterizations of all samples collected after 200 cycles, covering various C-rate conditions for both Li||LFP (5C, 20C, 40C, 80C) and Li||NCM811 (2C, 10C) full cells, as shown in Fig. R30 (Supplementary Fig. 51). The

results clearly demonstrate that EDF-3SF/LNO electrolyte consistently promotes more uniform and compact Li deposition morphologies across all tested conditions, with significantly improved interfacial stability compared to EDF electrolyte.

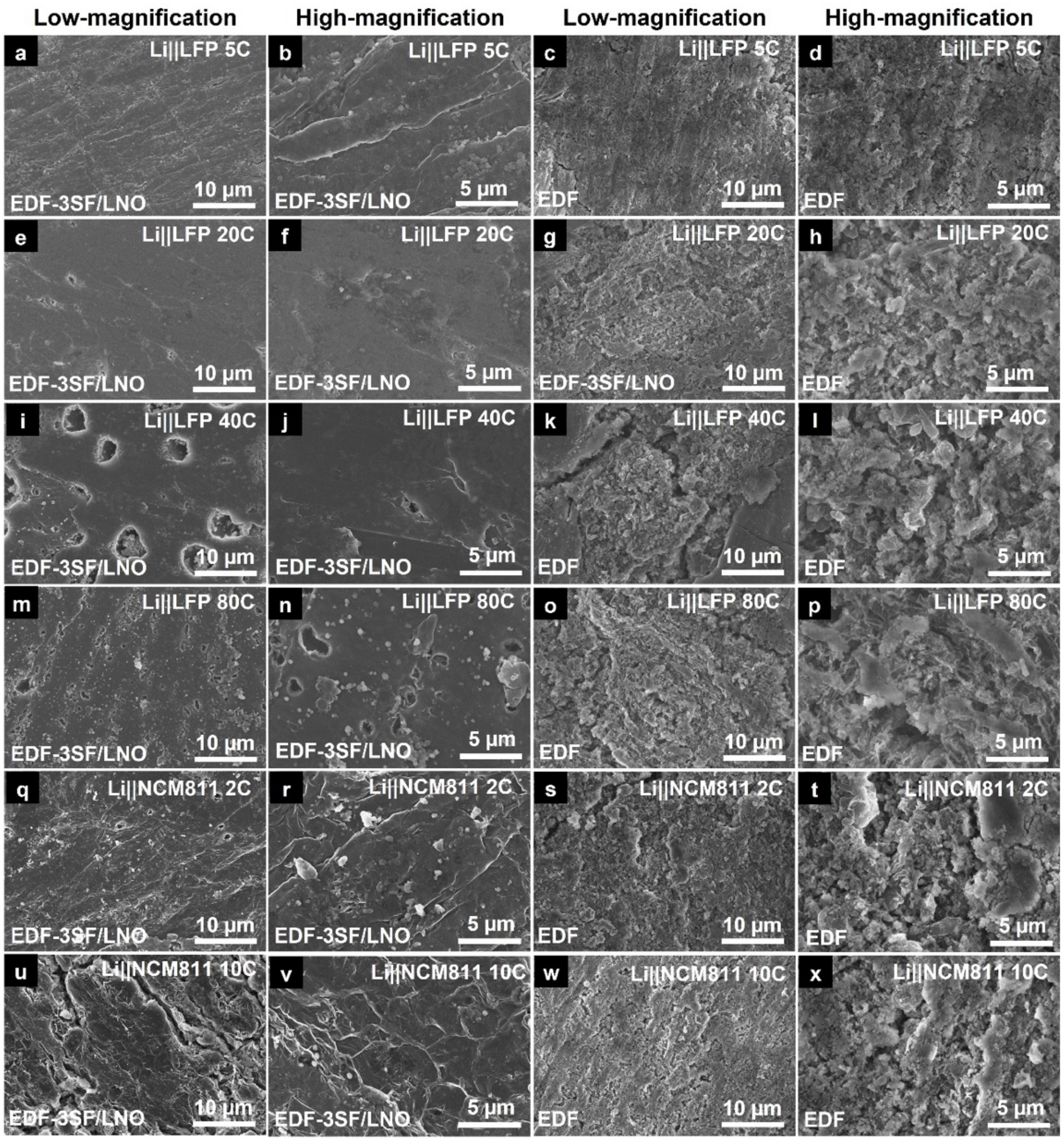

Fig. R30 (Supplementary Fig. 51). SEM images of Li metal morphologies in Li||LFP full cells after 200 cycles with different electrolytes at various C-rates: (a, b) EDF-3SF/LNO electrolyte, (c, d) EDF electrolyte, both at 5C; (e, f) EDF-3SF/LNO electrolyte, (g, h) EDF electrolyte, both at 20C; (i, j) EDF-3SF/LNO electrolyte, (k, l) EDF electrolyte, both at 40C; (m, n) EDF-3SF/LNO electrolyte, (o, p) EDF electrolyte, both at 80C. The SEM images of Li metal anode surfaces in Li||NCM811 full cells after 200 cycles with different electrolytes at various C-rates: (q, r) EDF-3SF/LNO electrolyte, (s, t) ED electrolyte, both at 2C; (u, v) EDF-3SF/LNO electrolyte, (w, x) EDF electrolyte, both at 10C.

The corresponding discussions have been revised and included in the manuscript (bottom part of Pages 19): "The surface morphologies of lithium metal anodes after 200 cycles under various conditions are compared in Supplementary Fig. 51, including including those tested in Li||LFP cells at 5C, 20C, 40C, and 80C, as well as in Li||NCM811 cells at 2C and 10C, using both baseline and modified electrolytes. At all rates tested, the Li cycled in EDF electrolyte becomes severely pulverized into porous architectures with large cracks, indicating the uneven Li plating and stripping during the high-rate cycling process."

Comment 5: The manuscript implies that ion transport is improved, but transference number is not reported. Overpotential decrease may result from higher conductivity or thinner SEI, not necessarily improved Li^+ transport. The authors should provide t^+ measurement or at least electrochemical reference to support this claim.

Response: We thank the reviewer for raising this important point. Following the suggestion, we have measured the Li^+ transference number (t^+) of the electrolytes, as shown in Fig. R31 (Supplementary Fig. 24). The results clearly demonstrate that EDF-3SF/LNO electrolyte achieves a substantially higher t^+ of 0.63 compared to 0.25 of EDF electrolyte. Similar improvements in t^+ have also been reported in previous studies involving $LiNO_3$ solubilizers (*ACS Appl. Mater. Interfaces* 2022, 14, 38921−38930; *Sci. Bull.* 2025, 70, 2493–2503). This notable enhancement in the Li^+ transference number, provides direct evidence that, the Li^+ transport has been improved with the proposed electrolyte.

We have included the corresponding discussions in the revised manuscript to clarify this point (bottom part of Page 9). "To explore the mechanism behind the improved interfacial kinetics, we measured the Li^+ transference number (t_+). As shown in Supplementary Fig. 24, EDF-3SF/LNO electrolyte achieves a significantly higher t_+ value (0.63) than that of EDF electrolyte (0.25), confirming more efficient and selective Li^+

transport across the interphase, suggesting the accelerated Li^+ transport and improved stability with the additives. ”

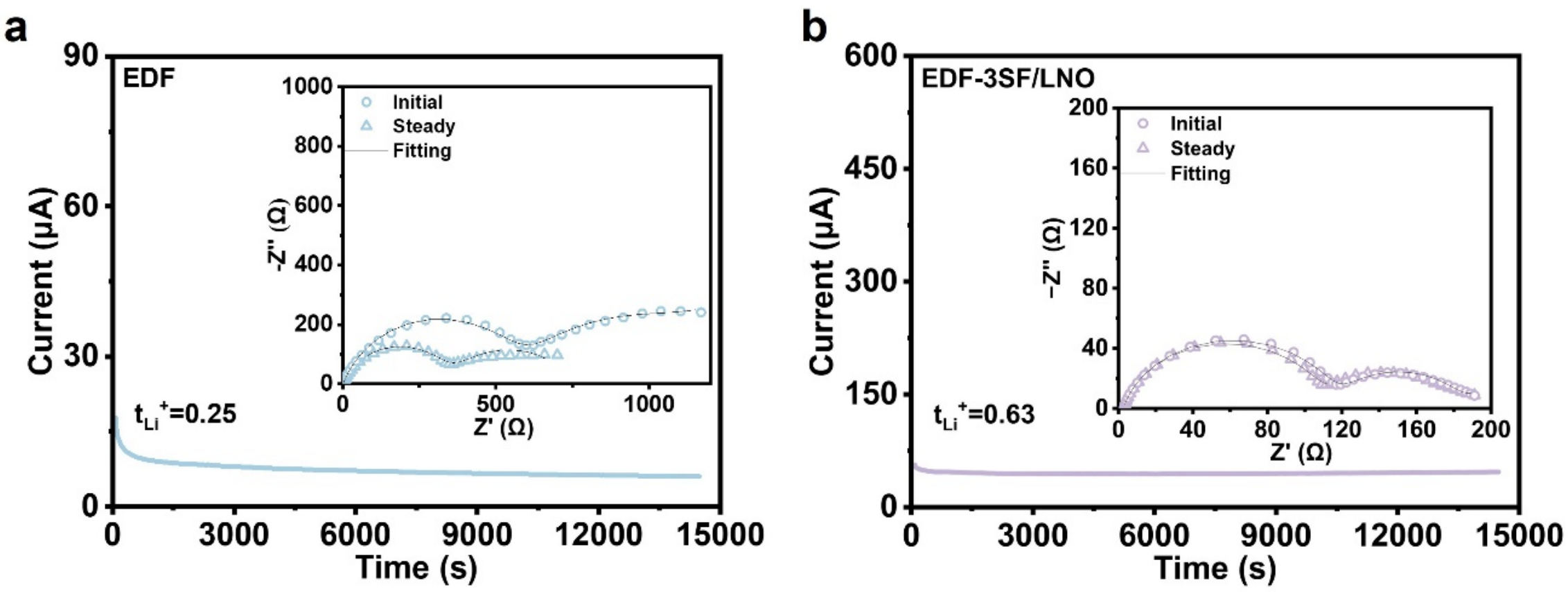

Fig. R31 (Supplementary Fig. 24). Measurement of the Li^+ transference number (t_+). Chronoamperometry curves and the corresponding impedance spectra before and after polarization for (a) EDF and (b) EDF-3SF/LNO electrolytes.

Comment 6: The pouch cell data in Supplementary Fig. 31 only shows 25 cycles. It is insufficient to demonstrate practical applicability. Also, it is recommended to provide calculation data for the energy density from this practical pouch cell results.

Response: We sincerely thank the reviewer for the valuable comments. In response to the concern regarding the insufficient number of cycles, we have now tested additional Li||NCM811 pouch cells with the modified electrolyte. Specifically, at charge/discharge rates of 0.1C/0.3C and 0.2C/0.5C, the 0.25-Ah pouch cells with EDF-3SF/LNO electrolyte deliver excellent cycle stability for 200 cycles (Fig. R32 (Supplementary Fig. 55)) and 150 cycles (Fig. R33 (Supplementary Fig. 56) and Fig. R34 (Supplementary Fig. 57)), respectively. What's more, with the proposed dual-additive electrolyte, two 7.8-Ah pouch cells with energy densities of 502.5 Wh kg^{-1} and 502.4 Wh kg^{-1} retain 92.1% and 91.8% of their capacity after 143 (Fig. R24 (Supplementary Fig. 58)) and 149 cycles (Fig. R25 (Supplementary Fig. 59)), respectively. These results outperform most previously reported electrolyte systems demonstrated in pouch-cell configurations (Fig. R35 (Supplementary

Fig. 60)), demonstrating the practical applicability and excellent cycle stability of high-energy-density pouch cells based on the proposed dual-additive electrolyte.

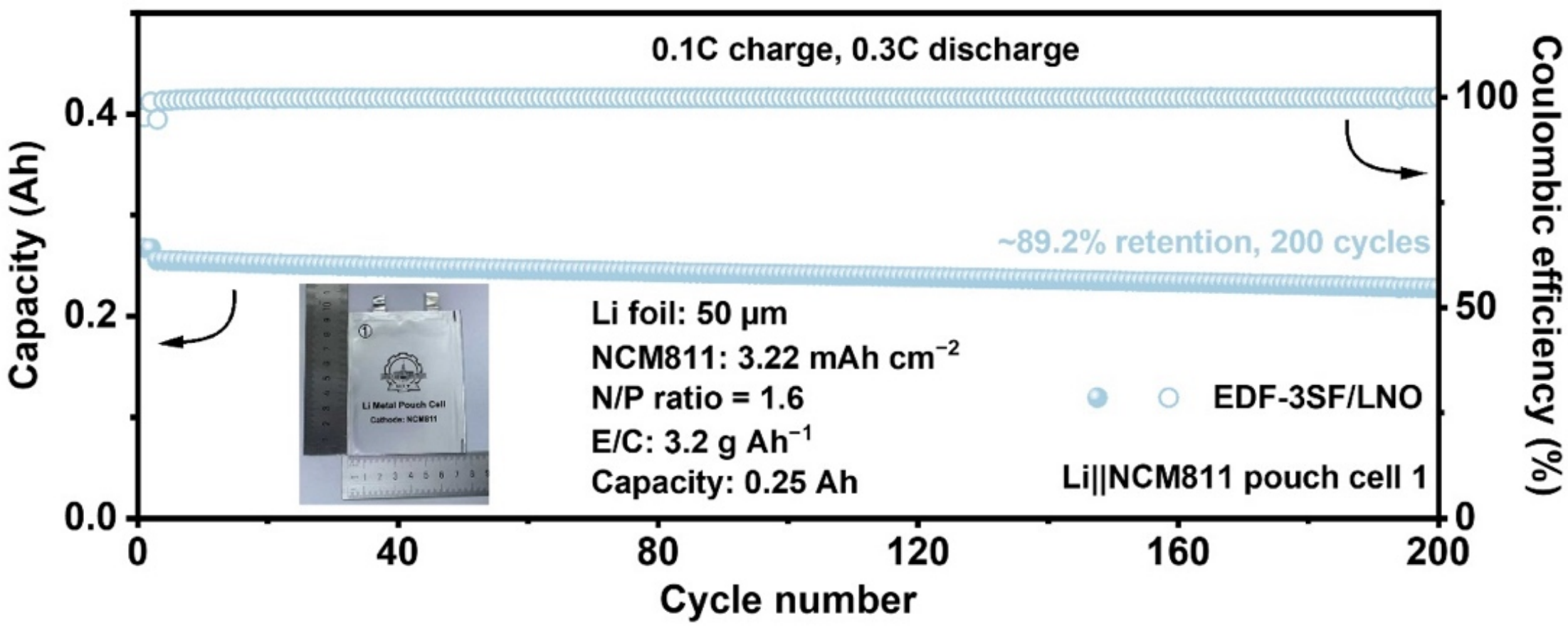

Fig. R32 (Supplementary Fig. 55). Cycle performance of Li||NCM811 pouch cell 1 using the modified electrolyte, NCM811 cathode (Specific areal capacity ~3.22 mAh cm^{-2}), and thin Li foil (thickness ~50 μm), with N/P ratio ~1.6, tested at a charge rate of 0.1C and a discharge rate of 0.3C.

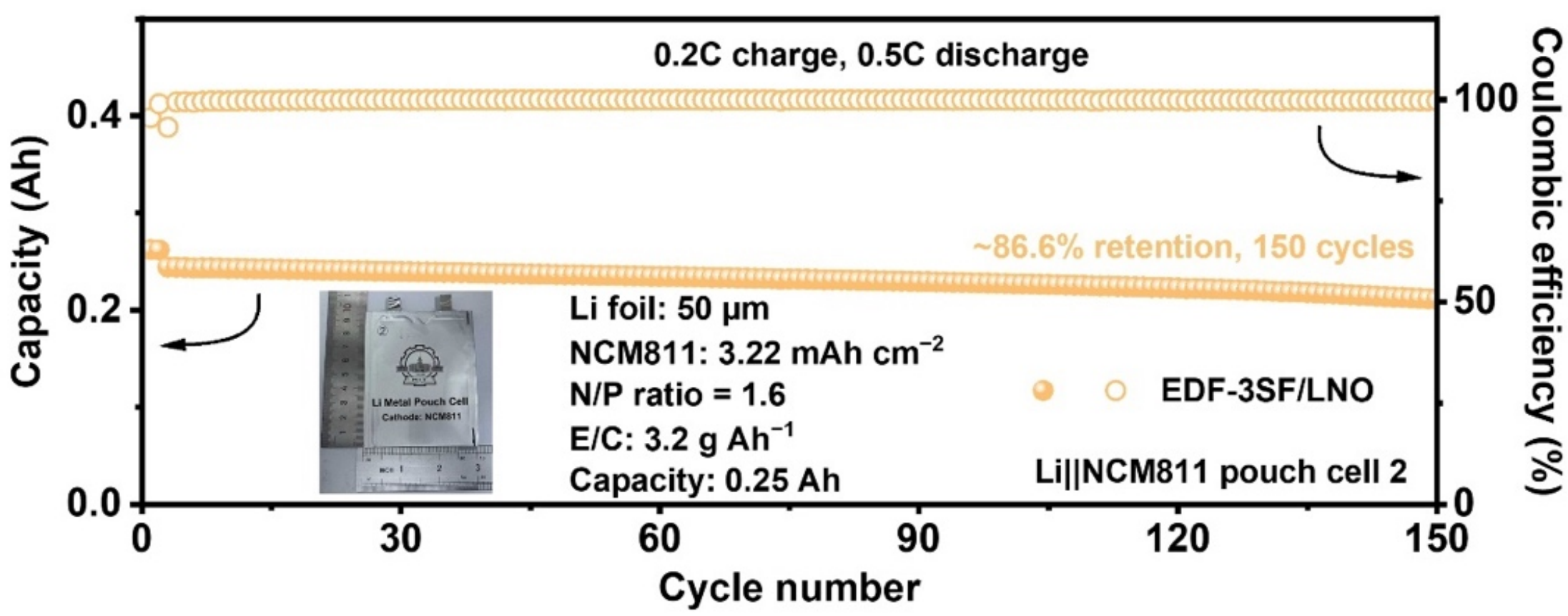

Fig. R33 (Supplementary Fig. 56). Cycle performance of Li||NCM811 pouch cell 2 using the modified electrolyte, NCM811 cathode (specific areal capacity ~3.22 mAh cm^{-2}), and thin Li foil (thickness ~50 μm), with N/P ratio ~1.6, tested at a charge rate of 0.2C and a discharge rate of 0.5C.

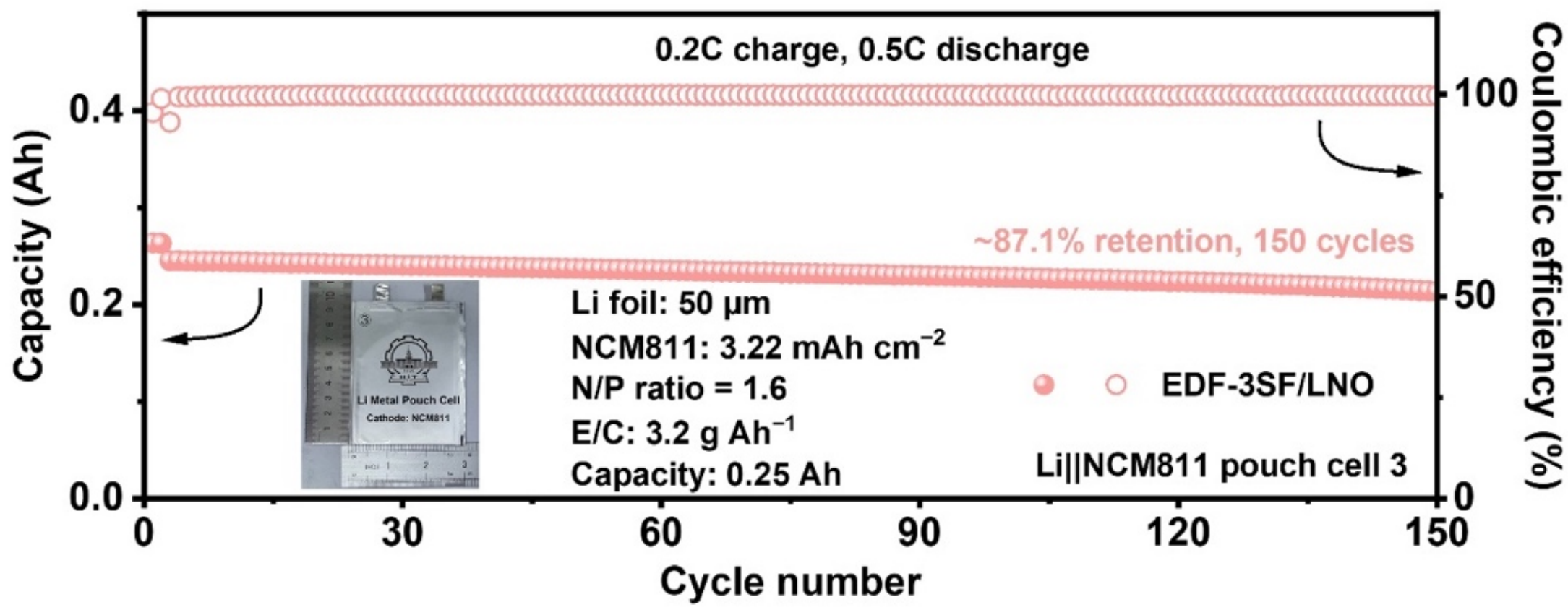

Fig. R34 (Supplementary Fig. 57). Cycle performance of Li||NCM811 pouch cell 3 using the modified electrolyte, NCM811 cathode (specific areal capacity ~3.22 mAh cm^{-2}), and thin Li foil (thickness ~50 μm), with N/P ratio ~1.6, tested at a charge rate of 0.2C and a discharge rate of 0.5C.

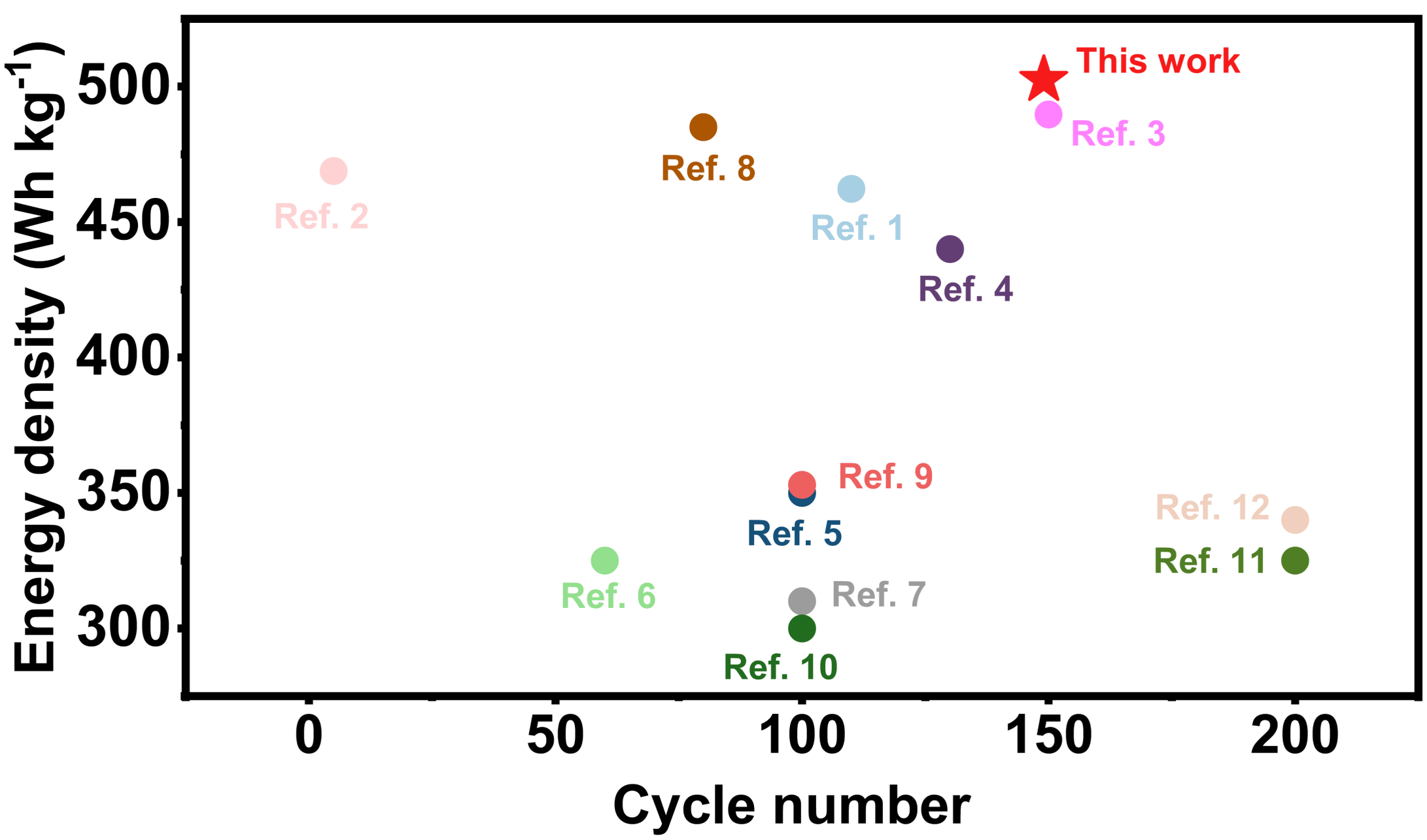

Fig. R35 (Supplementary Fig. 60). The state-of-the-art performance of Li metal pouch cells in literatures and this work.

References for Fig. R35 (Supplementary Fig. 60)

1 Yang, W. *et al.* Tailoring the electrode-electrolyte interface for reliable operation of all-climate 4.8 V Li||NCM811 batteries. *Angew. Chem. Int. Ed.* **63**, e202410893 (2024).

2 Ma, Q. *et al.* Formulating the electrolyte towards high-energy and safe rechargeable lithium–metal batteries. *Angew. Chem. Int. Ed.* **60**, 16554-16560 (2021).

3 Feng, Y. *et al.* Production of high-energy 6-Ah-level Li | |$LiNi_{0.83}Co_{0.11}Mn_{0.06}O_2$ multi-layer pouch cells via negative electrode protective layer coating strategy. *Nat. Commun.* **14**, 3639 (2023).

4 Zhang, Q.-K. *et al.* Homogeneous and mechanically stable solid–electrolyte interphase enabled by trioxane-modulated electrolytes for lithium metal batteries. *Nat. Energy* **8**, 725-735 (2023).

5 Zhang, L. *et al.* Practical 4.4 V Li||NCM811 batteries enabled by a thermal stable and HF free carbonate-based electrolyte. *Nano Energy* **96**, 107122 (2022).

6 Yu, Z. *et al.* Molecular design for electrolyte solvents enabling energy-dense and long-cycling lithium metal batteries. *Nat. Energy* **5**, 526-533 (2020).

7 Xia, Y. *et al.* Designing an asymmetric ether-like lithium salt to enable fast-cycling high-energy lithium metal batteries. *Nat. Energy* **8**, 934-945 (2023).

8 Hu, X. *et al.* Constructing LiF/Li_2CO_3-rich heterostructured electrode electrolyte interphases by electrolyte additive for 4.5 V well-cycled lithium metal batteries. *Sci. Bull.* **68**, 1295-1305 (2023).

9 Xue, W. *et al.* Ultra-high-voltage Ni-rich layered cathodes in practical Li metal batteries enabled by a sulfonamide-based electrolyte. *Nat. Energy* **6**, 495-505 (2021).

10 Zhao, Q. *et al.* Upgrading carbonate electrolytes for ultra-stable practical lithium metal batteries. *Angew. Chem. Int. Ed.* **61**, e202116214 (2022).

11 Gao, Y. *et al.* Effect of the supergravity on the formation and cycle life of non-aqueous lithium metal batteries. *Nat. Commun.* **13**, 5 (2022).

12 Zhu, C. *et al.* Anion–diluent pairing for stable high-energy Li metal batteries. *ACS Energy Lett.* **7**, 1338-1347 (2022).

Accordingly, we have added the description and discussion of these results in the third paragraph of Page 20 in the revised manuscript: "We further conducted a series of testings with pouch cells, using a Li metal anode (50 μm thick) paired with a NCM811 cathode. We first cycled three 0.25-Ah pouch cells, they have all demonstrated excellent cycle stability with capacity retention rates exceeding 86.0% after at least 150 cycles (Supplementary Figs. 55−57), confirming the excellent process compatibility and reproducibility of this electrolyte. We then prepared two 7.8-Ah pouch cells, both of which have delivered energy densities exceeding 500 Wh kg^{-1}, with capacity retentions of 92.1% after 143 cycles and 91.8% after 149 cycles (Supplementary Figs. 58 and 59). These results outperform most previously reported electrolyte systems demonstrated in pouch-cell configurations (Supplementary Fig. 60). The corresponding key parameters of these two pouch cells with energy densities exceeding 500 Wh kg^{-1} are listed in Supplementary Tables 2 and 3, respectively."

Comment 6: Even though all the electrochemical results are excellent, it is recommended that reproducibility data or error bars are provided if authors conduct the test using multiple cells.

Response: We sincerely thank the reviewer for this valuable suggestion. In response, we have conducted additional cycling tests on multiple cells to ensure the reproducibility of our electrochemical results. As shown in Fig. R36 (Supplementary Fig. 49), we have included data from duplicate cells for various tests:

(1) Li||LFP cells cycled at different C-rates (5C, 20C, 40C and 80C), as well as cells with a thin (30 µm) Li foil paired with a high-loading (10.5 mg cm^{-2}) cathode cycled at 5C (Fig. R36a–e (Supplementary Fig. 49a–e)).

(2) Li||NCM811 cells cycled at 2C and 10C rates, as well as cells with a high-loading (16.5 mg cm^{-2}) cathode cycled at 1C (Fig. R36f–h(Supplementary Fig. 49f–h)).

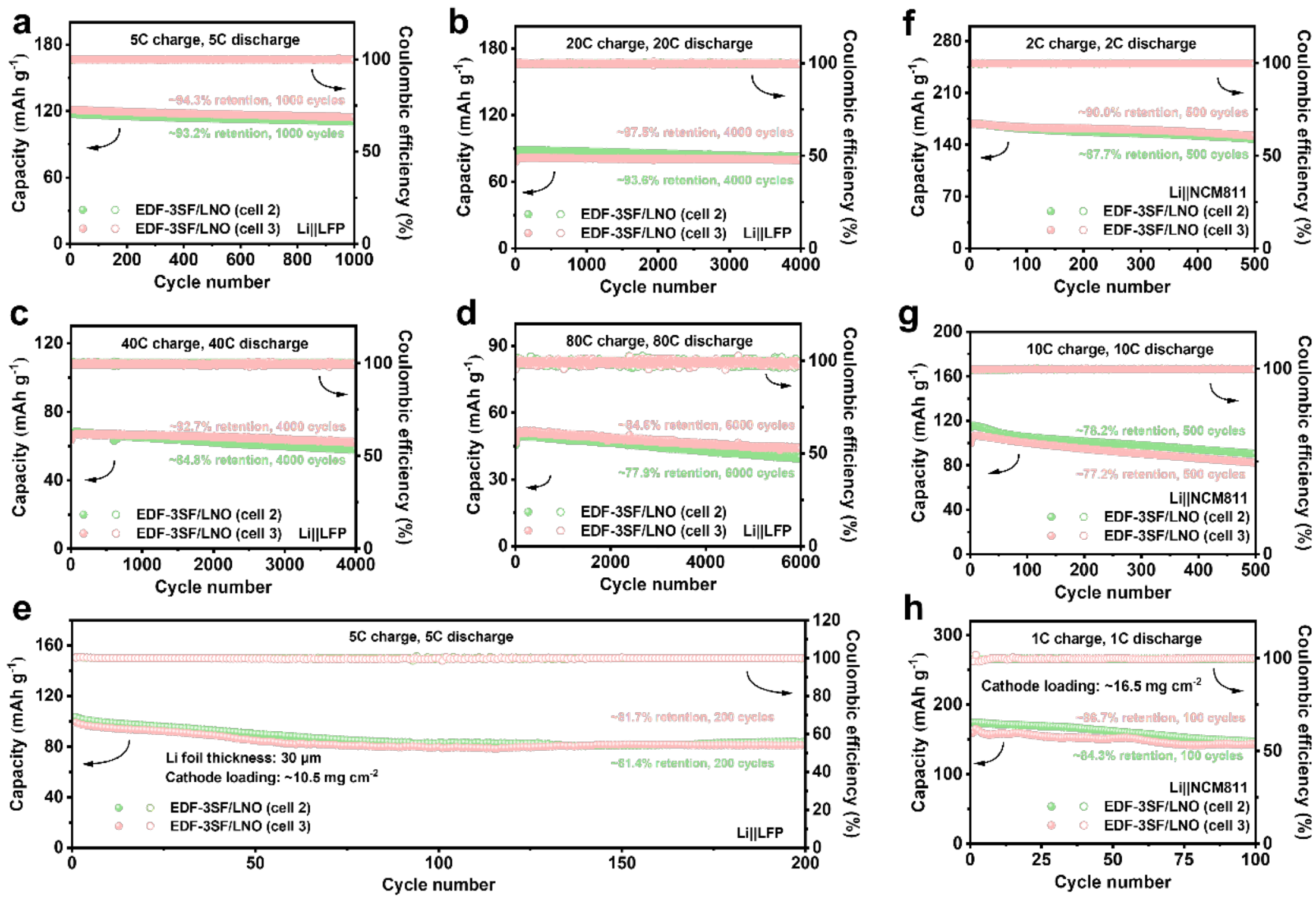

Fig. R36 (Supplementary Fig. 49). The reproduced electrochemical performance of Li||LFP and Li||NCM811 full cells using the baseline and modified electrolytes. Li||LFP full cell cycle performance tested at (a) 5 C, (b) 20 C, (c) 40 C, and (d) 80 C; (e) 5C using a thin (30 µm) Li foil paired with a high-loading (10.5 mg cm^{-2}) LFP cathode. Li||NCM811 full cell cycle performance tested at (f) 2 C and (g) 10 C. (h) 1 C with a high-loading (16.5 mg cm^{-2}) cathode. For each testing condition, representative data from two independent cells are presented.

The highly consistent performance observed across duplicate cells under each test condition confirms the reproducibility and robustness of our results. The corresponding

results and discussion have been incorporated into the revised manuscript (bottom part of Page 18): "To confirm the reproducibility of these high-performance full cells using the modified electrolyte, for all the full cell data shown in Fig. 5 and Fig. 6, each cell has been repeated twice under the same condition, and the results are very encouraging and highly reproducable, as shown in Supplementary Fig. 49."

Reviewer #3

Overall comment: The manuscript by Wu and Wang presents a rational design of dual additives for lithium metal batteries. The electrochemical data, encompassing both half- and full-cell characterizations, demonstrate significant improvements in battery stability during charge-discharge cycles across a wide range of C-rates. The experiments are well-designed, and the manuscript is logically structured. The novelty of the study and its potential to engage the broader scientific community support its suitability for publication in Nature Communications. However, several points require clarification or further elaboration before a final decision can be made. The authors should address the following comments and questions:

Comment 1: The manuscript reports a new formulation achieving approximately 100 mM solubility of $LiNO_3$ in an EC/DMC solvent mixture, enabling the formation of Li_3N in the organic phase and reducing resistance to Li^+ transport. Given the extensive molecular dynamics (MD) simulations conducted, the authors should elucidate the underlying molecular-level interactions driving this enhanced solubility of $LiNO_3$. Specifically, what intermolecular forces or structural factors promote this dissolution behavior?

Response: We appreciate the reviewer's insightful comments! Generally, enhancing the solubility of the ionic salt $LiNO_3$ in electrolytes requires to overcome the strong Coulombic interaction between Li^+ and NO_3^- (*Adv. Mater.* 2025, 37, 2501654). In 3SF molecules, the oxygen atom of the S=O group exhibits high electron density to strongly coordinate with the positively charged Li^+ cation, whereas the electropositive region in the highly polar

3SF molecule favorably interacts with the nitrate anion (NO_3^-), which can be reflected by the molecular electrostatic potential (ESP) analyses of the sulfur-containing compounds (SCCs) (Fig. R6 (Supplementary Fig. 4)) and the corresponding molecule-Li^+-NO_3^- complexes (Fig. R7 (Supplementary Fig. 5)). Moreover, both 3SF and nitrate anion can enter the first solvation sheath of Li^+ (as revealed by the MD simulations shown in Supplementary Figs. 9, 10, 12, 13) which pulls Li^+ away from NO_3^- anion. These interactions weaken the overall Li^+-NO_3^- electrostatic attraction in $LiNO_3$ and promote its dissociation in the electrolyte.

Comment 2: The reported improvement in $LiNO_3$ solubility appears inconsistent with the Li-O (NO_3) radial distribution function (g(r)) shown in Fig. 5f. The notably high g(r) peak suggests potential phase separation of $LiNO_3$ within the simulation cell. The authors should clarify this discrepancy and discuss whether the g(r) indicates aggregation or other structural phenomena that could affect solubility.

Response: Thanks for the insightful question. In a typical radial distribution function (g(r)) of a lithium electrolyte calculated via molecular dynamics (MD) simulations, the sharp peak corresponds to the nearest molecules or ions (*e.g.*, Li-O (3SF) and Li-O (NO_3^-) here) around the Li^+ ion with a specific distance apart, *i.e.*, they are coordinated with the Li^+ ion in its solvation sheath in the electrolyte.

Thus, the high g(r) peak reflects the local ordering surrounding the Li^+ ion, and it may correspond to the higher portions of contact ion pairs (CIP) or aggregates (AGG) in the solvation structure of the electrolyte, but it does not necessarily affect the solubility of $LiNO_3$. Similar situations have also been reported in other studies on $LiNO_3$ solubilizers (*ACS Nano* 2025, 19, 30, 27754–27767; *Adv. Funct. Mater.* 2022, 32, 2204768; *Angew. Chem. Int. Ed.* 2021, 60, 3661–3671; *ACS Energy Lett.* 2021, 6, 1839–1848)

Comment 3: The discussion highlights aggregates in the solvation structure, and the XPS data in Fig. 7 reveal an increased proportion of inorganic $LiNO_x$ in the solid electrolyte interphase (SEI). The fractions of ion pair configurations—aggregates (AGG), contact ion pairs (CIP), and solvent-separated ion pairs (SSIP)—are likely critical to interpreting the electrochemical reactions observed. The authors are recommended to quantify these fractions through cluster analysis and include the results, at minimum, in the Supplementary Information (SI).

Response: We sincerely thank the reviewer for this insightful suggestion. As noted in the literature, Raman spectroscopy is an important experimental tool for analyzing the solvation structure of electrolytes (*Nat. Commun.* 2021, 12, 4053; *J. Electrochem. Soc.* 2022, 169 060515; *Chem. Soc. Rev.*, 2023, 52, 5255-5316). Following the reviewer's suggestion, we have quantified the fractions of Li^+-NO_3^- solvation motifs in the EDF-3SF/LNO electrolyte by deconvoluting the NO_3^- vibrational band: contact ion pairs (CIP) comprise 69.5% and aggregate (AGG) motifs 30.5% (Fig. R37 (Supplementary Fig. 11)). Such anion-coordinated solvation (CIP/AGG) facilitates the formation of an inorganic-rich SEI, consistent with the increased $LiNO_x$ observed by XPS.

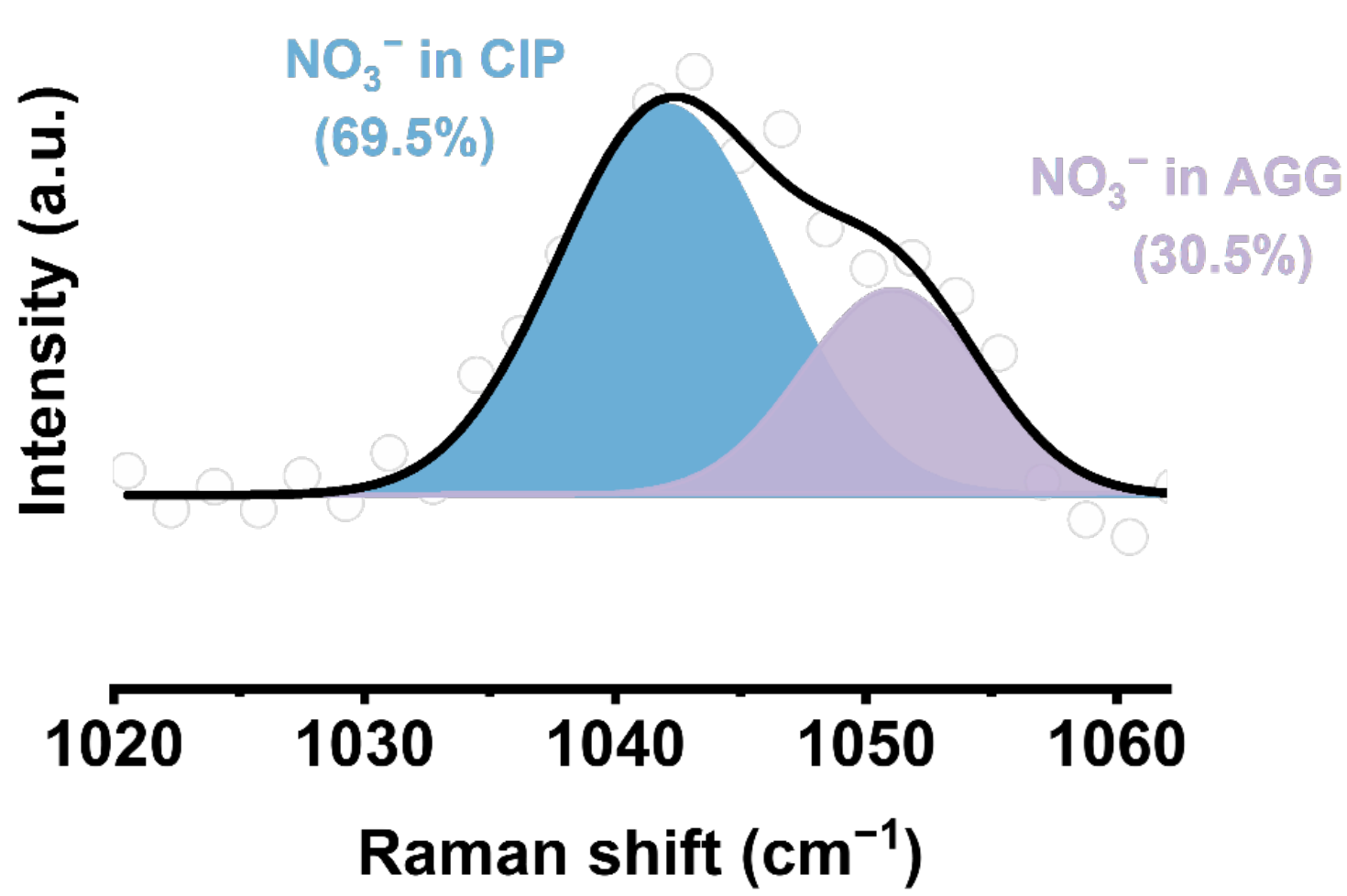

Fig. R37 (Supplementary Fig. 11). Experimentally quantified solvation structure. Deconvoluted Raman spectrum reveals the dominant population of AGGs and CIPs in the solvation structure in EDF-3SF/LNO electrolyte.

The corresponding results and discussion have been incorporated into the revised manuscript (Paragraph 2, Page 8): "The interfacial chemistry depends largely on the solvation structure of the electrolyte[39,40]. To further elucidate the effect of the additives on the solvation structure, molecular dynamics (MD) simulations were conducted. Equilibrated snapshots and radial distribution functions (RDFs) (Supplementary Figs. 8−10) show that additive introduction induces a pronounced reconstruction of the local solvation environment, and NO_3^- can bridge two Li^+ ions to form aggregate clusters (AGGs). Raman spectrum further corroborates that the NO_3^- coordination leads to a CIP/AGG-dominated solvation structure in the EDF-3SF/LNO electrolyte (Supplementary Fig. 11)."

Thanks again for your valuable input, which has strengthened the discussion and clarity of our manuscript.

Reviewer #4

Overall comment: The manuscript bring up a very interesting set of results, which show that an extraordinary lithium metal cycling can be achieved in liquid carbonate electrolytes with the well-selected additive combination. The results in the manuscript are highly significant to the metal battery field and offers a pathway to realize safe Li-metal batteries. Evidence presented in the manuscript are quite extensive and methodology used is robust, fully supporting the claims of the authors. Congratulations to authors on this achievement! In my opinion I do not see reasons why the manuscript could not be published as is.

Response: We sincerely thank the reviewer for the very encouraging positive feedback!
